# Supplementary figures and images for: Alkenyl oxindole is a novel PROTAC moiety that recruits the CRL4DCAF11 E3 ubiquitin ligase complex for targeted protein degradation
Source: PLoS Biol. 2024 May 20;22(5):e3002550. doi: 10.1371/journal.pbio.3002550 (PMC11104598; doi:10.1371/journal.pbio.3002550)

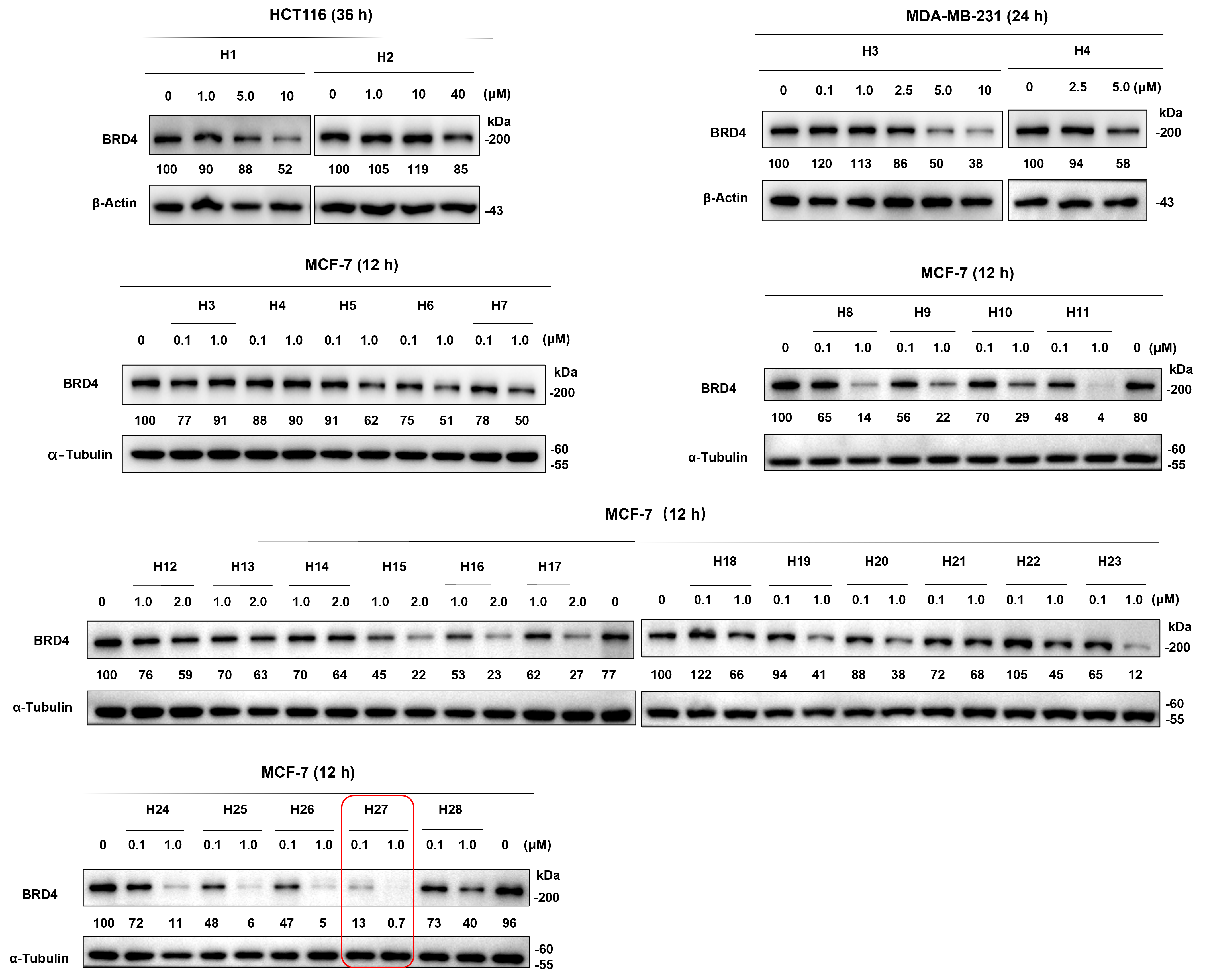

Supplement: S1 Fig — Western blotting results for BRD4 degradation. Image J was employed for relative quantitative analysis. The raw images for WB in the figure can be found in S1 Raw Images. (TIF) [file pbio.3002550.s001.tif]

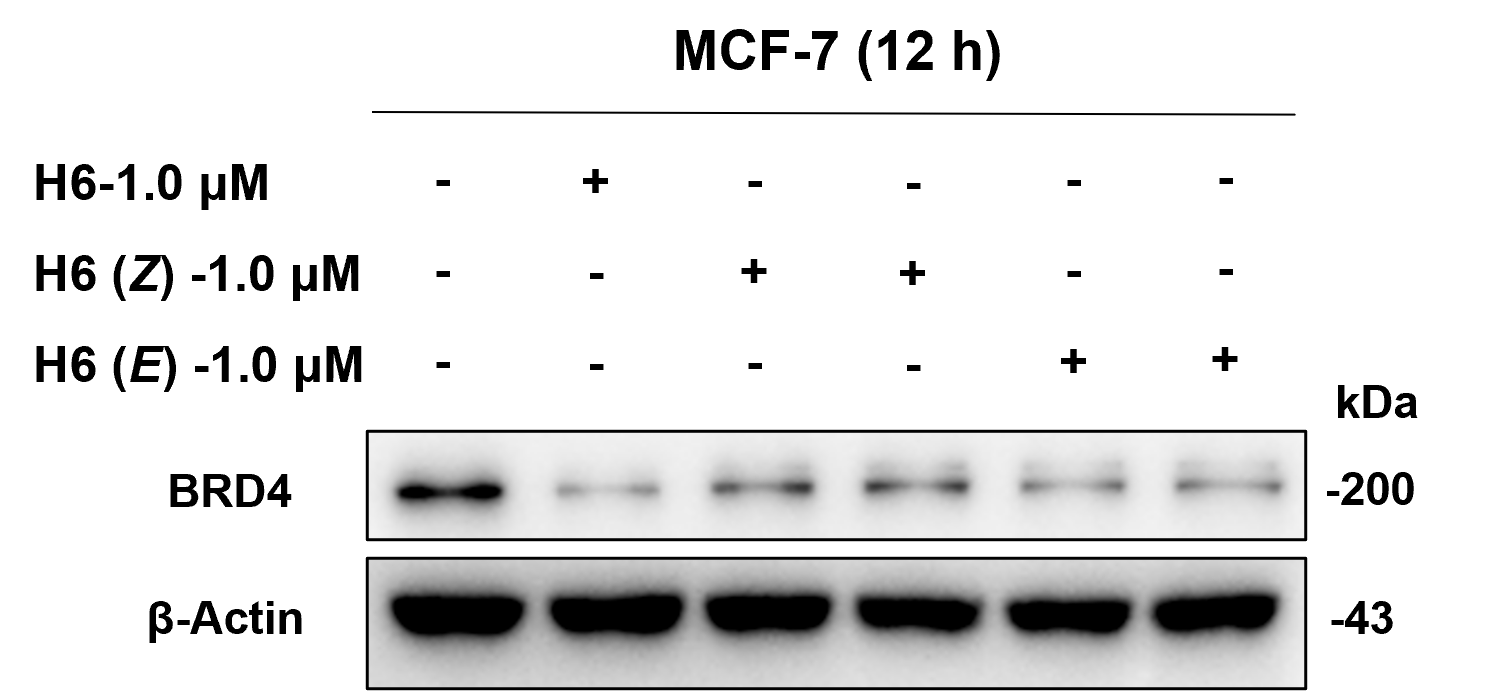

Supplement: S2 Fig — The raw images for WB in the figure can be found in S1 Raw Images. (TIF) [file pbio.3002550.s002.tif]

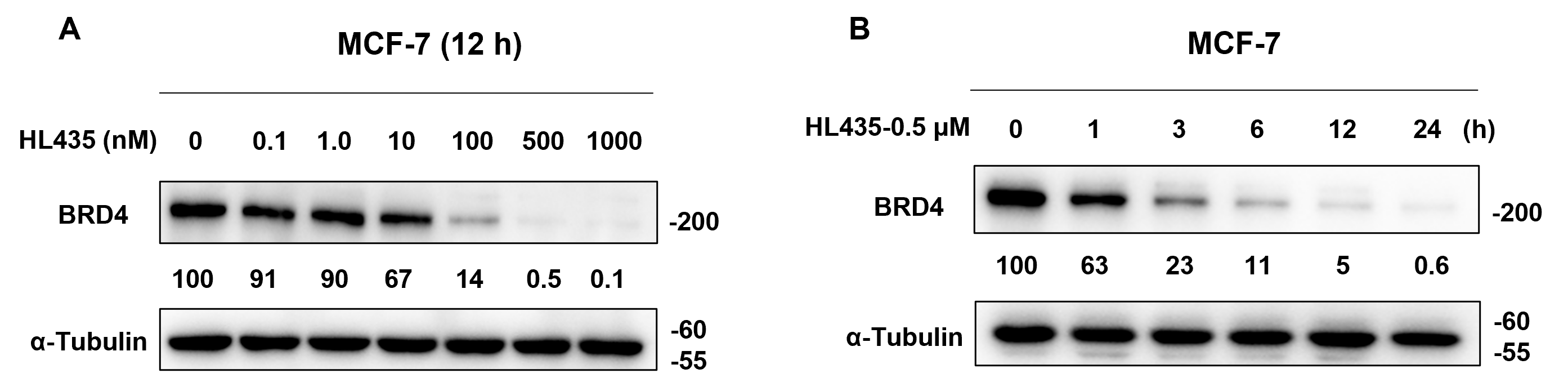

Supplement: S3 Fig — (A) Representative WB results for concentration-dependent studies of BRD4 degradation. (B) Representative WB results for kinetics studies of BRD4 degradation. Image J was employed for relative quantitative analysis. The raw images for WB in the figure can be found in S1 Raw Images. (TIF) [file pbio.3002550.s003.tif]

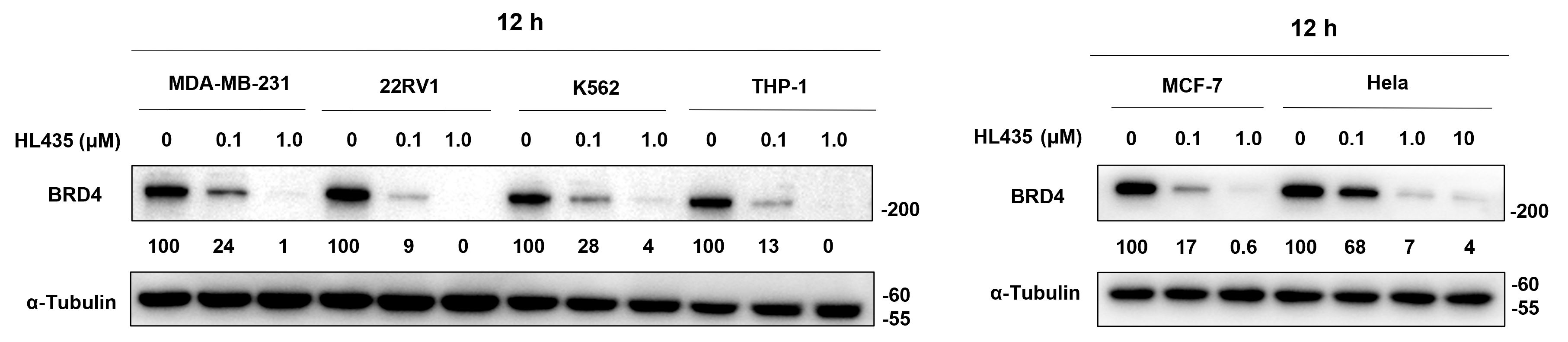

Supplement: S4 Fig — WB results for BRD4 degradation. Image J was employed for relative quantitative analysis. The raw images for WB in the figure can be found in S1 Raw Images. (TIF) [file pbio.3002550.s004.tif]

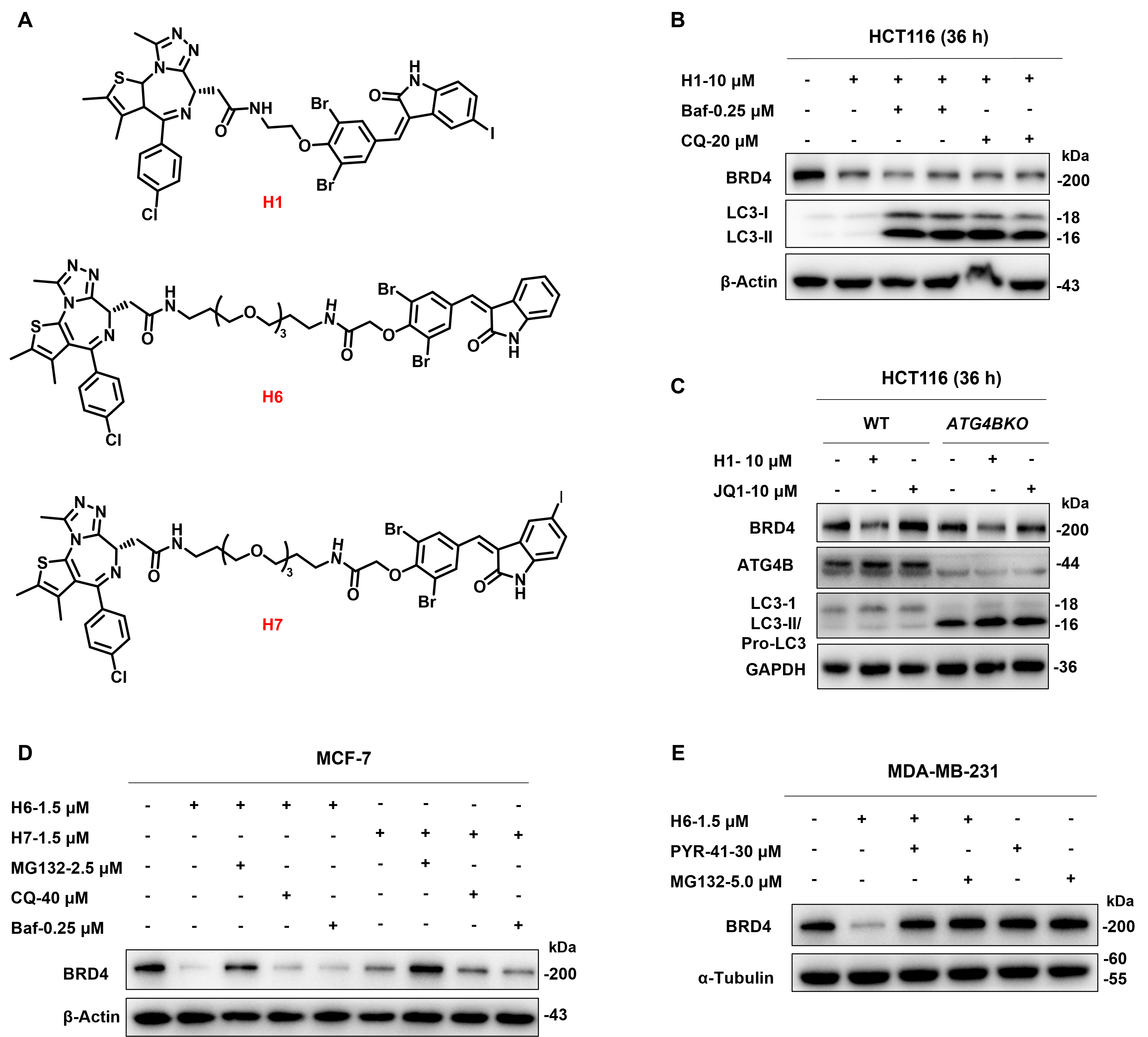

Supplement: S5 Fig — (A) Structure of compounds conjugated alkenyl oxindoles. (B) Autophagy-lysosome inhibitor CQ or Baf cannot rescue the degradation of BRD4 by H1. (C) H1 degraded BRD4 independent of LC3 and autophagosomes. (D) Proteasome inhibitor MG132 but not autophagy inhibitor CQ or Baf could rescue the degradation of BRD4 by H6 or H7. Cells were pretreated with MG132, CQ, or Baf for 2 hours, followed by H6 or H7 treatment for 6 hours. (E) Inhibitors of the ubiquitin-protease system could rescue the degradation of BRD4 by H6. Cells were pretreated with PYR-41 or MG132 for 2 hours, followed by H6 treatment for 6 hours. The raw images for WB in the figure can be found in S1 Raw Images. (TIF) [file pbio.3002550.s005.tif]

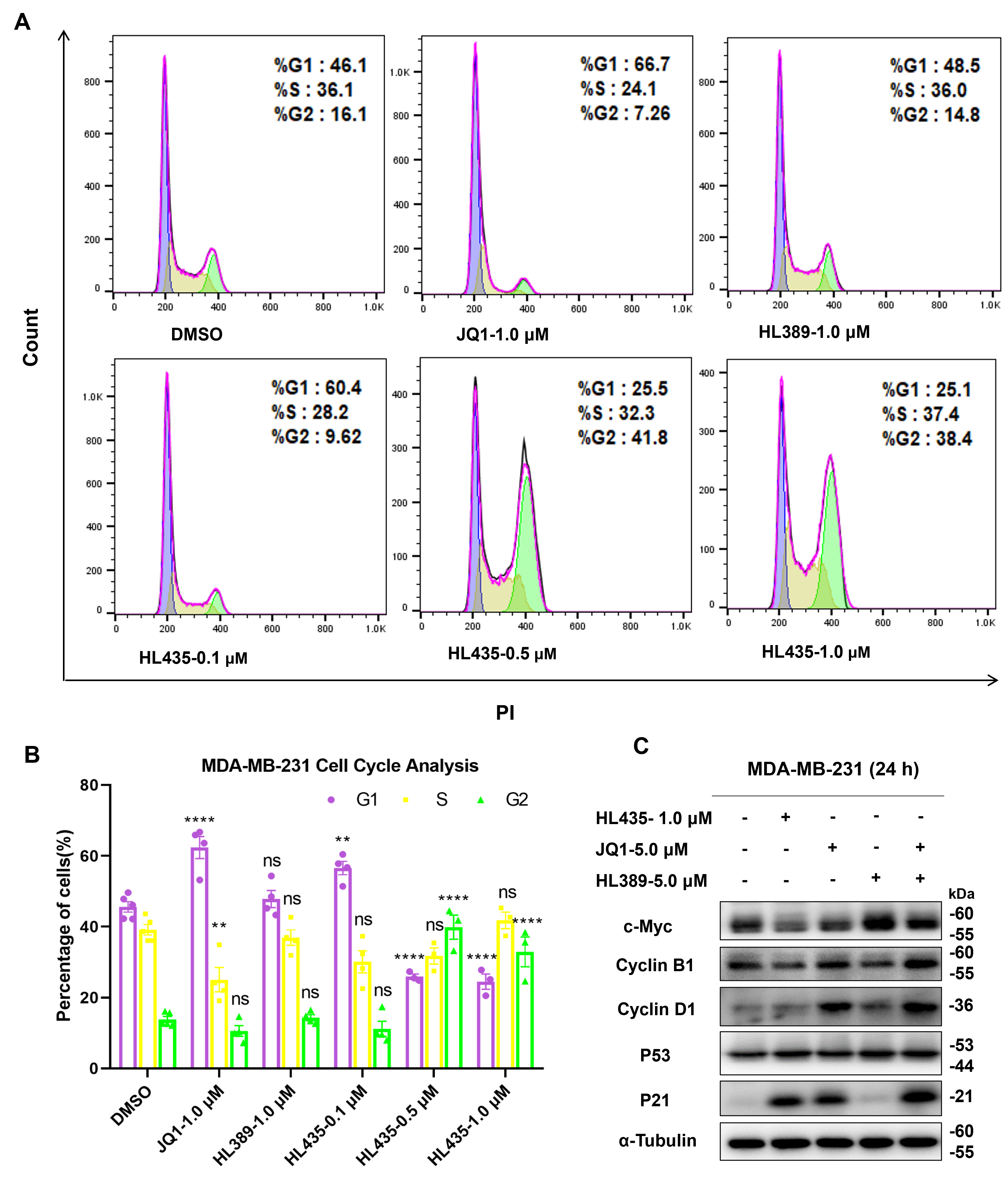

Supplement: S6 Fig — (A) Representative flow cytometry analysis results of the cell cycle. MDA-MB-231 cells were treated with indicated compounds for 24 hours before stained with PI. (B) Quantitative statistical analysis of cell cycle for A. (C) Representative WB results of cycle relevant proteins in MDA-MB-231 cells. The data underlying the graphs in the figure can be found in S5 Data; the raw images for WB in the figure can be found in S1 Raw Images. Data were presented as mean ± SEM (n = 3). Statistical significance was determined by one-way ANOVA. **P < 0.01, ***P < 0.001, ****P < 0.0001; ns, no statistical significance. (TIF) [file pbio.3002550.s006.tif]

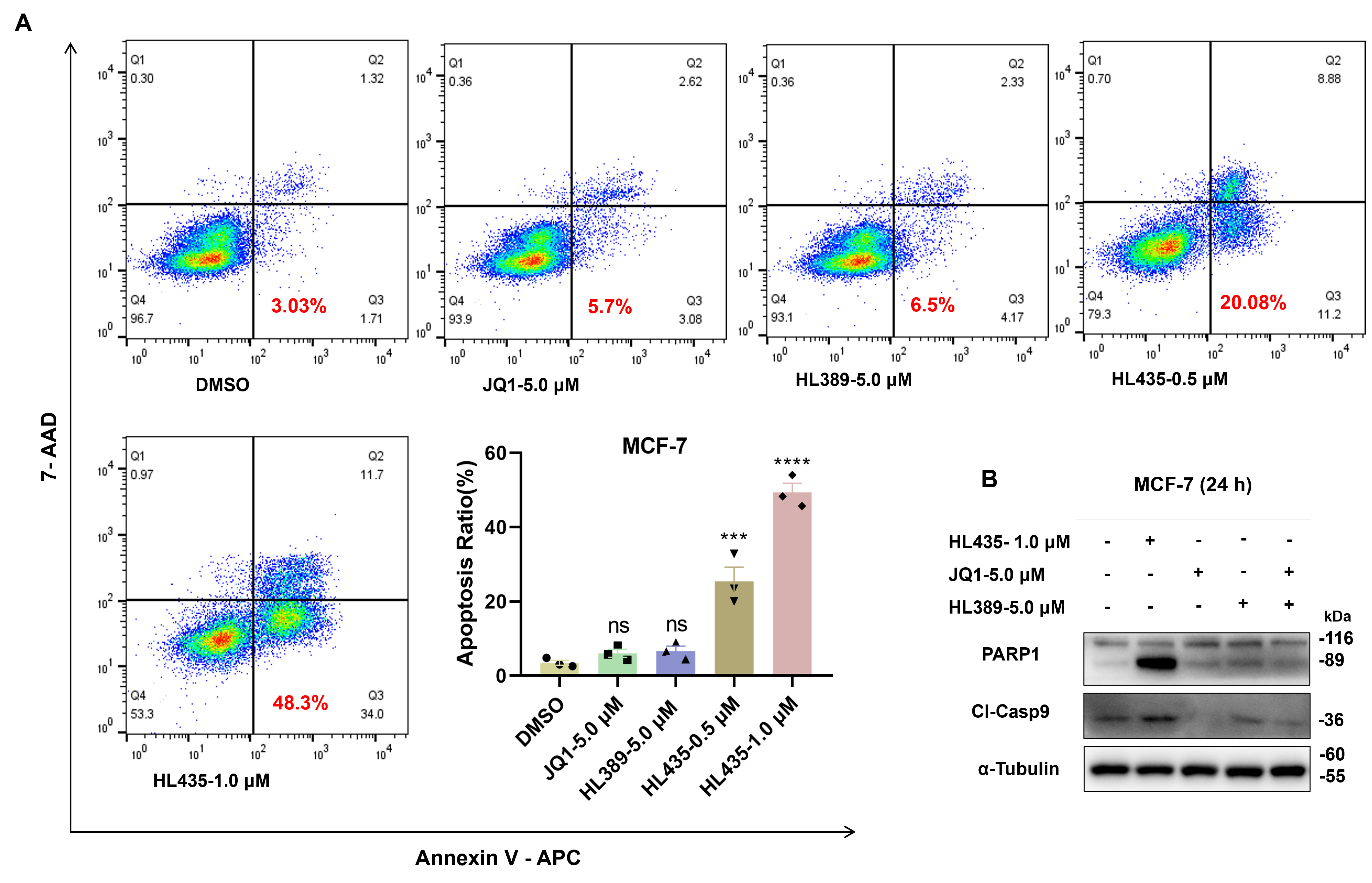

Supplement: S7 Fig — (A) Representative flow cytometry analysis results and quantitative statistical analysis of apoptosis; MCF-7 cells were treated with indicated compounds for 36 hours before stained with an 7AAD/APC Apoptosis Detection kit. (B) Representative WB results of apoptosis-relevant proteins. The data underlying the graphs in the figure can be found in S5 Data; the raw images for WB in the figure can be found in S1 Raw Images. Data were presented as mean ± SEM (n = 3). Statistical significance was determined by one-way ANOVA. ***P < 0.001, ****P < 0.0001; ns, no statistical significance. (TIF) [file pbio.3002550.s007.tif]

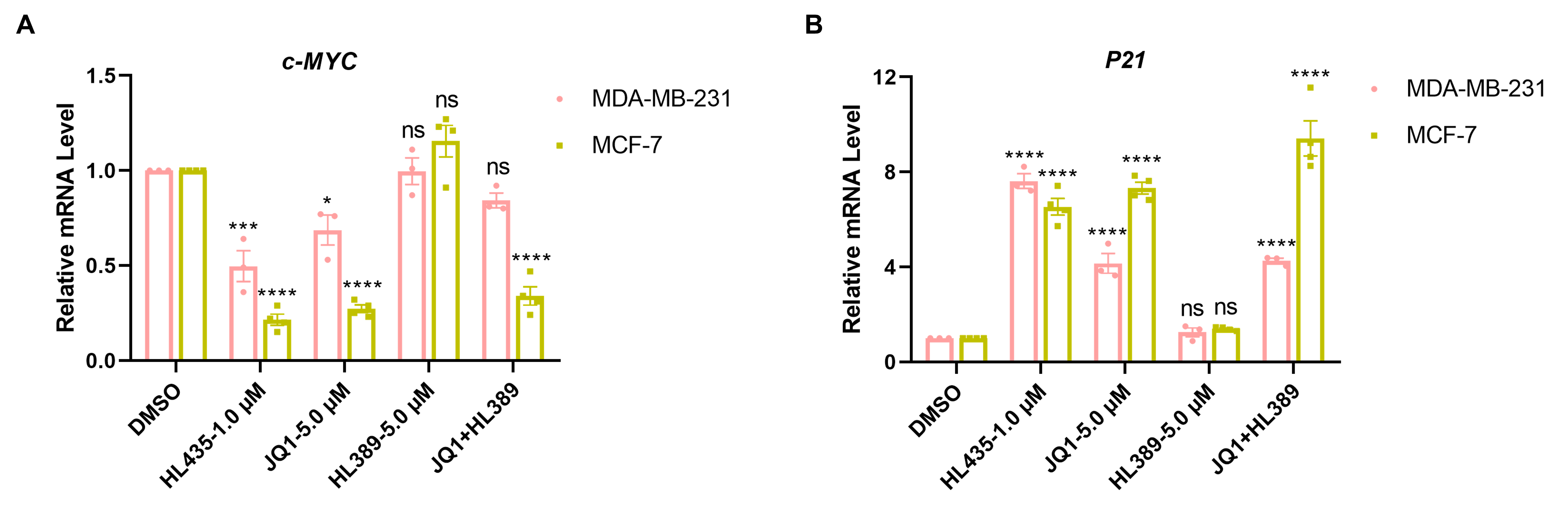

Supplement: S8 Fig — Cells were treated with indicated compounds for 12 hours; GAPDH was used as internal reference. The data underlying the graphs in the figure can be found in S5 Data. Data were presented as mean ± SEM. Statistical significance was determined by one-way ANOVA. *P < 0.05, ***P < 0.001, ****P < 0.0001; ns, no statistical significance. (TIF) [file pbio.3002550.s008.tif]

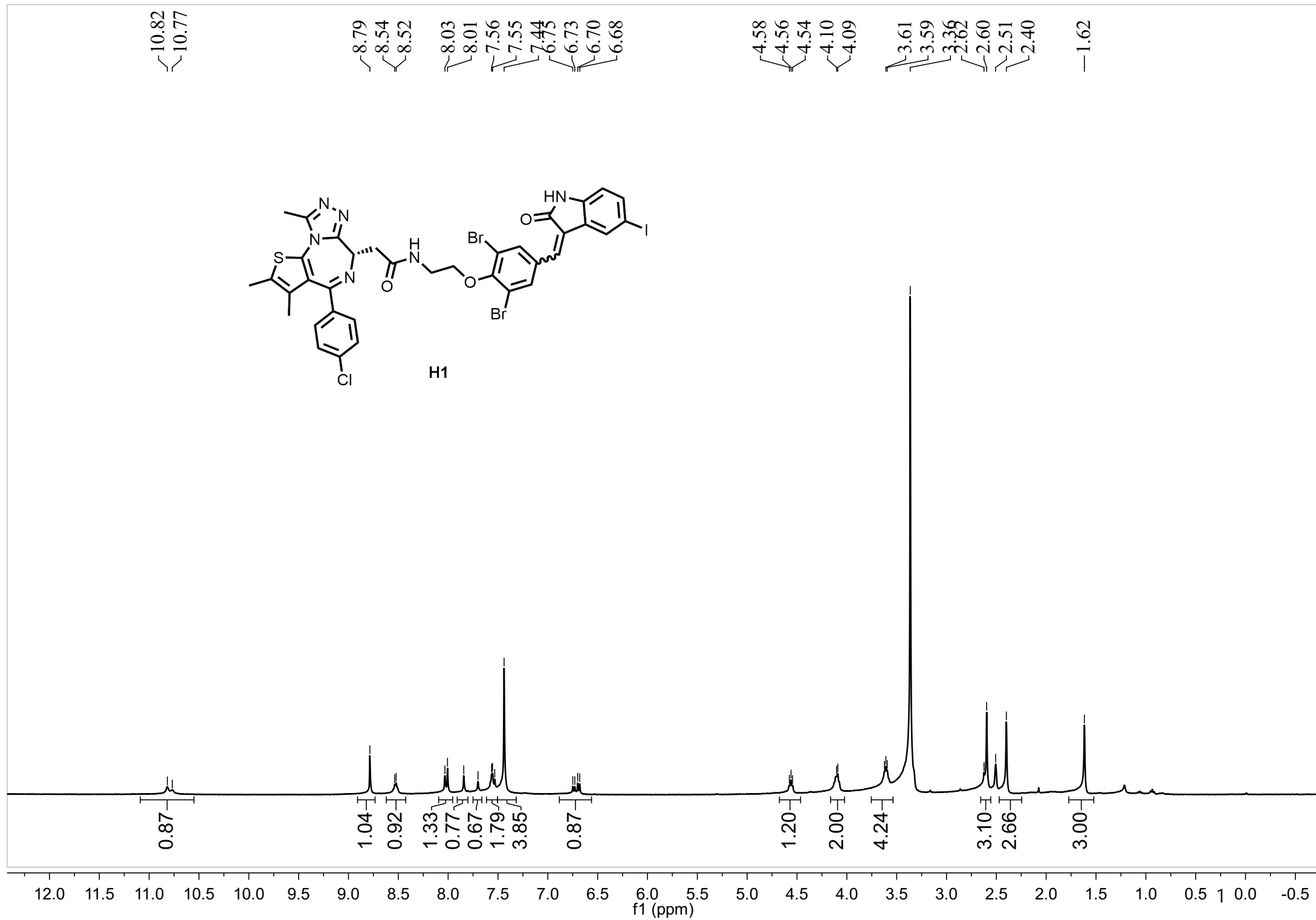

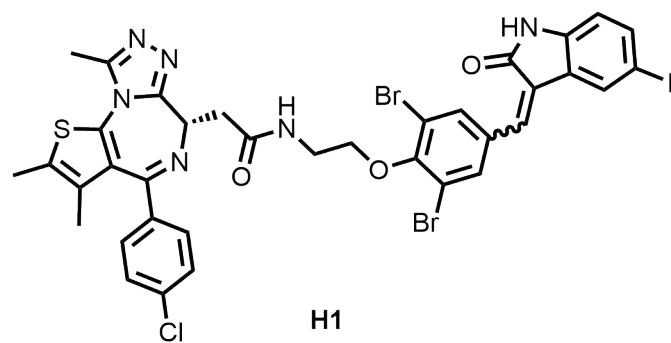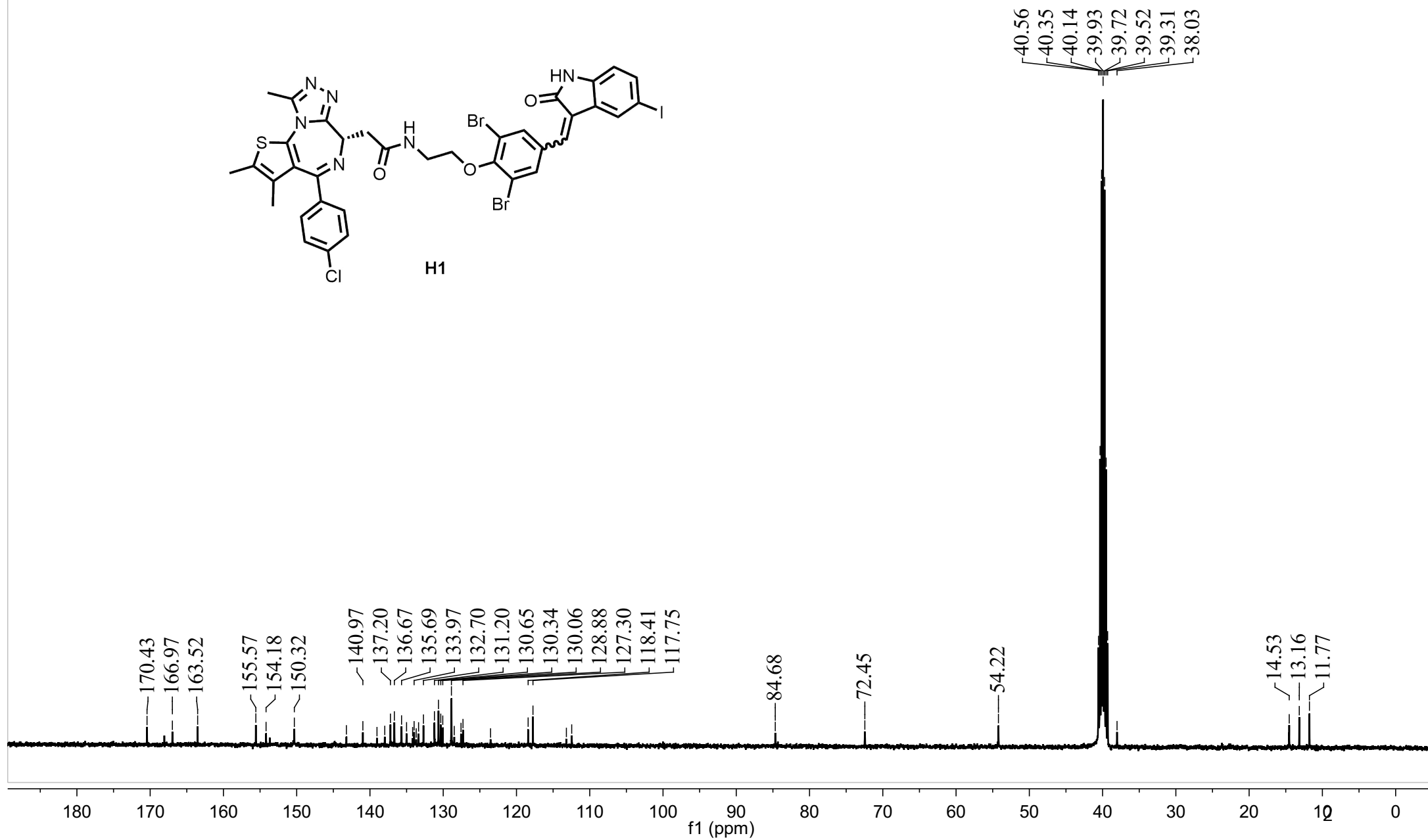

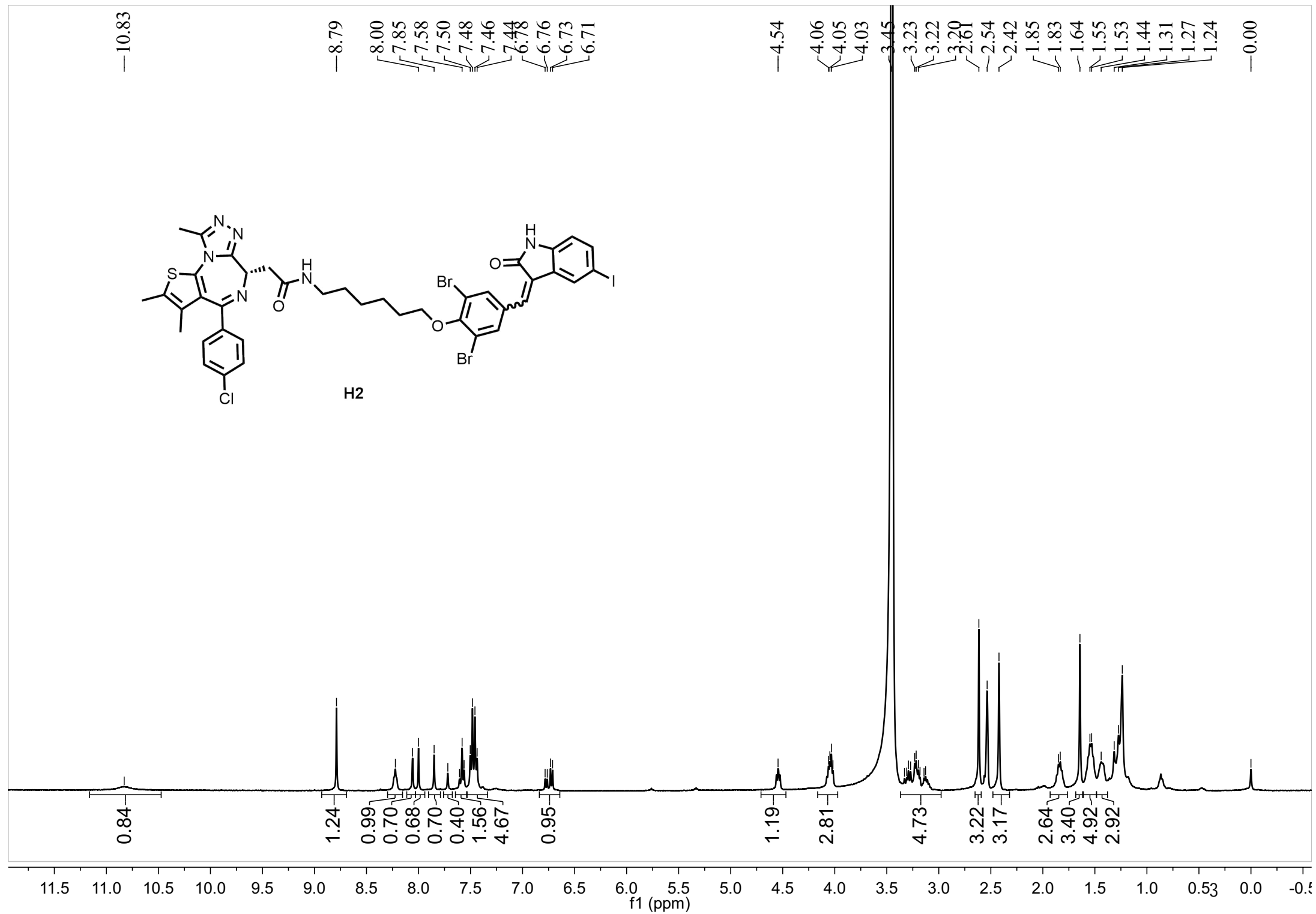

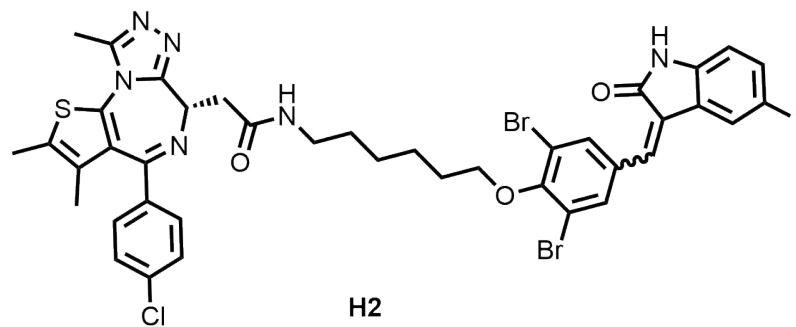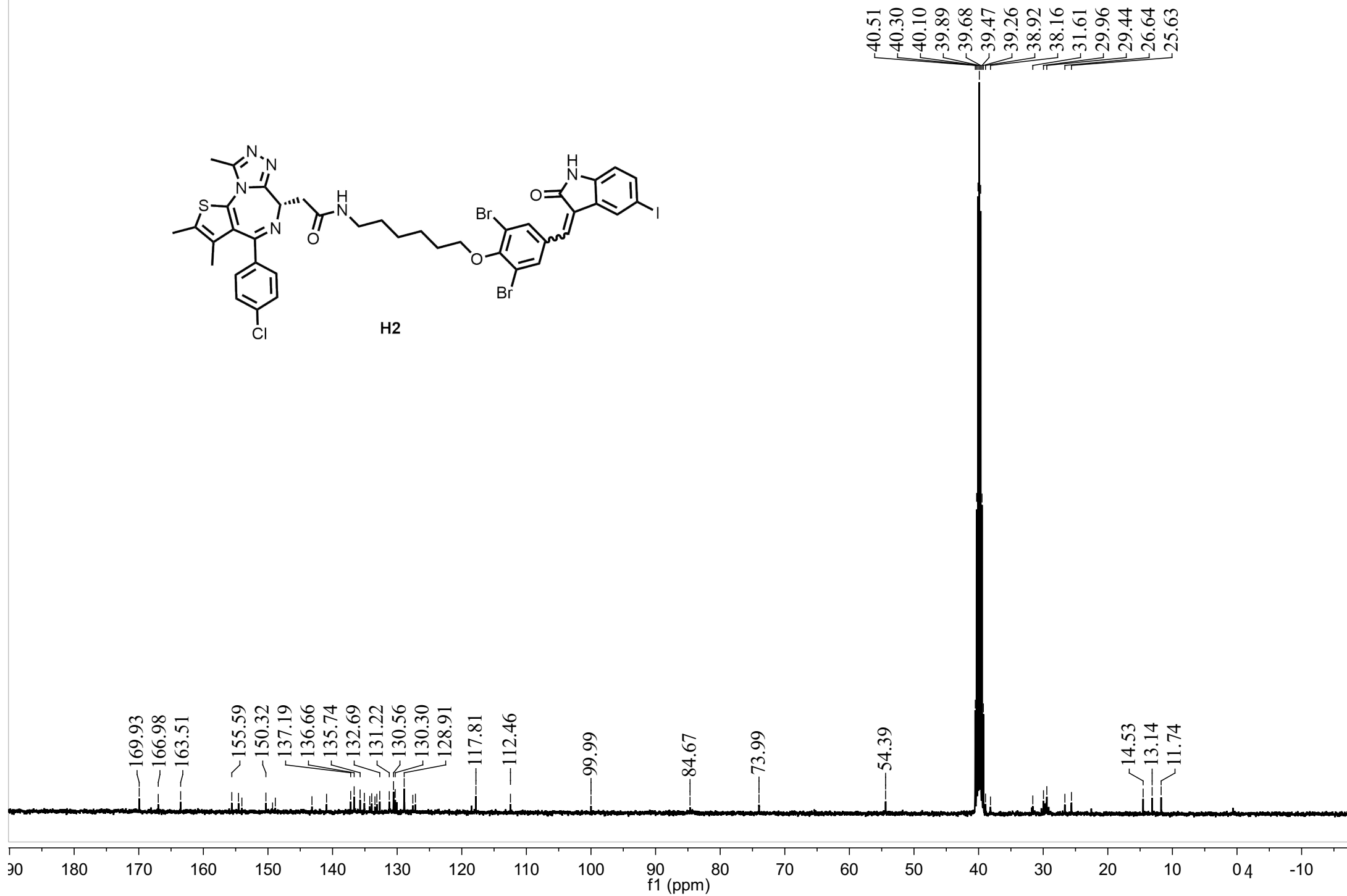

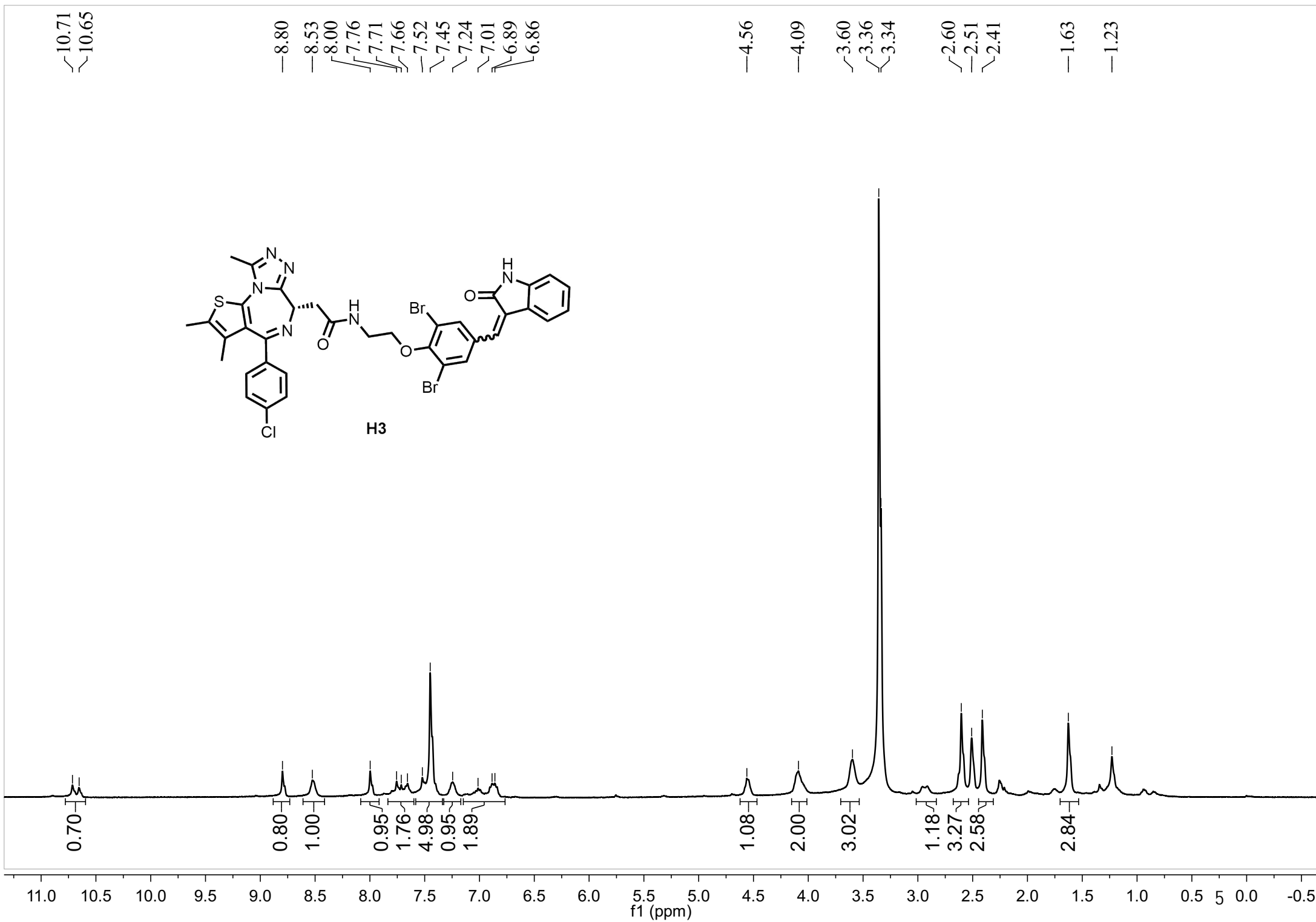

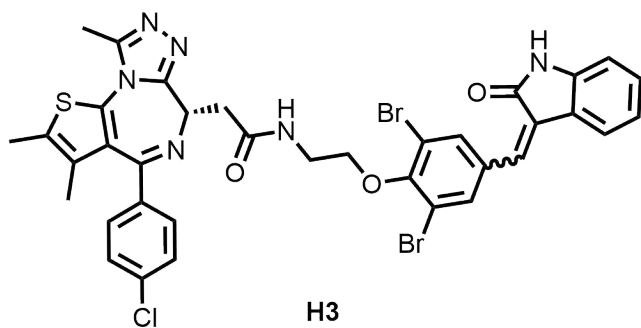

H3

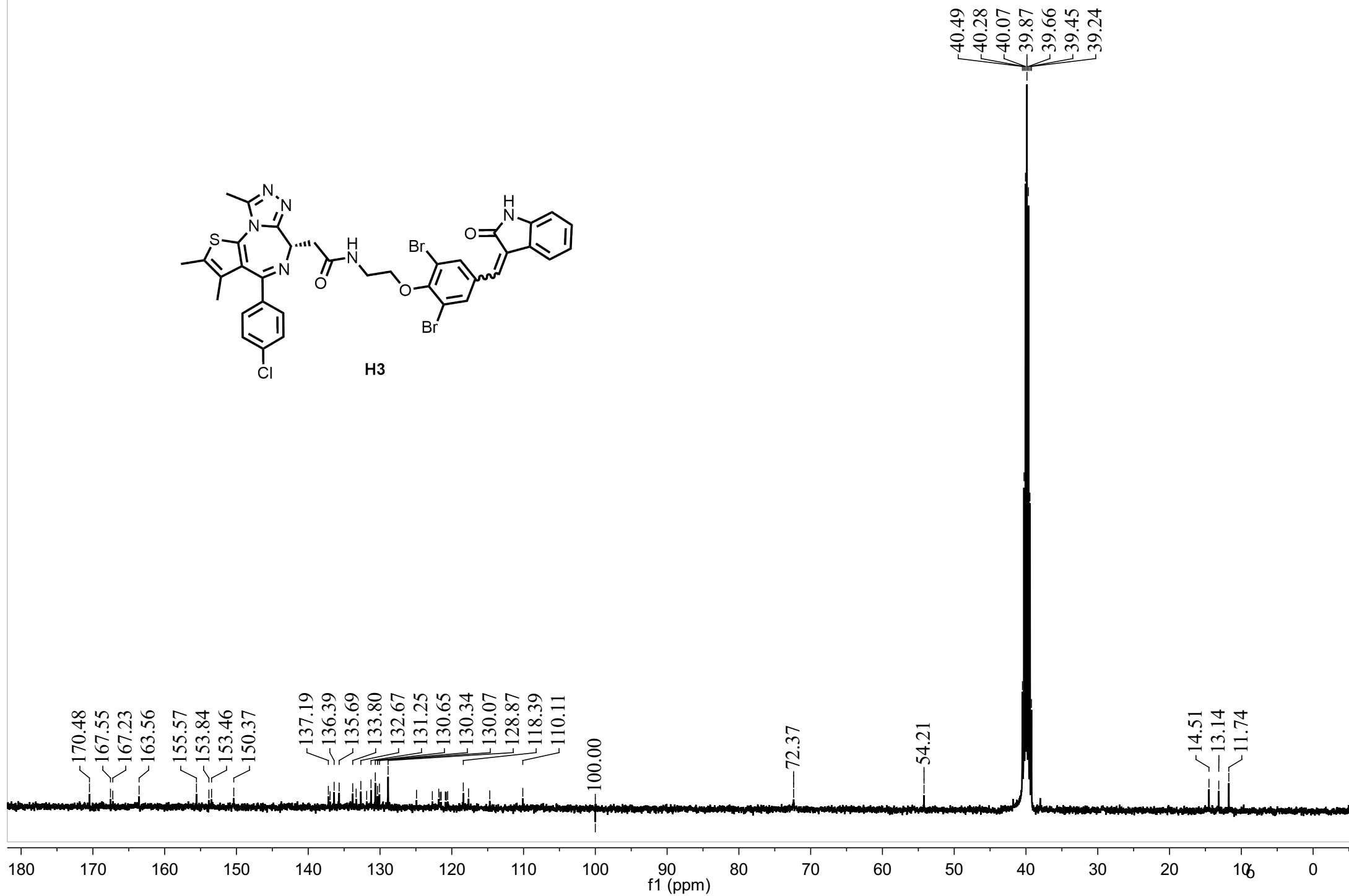

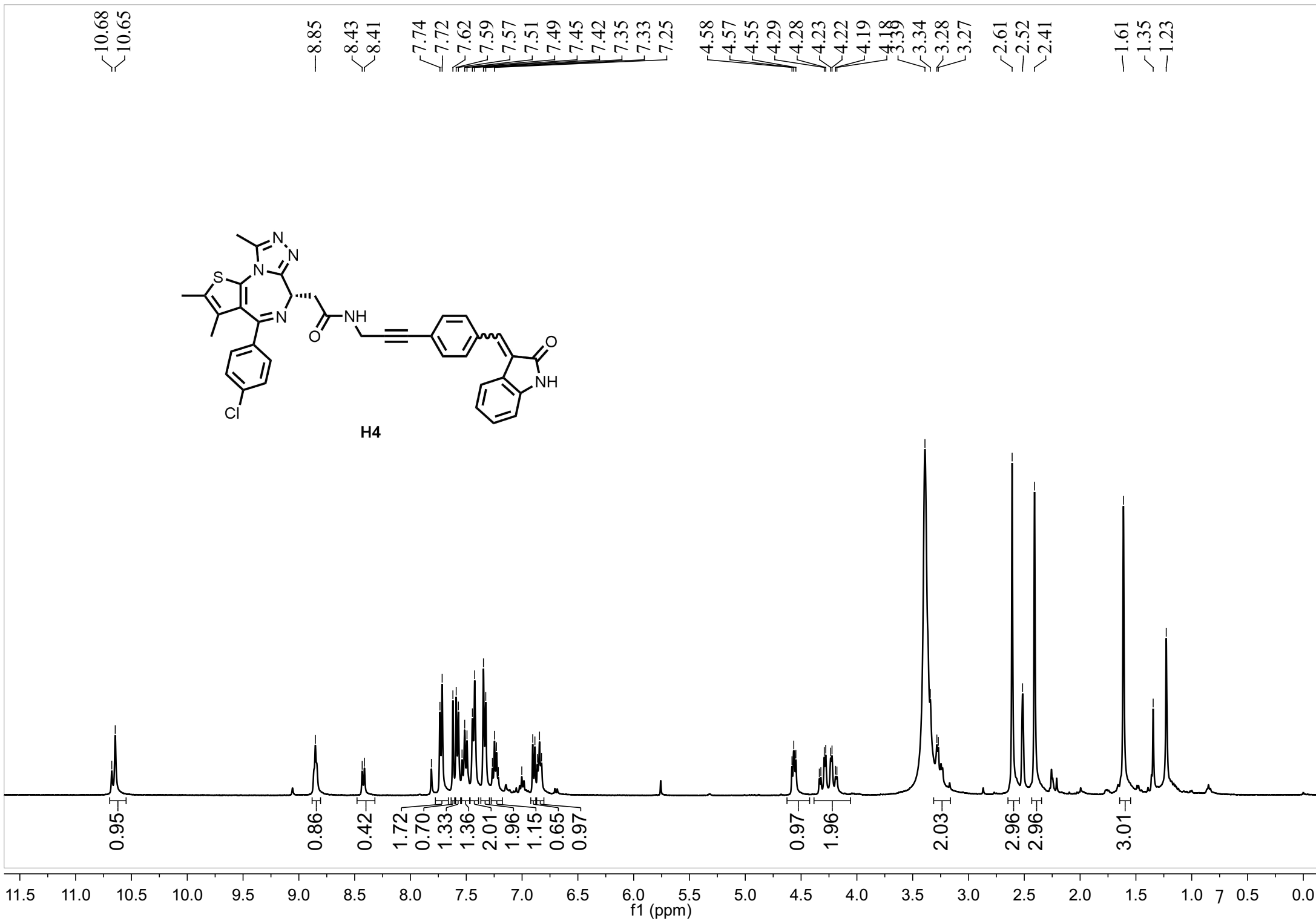

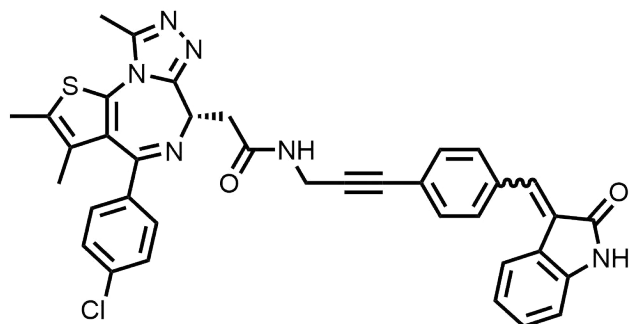

H4

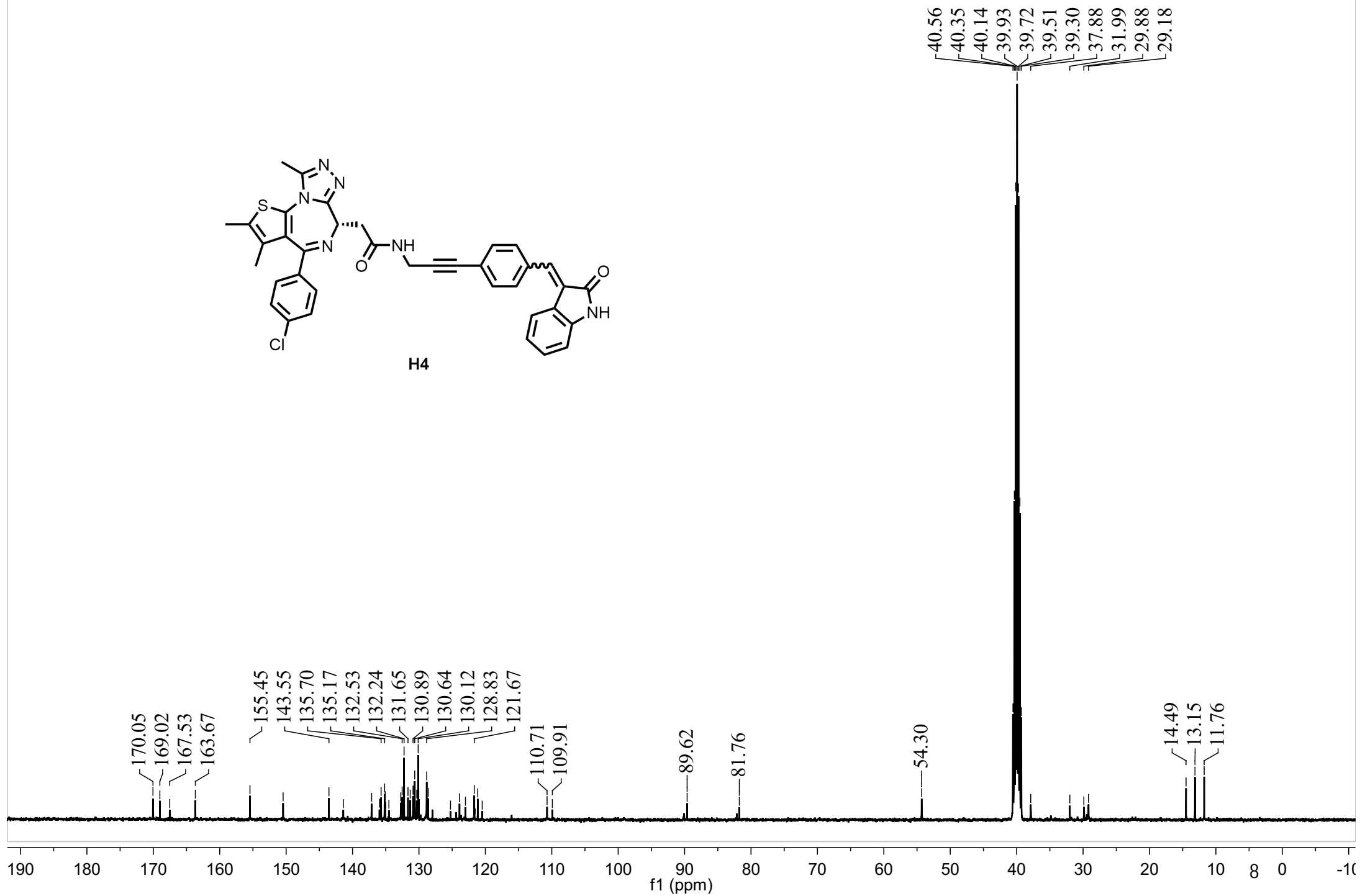

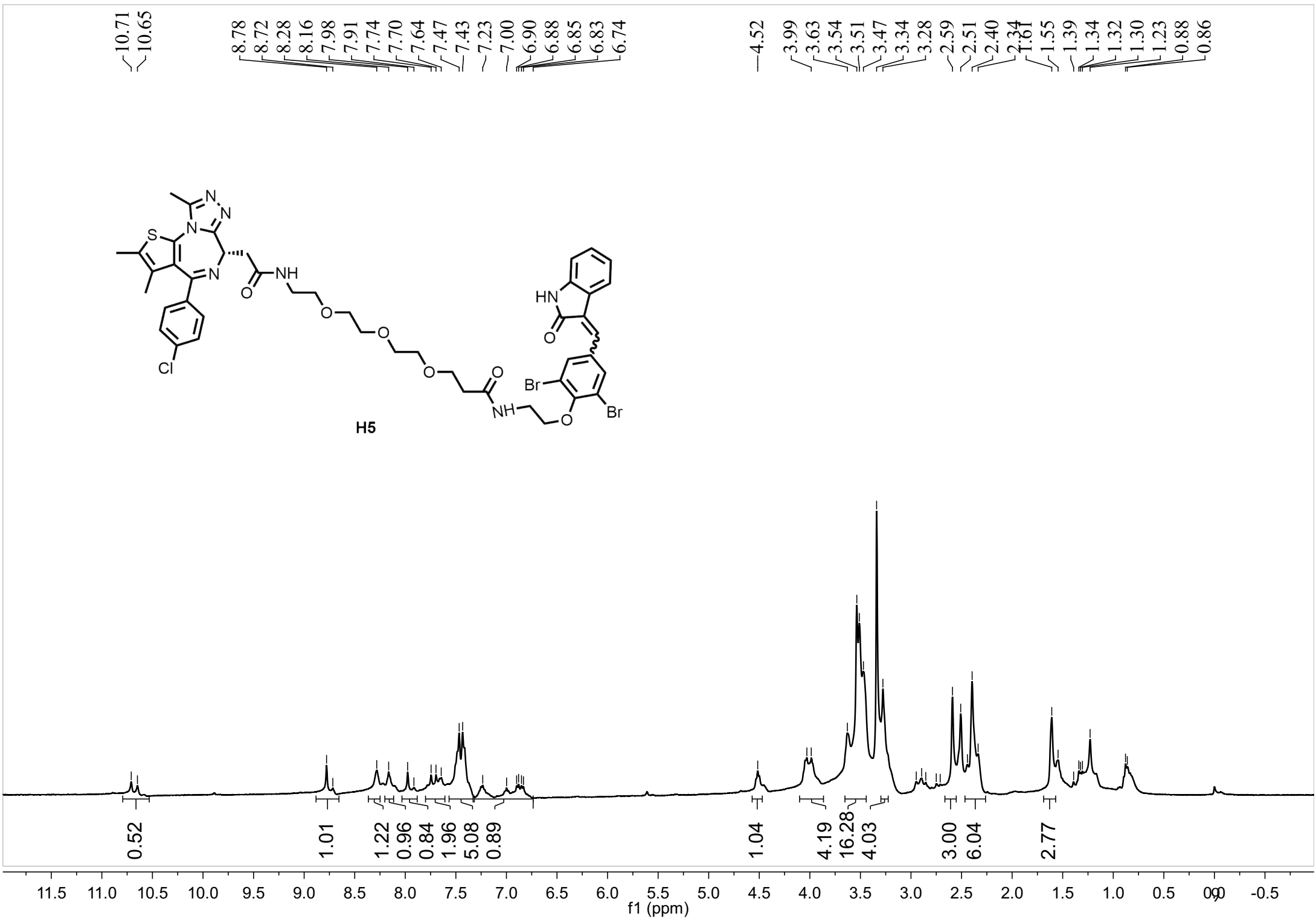

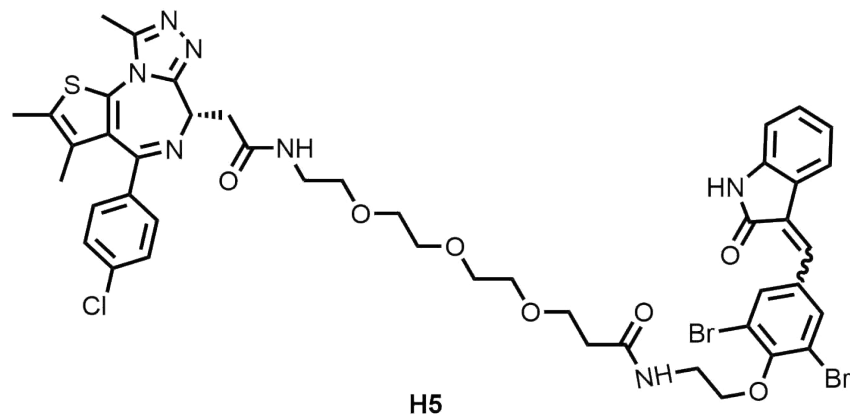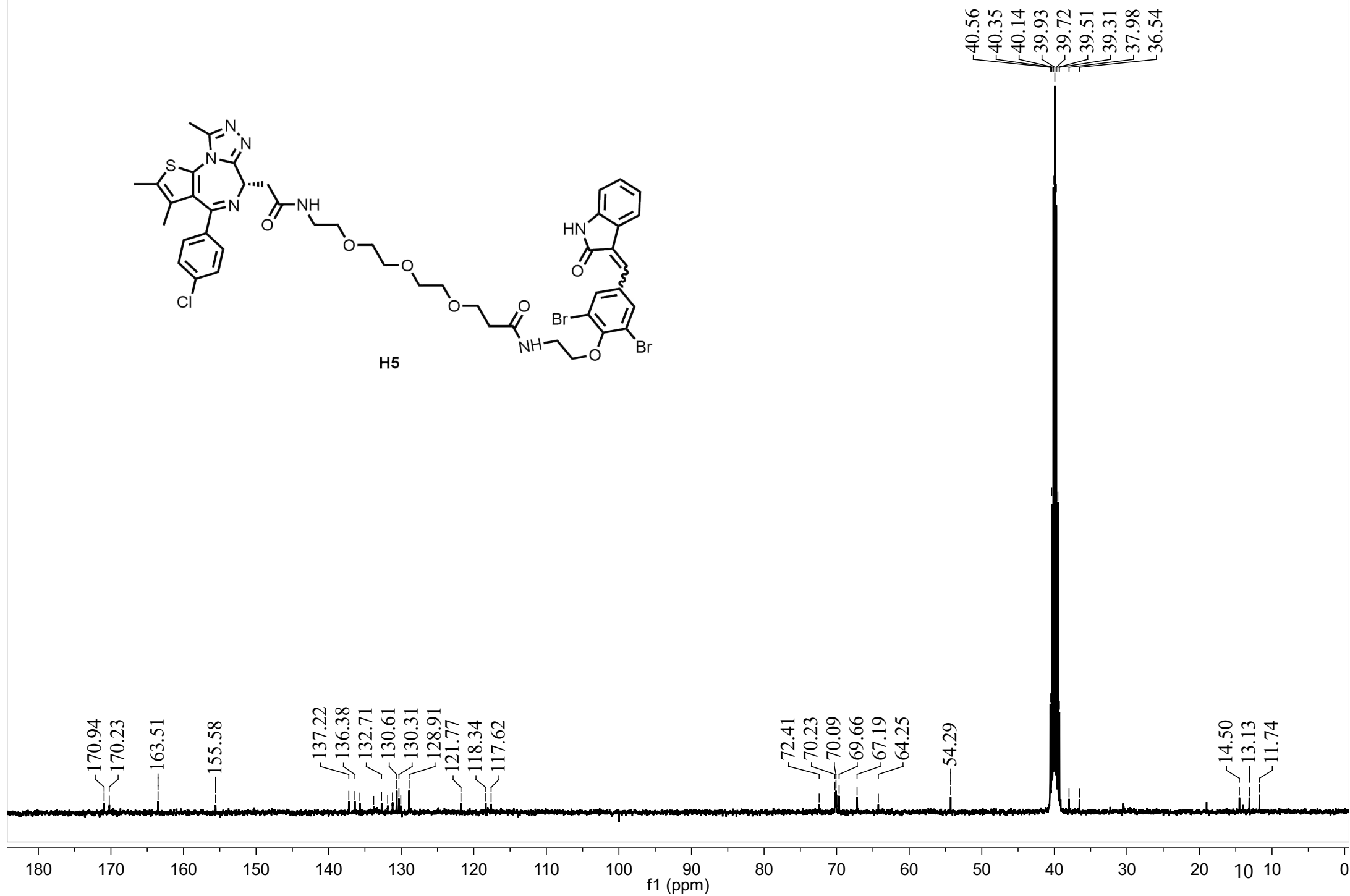

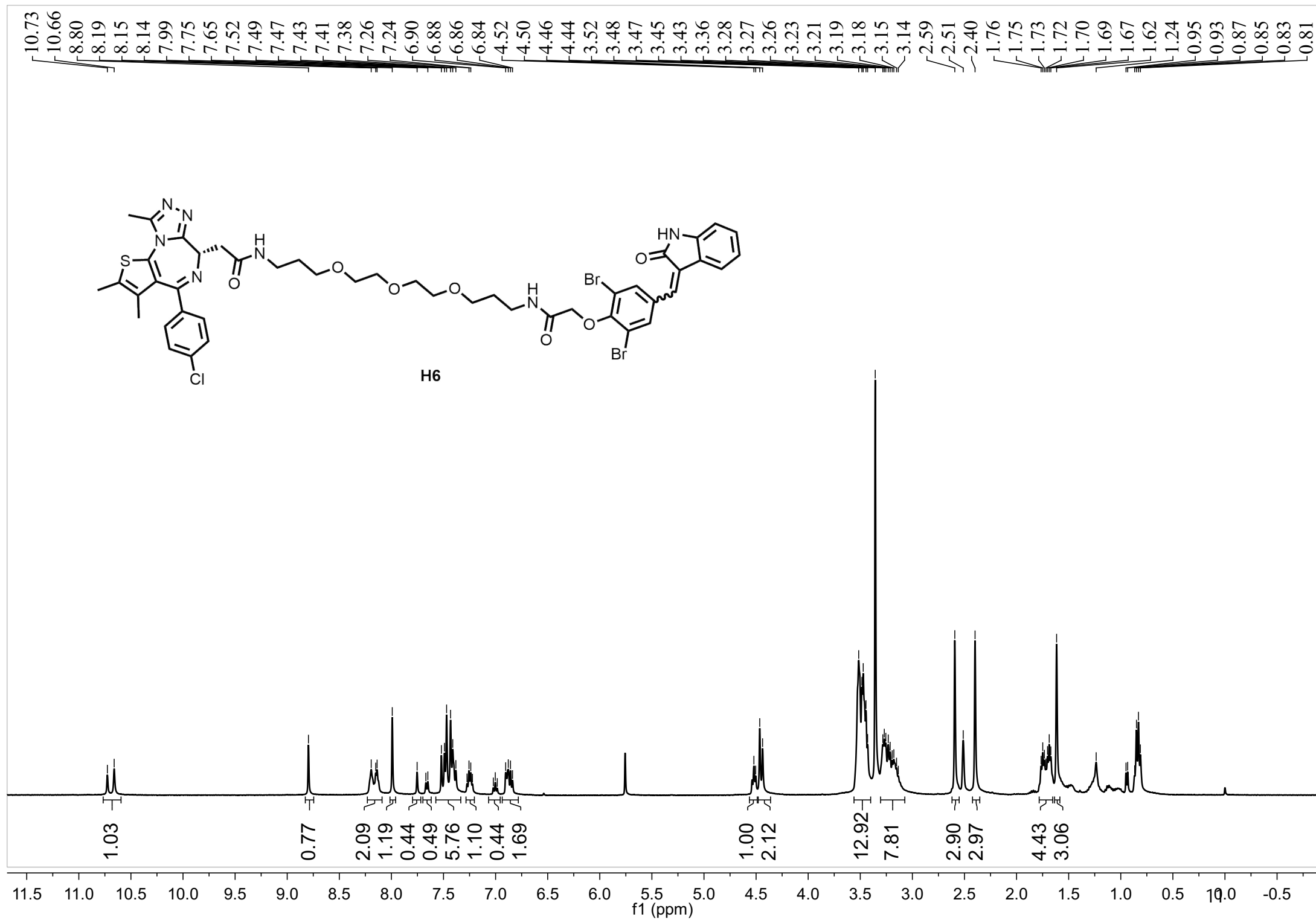

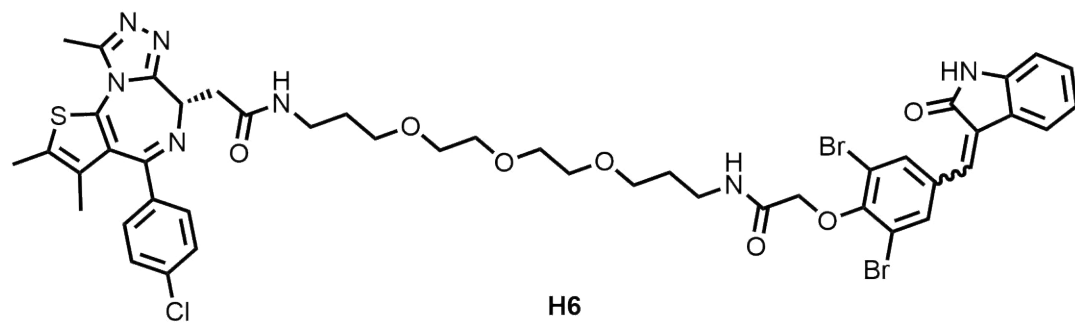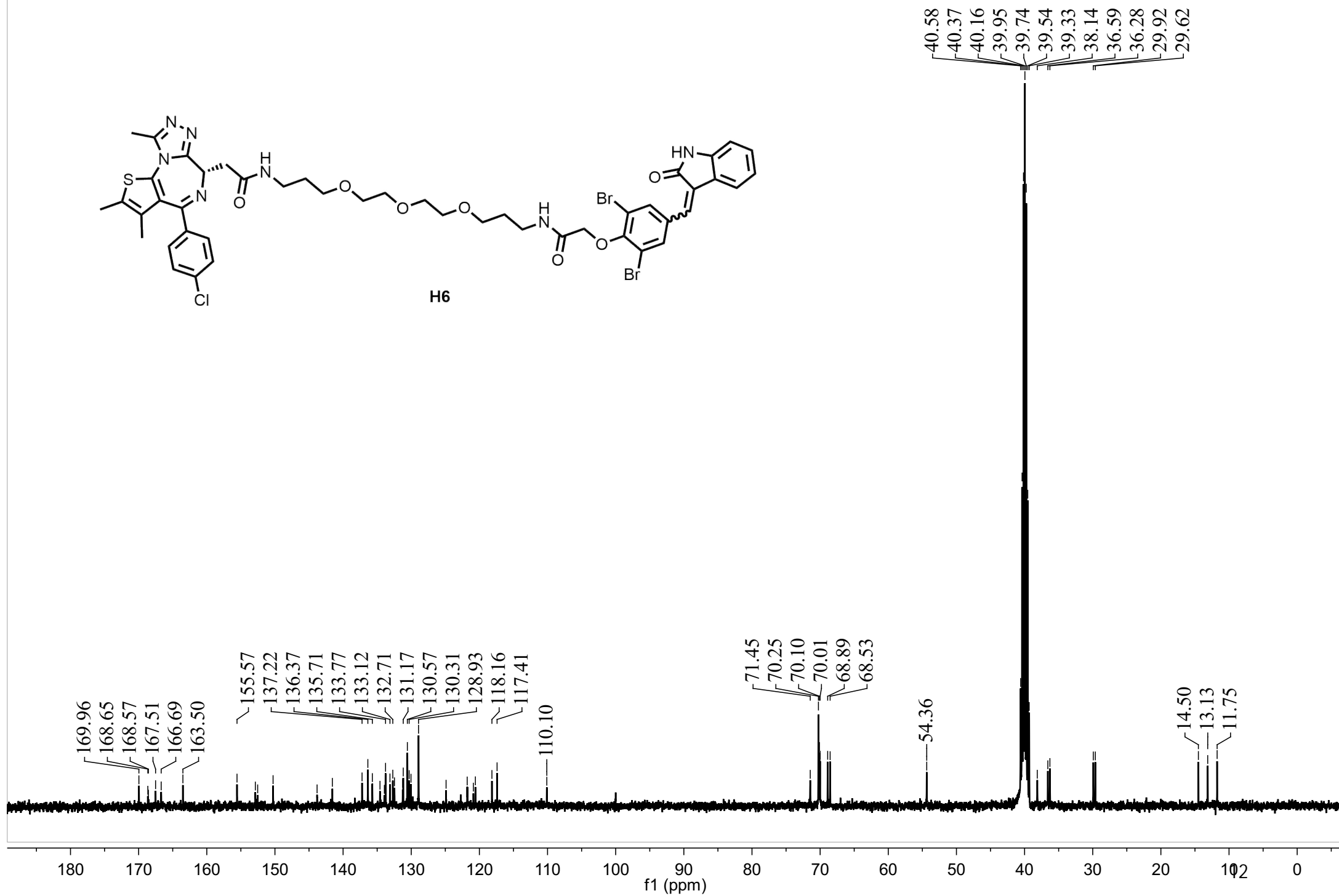

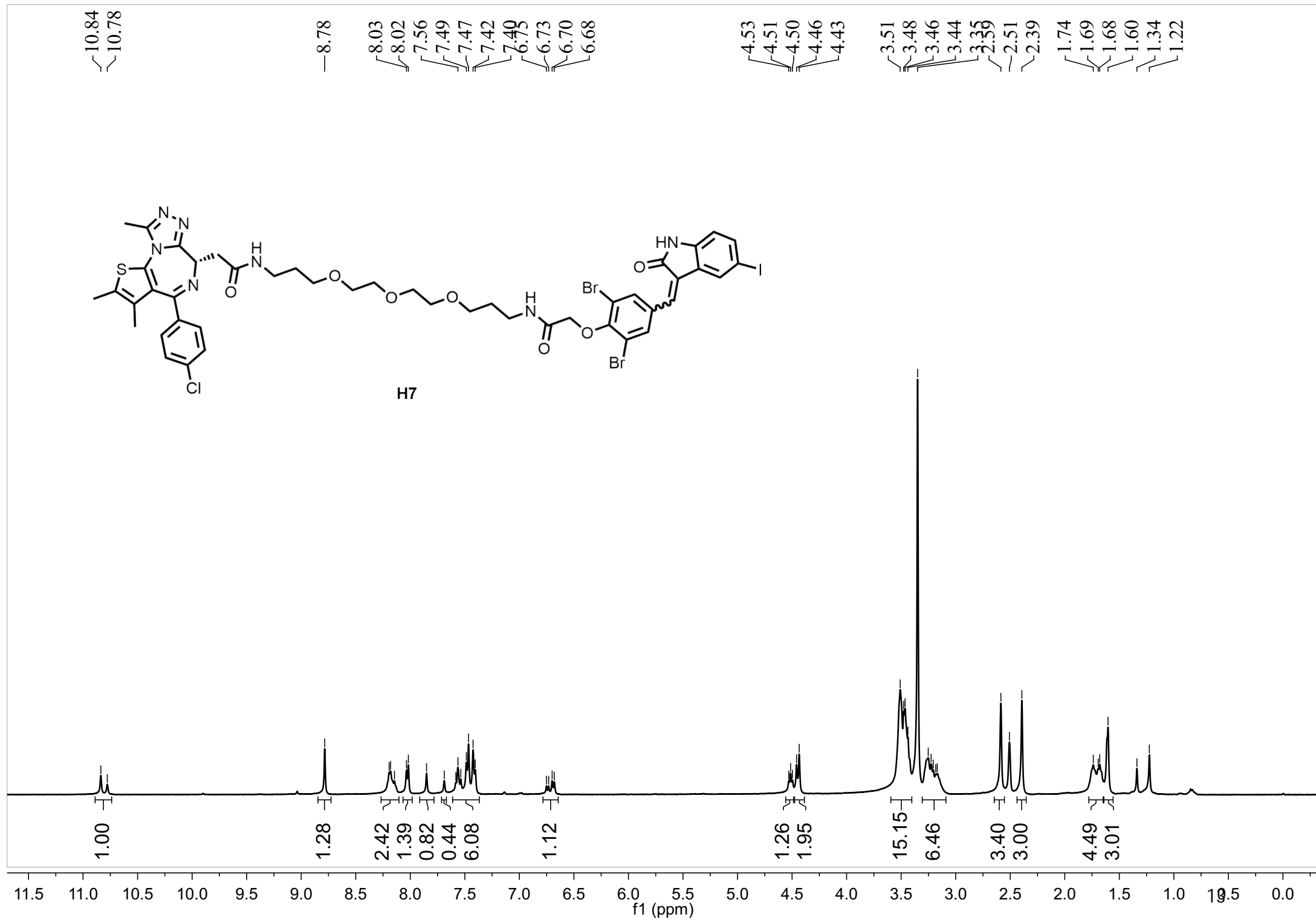

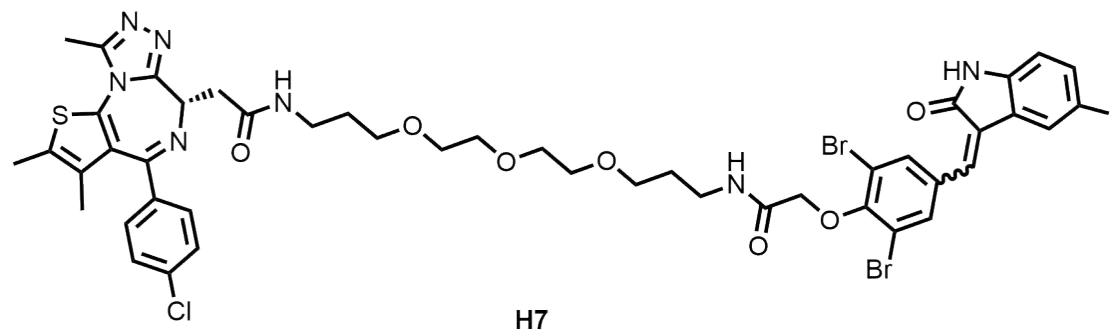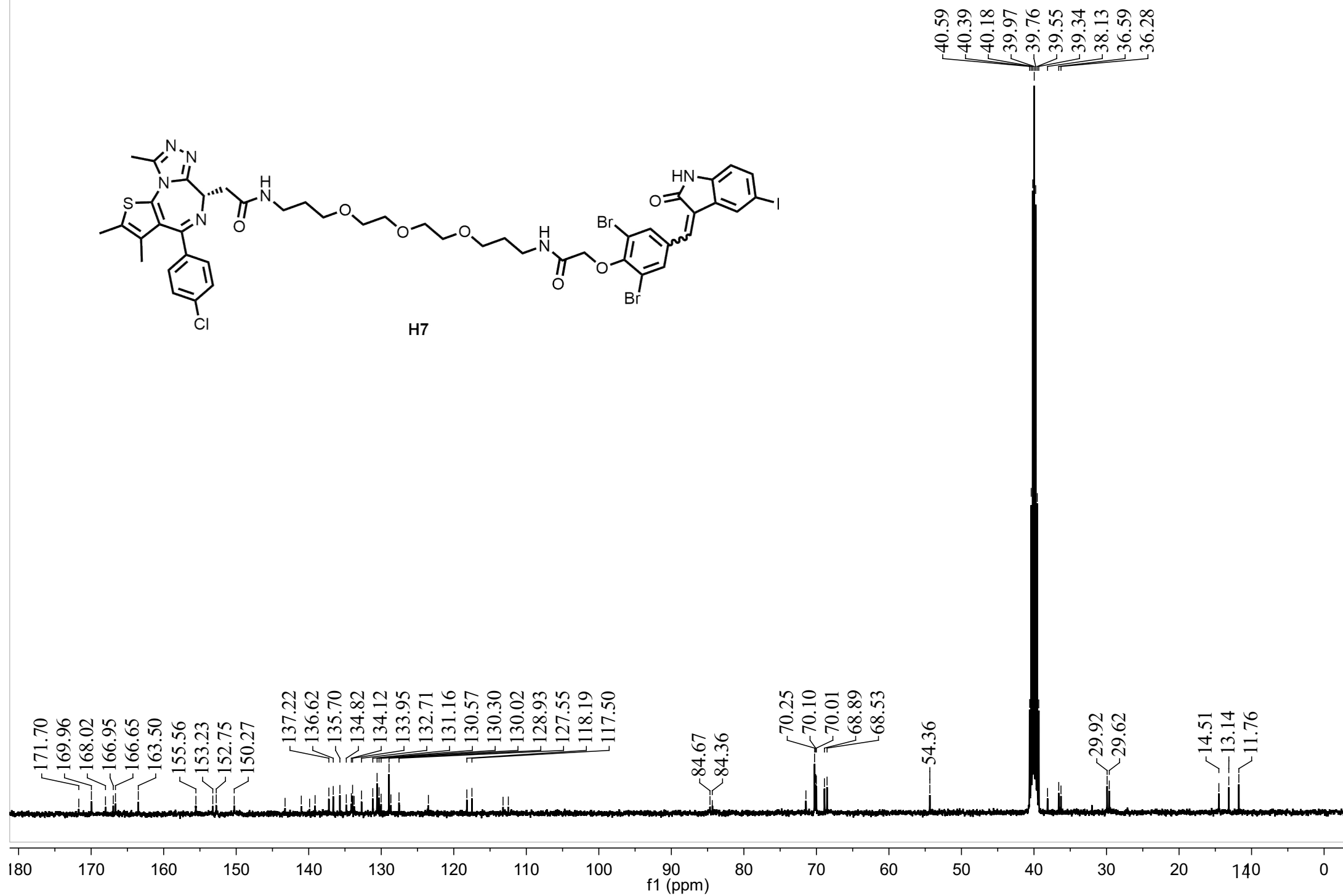

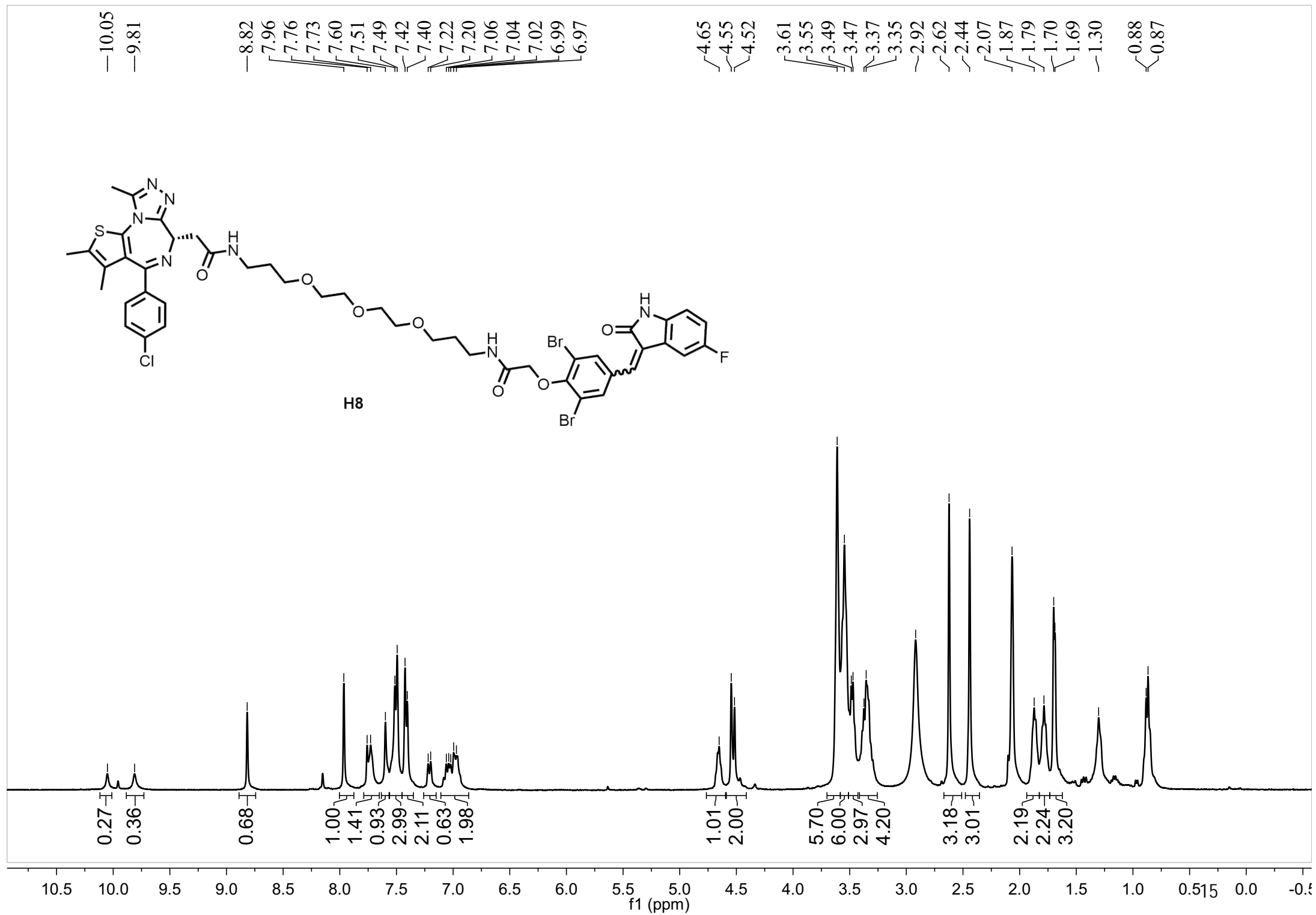

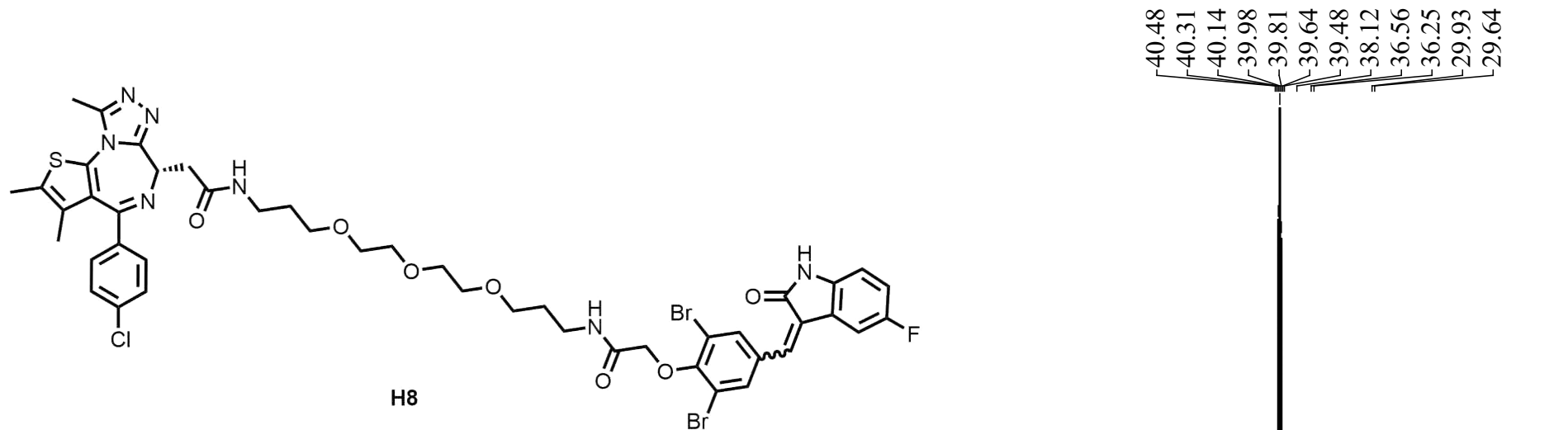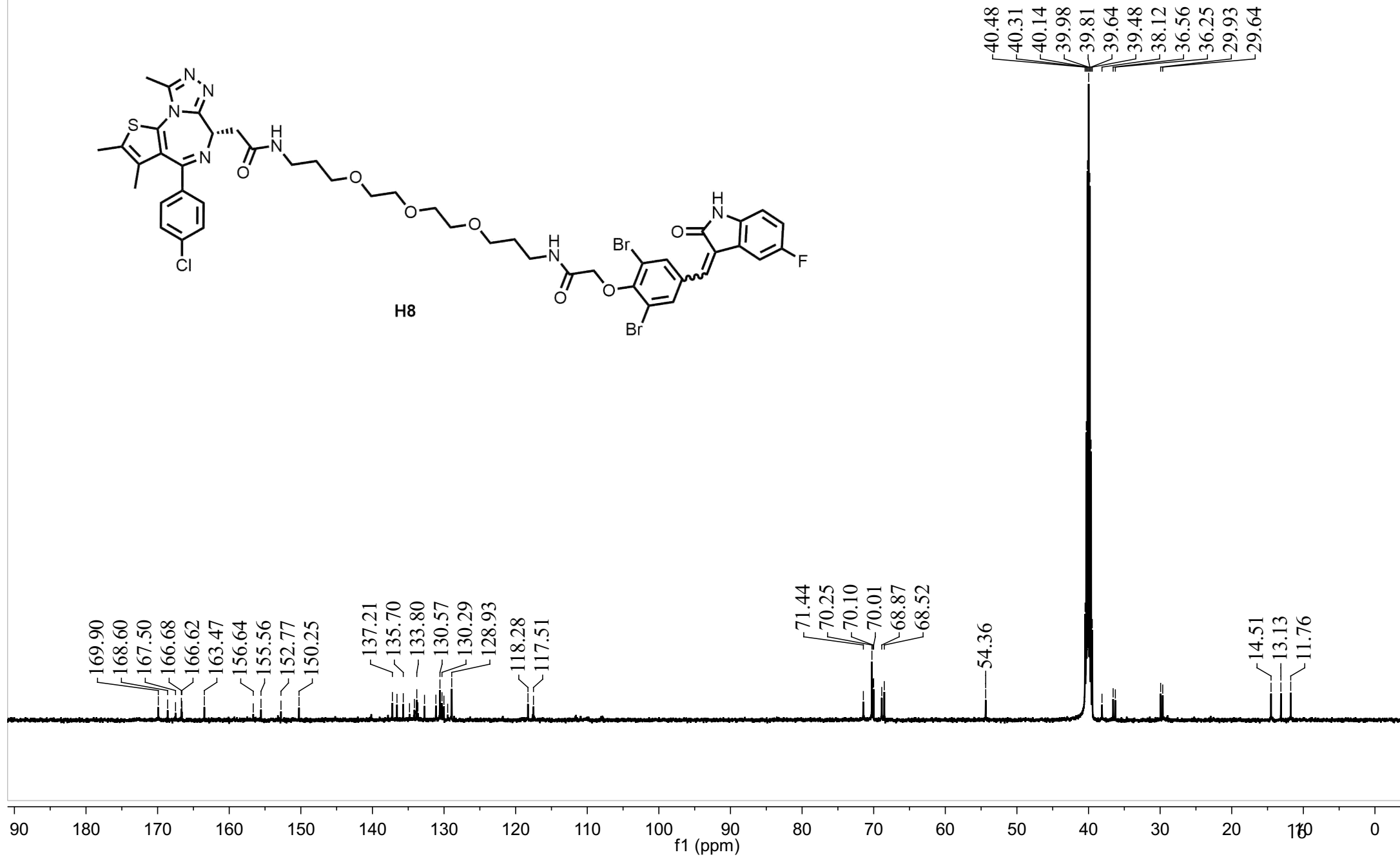

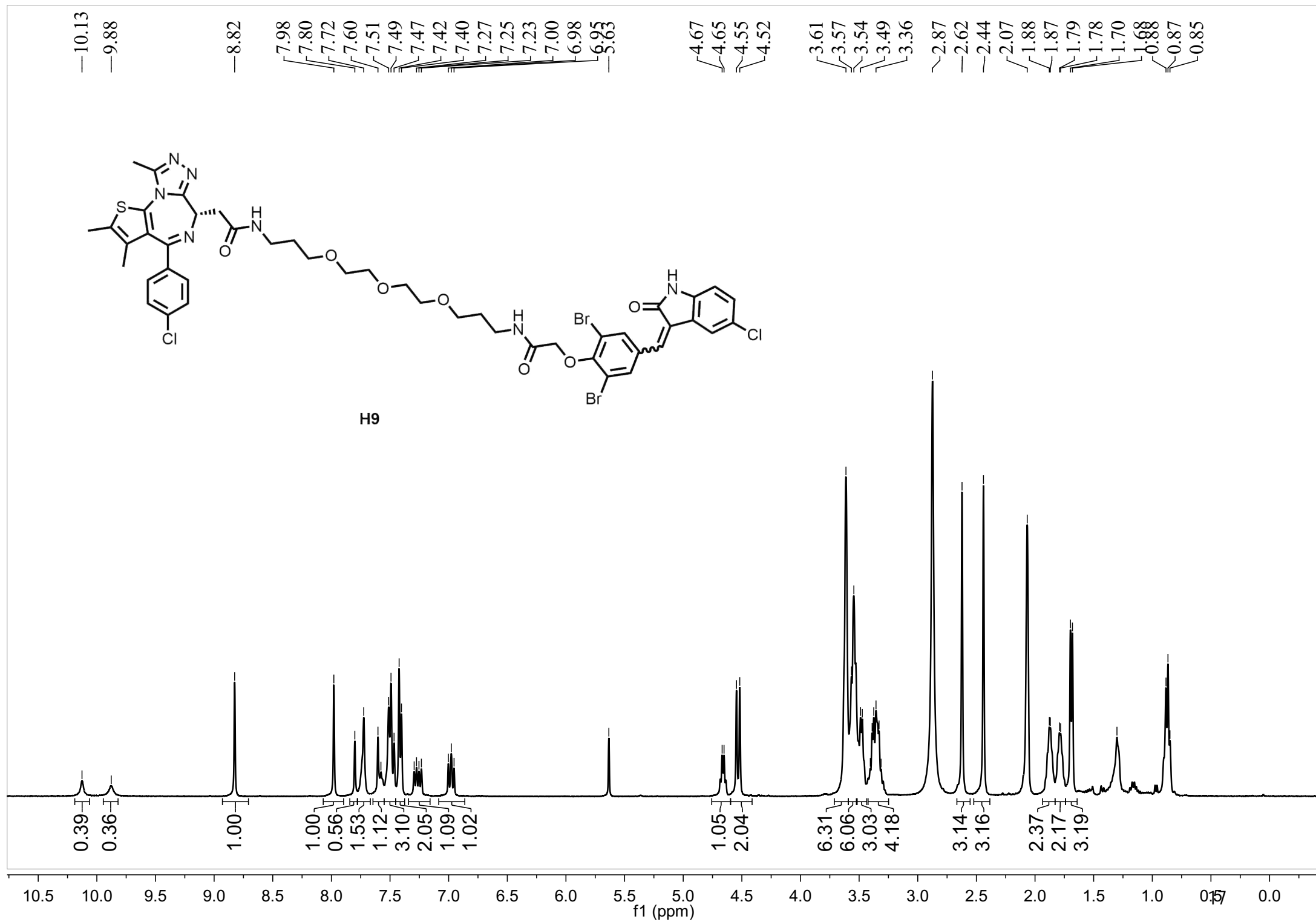

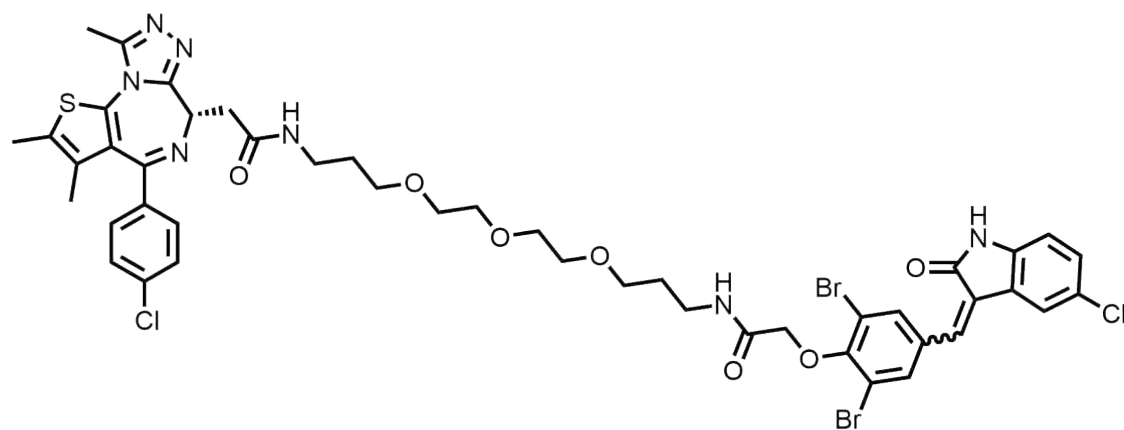

H9

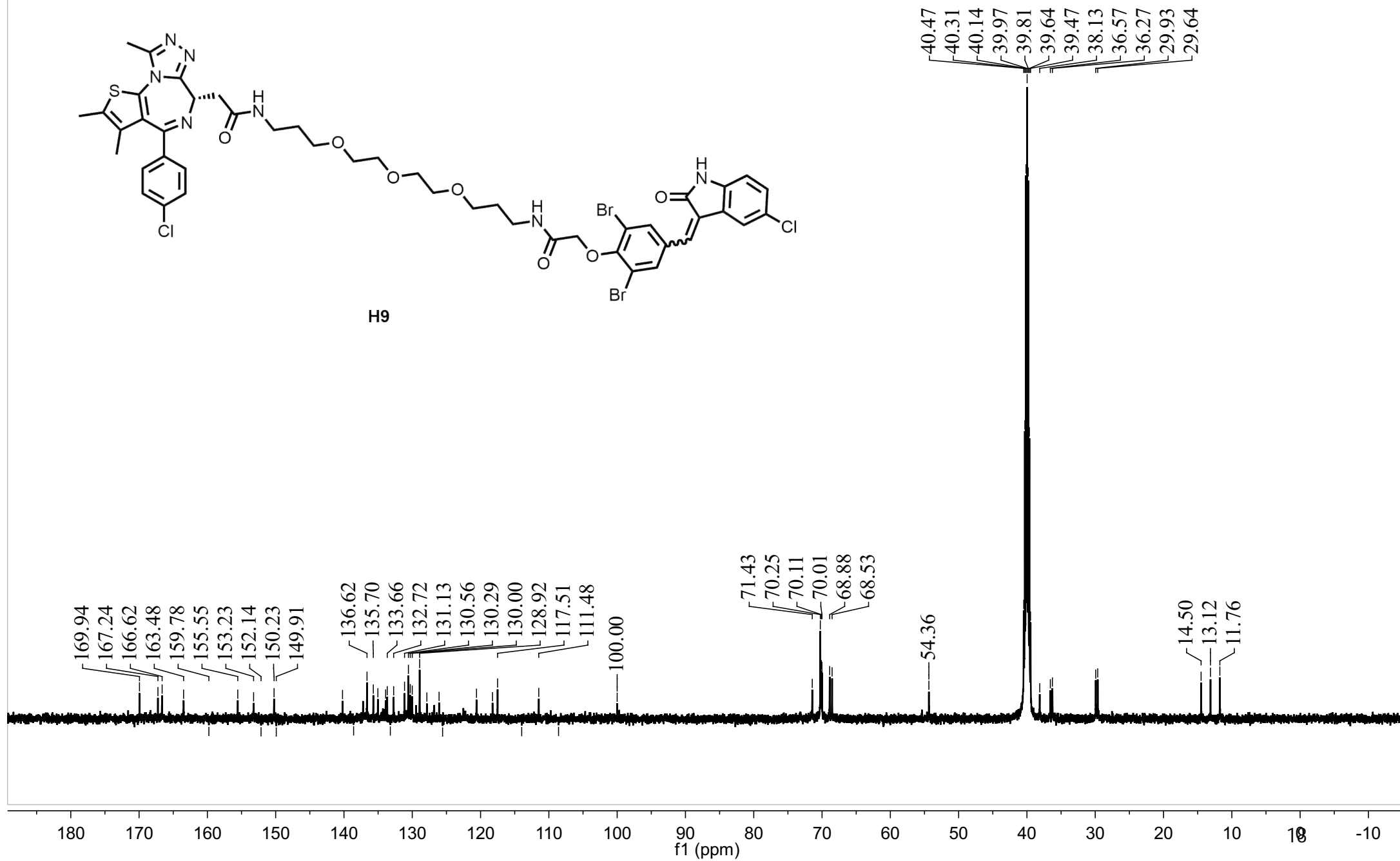

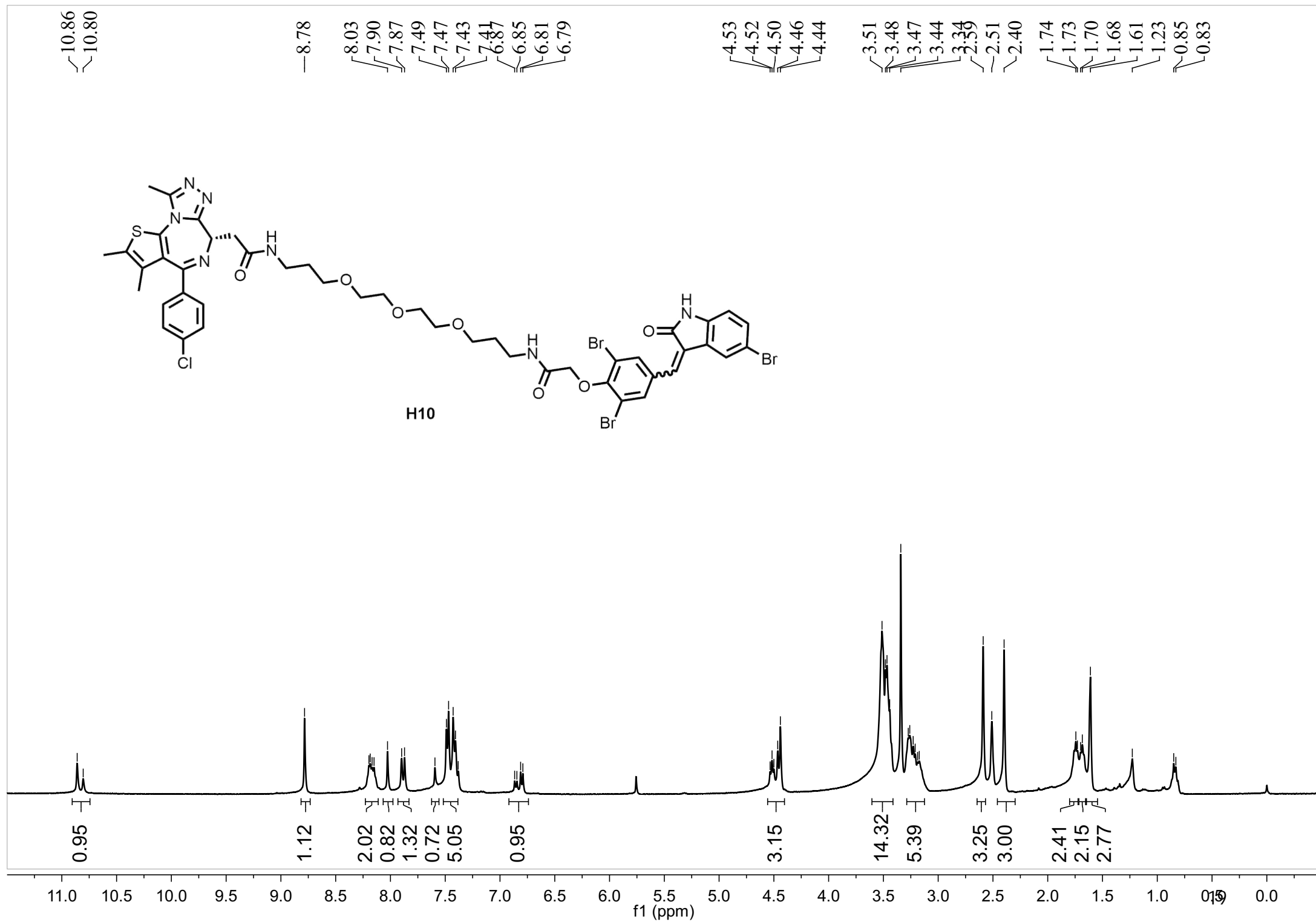

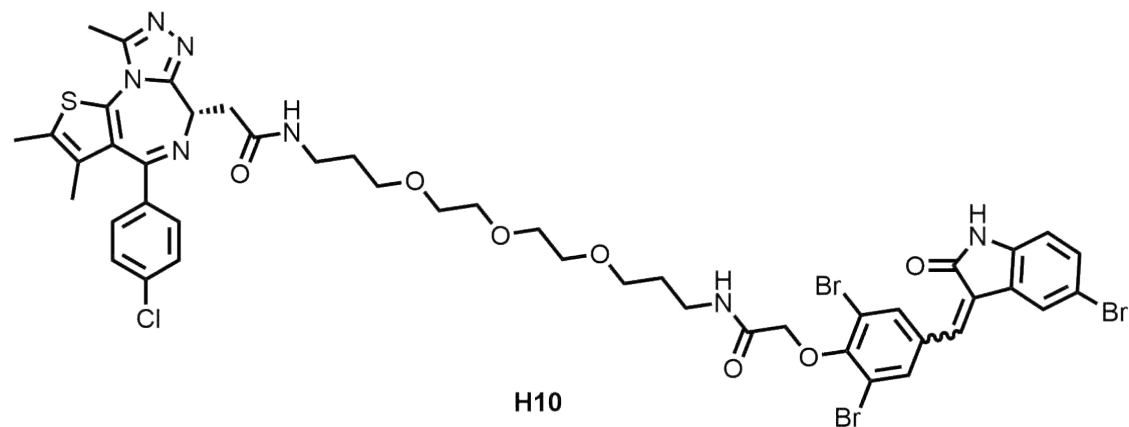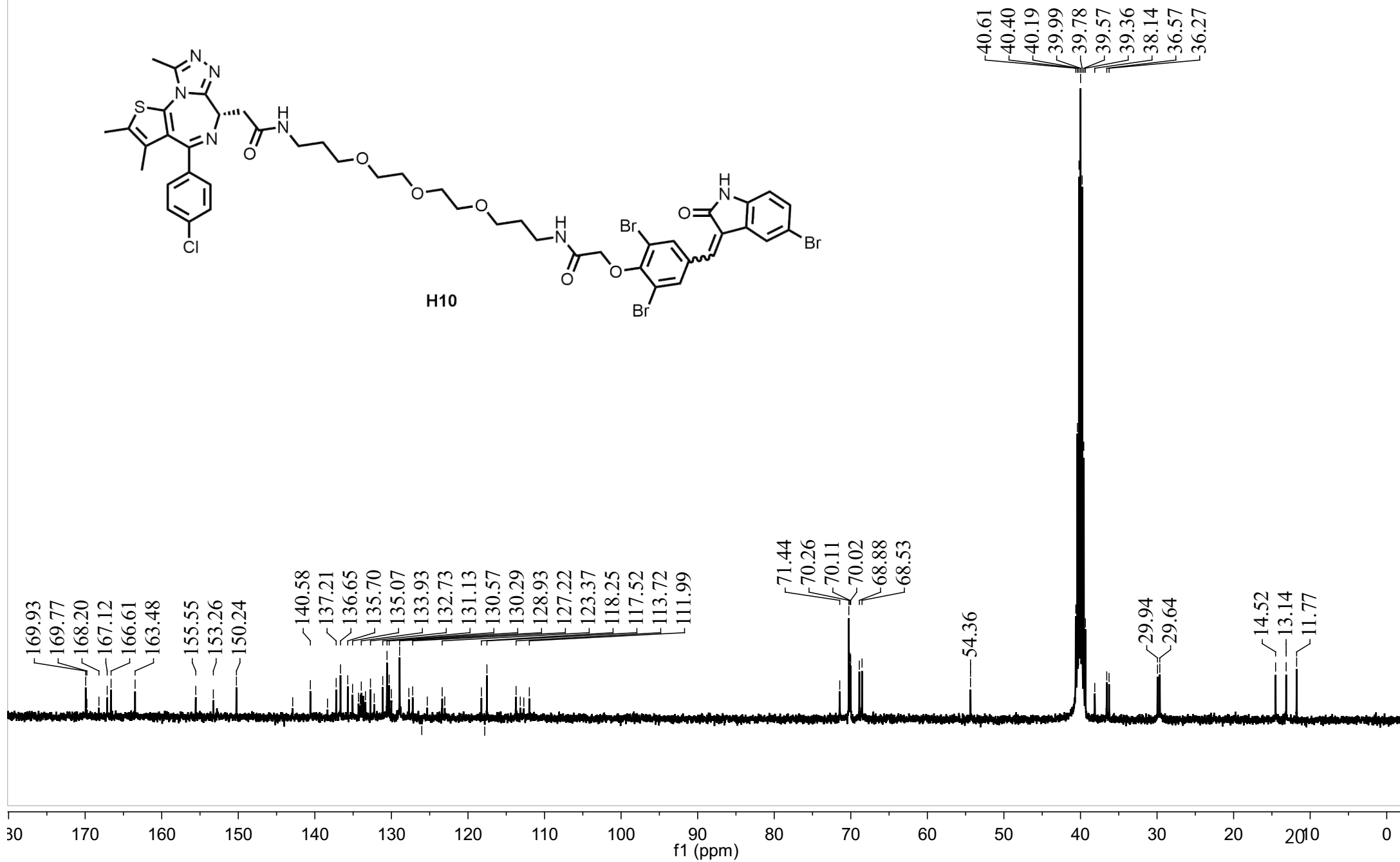

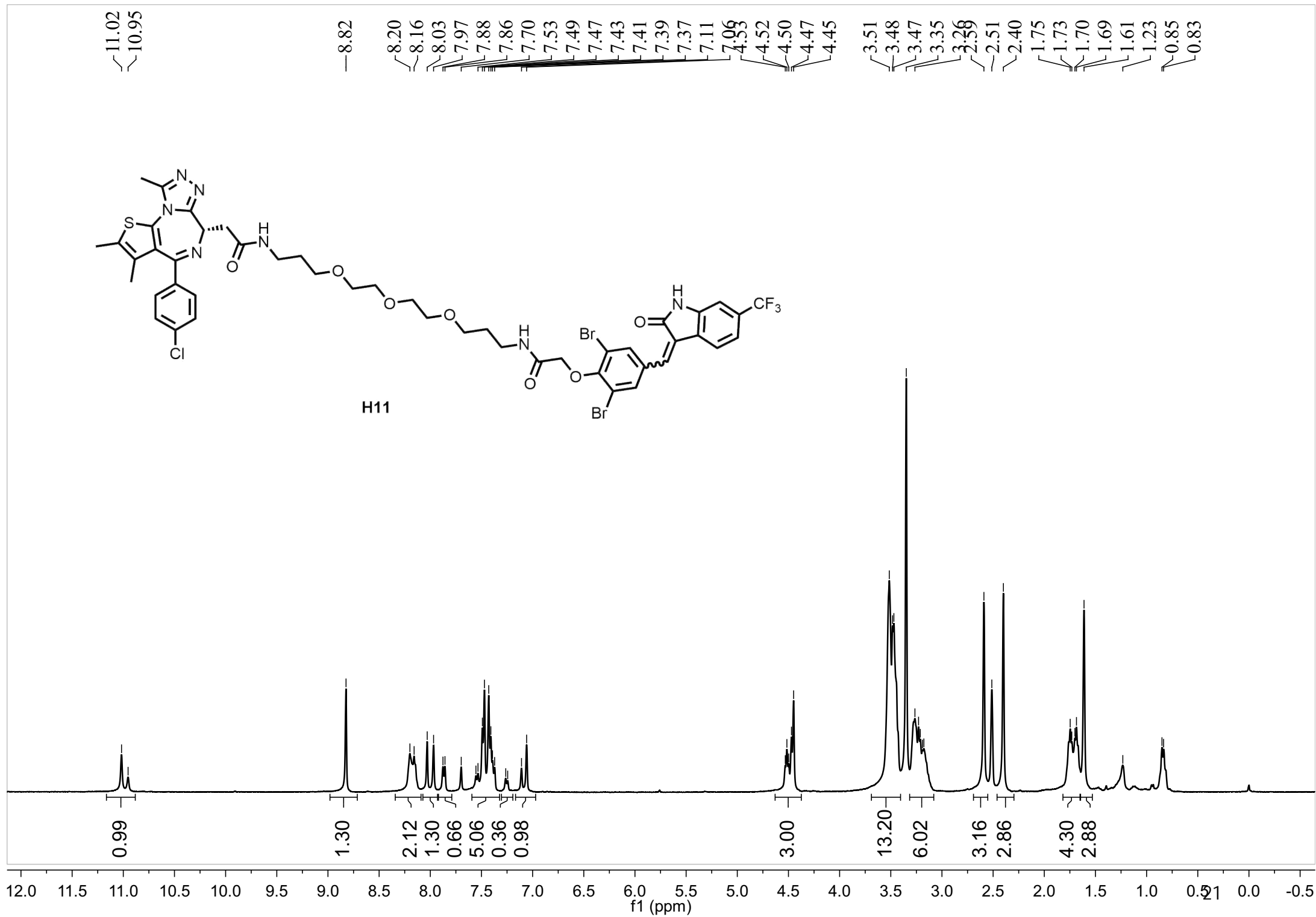

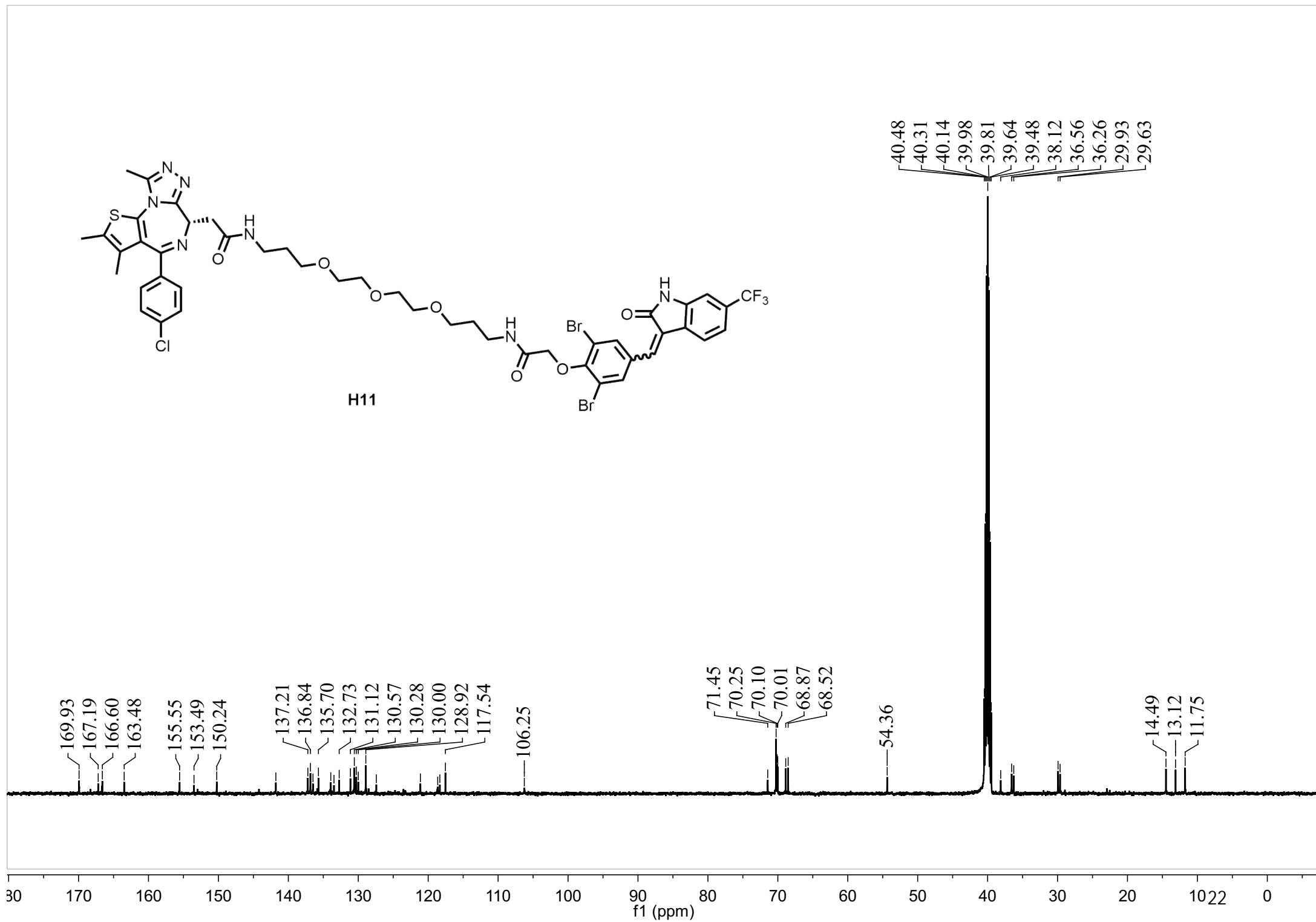

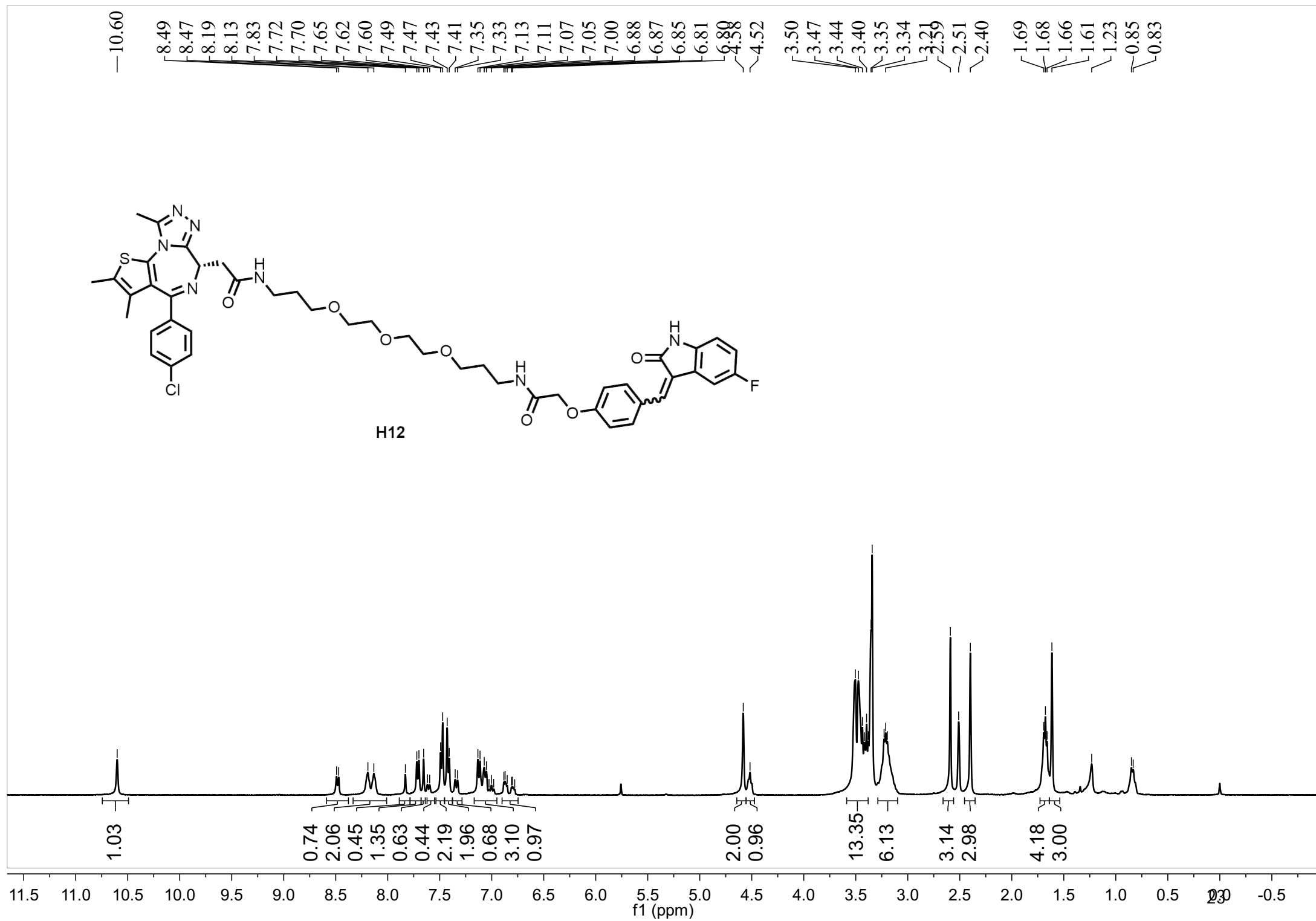

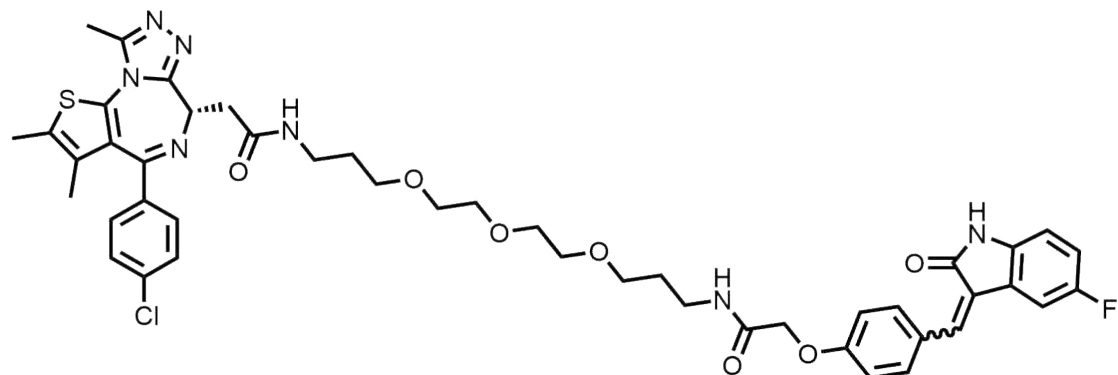

H12

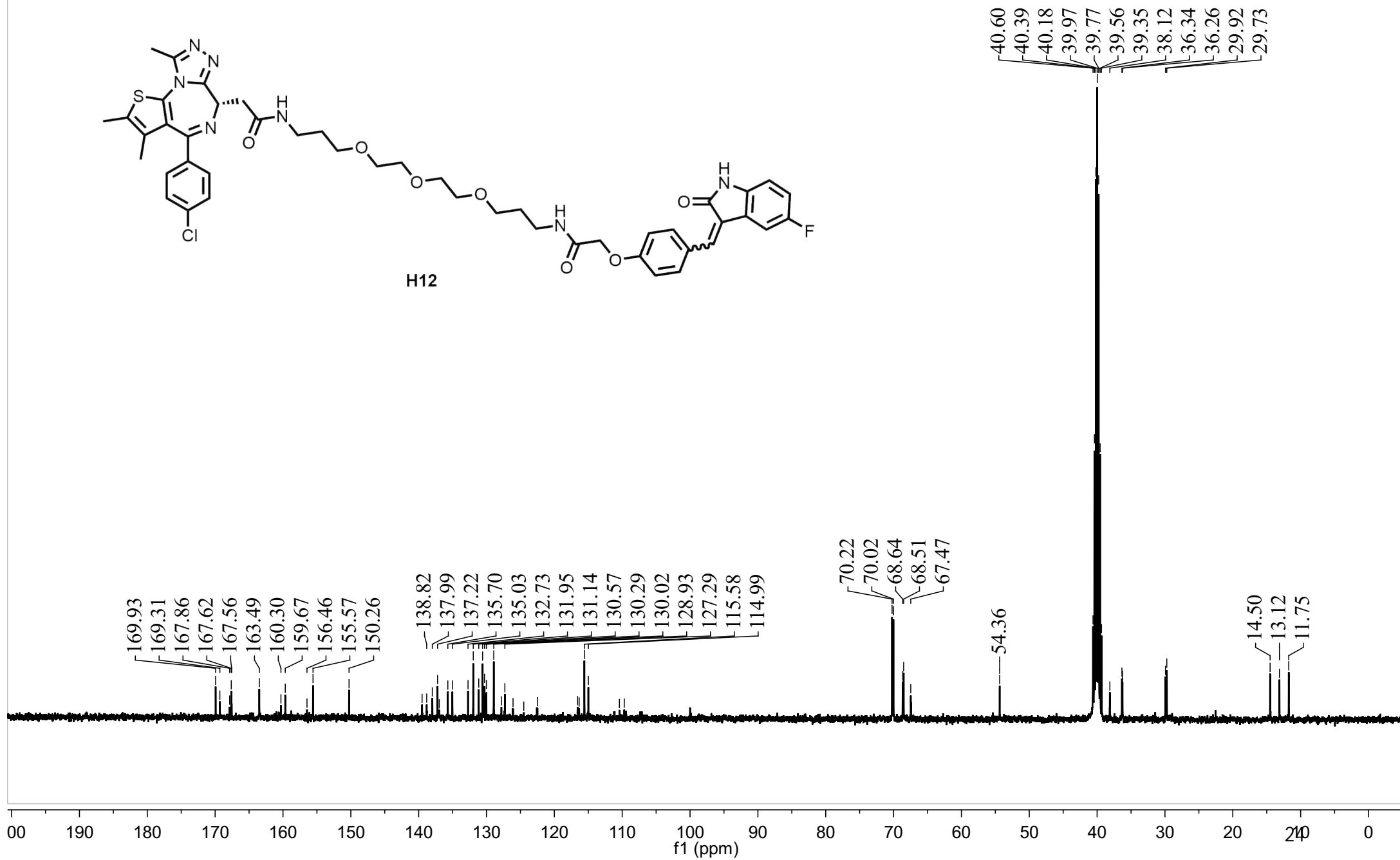

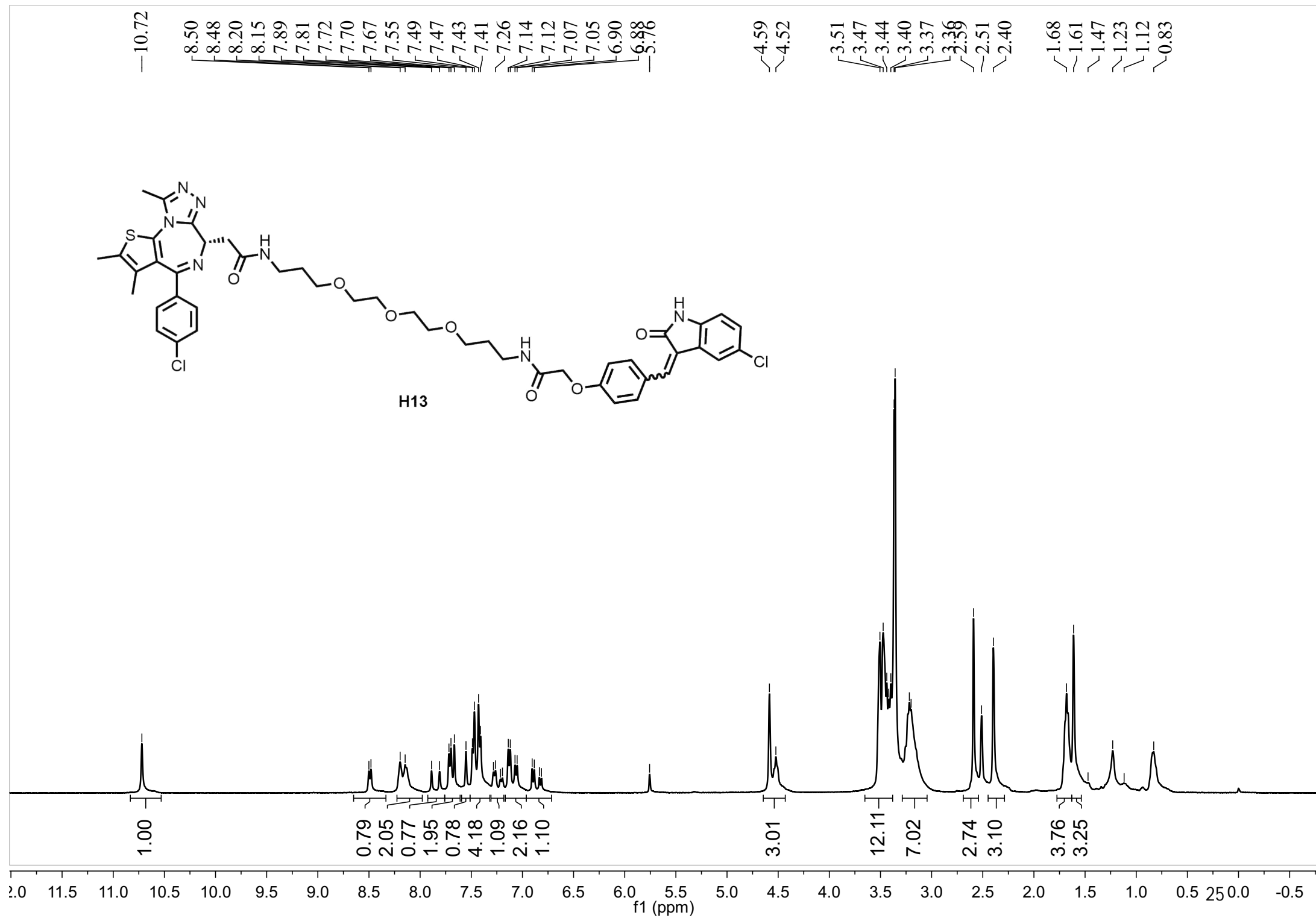

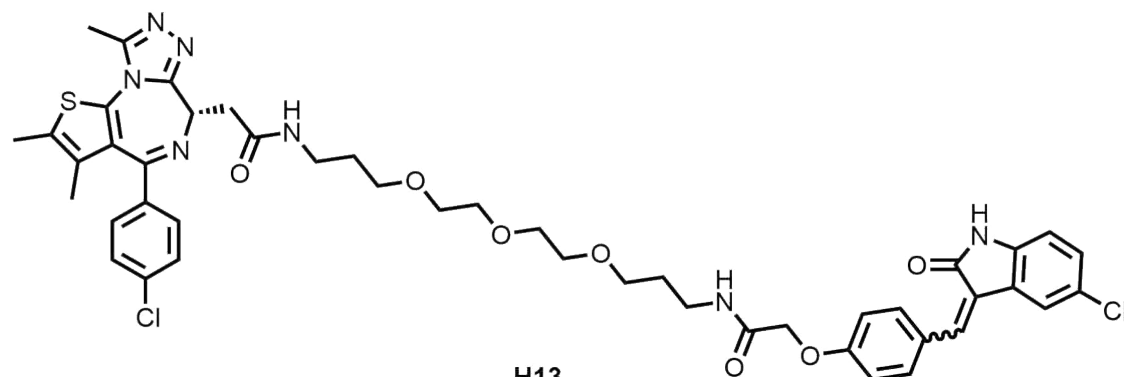

H13

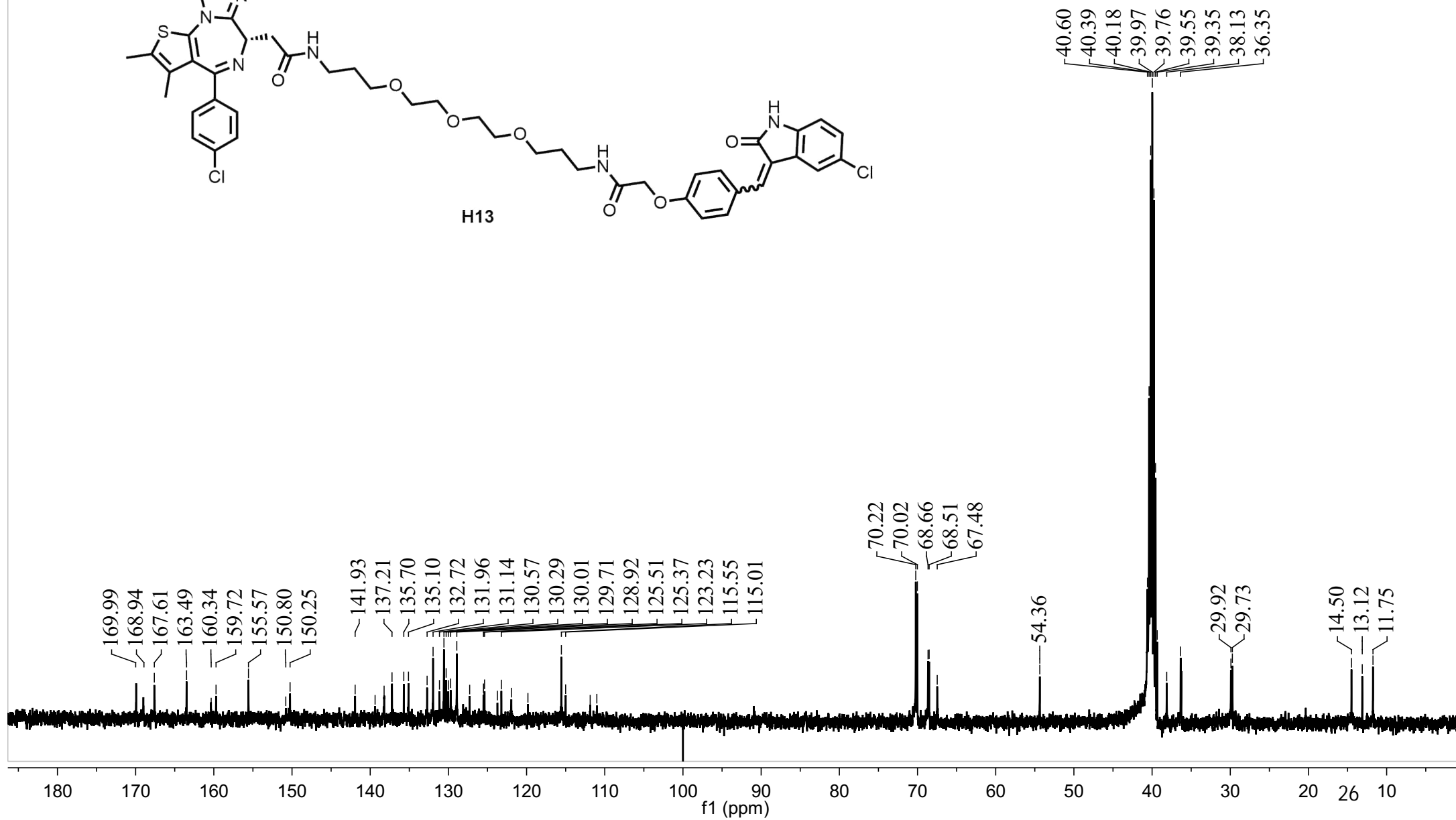

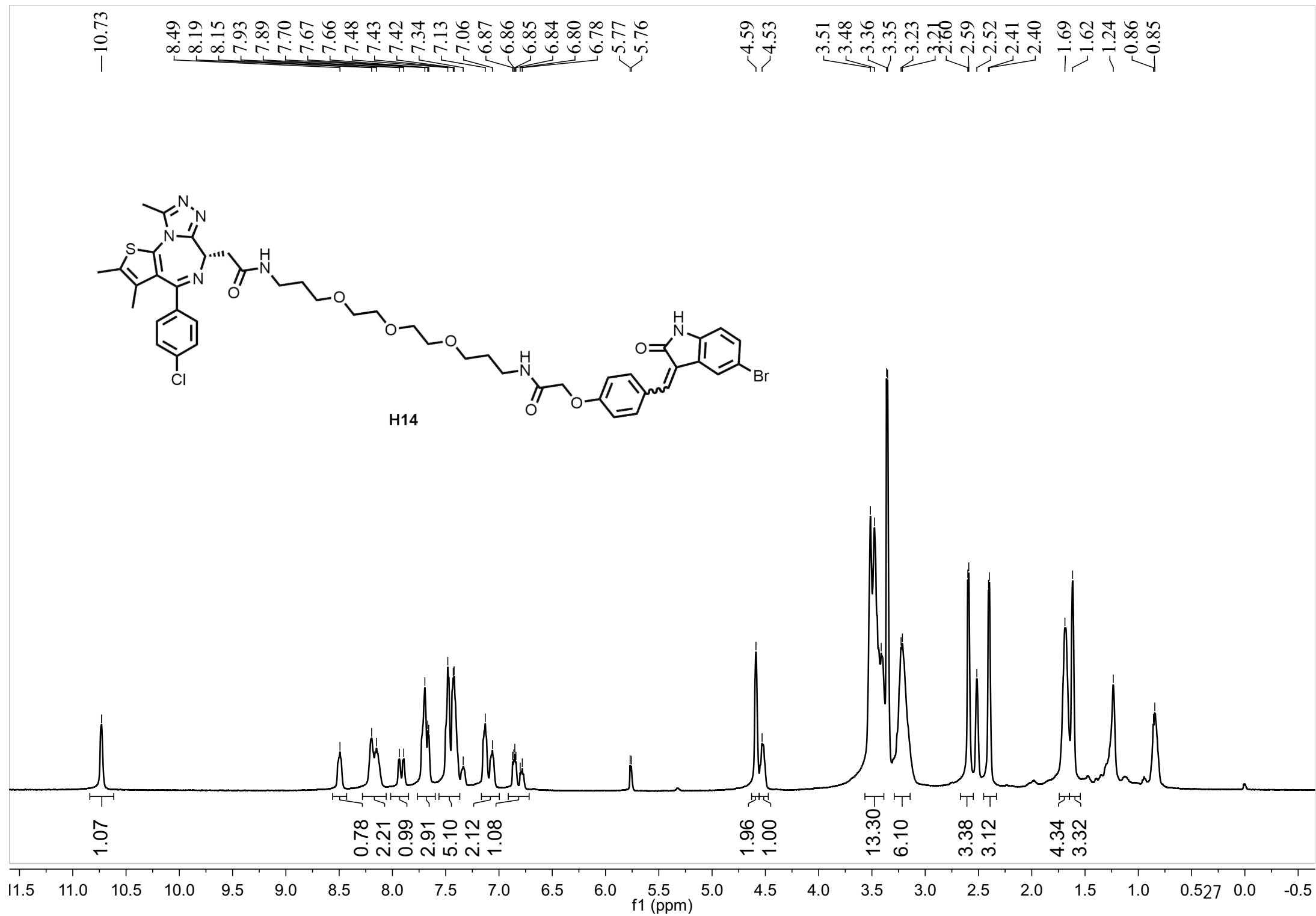

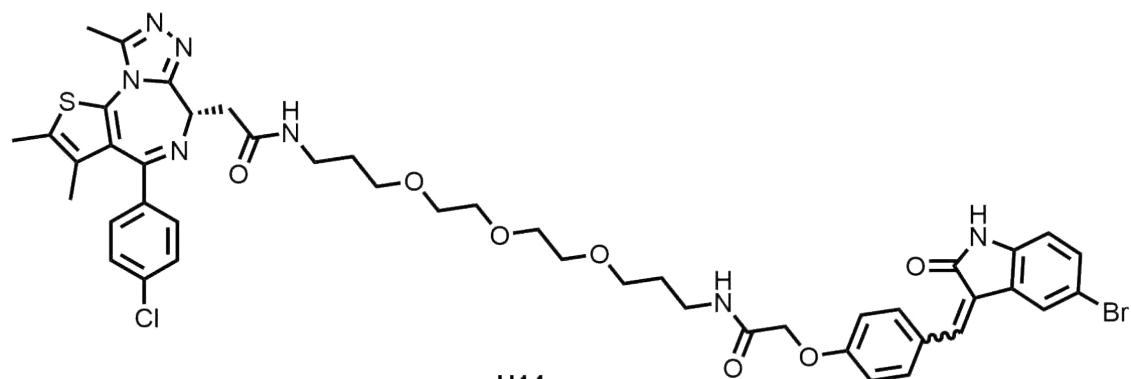

H14

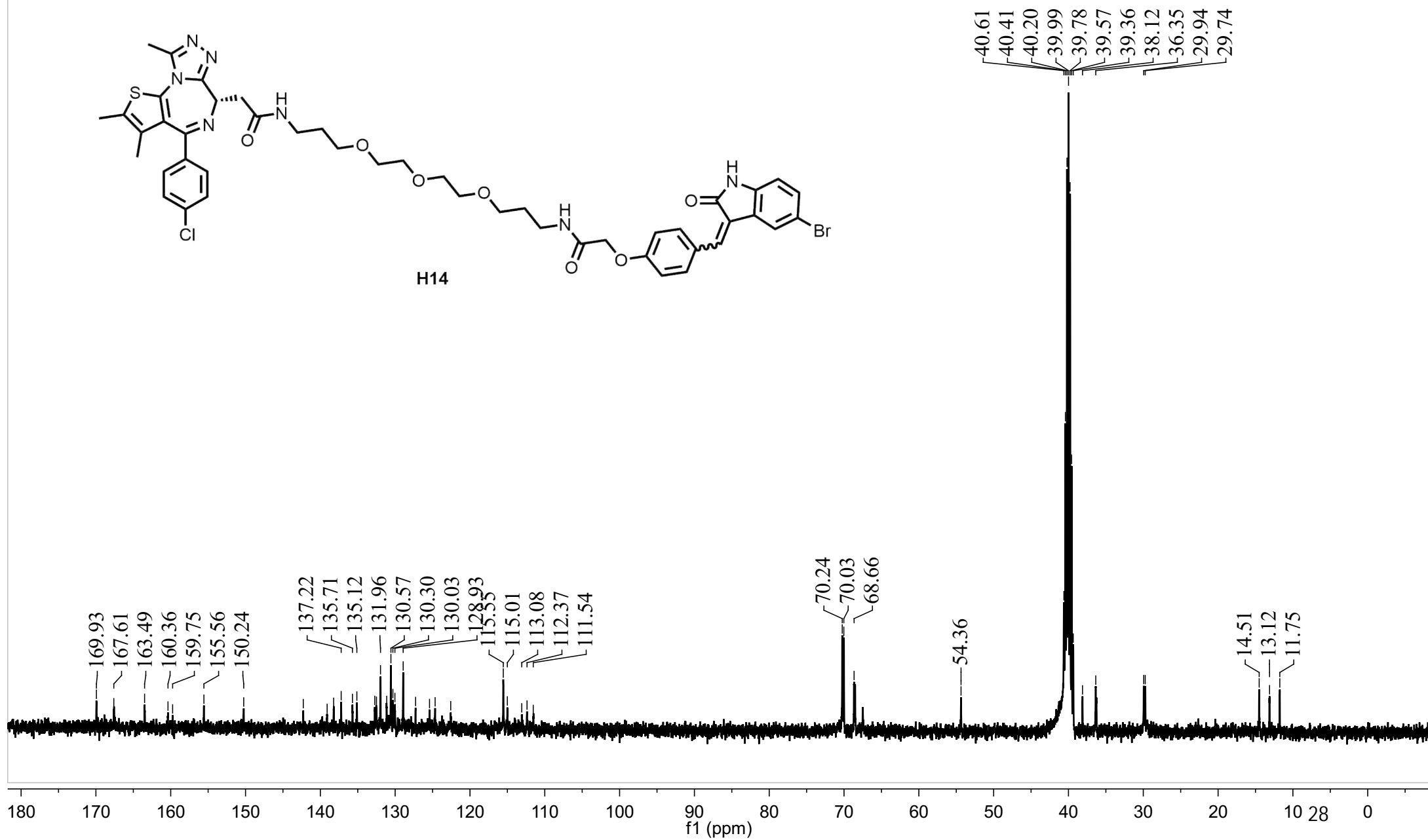

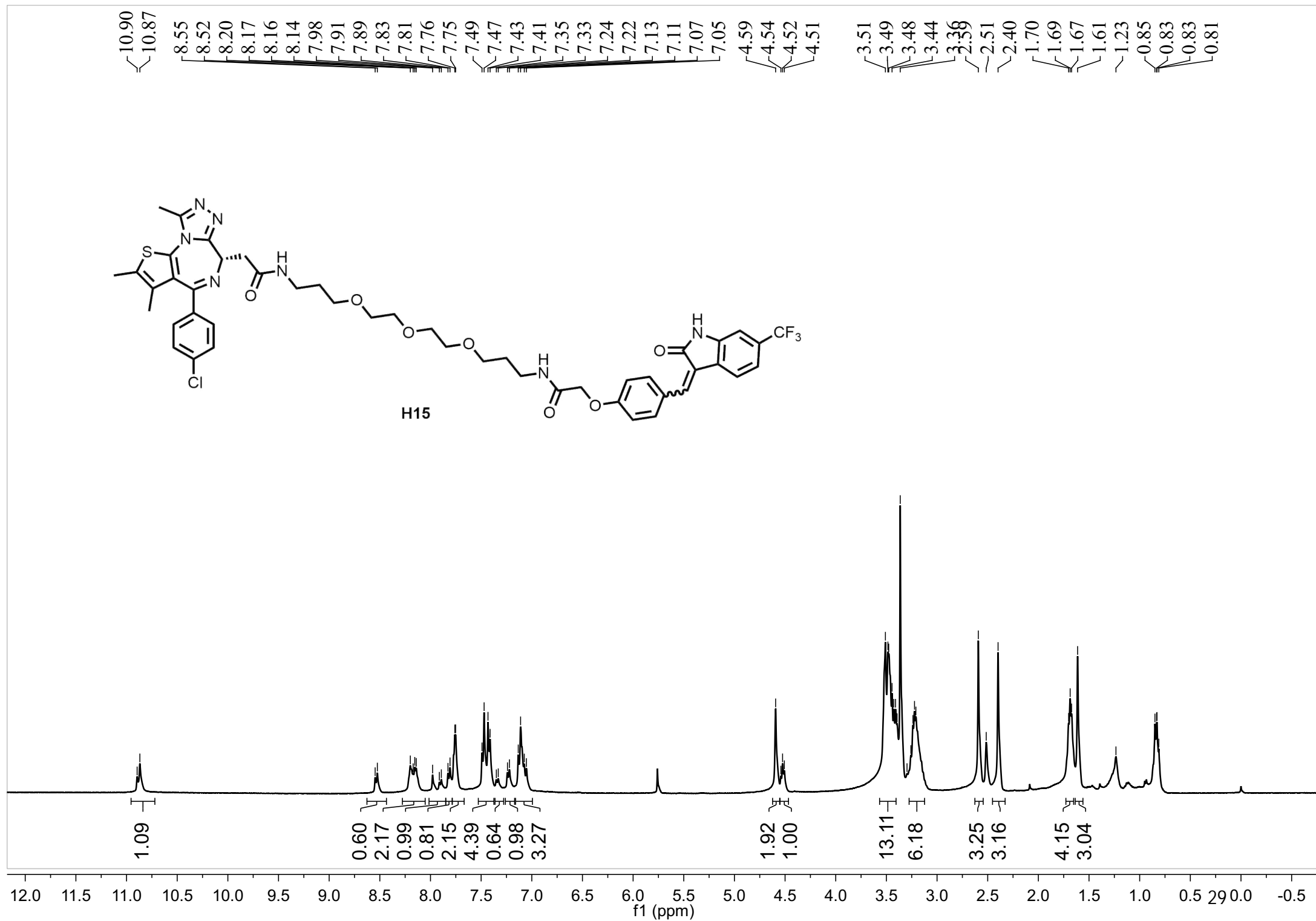

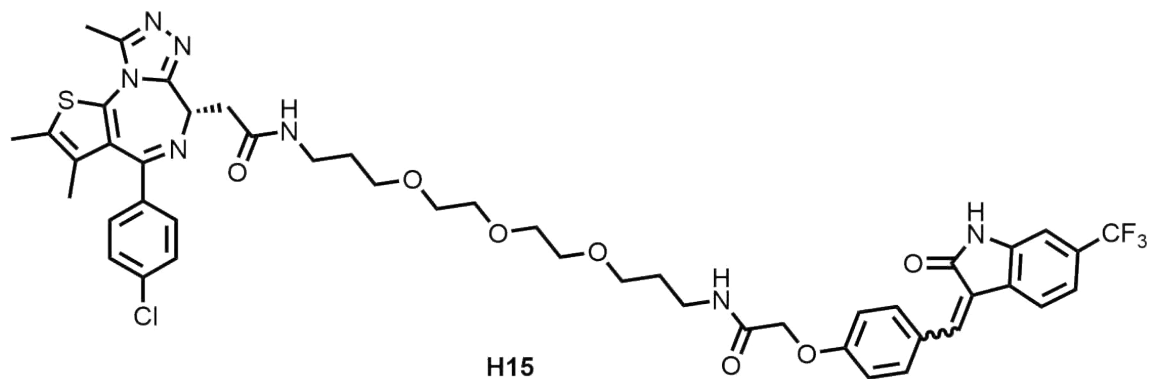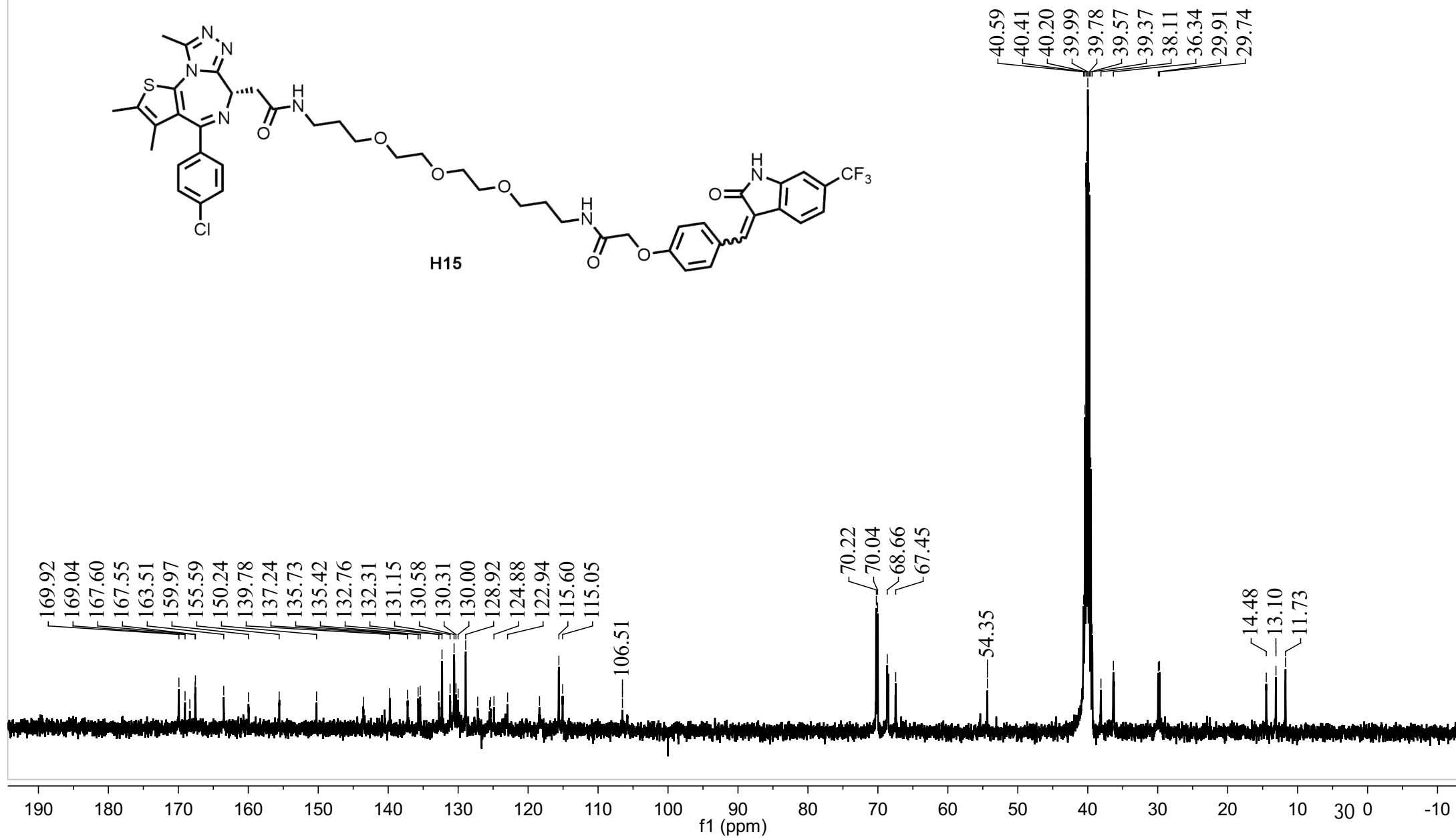

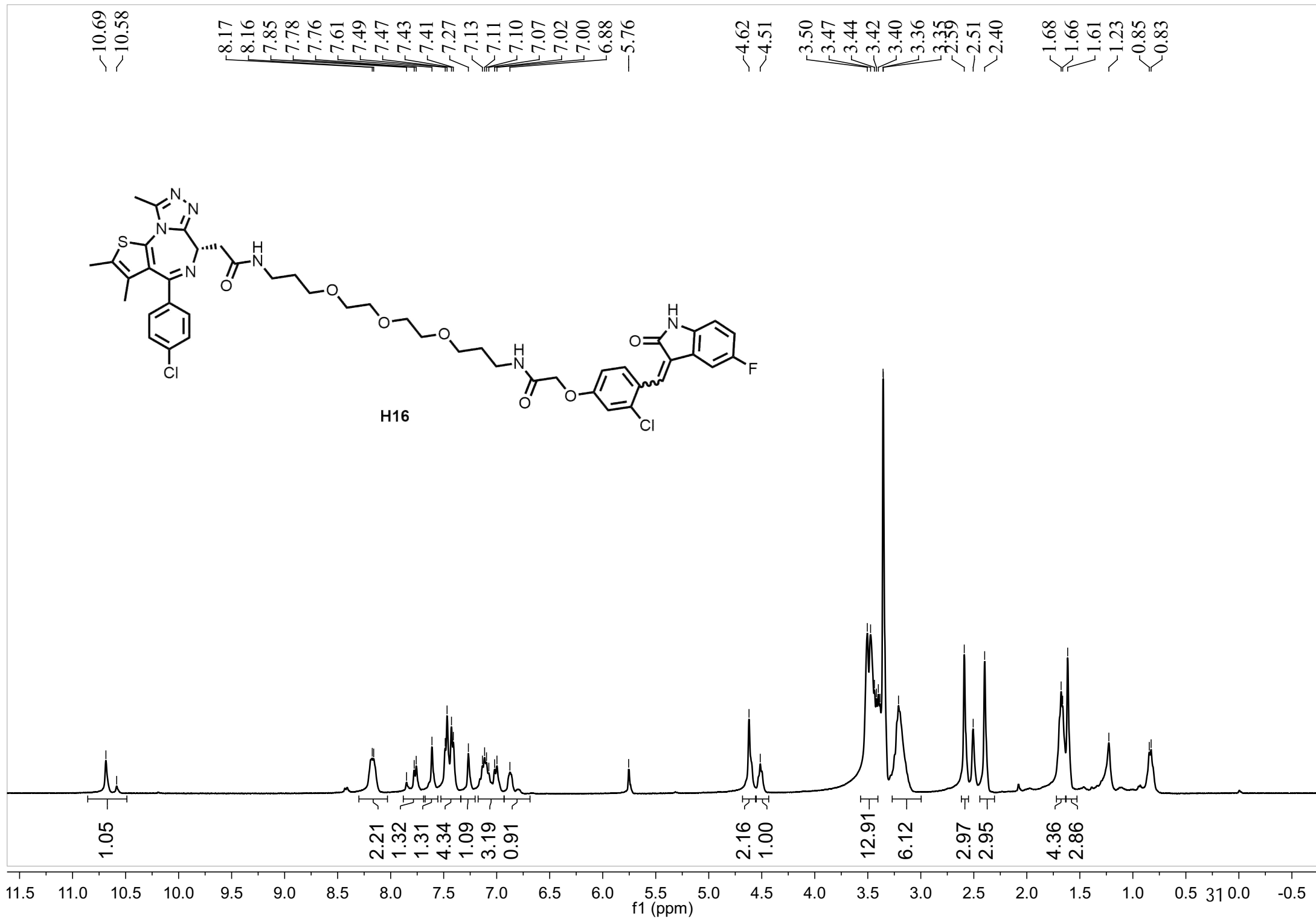

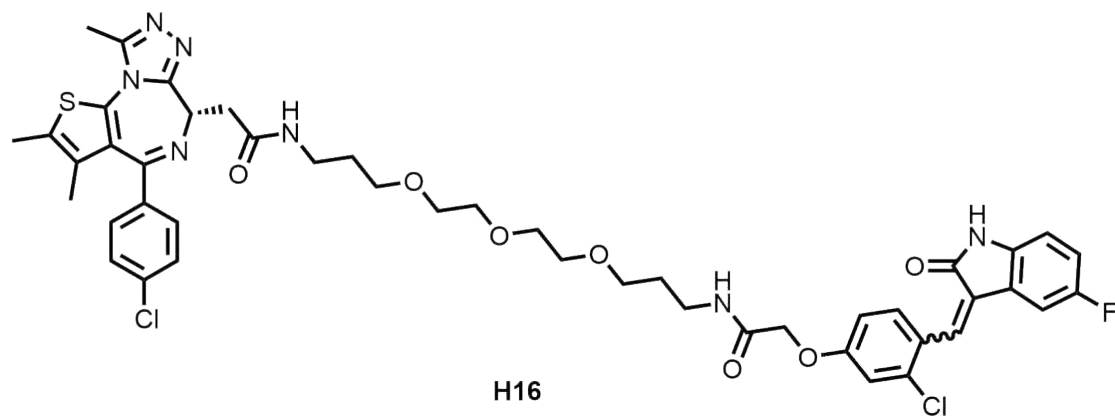

H16

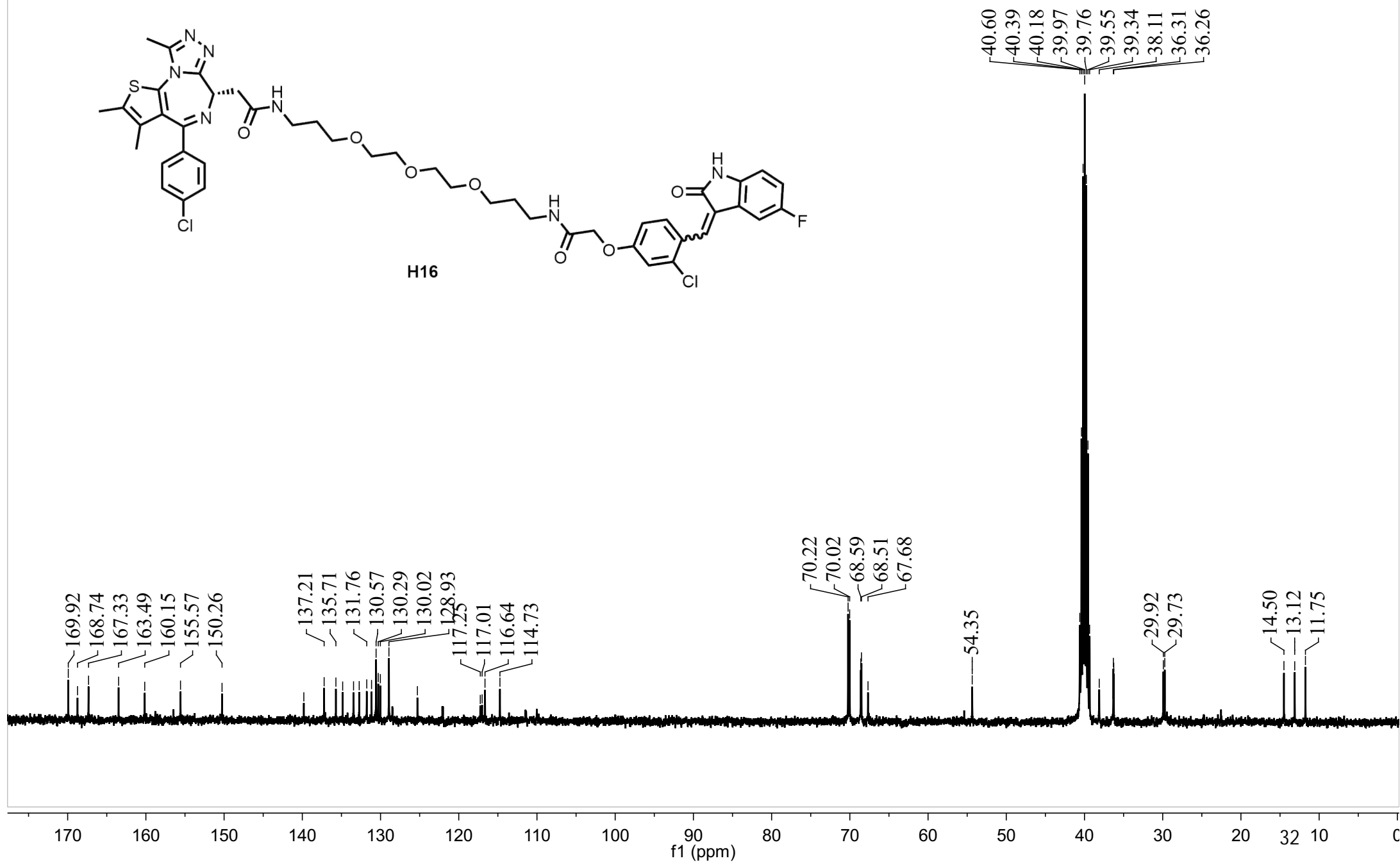

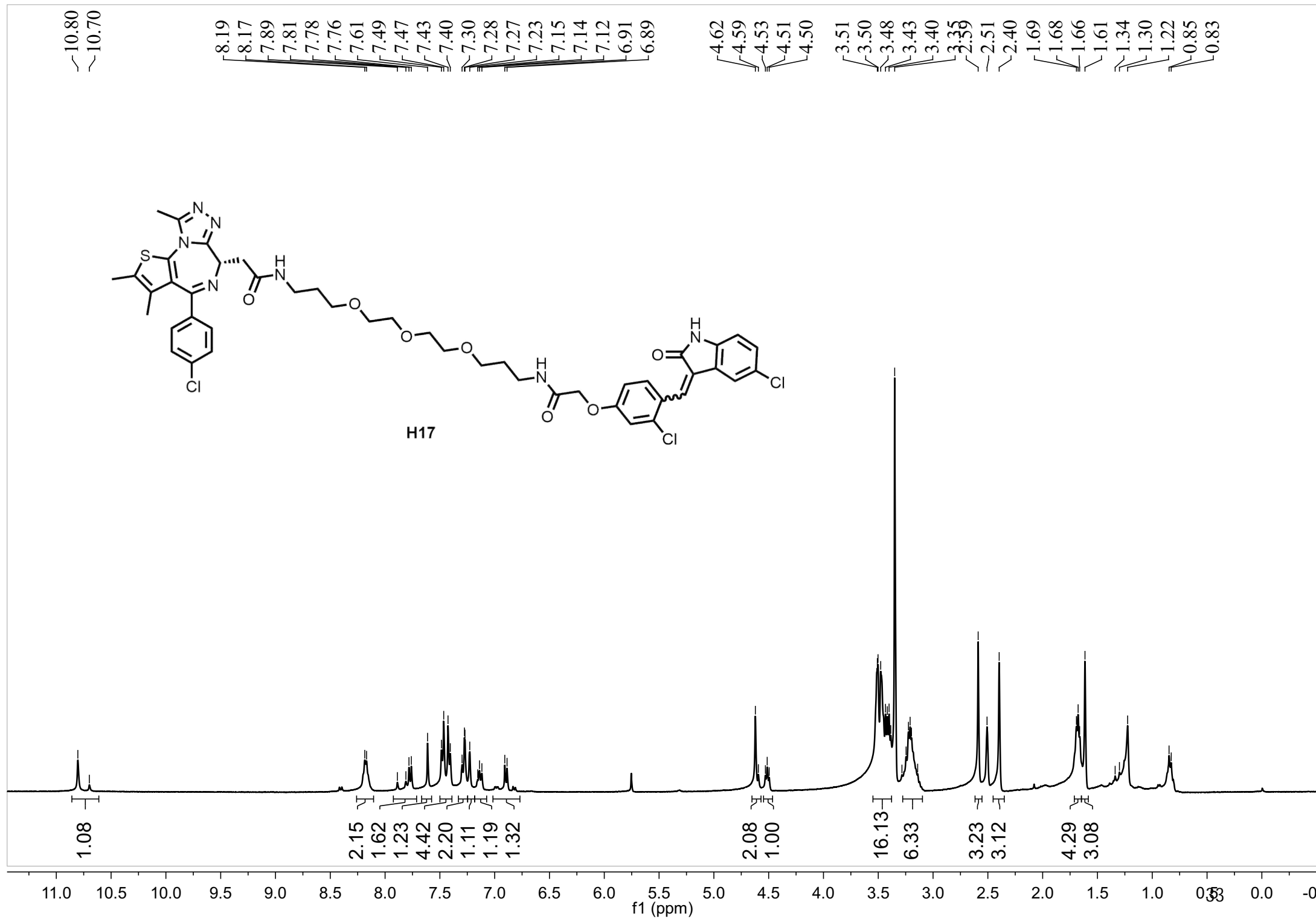

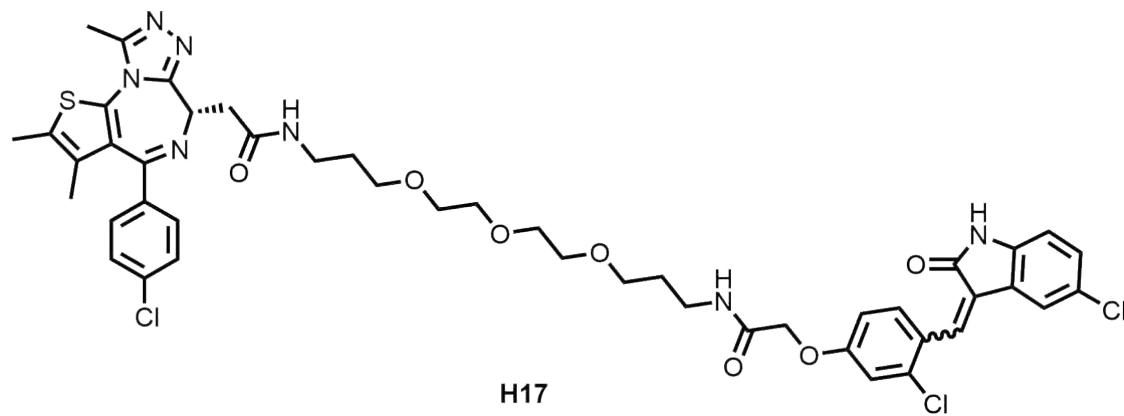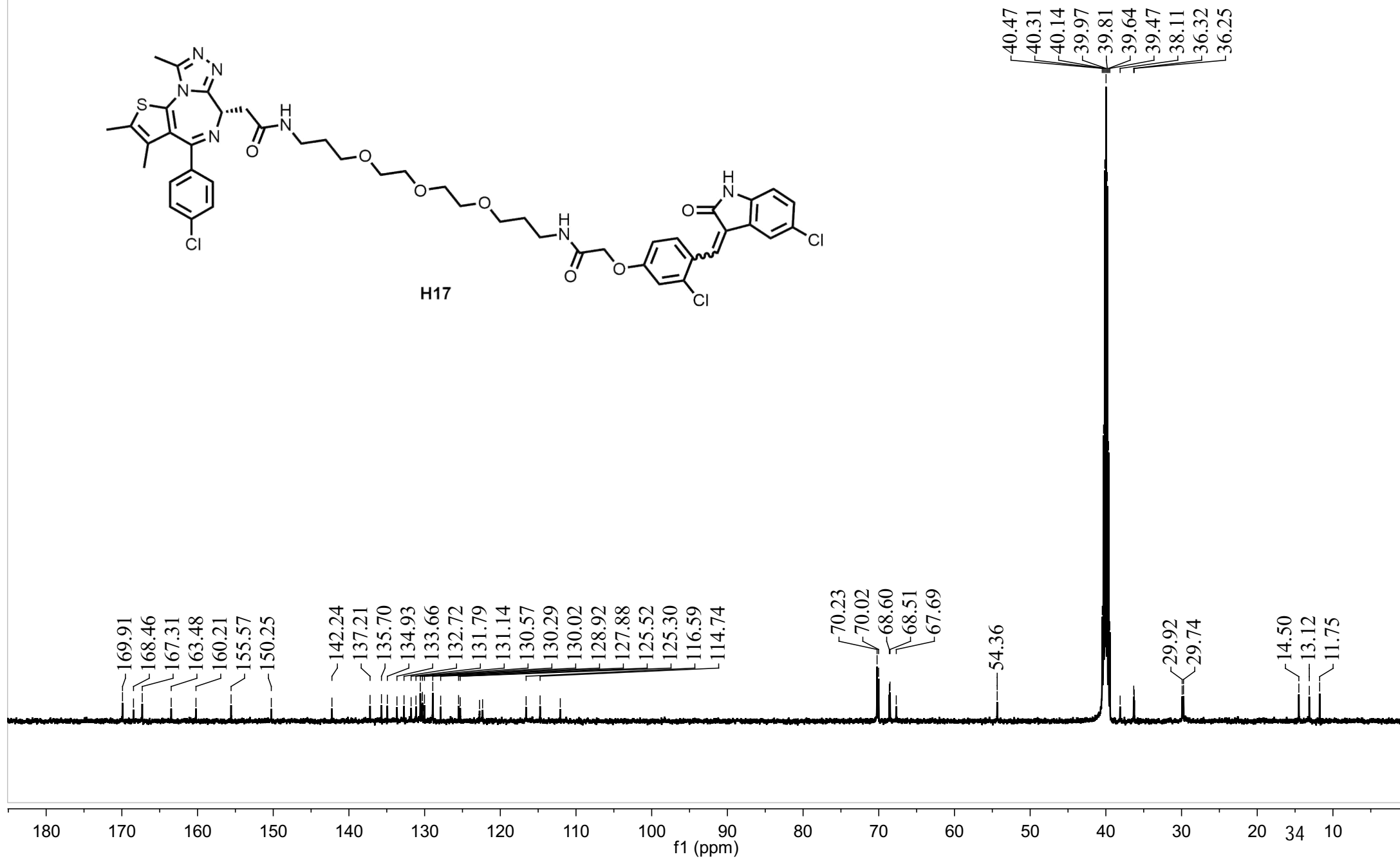

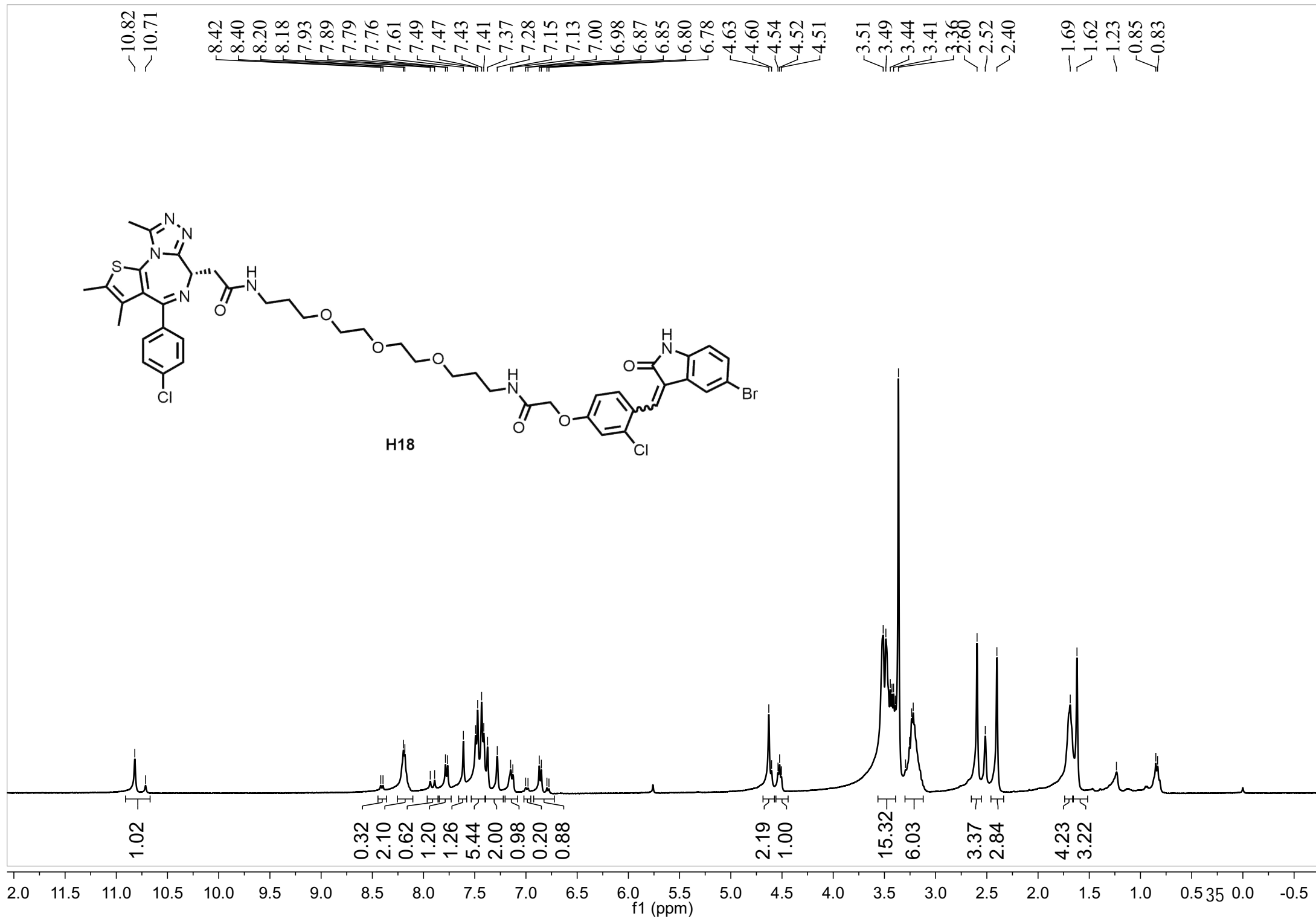

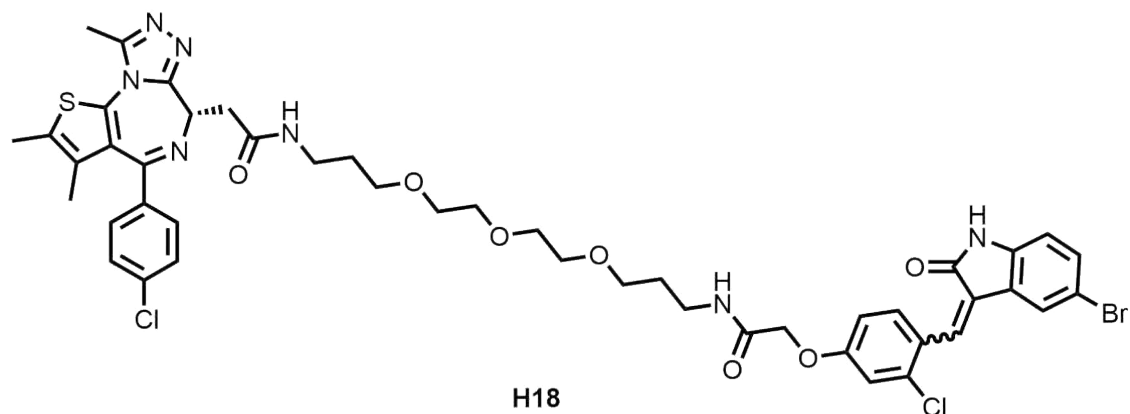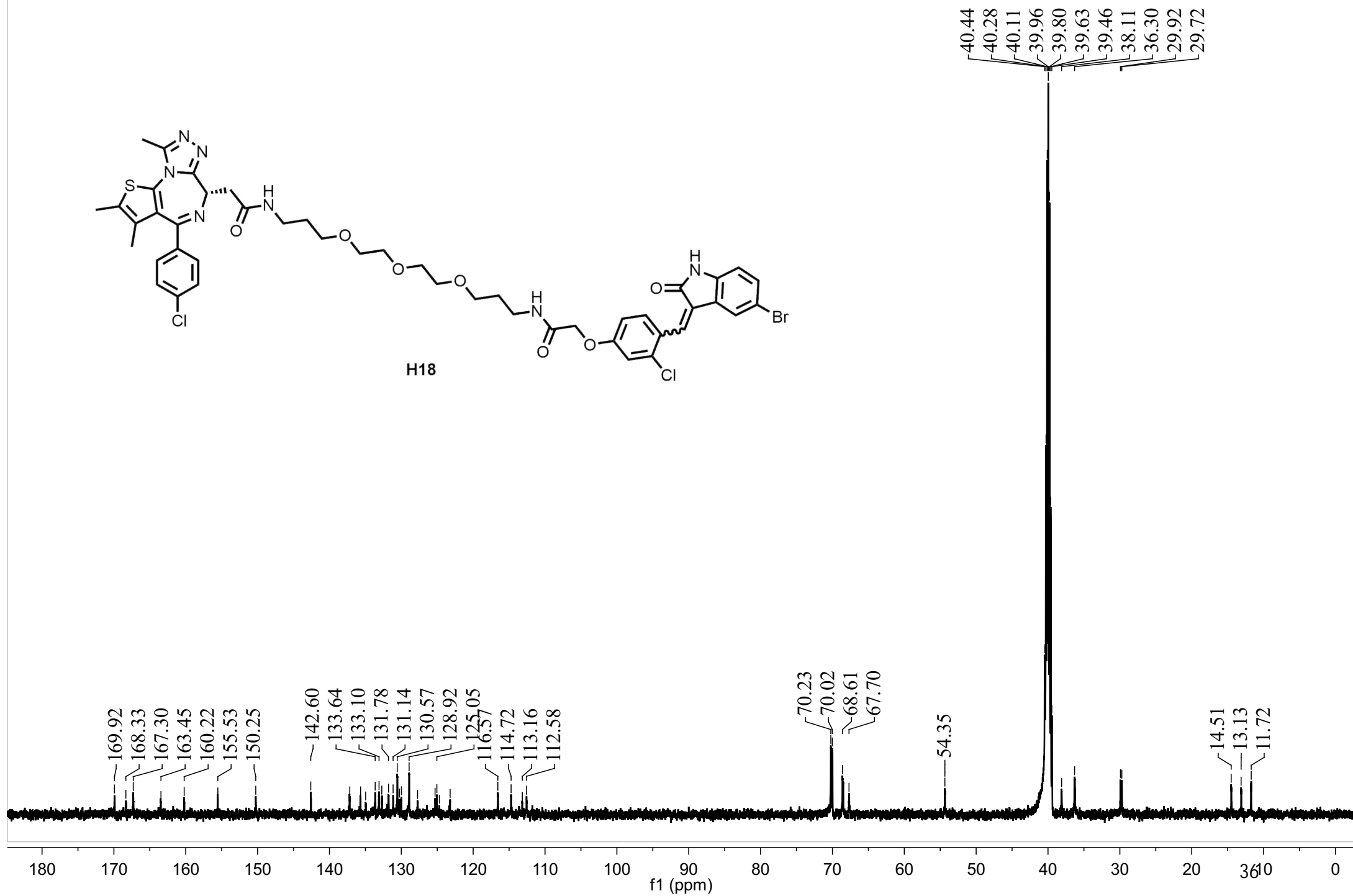

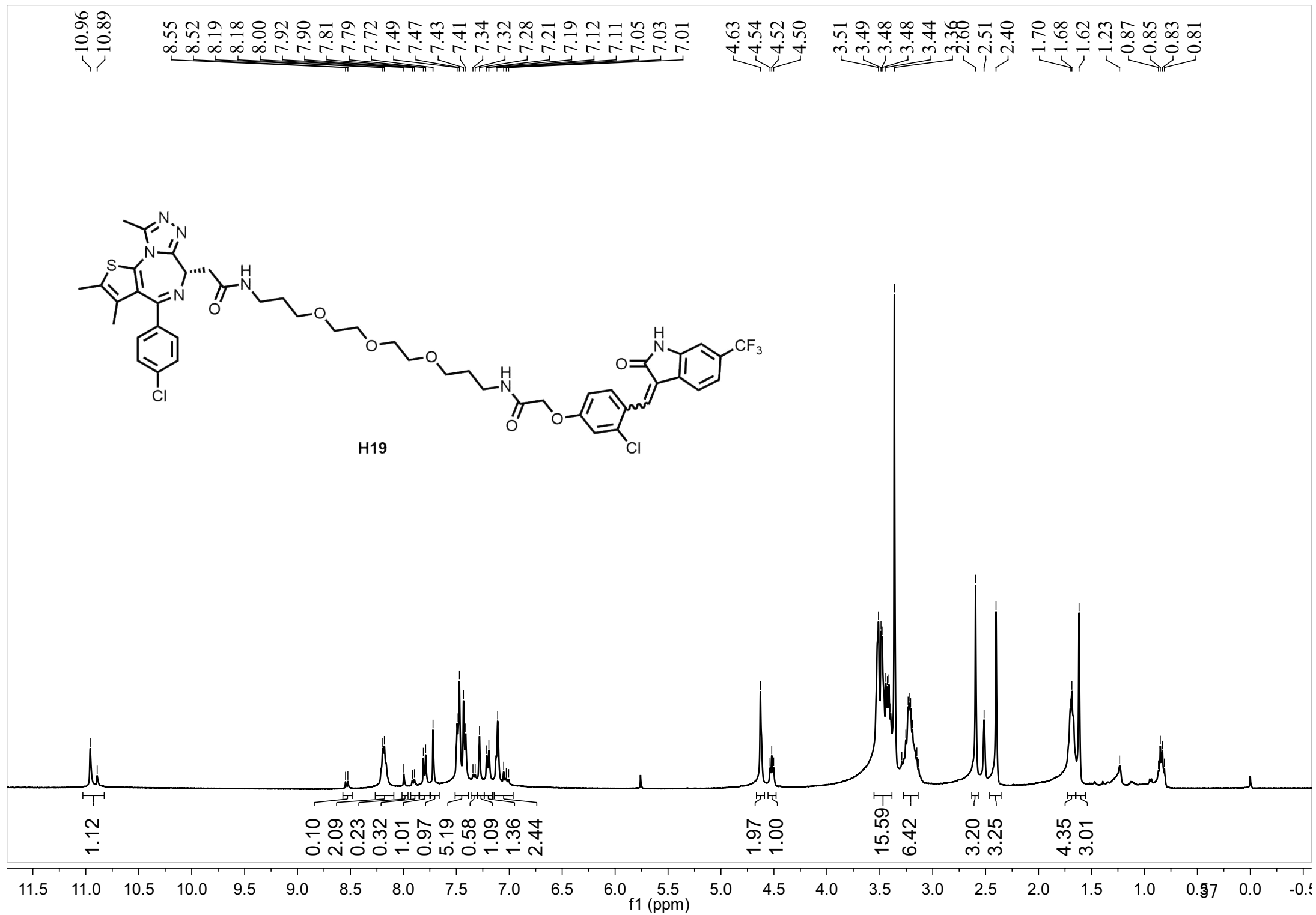

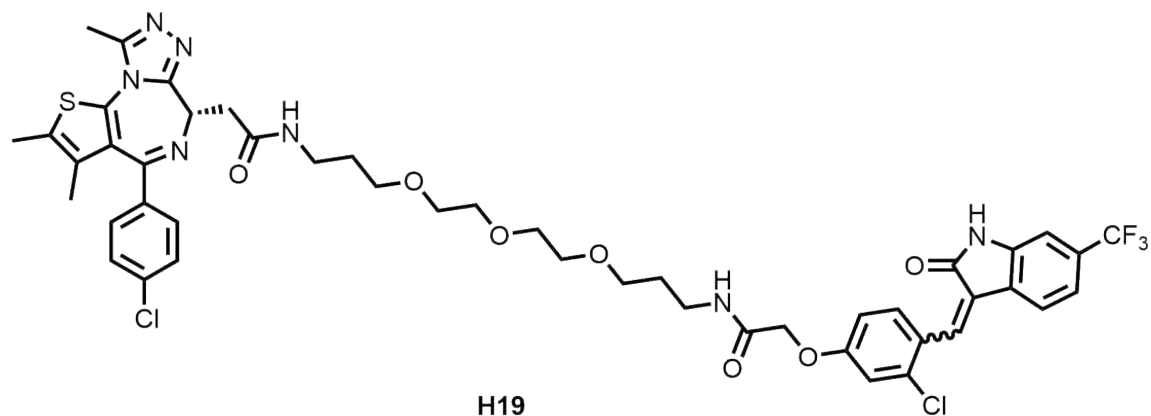

H19

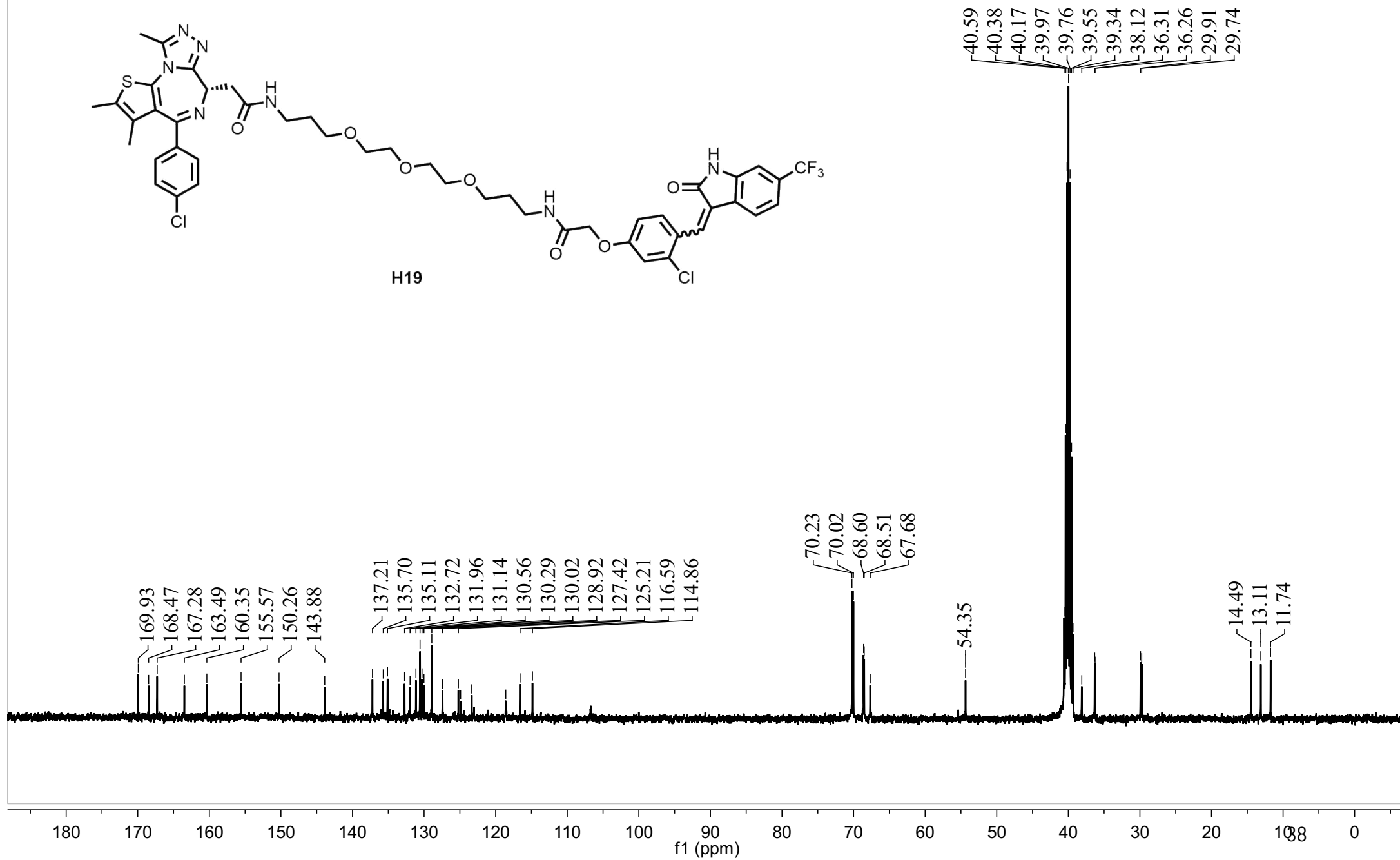

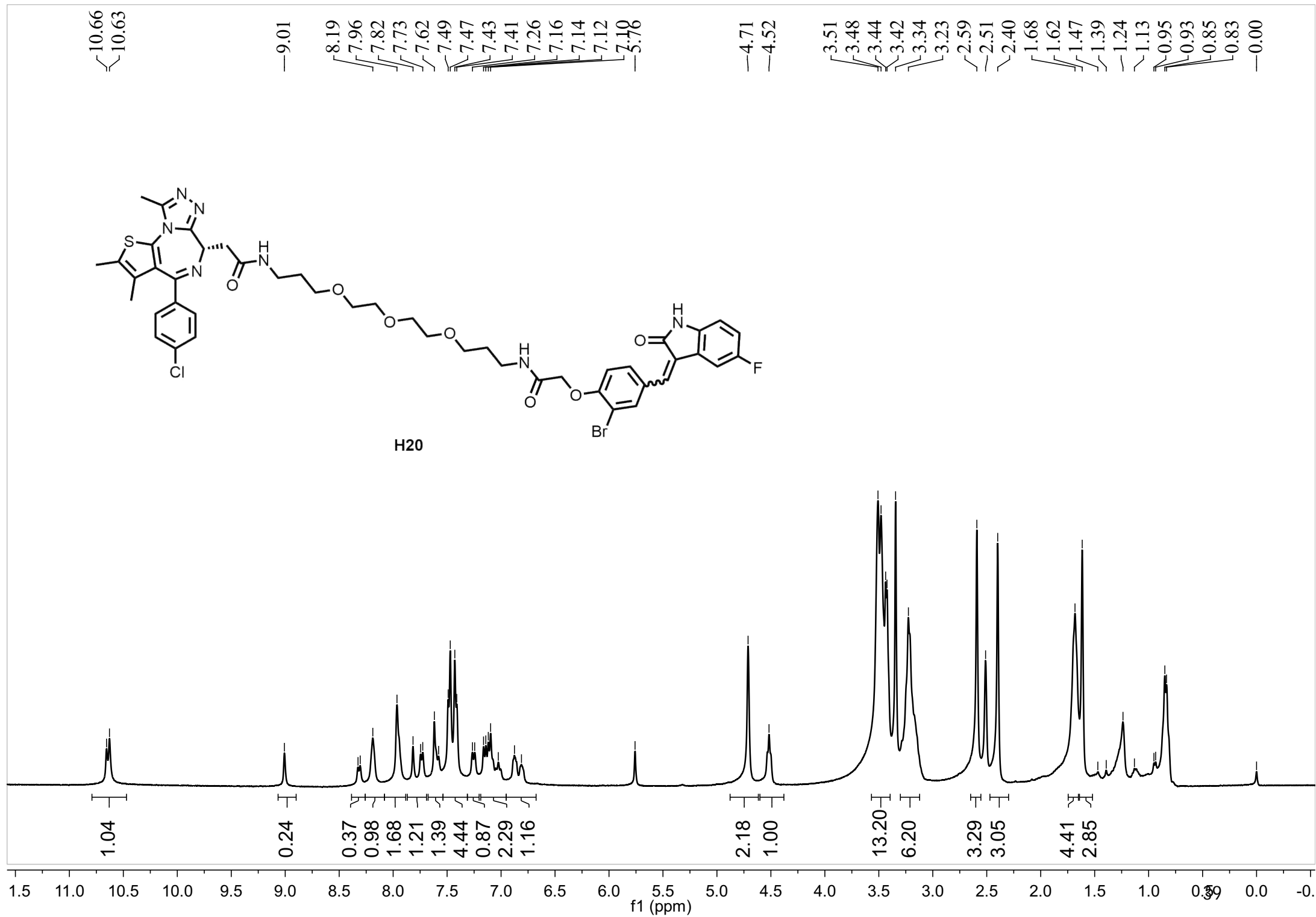

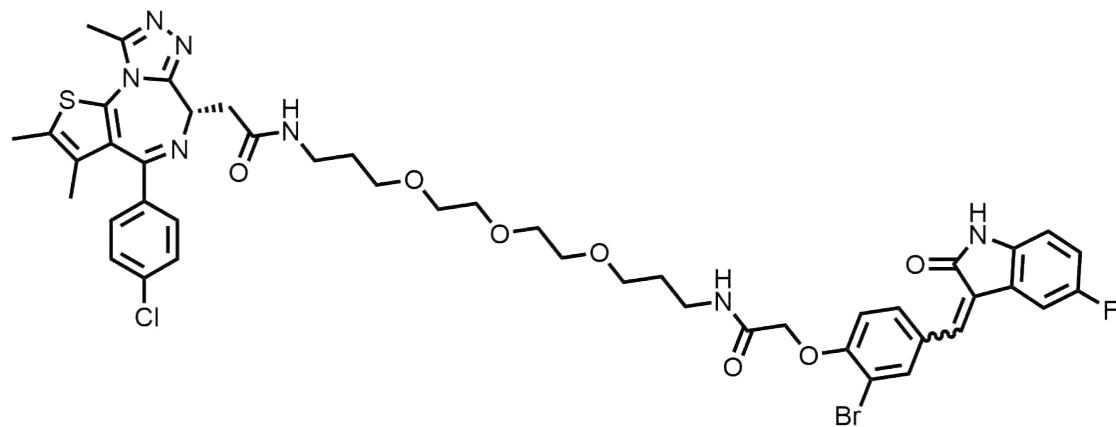

H20

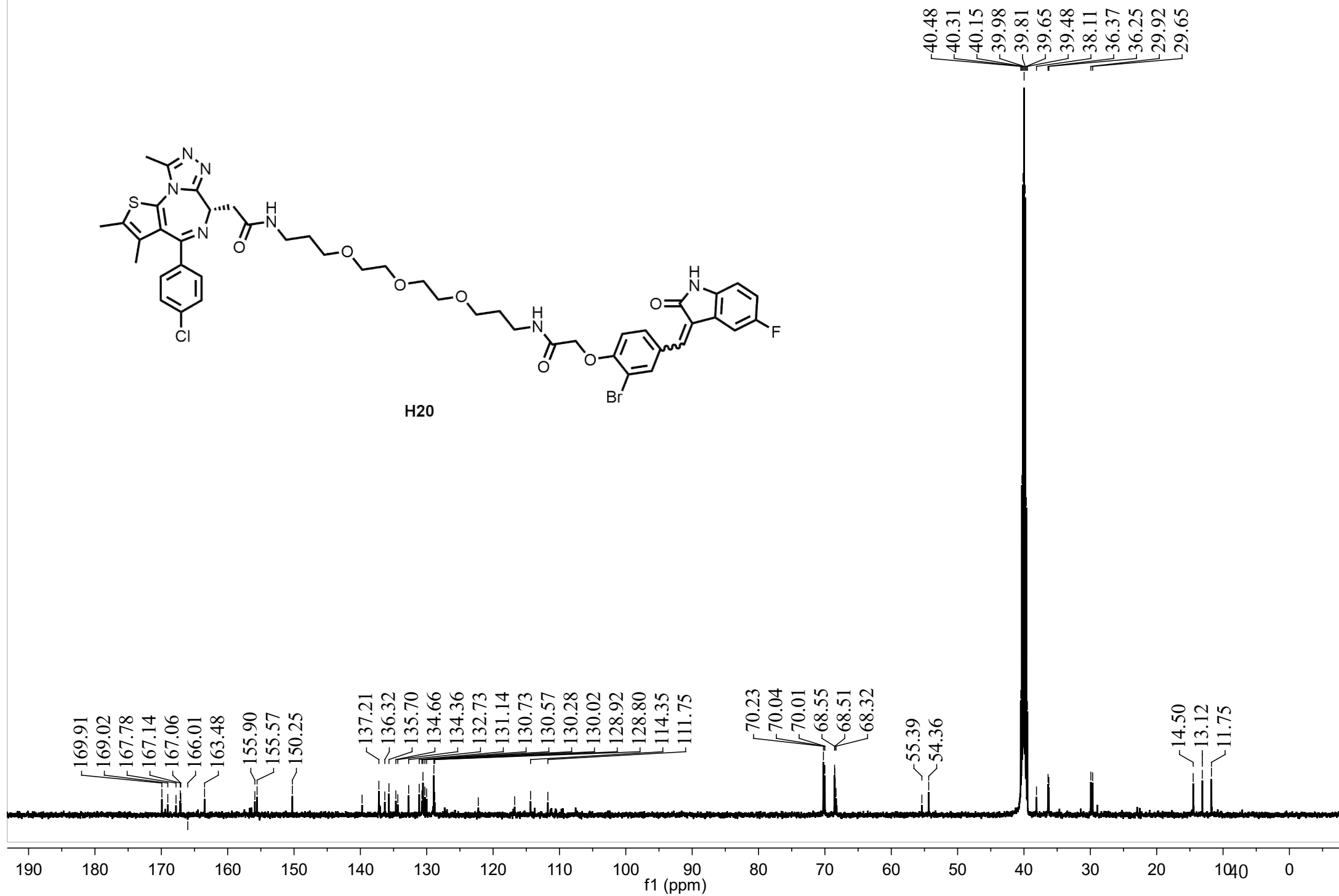

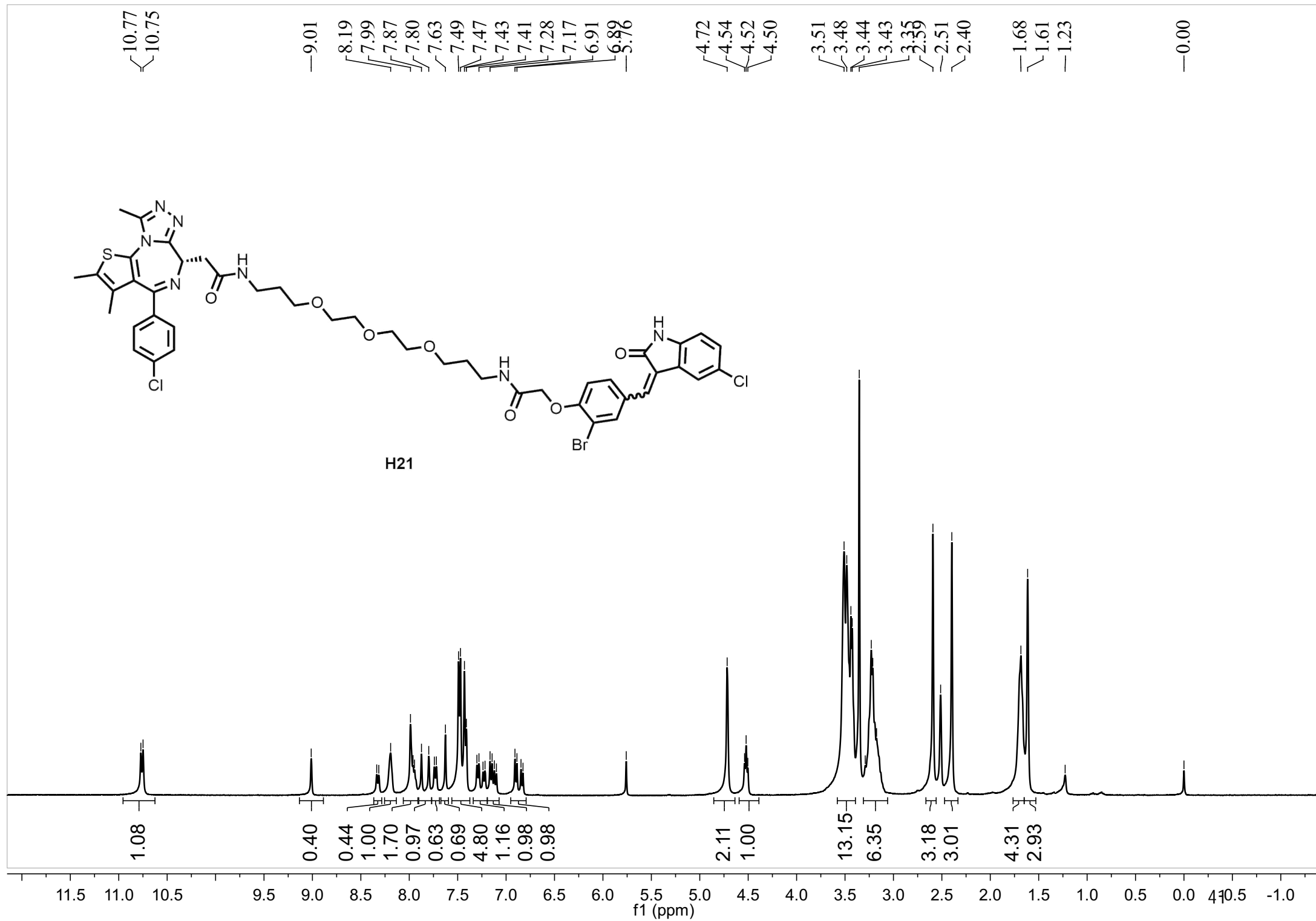

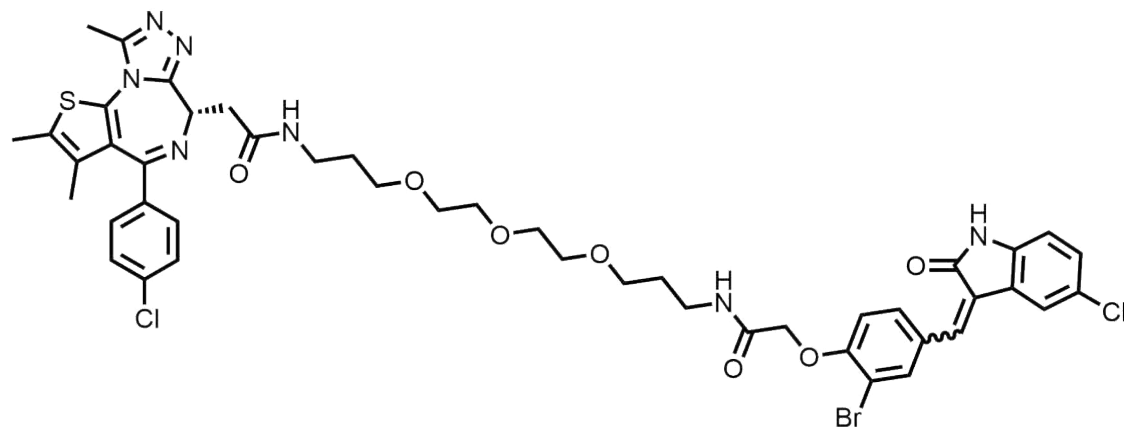

H21

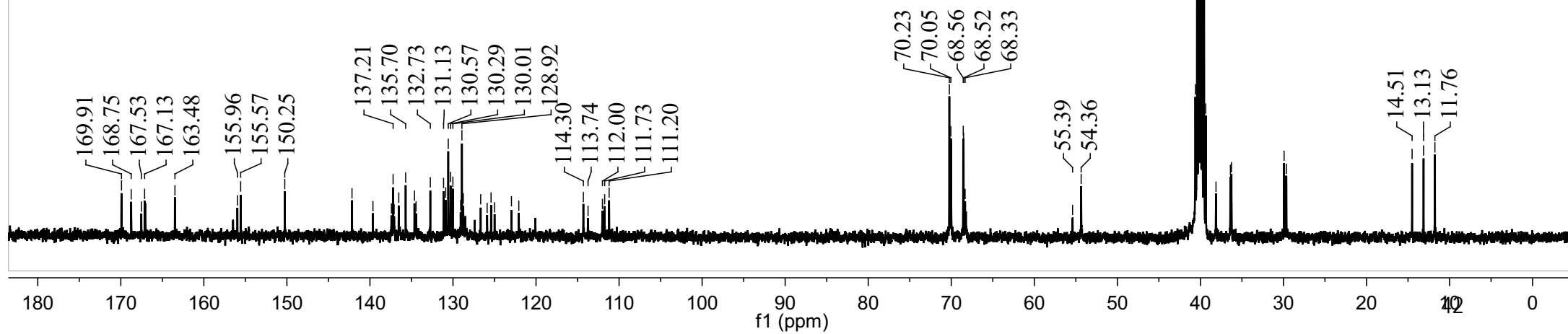

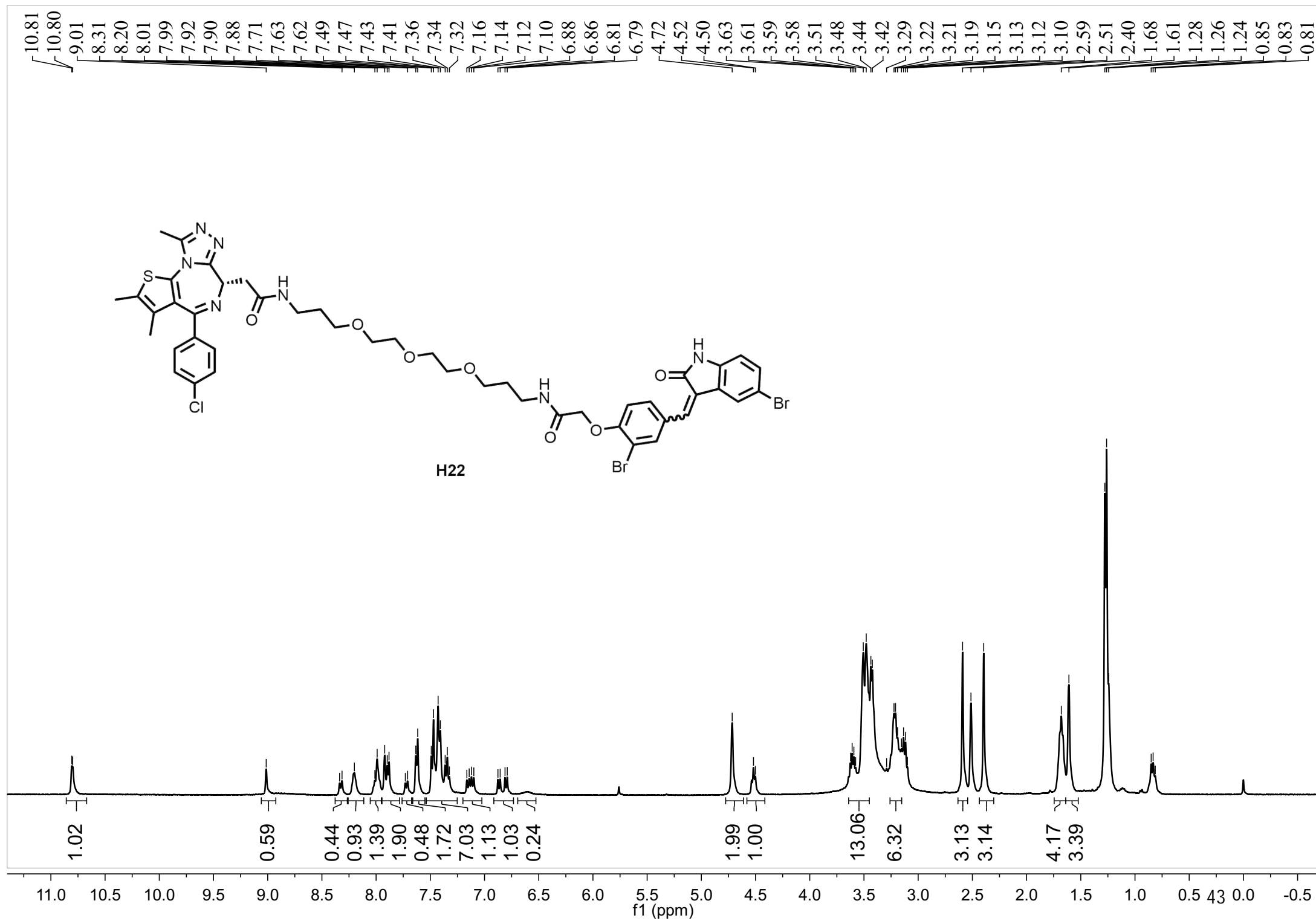

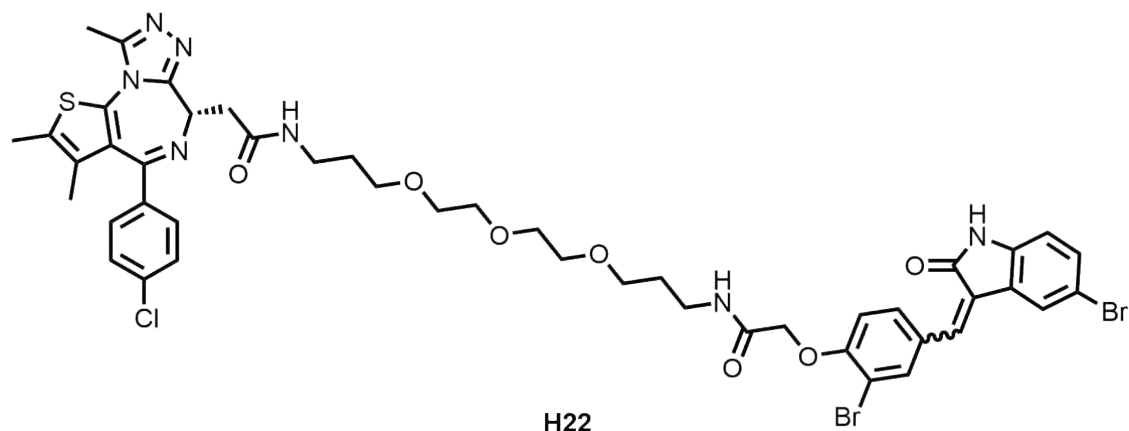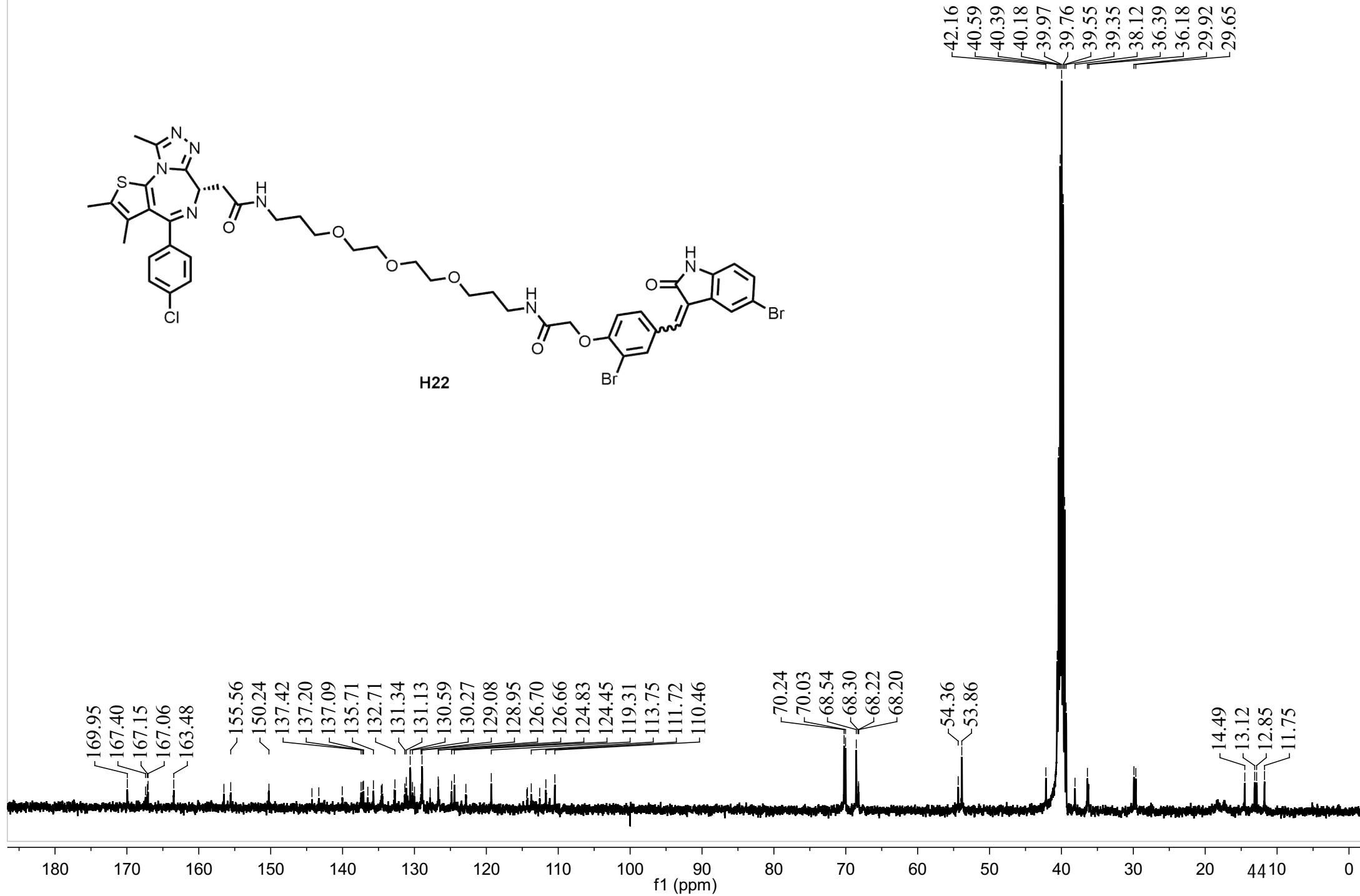

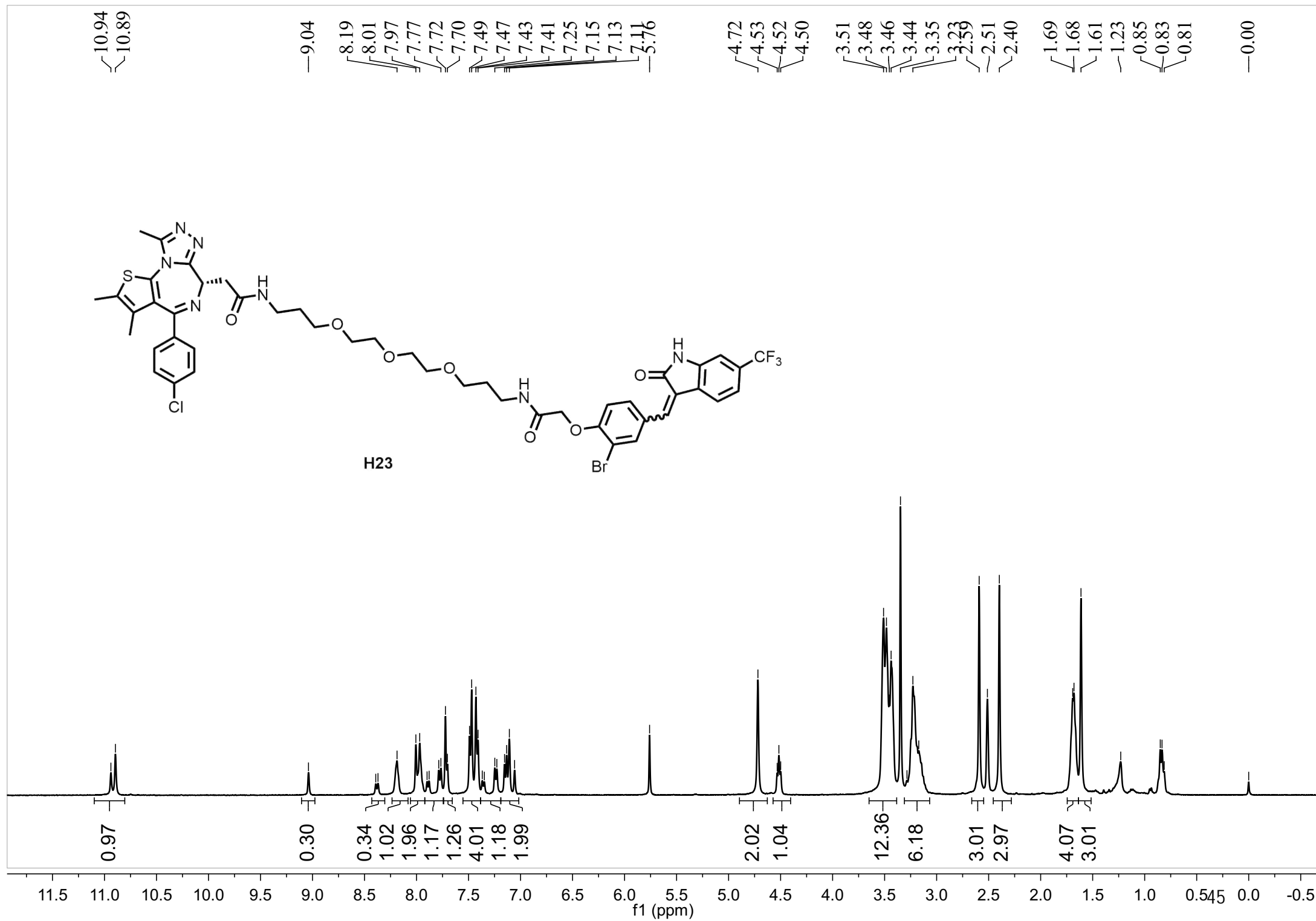

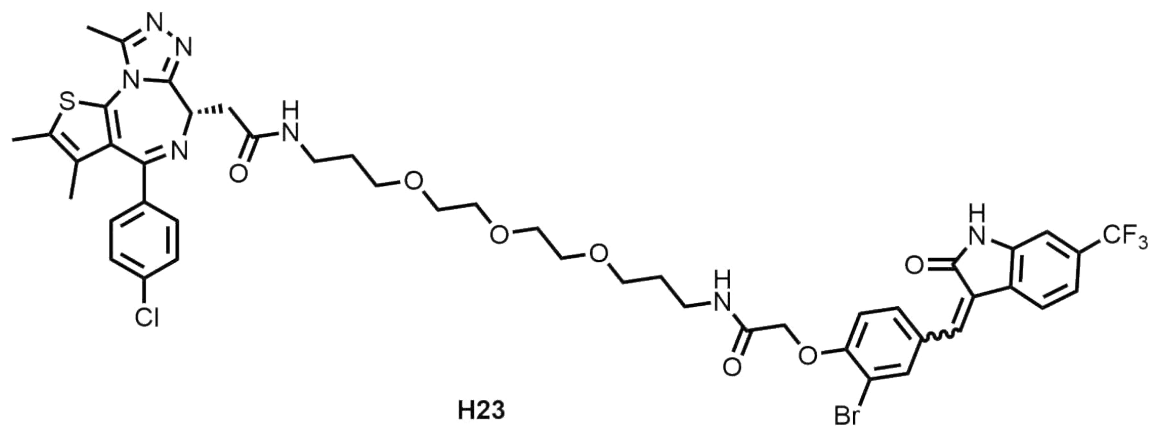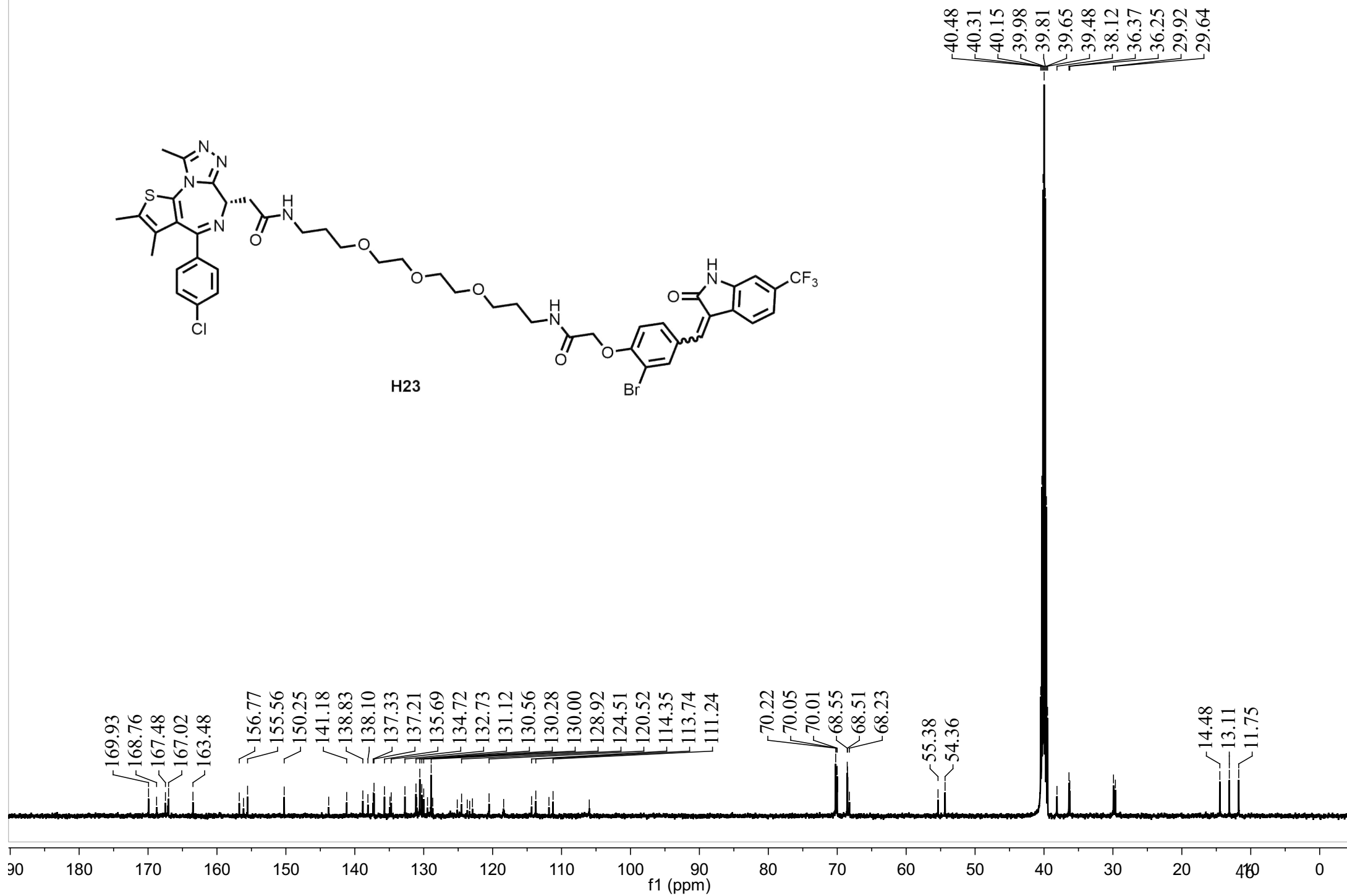

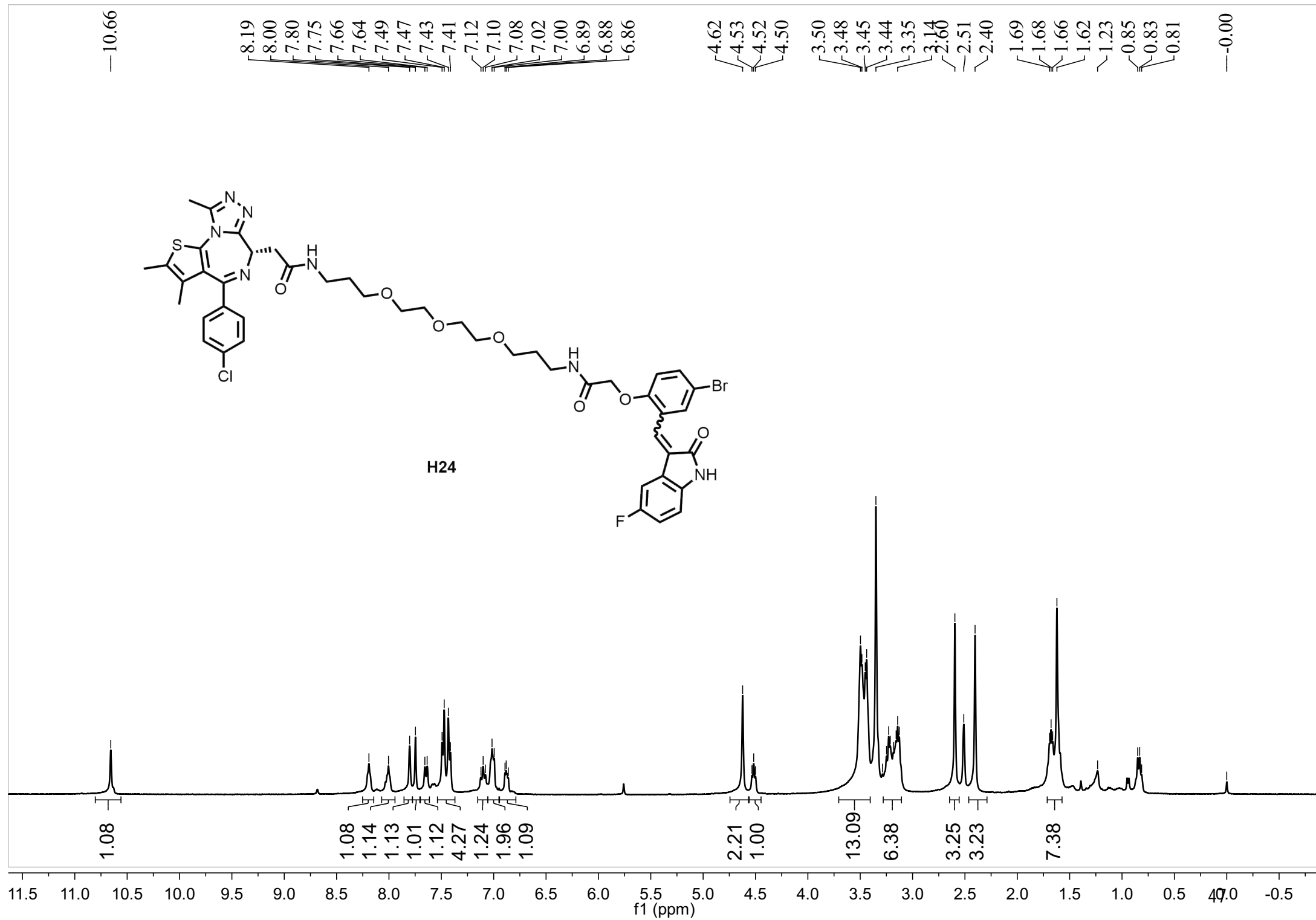

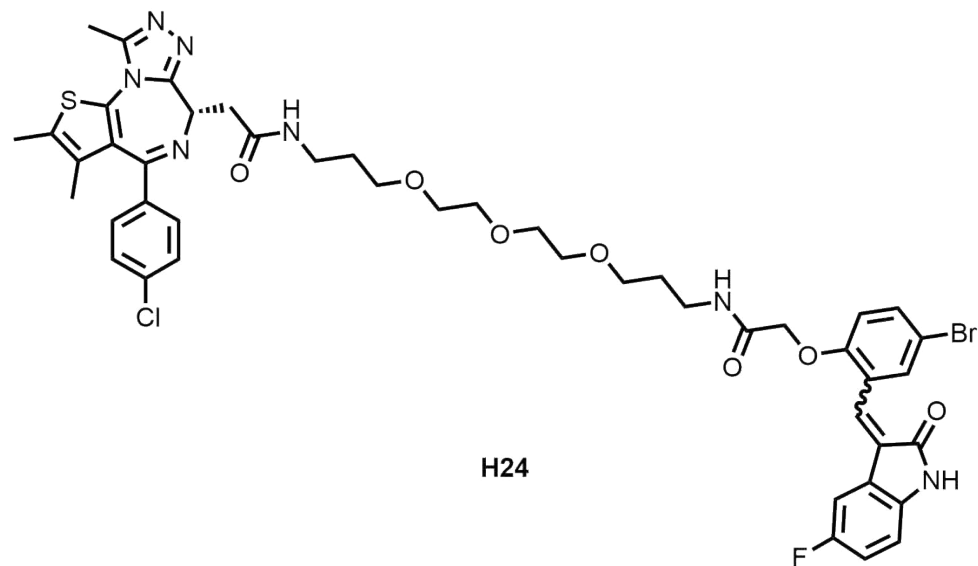

H24

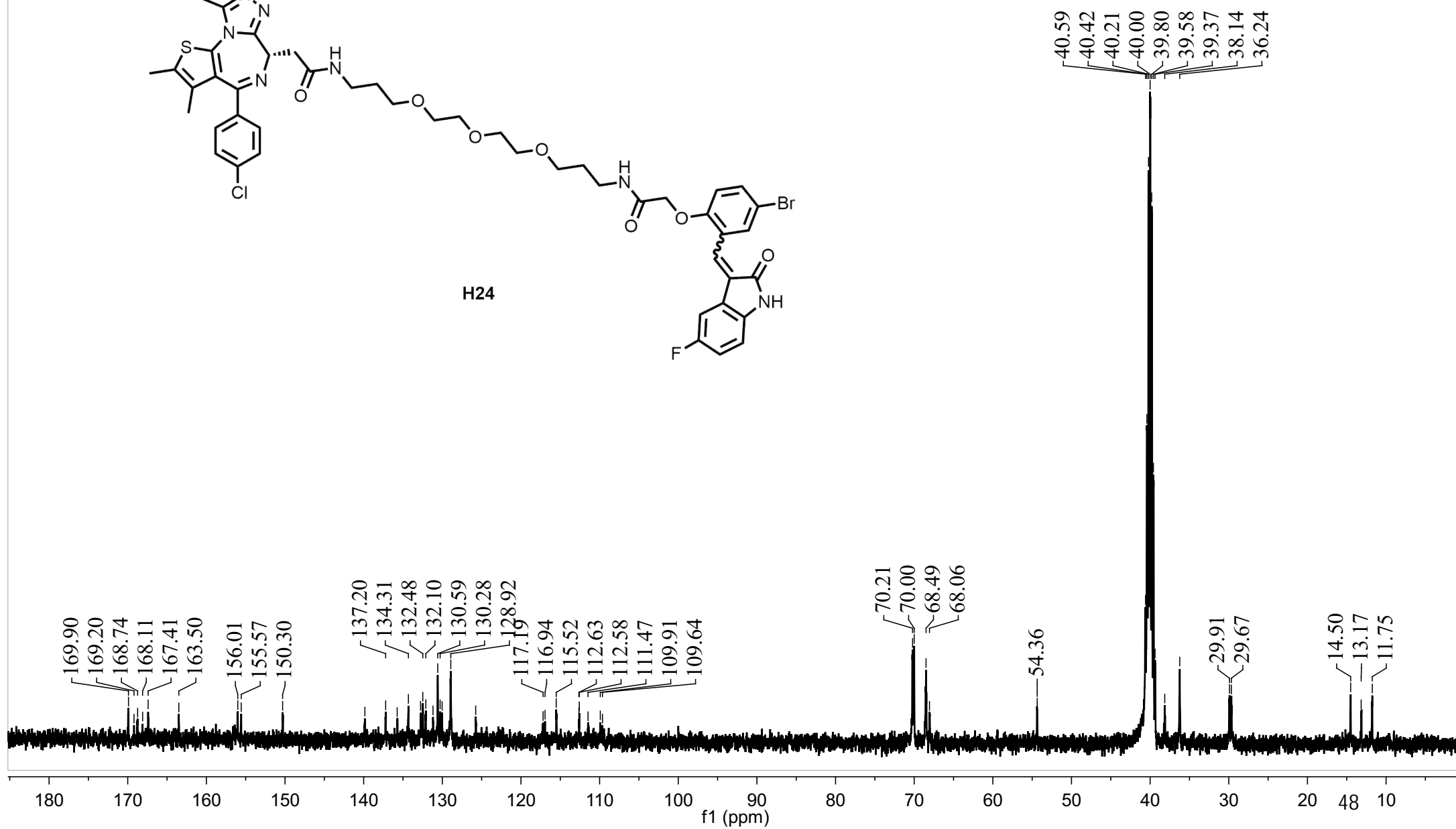

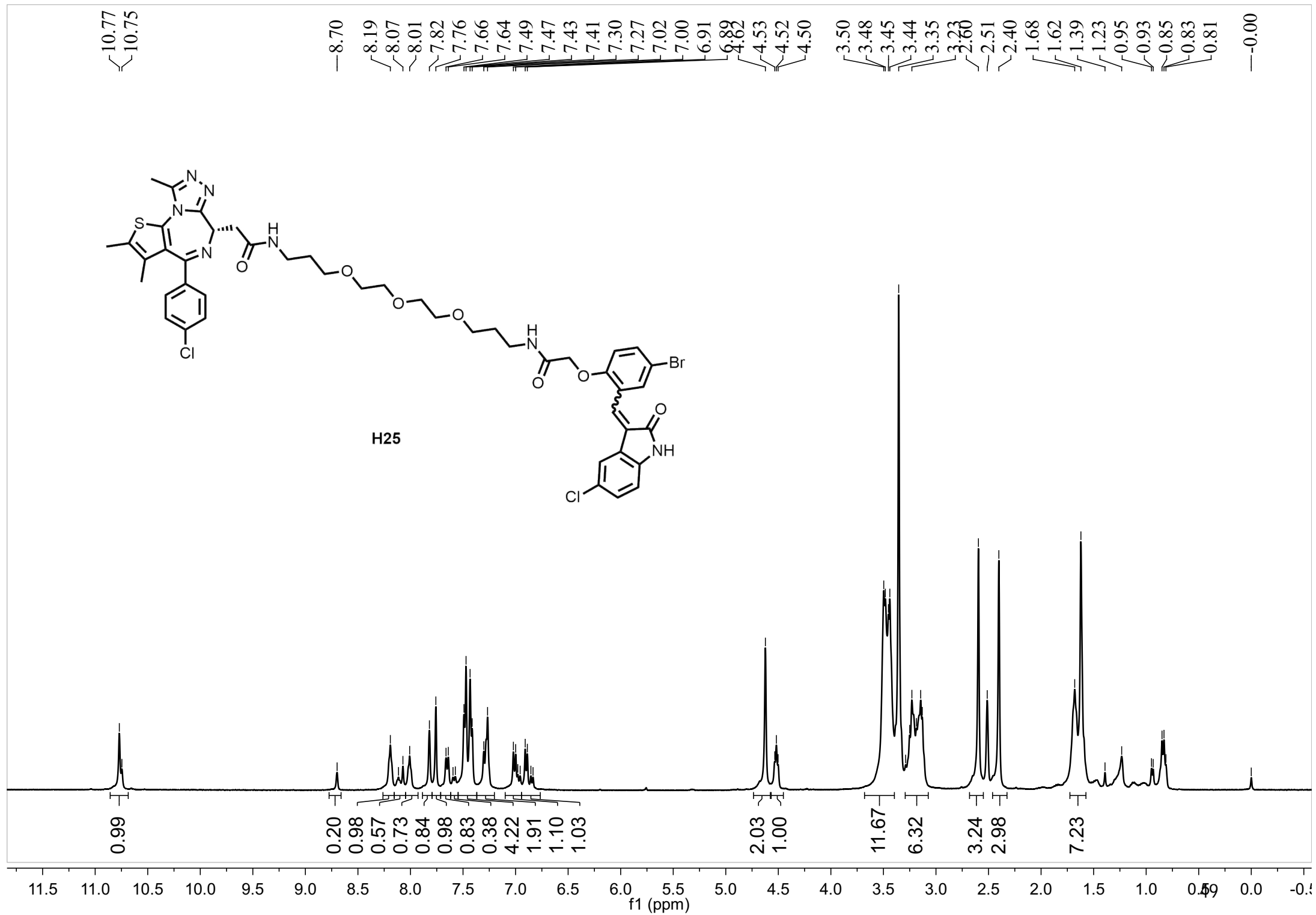

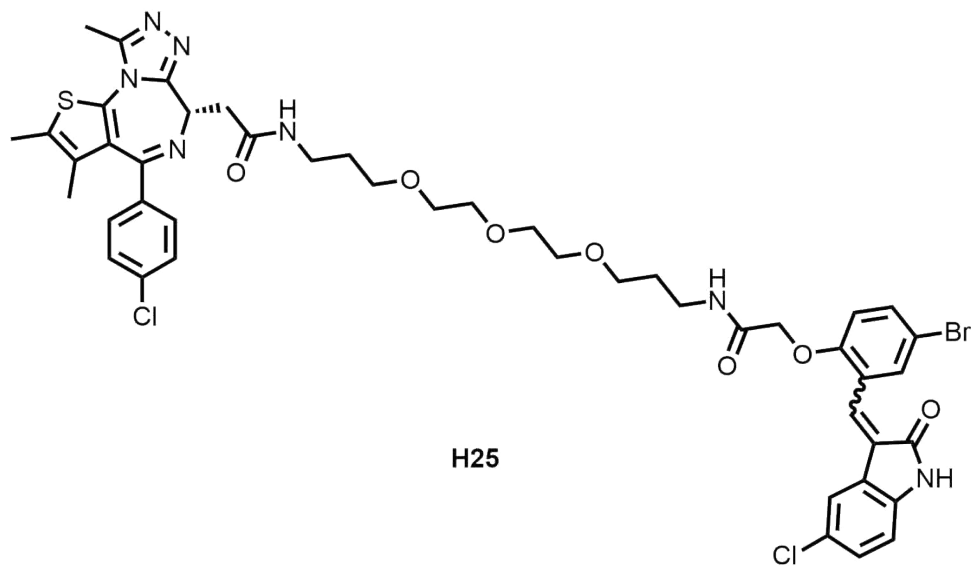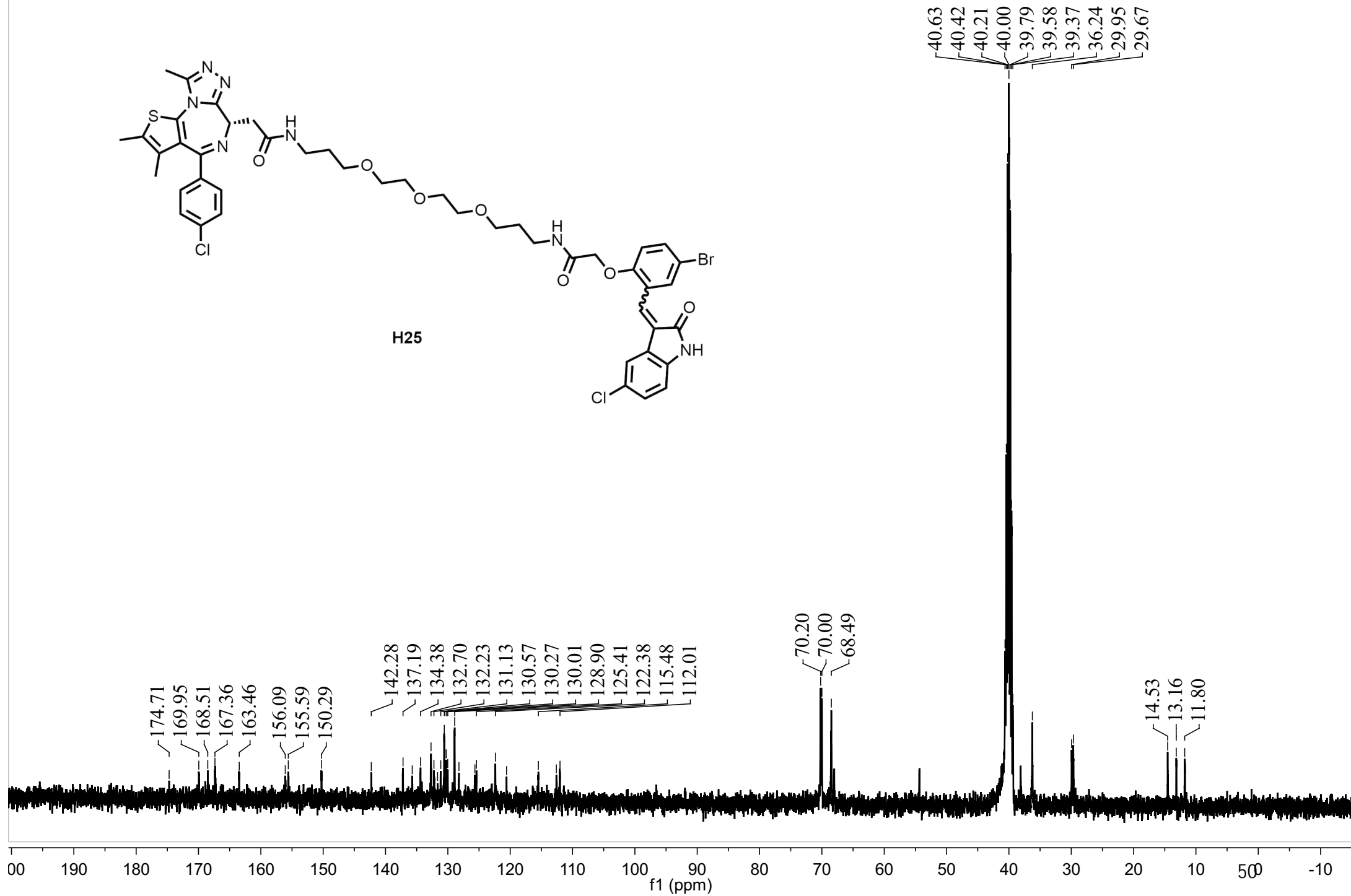

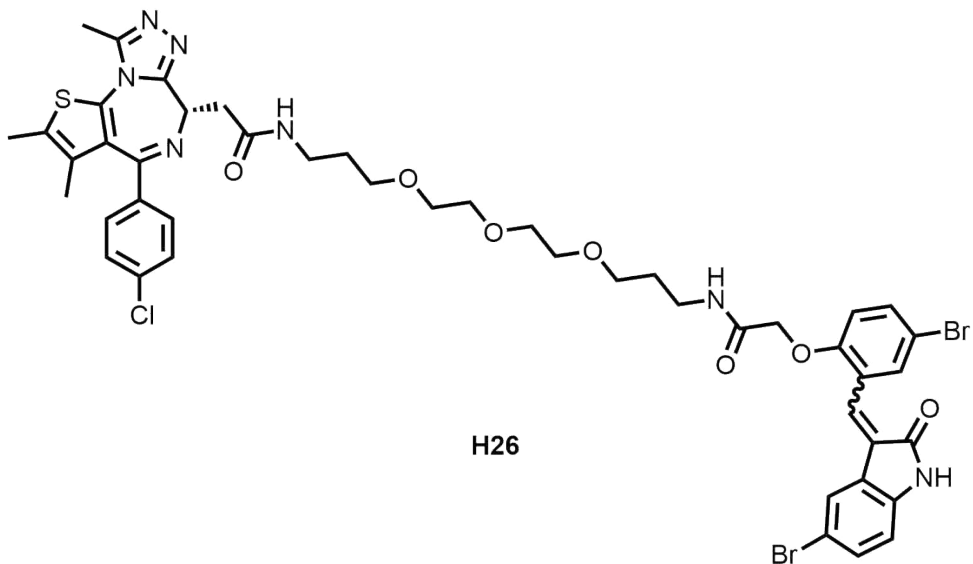

H26

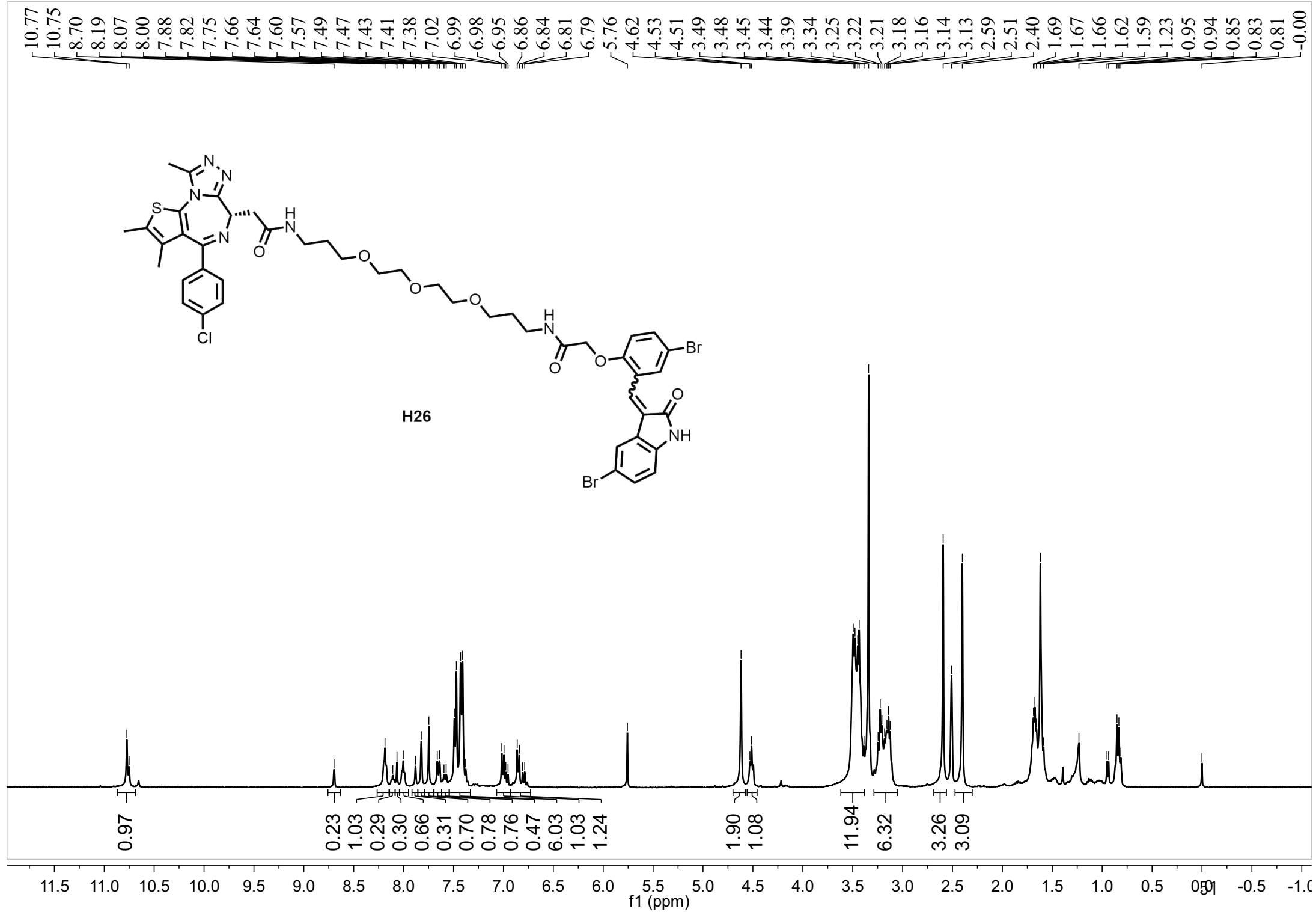

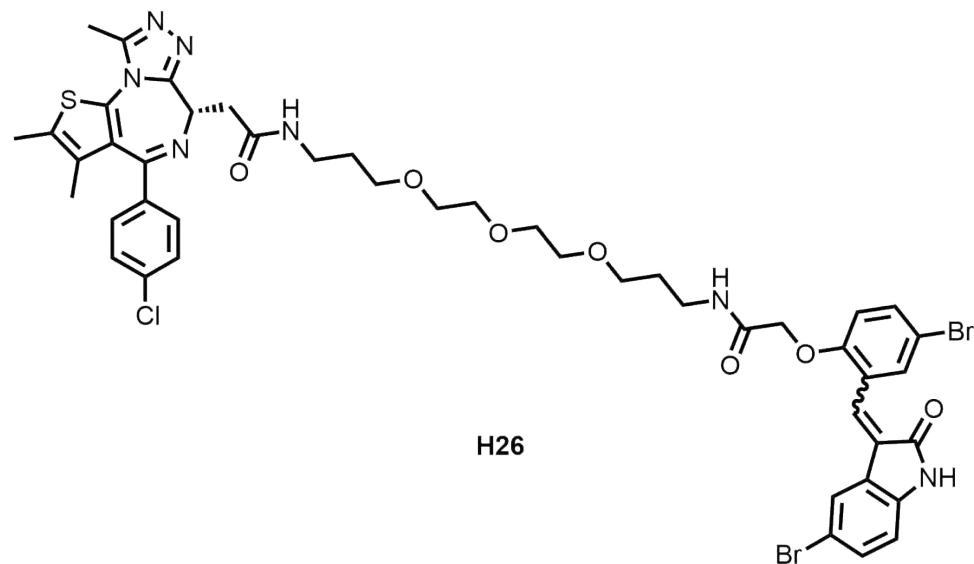

H26

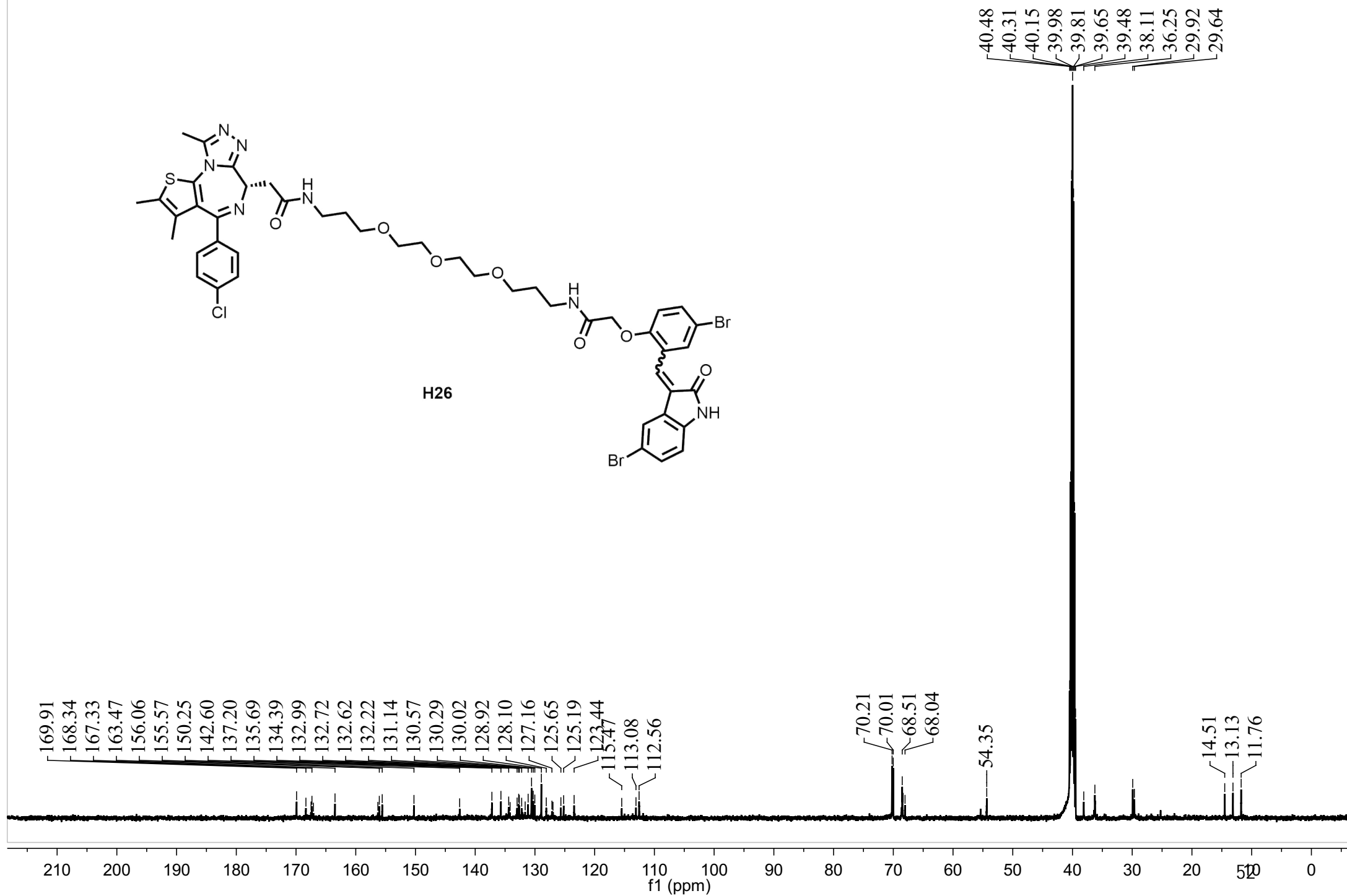

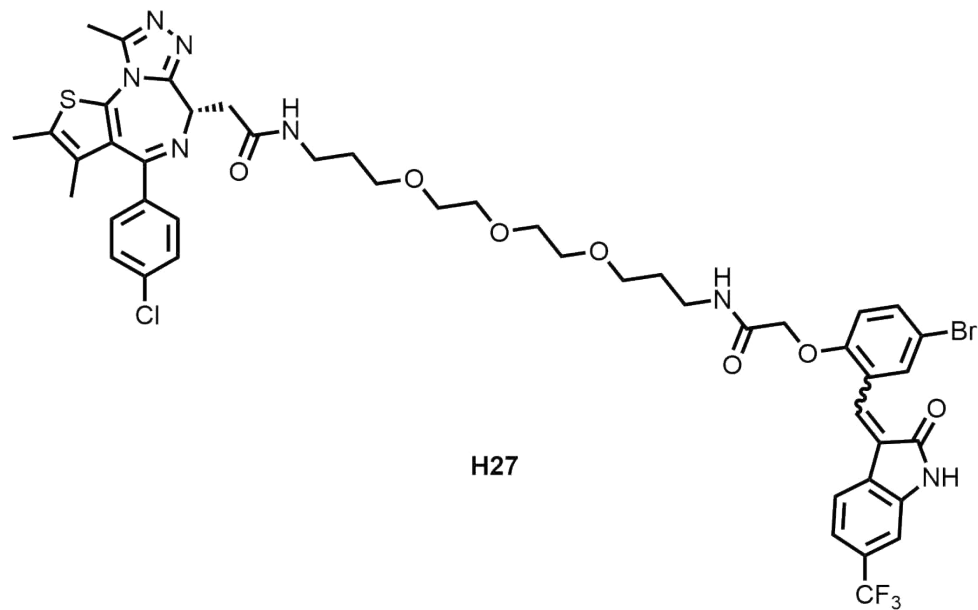

H27

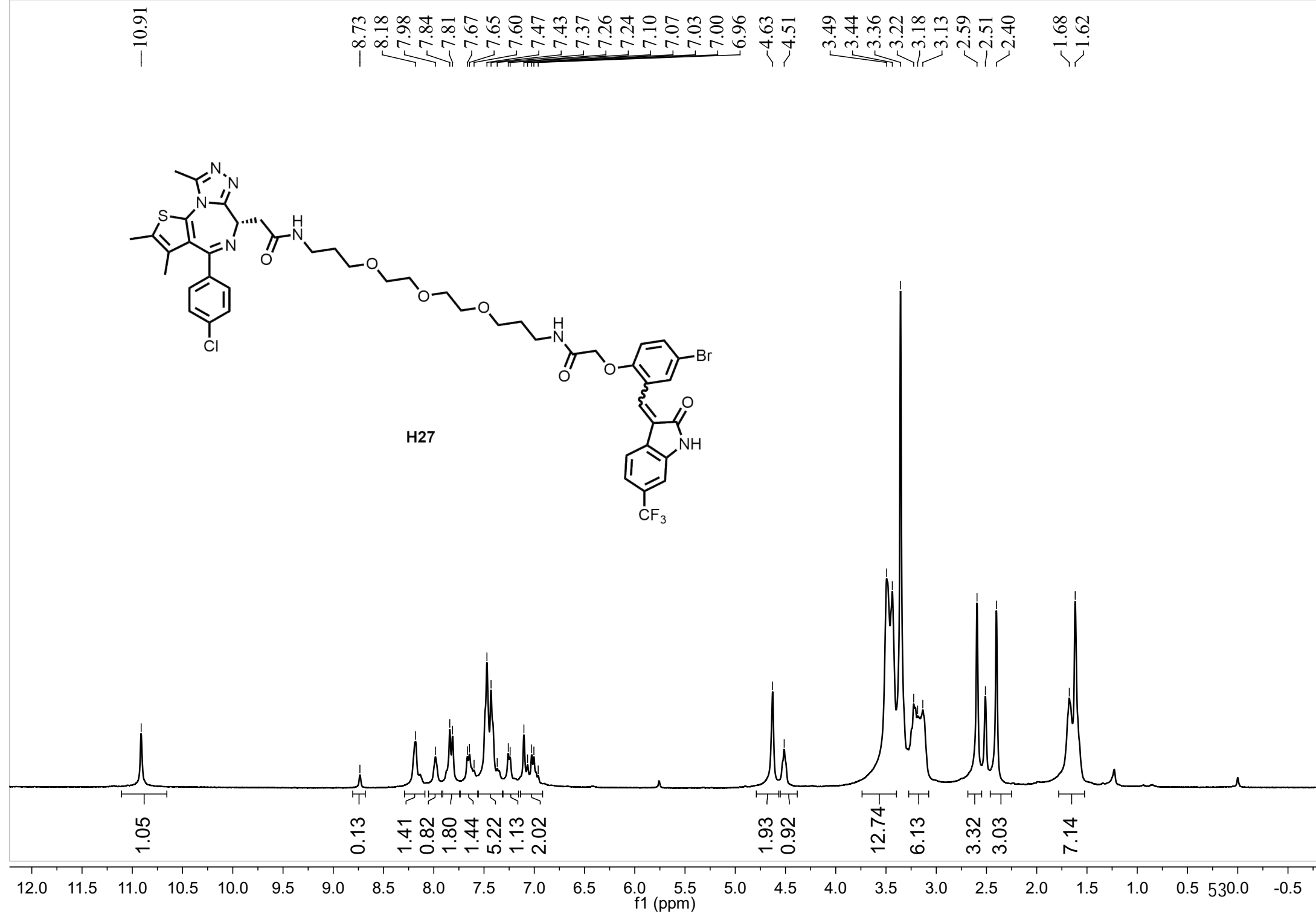

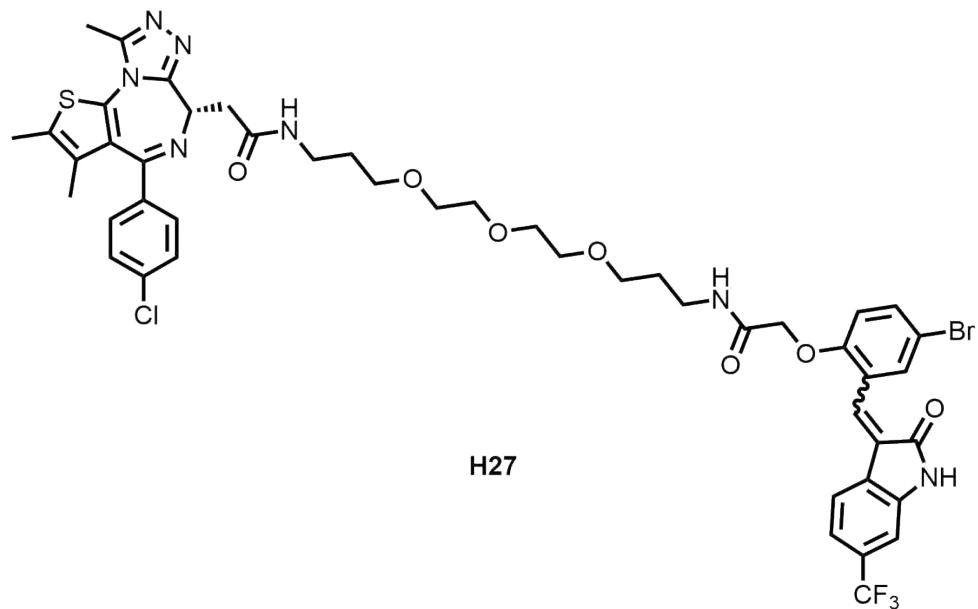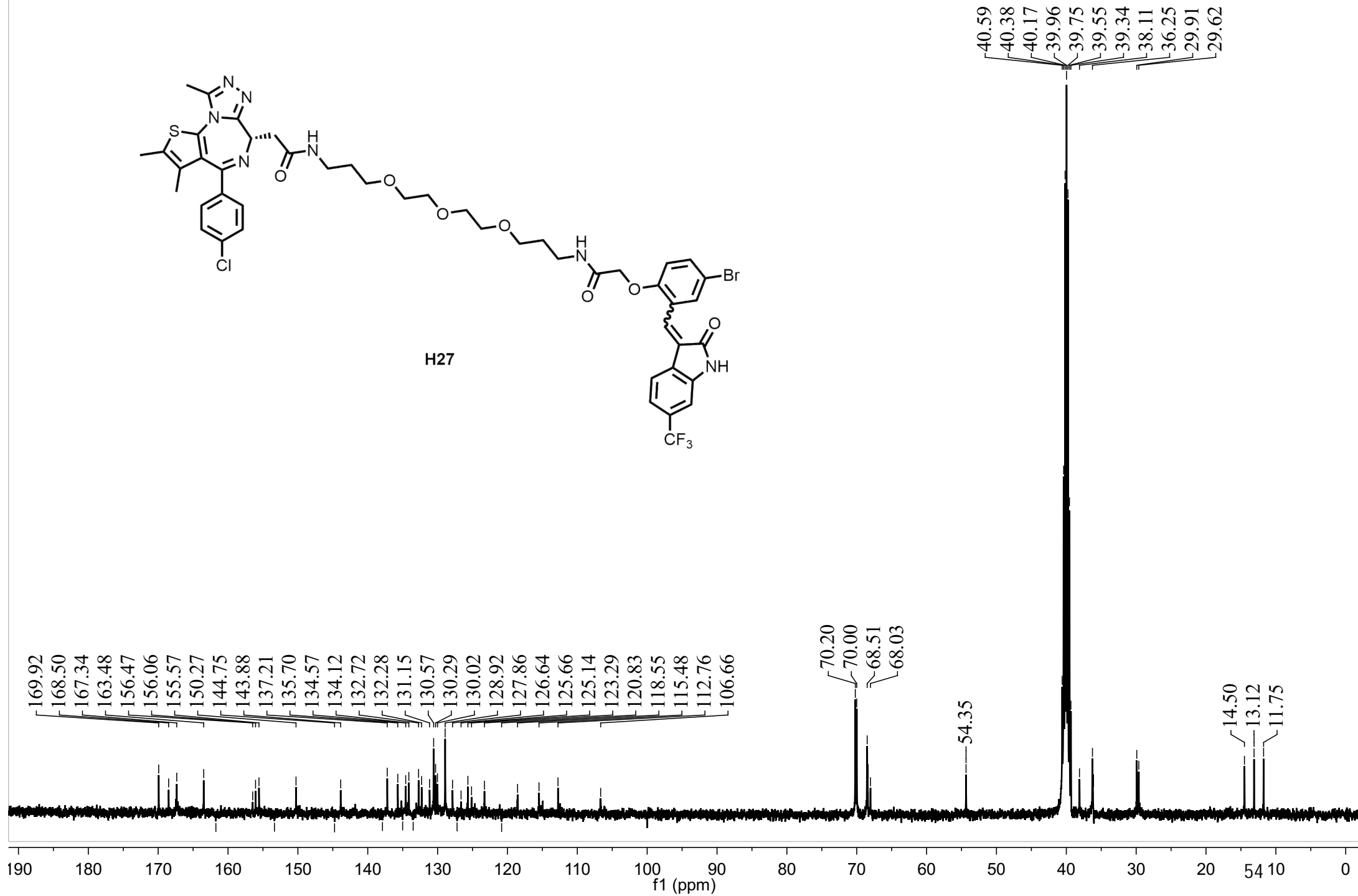

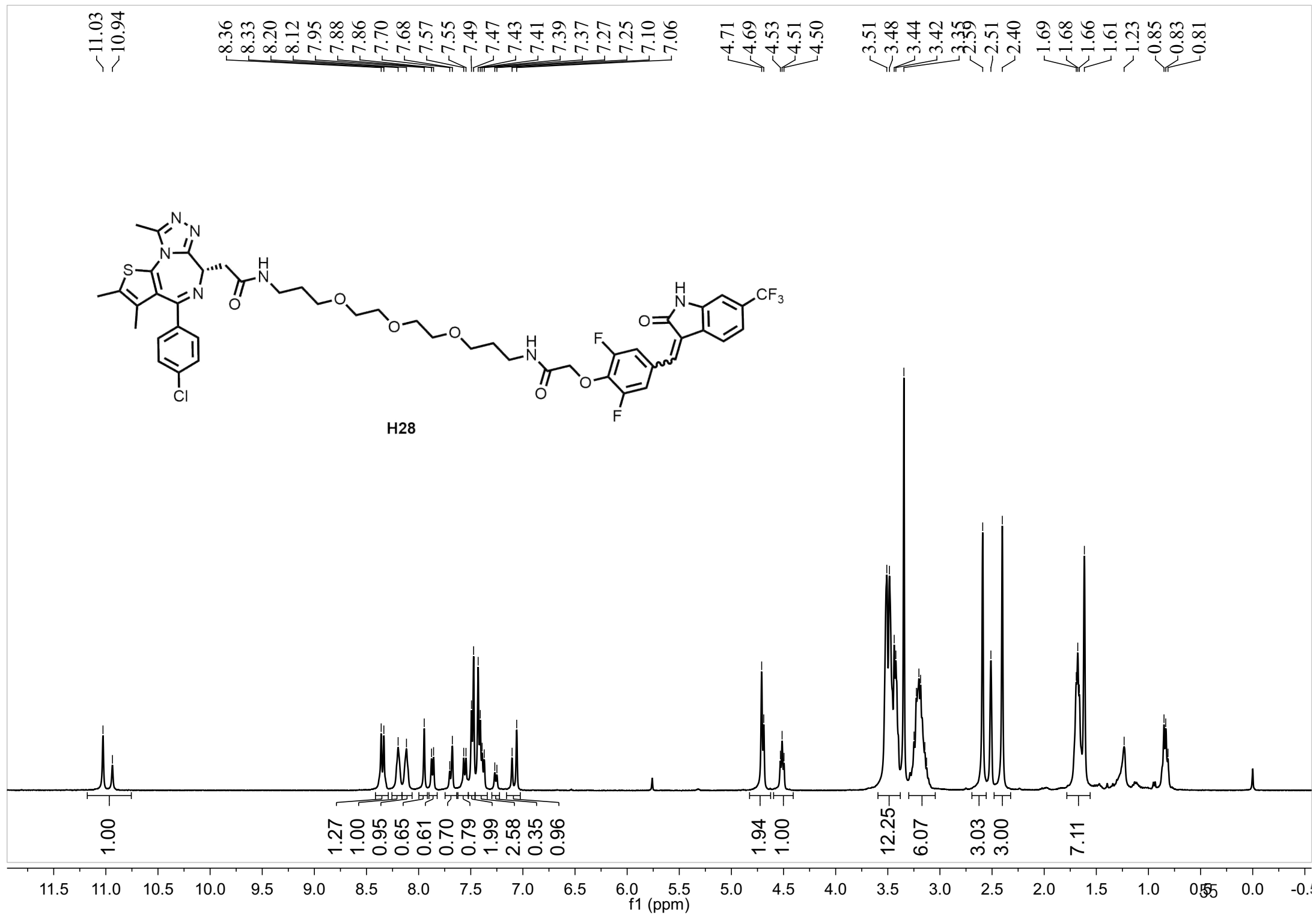

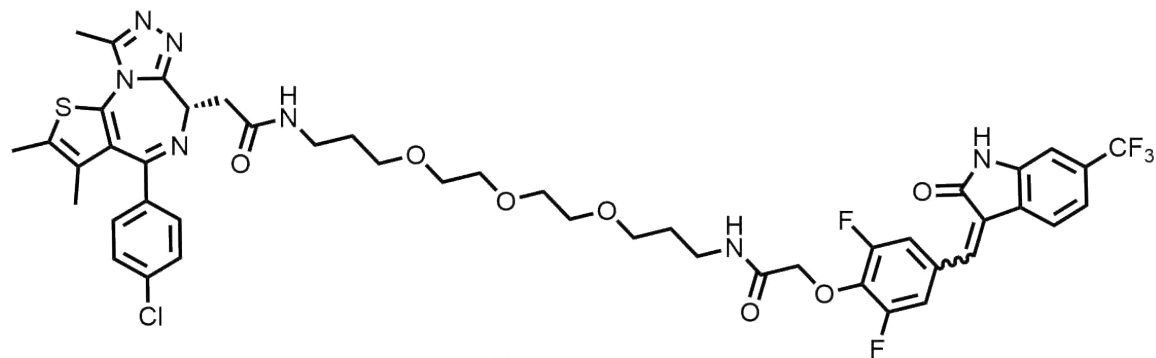

H28

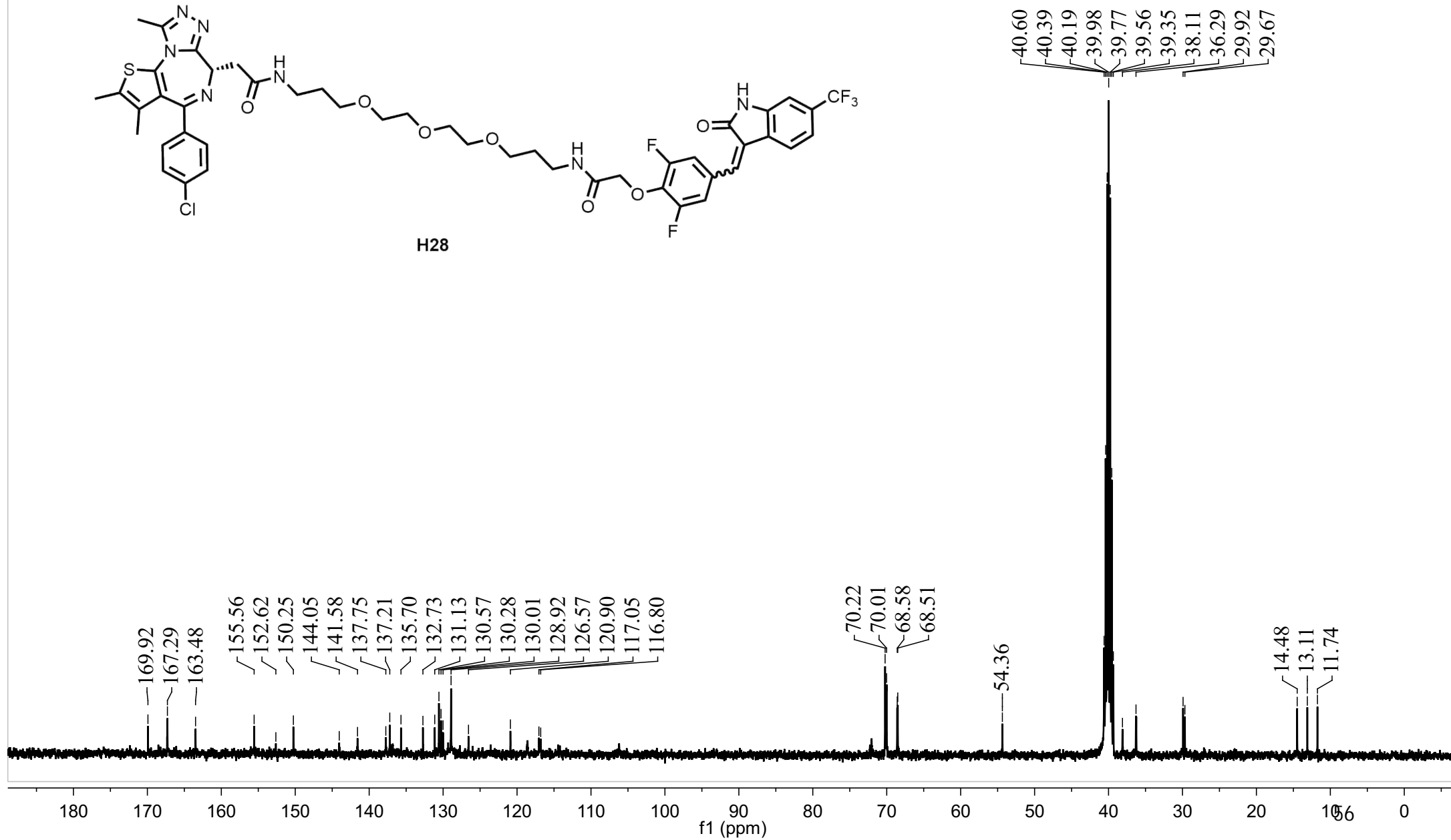

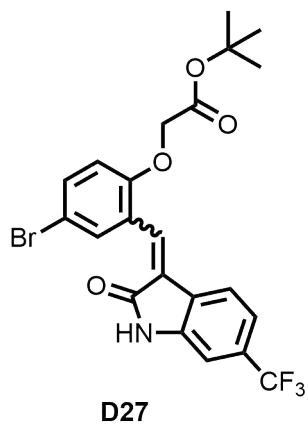

—10.92

7.83  
7.82  
7.75  
7.67  
7.67  
7.65  
7.64  
7.47  
7.45  
7.26  
7.24  
7.11  
7.09  
7.07

—4.81

—1.38

1.01

1.00  
0.96  
1.11  
1.01  
1.07  
2.13

2.18

9.06

12.0 11.5 11.0 10.5 10.0 9.5 9.0 8.5 8.0 7.5 7.0 6.5 6.0 5.5 5.0 4.5 4.0 3.5 3.0 2.5 2.0 1.5 1.0 0.5 0.0 -0.5

f1 (ppm)

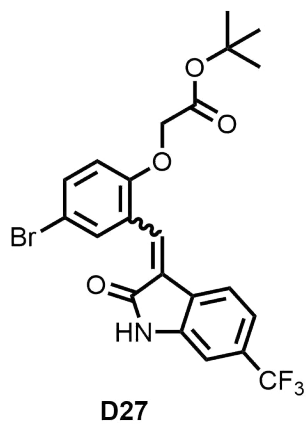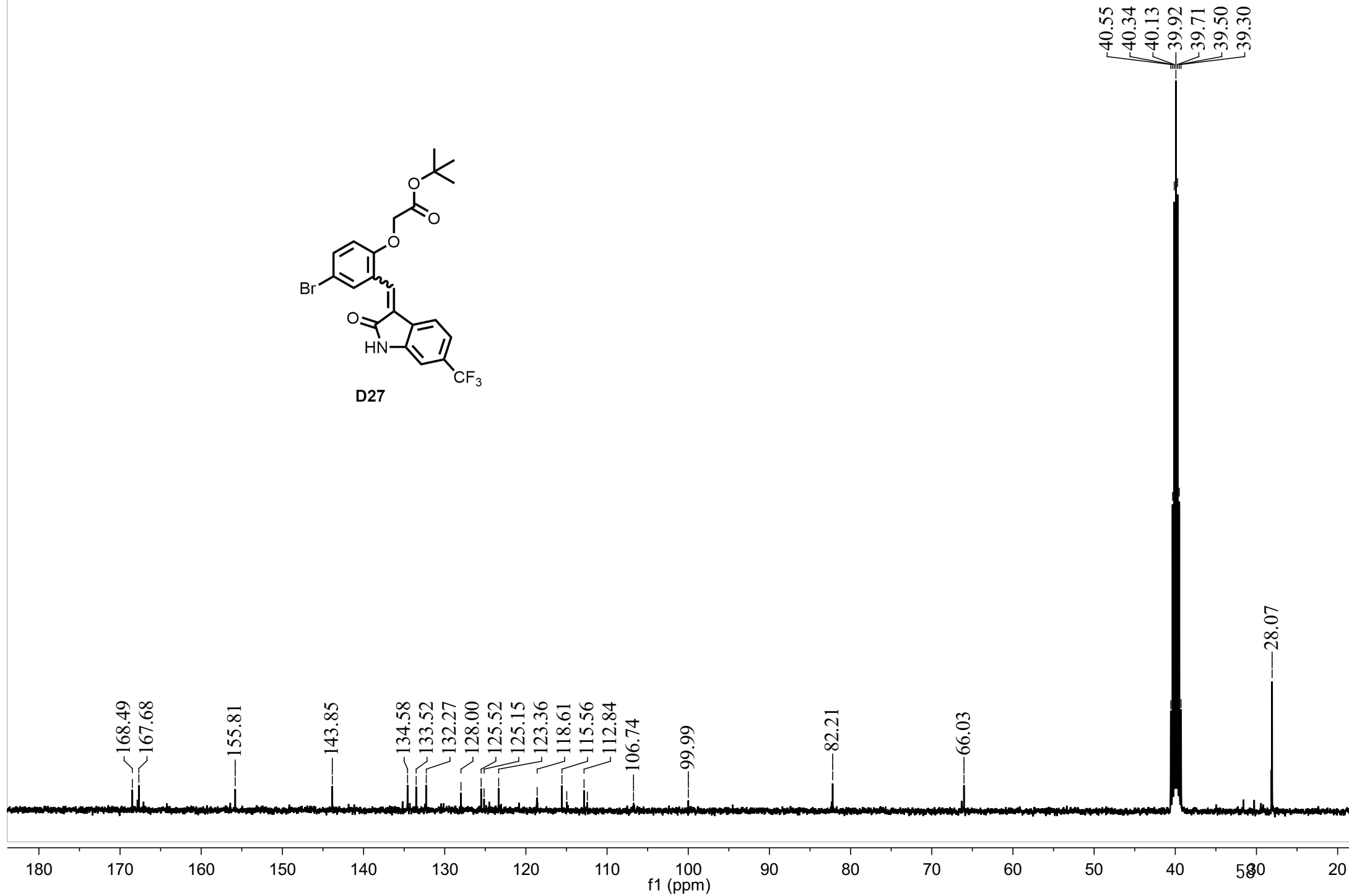

Supplement: S2 Data — (PDF) [file pbio.3002550.s010.pdf]
