# Supplementary material for: Alkenyl oxindole is a novel PROTAC moiety that recruits the CRL4DCAF11 E3 ubiquitin ligase complex for targeted protein degradation
Source: PLoS Biol. 2024 May 20;22(5):e3002550. doi: 10.1371/journal.pbio.3002550 (PMC11104598; doi:10.1371/journal.pbio.3002550)
Supplement: S1 Data — (PDF) [file pbio.3002550.s009.pdf]

The synthetic methods of compounds H1 - H28

Scheme I

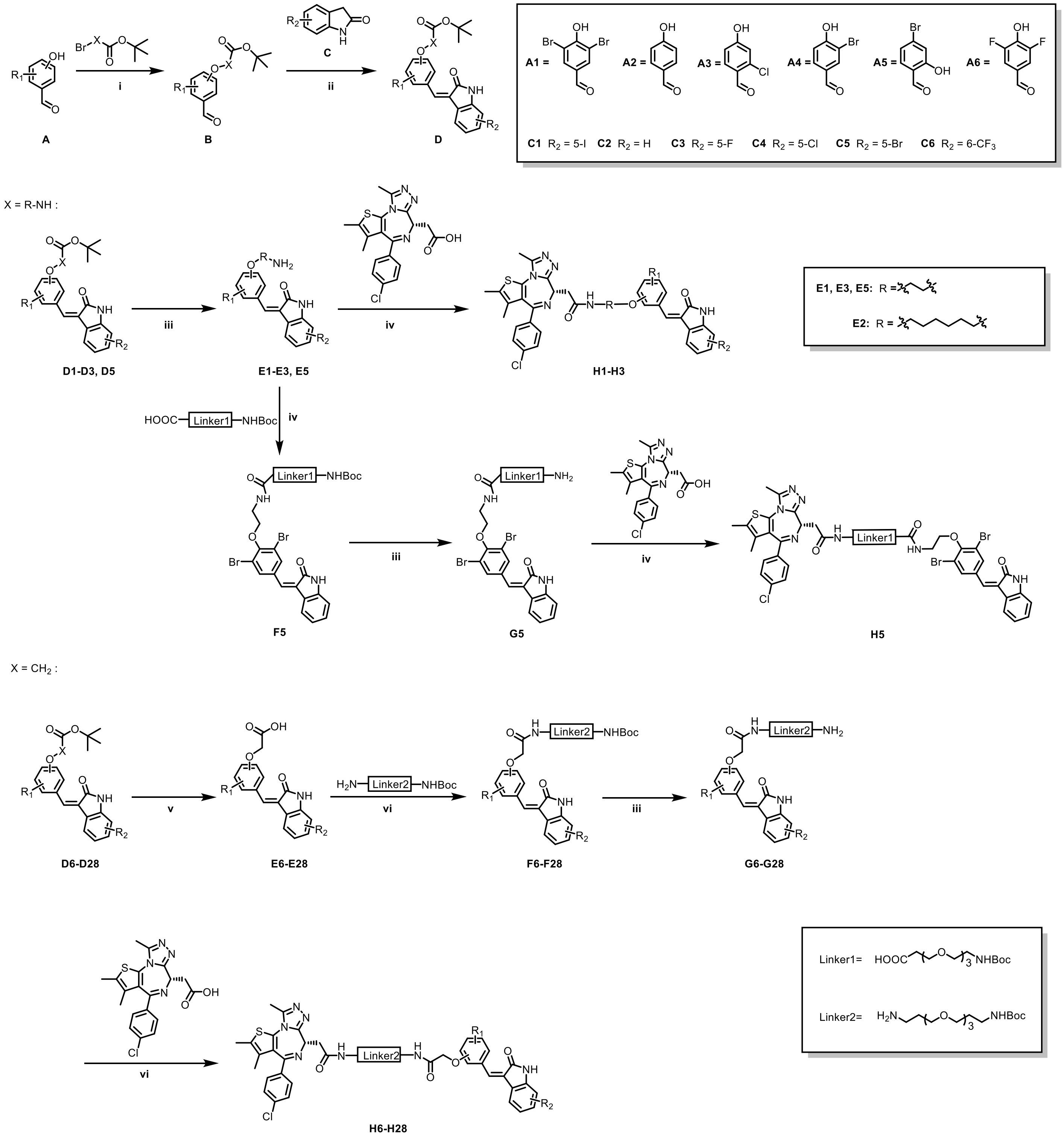

Scheme I: i) Cs_2CO_3, KI, Acetonitrile, 80°C reflux; ii) Piperidine, CH_3OH, 65°C reflux; iii) HCl, EA, rt; iv) HBTU, DIPEA, DMF, rt; v) TFA, DCM, rt; vi) HOBT, EDCI, DIPEA, DMF, rt.

(*S, Z*)-2-(4-(4-chlorophenyl)-2,3,9-trimethyl-6*H*-thieno[3,2-*f*][1,2,4]triazolo[4,3-*a*][1,4]diazepin-6-yl)-*N*-(2-(2,6-dibromo-4-((5-iodo-2-oxoindolin-3-ylidene)methyl)phenoxy)ethyl)acetamide (H1)

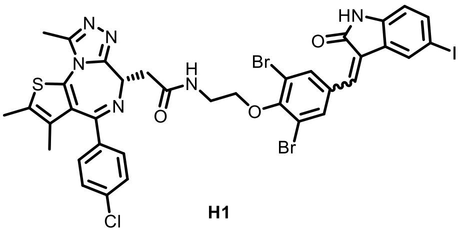

H1

The tert-butyl(2-bromoethyl)carbamate (2.91 g, 13 mmol) was added into a round-bottom flask filled with 3,5-dibromo-4-hydroxybenzaldehyde (**A1**, 2.80 g, 10 mmol), cesium carbonate (4.89 g, 15 mmol), potassium iodide (0.42 g, 2.5 mmol), acetonitrile (70 mL) and magnetic stirrer. The mixture refluxed at 80°C under argon atmosphere until the 3,5-dibromo-4-hydroxybenzaldehyde was consumed completely (monitored by TLC). Then, the mixture was extracted with methylene chloride (3 x 50 mL), and the combined organic layers were washed with saturated NaCl solution and dried with anhydrous Na_2SO_4. Subsequently, the solvent was evaporated under vacuum and the residue was purified by flash column chromatography on silica gel to obtain the product **B1** (white solid 0.79 g, 18.8% yield). The 5-iodolindolin-2-one (**C1**, 0.26 g, 1.0 mmol) was added into the solution of **B1** (0.46 g, 1.1mmol), piperidine (15 μL) and CH_3OH (10 mL), followed by refluxing at 65°C for 13 h. Then, the reaction product was purified by flash column chromatography to obtain the product **D1** (yellow solid 0.72 g, 77.32% yield).

5 mL HCl was added in 0.50 g **D1** (0.75 mmol) dissolved in 20 ml ethyl acetate and reacted at rt for 6 h. The mixture was extracted with ethyl acetate and ammonia, and the organic layers was purified to obtain the product **E1** (orange solid 0.41 g, 96% yield). Next, the mixture of JQ1 (carboxylic acid form, 0.4 g, 1.0 mmol), **E1** (0.41 g, 1.2 mmol), HBTU (0.56 g, 2.0 mmol) and DIPEA (0.6 mL, 4.0 mmol) were allowed to react in anhydrous DMF (3 mL) at room temperature for 3 h. After completion of the reaction, the reaction solution was extracted with ethyl acetate and the combine organic layers were dehydrated with anhydrous Na_2SO_4 and the solvent evaporated. The product was purified by flash column chromatography to afford **H1** (yellow solid, 0.50 g, 53.0% yield). **^{1}H NMR** (400 MHz, DMSO-d_6) δ 10.79 (d, J = 19.8 Hz, 1H), 8.79 (s, 1H), 8.53 (d, J =

5.9 Hz, 1H), 8.02 (d, J = 10.3 Hz, 1H), 7.84 (s, 1H), 7.70 (s, 1H), 7.62 – 7.50 (m, 2H), 7.44 (s, 4H), 6.71 (dd, J = 19.8, 8.1 Hz, 1H), 4.56 (t, J = 6.4 Hz, 1H), 4.10 (d, J = 5.0 Hz, 2H), 3.75 – 3.54 (m, 4H), 2.61 (d, J = 10.8 Hz, 3H), 2.40 (s, 3H), 1.62 (s, 3H). ^{13}**C NMR** (101 MHz, DMSO-d_6) δ 170.43, 166.97, 163.52, 155.57, 154.18, 150.32, 143.21, 140.97, 139.04, 137.98, 137.20, 136.67, 135.69, 135.00, 134.17, 133.97, 133.69, 133.36, 132.70, 131.20, 130.65, 130.34, 130.06, 128.88, 128.49, 127.57, 127.30, 123.54, 118.41, 117.75, 113.19, 112.46, 84.68, 72.45, 54.22, 38.03, 14.53, 13.16, 11.77. **ESI-MS**: m/z [M + H]$^+$ calcd for $C_{36}H_{29}O_3N_6ClBr_2IS^+$, 946.9088; found, 946.9087; purity: 95.2%.

(*S, Z*)-2-(4-(4-chlorophenyl)-2,3,9-trimethyl-6*H*-thieno[3,2-*f*][1,2,4]triazolo[4,3-*a*][1,4]diazepin-6-yl)-*N*-(6-(2,6-dibromo-4-((5-iodo-2-oxoindolin-3-ylidene)methyl)phenoxy)hexyl)acetamide (H2)

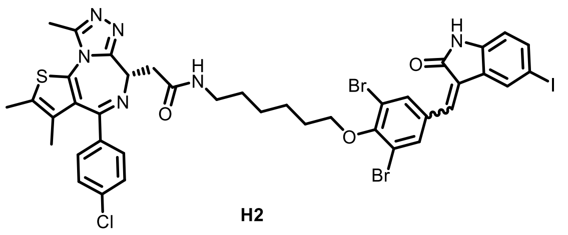

H2

The title compound **H2** (yellow solid, 35.2% yield) was synthesized according to the procedures for the preparation of **H1** from **A1** (3,5-dibromo-4-hydroxybenzaldehyde), **C1** (5-iodoindolin-2-one) and tert-butyl (6-bromohexyl) carbamate. ^{1}**H NMR** (400 MHz, DMSO-d_6) δ 10.83 (s, 1H), 8.79 (s, 1H), 8.22 (s, 1H), 8.06 (s, 1H), 8.00 (s, 1H), 7.85 (s, 1H), 7.58 (t, J = 8.7 Hz, 2H), 7.47 (dd, J = 18.1, 8.4 Hz, 5H), 6.75 (dd, J = 20.6, 8.2 Hz, 1H), 4.54 (s, 1H), 4.04 (dd, J = 11.5, 5.8 Hz, 3H), 3.37 – 2.98 (m, 5H), 2.61 (s, 3H), 2.42 (s, 3H), 1.84 (d, J = 6.8 Hz, 3H), 1.64 (s, 3H), 1.54 (d, J = 6.7 Hz, 5H), 1.44 (s, 3H). ^{13}**C NMR** (101 MHz, DMSO-d_6) δ 169.93, 166.98, 163.51, 155.59, 154.56, 154.04, 150.32, 149.29, 148.86, 143.19, 140.93, 137.19, 136.66, 135.74, 135.08, 134.24, 133.94, 133.44, 133.14, 132.69, 131.22, 130.56, 130.30, 128.91, 127.58, 127.17, 117.81, 112.46, 99.99, 84.67, 73.99, 54.39, 38.92, 38.16, 31.61, 29.96, 29.44, 26.64, 25.63, 14.53, 13.14, 11.74. **ESI-MS**: m/z [M + H]$^+$ calcd for $C_{40}H_{37}O_3N_6ClBr_2IS^+$, 1002.9722; found, 1002.9720; purity: 97%.

(*S, Z*)-2-(4-(4-chlorophenyl)-2,3,9-trimethyl-6*H*-thieno[3,2-*f*][1,2,4]triazolo[4,3-*a*][1,4]diazepin-6-yl)-*N*-(2-(2,6-dibromo-4-((2-oxoindolin-3-ylidene)methyl)phenoxy)ethyl)acetamide (H3)

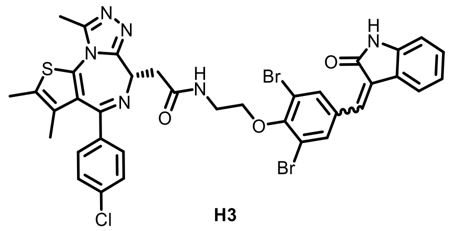

H3

The title compound **H3** (yellow solid, 54.7% yield) was synthesized according to the procedures for the preparation of **H1** from **A1** (3,5-dibromo-4-hydroxybenzaldehyde), **C2** (indolin-2-one) and tert-butyl(2-bromoethyl) carbamate. **^{1}H NMR** (400 MHz, DMSO-d_6) δ 10.68 (d, J = 24.4 Hz, 1H), 8.80 (s, 1H), 8.53 (s, 1H), 8.00 (s, 1H), 7.84 – 7.60 (m, 2H), 7.49 (d, J = 28.7 Hz, 5H), 7.24 (s, 1H), 7.15 – 6.77 (m, 2H), 4.56 (s, 1H), 4.09 (s, 2H), 3.60 (s, 3H), 2.94 (d, J = 17.6 Hz, 1H), 2.60 (s, 3H), 2.41 (s, 3H), 1.63 (s, 3H). **^{13}C NMR** (101 MHz, DMSO-d_6) δ 170.48, 167.55, 167.23, 163.56, 155.57, 153.84, 153.46, 150.37, 137.19, 136.95, 136.39, 135.69, 133.80, 133.32, 132.67, 131.86, 131.25, 130.65, 130.34, 130.07, 128.87, 124.91, 122.71, 121.82, 121.63, 121.47, 120.90, 120.76, 120.57, 118.39, 117.66, 114.71, 110.11, 100.00, 72.37, 54.21, 14.51, 13.14, 11.74. **ESI-MS**: m/z [M + H]$^+$ calcd for $C_{36}H_{30}O_3N_6ClBr_2S^+$, 821.0131; found, 821.0116; purity: > 99%.

Scheme II

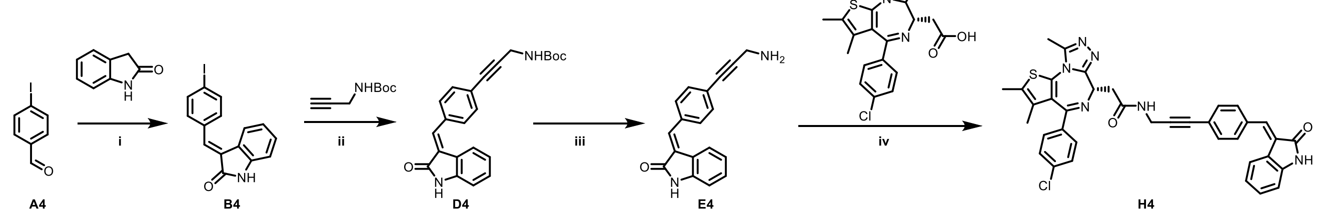

Scheme II: i) Piperidine, CH_3OH, 65°C reflux; ii) $PdCl_2(Pph)_3$, CuI, Et_3N, 50°C, Argon; iii) HCl, EA, rt; iv) HBTU, DIPEA, DMF, rt.

(*S, E*)-2-(4-(4-chlorophenyl)-2,3,9-trimethyl-6*H*-thieno[3,2-*f*][1,2,4]triazolo[4,3-*a*][1,4]diazepin-6-yl)-*N*-(3-(4-((2-oxoindolin-3-ylidene)methyl)phenyl)prop-2-yn-1-yl)acetamide (H4)

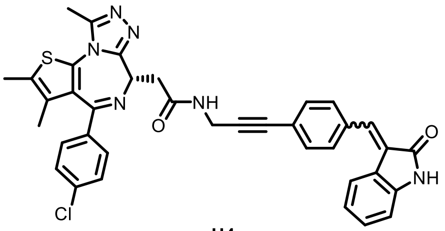

H4

The indolin-2-one (0.21 g, 1.6 mmol) was added into a round-bottom flask filled with 4-iodobenzaldehyde (**A4**, 0.46 g, 2 mmol), pyridine (34.06 mg, 0.4 mmol), CH_3OH (8 mL) and magnetic stirrer. The mixture refluxed at 65°C until the indolin-2-one was consumed completely. After solvent evaporated, the residue was purified by flash column chromatography to afford **B4** (0.49 g, 87.5% yield). Then, the mixture of **B4** (0.49 g, 1.4 mmol), tert-butyl prop-2-yn-1-ylcarbamate (0.33 g, 2.1 mmol), $PdCl_2(Pph)_3$ (24.74 mg, 0.036 mmol), CuI (13.42 mg, 0.007 mmol) and Et_3N (3 mL) were allowed to react at 50°C under argon atmosphere to afford the product **D4** (0.48 g, 91.6% yield). After that, 5 mL HCl was added into 0.48 g **D4** (1.28 mmol) dissolved in 20 mL ethyl acetate and reacted at rt for 6 h. The mixture was extracted with ethyl acetate and ammonia, and the organic layers was purified to obtain the product **E4** (0.31 g, 88.9% yield). Next, the mixture of JQ1 (carboxylic acid form, 0.4 g, 1.0 mmol), **E4** (0.31 g, 1.14 mmol), HBTU (0.56 g, 2.0 mmol) and DIPEA (0.6 mL, 4.0 mmol) were allowed to react in anhydrous DMF (3 mL) at room temperature for 3 h. After completion of the reaction, the reaction solution was extracted with ethyl acetate and the combine organic layers were dehydrated with anhydrous Na_2SO_4 and the solvent evaporated. The product was purified by flash column chromatography to afford **H4** (yellow solid, 0.32 g, 48.3% yield). **^{1}H NMR** (400 MHz, DMSO-d_6) δ 10.66 (d, J = 12.4 Hz, 1H), 8.85 (s, 1H), 8.42 (d, J = 8.1 Hz, 1H), 7.73 (d, J = 7.9 Hz, 2H), 7.62 (s, 1H), 7.58 (d, J = 8.0 Hz, 1H), 7.54 – 7.47 (m, 1H), 7.43 (d, J = 8.3 Hz, 2H), 7.34 (d, J = 8.3 Hz, 2H), 7.24 (dd, J = 14.2, 7.0 Hz, 1H), 6.89 (d, J = 7.8 Hz, 1H), 6.84 (t, J = 7.3 Hz, 1H), 4.63 – 4.42 (m, 1H), 4.26 (ddd, J = 22.2, 17.7, 5.2 Hz, 2H), 3.28 (d, J = 5.3 Hz, 2H), 2.61 (s, 3H), 2.41 (s, 3H), 1.61 (s, 3H). **^{13}C NMR** (101 MHz, DMSO-d_6) δ 170.05, 169.02, 167.53, 163.67, 155.45, 150.46, 143.55, 141.39, 137.12, 135.95, 135.70, 135.17, 135.07, 134.53, 132.73, 132.53, 132.24, 131.65, 131.32, 130.89, 130.64, 130.32, 130.12, 130.03, 128.83, 128.61, 125.25, 123.90, 123.00, 121.67, 121.15, 120.48, 110.71, 109.91, 89.62, 81.76, 54.30, 37.88,

31.99, 29.88, 29.18, 14.49, 13.15, 11.76. **ESI-MS**: *m/z* $[M + H]^+$ calcd for $C_{37}H_{29}O_2N_6ClS^+$, 657.1834; found, 657.1835; purity: > 99%.

(*S*,*Z*)-3-(2-(2-(2-(2-(4-(4-chlorophenyl)-2,3,9-trimethyl-6*H*-thieno[3,2-*f*][1,2,4]triazolo[4,3-*a*][1,4] diazepin-6-yl)acetamido)ethoxy)ethoxy)ethoxy)-*N*-(2-(2,6-dibromo-4-((2-oxoindolin-3-ylidene)methyl) phenoxy)ethyl)propenamide (H5)

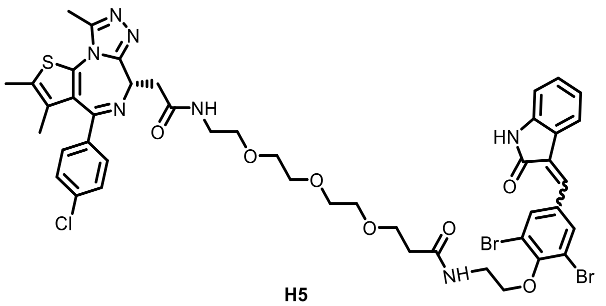

H5

The compound **E5** was synthesized through the same synthetic method of **E1**. The mixture of **E5** (0.44 g, 1.0 mmol), 2,2-dimethyl-4-oxo-3,8,11,14-tetraoxa-5-azaheptadecan-17-oic acid (Linker 1, 0.39 g, 1.2 mmol), HBTU (0.56 g, 2.0 mmol) and DIPEA (0.6 mL, 4.0 mmol) were reacted in anhydrous DMF (2 mL) at room temperature for 3 h. After completion of the reaction, the reaction solution was extracted with ethyl acetate and the combine organic layers were dehydrated with anhydrous Na_2SO_4 and the solvent evaporated. The product was purified by flash column chromatography to afford **F5** (0.31 g, 41.2% yield) and was dissolved in 10 mL ethyl acetate follow by adding 4 mL HCl to react at RT for 6 h. The mixture was extracted with ethyl acetate and ammonia, and the organic layers was purified to obtain the product **G5** (0.23 g, 86.3% yield). Then, JQ1 (carboxylic acid form, 0.12 g, 0.3 mmol), **G5** (0.23 g, 0.36 mmol), HBTU (0.19 g, 0.67 mmol) and DIPEA (0.2 mL, 1.33 mmol) were dissolved in 2 mL anhydrous DMF and reacted at room temperature for 3 h. After completion of the reaction, the reaction solution was extracted with ethyl acetate and the combine organic layers were dehydrated with anhydrous Na_2SO_4 and the solvent evaporated. The product was purified by flash column chromatography to afford **H5** (yellow solid, 0.12 g, 37.5% yield). **^{1}H NMR** (400 MHz, DMSO-*d*$_6$) δ 10.68 (d, *J* = 24.5 Hz, 1H), 8.75 (d, *J* = 24.8 Hz, 1H), 8.28 (s, 1H), 8.16 (s, 1H), 7.95 (d, *J* = 24.9 Hz, 1H), 7.80 – 7.61 (m, 2H), 7.45 (d, *J* = 13.8 Hz, 5H), 7.32 – 6.74 (m, 1H), 4.52 (s,

1H), 4.01 (d, J = 17.3 Hz, 4H), 3.54 (dd, J = 36.8, 26.6 Hz, 16H), 3.28 (s, 4H), 2.59 (s, 3H), 2.39 (t, J = 21.2 Hz, 6H), 1.61 (s, 3H). **^{13}C NMR** (101 MHz, DMSO-d_6) δ 170.94, 170.23, 163.51, 155.58, 137.22, 136.38, 135.70, 133.79, 132.71, 131.86, 131.20, 130.61, 130.31, 130.06, 128.91, 121.77, 118.34, 117.62, 72.41, 70.23, 70.09, 69.66, 67.19, 64.25, 54.29, 37.98, 36.54, 14.50, 13.13, 11.74. **ESI-MS**: m/z [M + H]$^+$ calcd for $C_{45}H_{47}O_7N_7ClBr_2S^+$, 1024.1078; found, 1024.1074; purity: > 99%.

(*S, Z*)-2-(4-(4-chlorophenyl)-2,3,9-trimethyl-6*H*-thieno[3,2-*f*][1,2,4]triazolo[4,3-*a*][1,4]diazepin-6-yl)-*N*-(1-(2,6-dibromo-4-((2-oxoindolin-3-ylidene)methyl)phenoxy)-2-oxo-7,10,13-trioxa-3-azahexadecan-16-yl)acetamide (H6)

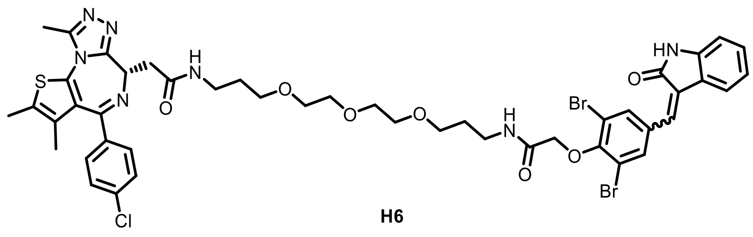

H6

The compound **D6** was synthesized through the same synthetic method of **D1**. Trifluoroacetic acid (TFA, 3 mL) was added to 3 mL of methylene chloride dissolved **D6** (0.51 g, 1.0 mmol) and stirred at room temperature. After full conversion of **D6**, the reaction mixture was concentrated and the product was purified by column chromatography to afford **E6** (0.42 g, 93.4% yield). Then, the mixture of **E6** (0.42 g, 0.93 mmol), tert-butyl (3-(2-(2-(3-aminopropoxy)ethoxy)ethoxy)propyl) carbamate (Linker 2, 0.36 g, 1.13 mmol), EDCI (0.27 g, 1.40 mmol), HOBT (0.40 g, 1.86 mmol) and DIPEA (0.45 mL, 2.80 mmol) in 10 mL of DMF was stirred at room temperature for 3 h. After completion of the reaction, the mixture was extracted with ethyl acetate and the combined organic layers were dried over Na_2SO_4. After the solvent was evaporated, the **F6** (0.29 g, 41.2% yield) was purified by flash column chromatography. The **F6** (0.29 g, 0.38 mmol) was dissolved in 10 mL ethyl acetate follow by adding 4 mL HCl to react at rt for 6 h. The mixture was extracted with ethyl acetate and ammonia, and the organic layer was purified to obtain the product **G6** (0.19 g, 77.6% yield). Then, the mixture of JQ1 (carboxylic acid form, 0.10 g, 0.25 mmol), **G6** (0.19 g, 0.30 mmol), EDCI (0.23 g, 1.17 mmol), HOBT (0.34 g, 1.57 mmol) and DIPEA (0.38 mL, 2.34 mmol) in 10 mL of DMF was stirred at room

temperature for 3 h. After completion of the reaction, the mixture was extracted with ethyl acetate and the combined organic layers were dried over Na_2SO_4. After the solvent was evaporated, the **F6** (0.10 g, 38.9% yield) was purified by flash column chromatography. **^{1}H NMR** (400 MHz, DMSO-*d*6) *δ* 10.69 (d, *J* = 26.1 Hz, 1H), 8.80 (s, 1H), 8.23 – 8.09 (m, 2H), 7.99 (s, 1H), 7.45 (td, *J* = 20.1, 11.9 Hz, 6H), 7.25 (dd, *J* = 13.8, 7.0 Hz, 1H), 6.87 (dd, *J* = 17.5, 8.9 Hz, 2H), 4.51 (d, *J* = 6.9 Hz, 1H), 4.45 (d, *J* = 10.9 Hz, 2H), 3.56 – 3.40 (m, 13H), 3.31 – 3.07 (m, 8H), 2.59 (s, 3H), 2.40 (s, 3H), 1.78 – 1.65 (m, 4H), 1.62 (s, 3H). **^{13}C NMR** (101 MHz, DMSO-*d*6) *δ* 169.96, 168.65, 168.57, 167.51, 166.69, 163.50, 155.57, 152.88, 152.53, 150.27, 143.82, 141.57, 137.22, 136.37, 135.71, 134.58, 133.94, 133.77, 133.12, 132.71, 132.46, 131.25, 131.17, 130.57, 130.31, 130.04, 128.93, 124.89, 121.79, 120.88, 120.59, 118.16, 117.41, 110.10, 71.45, 70.25, 70.10, 70.01, 68.89, 68.53, 54.36, 38.14, 36.59, 36.28, 29.92, 29.62, 14.50, 13.13, 11.75. **ESI-MS**: *m*/*z* [M + H]$^+$ calcd for $C_{46}H_{49}O_7N_7ClBr_2S^+$, 1038.1445; found, 1038.1439; purity: > 99%.

(*S, Z*)-2-(4-(4-chlorophenyl)-2,3,9-trimethyl-6*H*-thieno[3,2-*f*][1,2,4]triazolo[4,3-*a*][1,4]diazepin-6-yl)-*N*-(1-(2,6-dibromo-4-((5-iodo-2-oxoindolin-3-ylidene)methyl)phenoxy)-2-oxo-7,10,13-trioxa-3-azahexadecan-16-yl)acetamide (H7)

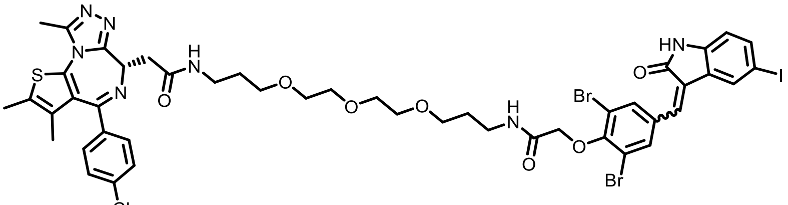

H7

The title compound **H7** (yellow solid, 31.2% yield) was synthesized according to the procedures for the preparation of **H6** from **A1**(3,5-dibromo-4-hydroxybenzaldehyde), **C1** (5-iodoindolin-2-one), tert-butyl 2-bromoacetate and Linker 2. **^{1}H NMR** (400 MHz, DMSO-*d*6) *δ* 10.81 (d, *J* = 23.8 Hz, 1H), 8.78 (s, 1H), 8.27 – 8.10 (m, 2H), 8.03 (d, *J* = 6.9 Hz, 1H), 7.85 (s, 1H), 7.61 – 7.37 (m, 6H), 6.72 (dd, *J* = 20.7, 8.0 Hz, 1H), 4.51 (t, *J* = 6.8 Hz, 1H), 4.45 (d, *J* = 9.6 Hz, 2H), 3.47 (dd, *J* = 16.7, 11.5 Hz, 15H), 3.31 – 3.09 (m, 6H), 2.59 (s, 3H), 2.39 (s, 3H), 1.78 – 1.65 (m, 4H), 1.60 (s, 3H). **^{13}C NMR** (101 MHz, DMSO-*d*6) *δ* 171.70, 169.96, 168.02, 166.95, 166.65, 163.50, 155.56, 153.23, 152.75, 150.27, 143.26, 141.02, 139.88, 139.12, 137.22,

136.62, 135.70, 134.82, 134.12, 133.95, 133.73, 132.71, 131.16, 130.57, 130.30, 130.02, 128.93, 128.67, 127.55, 123.50, 118.19, 117.50, 113.21, 112.48, 84.67, 84.36, 71.45, 70.25, 70.10, 70.01, 68.89, 68.53, 54.36, 38.13, 36.59, 36.28, 29.92, 29.62, 14.51, 13.14, 11.76. **ESI-MS**: *m/z* $[M + H]^+$ calcd for $C_{46}H_{48}O_7N_7ClBr_2IS^+$, 1164.0401; found, 1164.0399; purity: > 99%.

(*S, Z*)-2-(4-(4-chlorophenyl)-2,3,9-trimethyl-6*H*-thieno[3,2-*f*][1,2,4]triazolo[4,3-*a*][1,4]diazepin-6-yl)-*N*-(1-(2,6-dibromo-4-((5-fluoro-2-oxoindolin-3-ylidene)methyl)phenoxy)-2-oxo-7,10,13-trioxa-3-azahexadecan-16-yl)acetamide (H8)

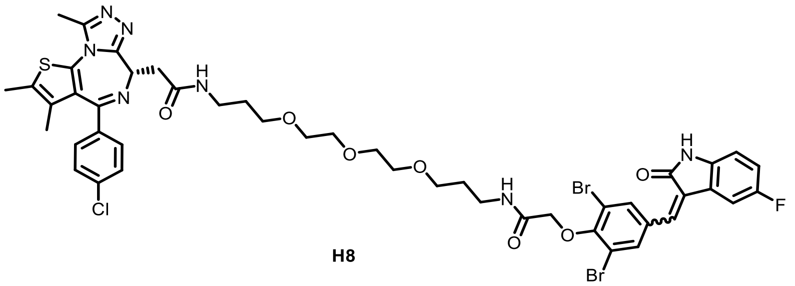

H8

The title compound **H8** (yellow solid, 24.6% yield) was synthesized according to the procedures for the preparation of **H6** from **A1** (3,5-dibromo-4-hydroxybenzaldehyde), **C3** (5-fluoroindolin-2-one), tert-butyl 2-bromoacetate and Linker 2. ^{1}**H NMR** (400 MHz, DMSO-d_6) δ 10.05 (s, 1/3H), 9.81 (s, 1/3H), 8.82 (s, 2/3H), 7.96 (s, 1H), 7.74 (d, J = 12.2 Hz, 5/3H), 7.60 (s, 1H), 7.50 (d, J = 8.1 Hz, 3H), 7.41 (d, J = 7.9 Hz, 2H), 7.21 (d, J = 8.9 Hz, 2/3H), 7.11 – 6.86 (m, 2H), 4.65 (s, 1H), 4.53 (d, J = 11.5 Hz, 2H), 3.61 (s, 6H), 3.55 (s, 6H), 3.48 (d, J = 6.0 Hz, 3H), 3.36 (d, J = 7.8 Hz, 4H), 2.62 (s, 3H), 2.44 (s, 3H), 1.87 (s, 2H), 1.79 (s, 2H), 1.69 (d, J = 4.5 Hz, 3H). ^{13}**C NMR** (101 MHz, DMSO-d_6) δ 169.90, 168.60, 167.50, 166.68, 166.62, 163.47, 156.64, 155.56, 152.77, 150.25, 137.21, 136.57, 135.70, 134.82, 134.14, 133.80, 133.65, 132.73, 131.14, 130.57, 130.29, 130.02, 129.49, 128.93, 118.28, 117.51, 71.44, 70.25, 70.10, 70.01, 68.87, 68.52, 54.36, 38.12, 36.56, 36.25, 29.93, 29.64, 14.51, 13.13, 11.76. **ESI-MS**: *m/z* $[M + H]^+$ calcd for $C_{46}H_{48}O_7N_7ClBr_2FS^+$, 1056.1349; found, 1056.1354; purity: > 99%.

(*S, Z*)-2-(4-(4-chlorophenyl)-2,3,9-trimethyl-6*H*-thieno[3,2-*f*][1,2,4]triazolo[4,3-*a*][1,4]diazepin-6-yl)-*N*-(1-(2,6-dibromo-4-((5-chloro-2-oxoindolin-3-ylidene)methyl)phenoxy)-2-oxo-7,10,13-trioxa-3-

azahexadecan-16-yl)acetamide (H9)

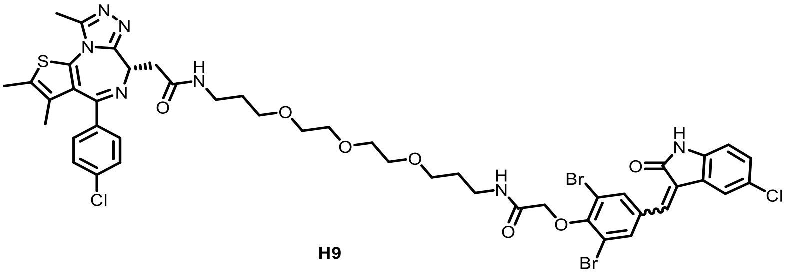

H9

The title compound **H9** (yellow solid, 33.2% yield) was synthesized according to the procedures for the preparation of **H6** from **A1** (3,5-dibromo-4-hydroxybenzaldehyde), **C4** (5-chloroindolin-2-one), tert-butyl 2-bromoacetate and Linker 2. **^{1}H NMR** (400 MHz, DMSO-d_6) δ 10.13 (s, 1/3H), 9.88 (s, 1/3H), 8.82 (s, 1H), 7.98 (s, 1H), 7.80 (s, 2/3H), 7.72 (s, 5/3H), 7.59 (d, J = 10.1 Hz, 1H), 7.49 (t, J = 9.2 Hz, 3H), 7.41 (d, J = 8.1 Hz, 2H), 7.26 (dd, J = 16.5, 8.2 Hz, 1H), 7.08 – 6.86 (m, 1H), 4.66 (d, J = 6.5 Hz, 1H), 4.53 (d, J = 11.2 Hz, 2H), 3.61 (s, 6H), 3.56 (d, J = 8.3 Hz, 6H), 3.48 (d, J = 5.9 Hz, 3H), 3.42 – 3.25 (m, 4H), 2.62 (s, 3H), 2.44 (s, 3H), 1.87 (d, J = 3.5 Hz, 2H), 1.79 (d, J = 4.1 Hz, 2H), 1.69 (d, J = 6.9 Hz, 3H). **^{13}C NMR** (101 MHz, DMSO-d_6) δ 169.94, 167.24, 166.62, 163.48, 159.78, 155.55, 153.23, 152.14, 150.23, 149.91, 140.21, 138.59, 136.62, 135.70, 135.03, 133.90, 133.66, 133.21, 132.72, 131.13, 130.56, 130.29, 130.00, 128.92, 127.86, 126.07, 125.56, 120.59, 118.25, 117.51, 113.97, 111.48, 108.58, 100.00, 71.43, 70.25, 70.11, 70.01, 68.88, 68.53, 54.36, 38.13, 36.57, 36.27, 29.93, 29.64, 14.50, 13.12, 11.76. **ESI-MS**: *m/z* $[M + H]^+$ calcd for $C_{46}H_{48}O_7N_7Cl_2Br_2S^+$, 1072.1054; found, 1072.1058; purity: > 99%.

(*S, Z*)-2-(4-(4-chlorophenyl)-2,3,9-trimethyl-6*H*-thieno[3,2-*f*][1,2,4]triazolo[4,3-*a*][1,4]diazepin-6-yl)-*N*-(1-(2,6-dibromo-4-((5-bromo-2-oxoindolin-3-ylidene)methyl)phenoxy)-2-oxo-7,10,13-trioxa-3-azahexadecan-16-yl)acetamide (H10)

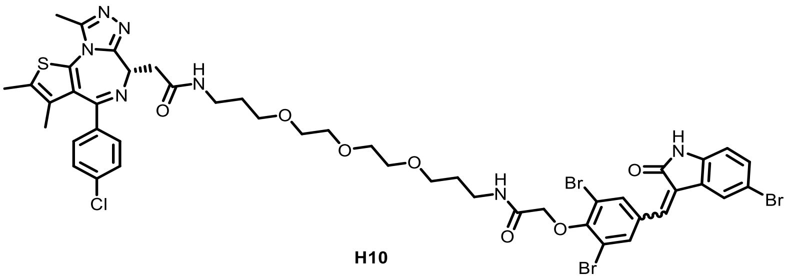

H10

The title compound **H10** (yellow solid, 56.8% yield) was synthesized according to the procedures for the preparation of **H6** from **A1** (3,5-dibromo-4-hydroxybenzaldehyde), **C5** (5-bromoindolin-2-one), tert-butyl 2-bromoacetate and Linker 2. **^{1}H NMR** (400 MHz, DMSO-d_6) δ 10.13 (s, 1/3H), 9.88 (s, 1/3H), 8.82 (s, 1H), 7.98 (s, 1H), 7.80 (s, 2/3H), 7.72 (s, 5/3H), 7.59 (d, J = 10.1 Hz, 1H), 7.49 (t, J = 9.2 Hz, 3H), 7.41 (d, J = 8.1 Hz, 2H), 7.26 (dd, J = 16.5, 8.2 Hz, 1H), 7.08 – 6.86 (m, 1H), 4.66 (d, J = 6.5 Hz, 1H), 4.53 (d, J = 11.2 Hz, 2H), 3.61 (s, 6H), 3.56 (d, J = 8.3 Hz, 6H), 3.48 (d, J = 5.9 Hz, 3H), 3.42 – 3.25 (m, 4H), 2.62 (s, 3H), 2.44 (s, 3H), 1.87 (d, J = 3.5 Hz, 2H), 1.79 (d, J = 4.1 Hz, 2H), 1.69 (d, J = 6.9 Hz, 3H). **^{13}C NMR** (101 MHz, DMSO-d_6) δ 169.93, 169.77, 168.20, 167.12, 166.61, 163.48, 155.55, 153.26, 150.24, 142.89, 140.58, 138.34, 137.21, 136.65, 135.70, 135.07, 134.29, 134.07, 133.93, 133.39, 132.73, 132.24, 131.13, 130.57, 130.29, 130.00, 128.93, 127.72, 127.22, 126.03, 125.32, 123.37, 123.03, 118.25, 117.81, 117.52, 113.72, 113.17, 112.71, 111.99, 71.44, 70.26, 70.11, 70.02, 68.88, 68.53, 54.36, 38.14, 36.57, 36.27, 29.94, 29.64, 14.52, 13.14, 11.77. **ESI-MS**: m/z [M + H]$^+$ calcd for $C_{46}H_{48}O_7N_7ClBr_3S^+$, 1118.0528; found, 1118.0527; purity: > 99%.

(*S, Z*)-2-(4-(4-chlorophenyl)-2,3,9-trimethyl-6*H*-thieno[3,2-*f*][1,2,4]triazolo[4,3-*a*][1,4]diazepin-6-yl)-*N*-(1-(2,6-dibromo-4-((2-oxo-6-(trifluoromethyl)indolin-3-ylidene)methyl)phenoxy)-2-oxo-7,10,13-trioxa-3-azahexadecan-16-yl)acetamide (H11)

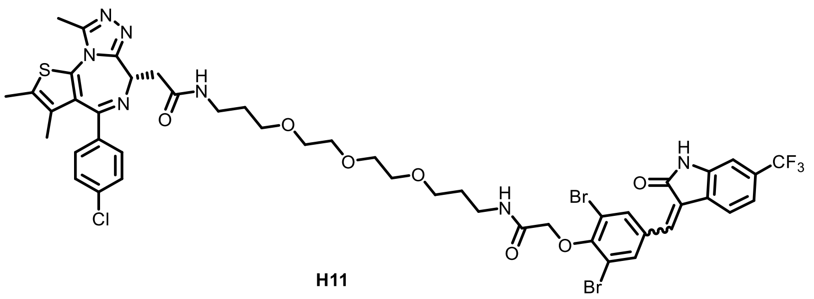

H11

The title compound **H11** (yellow solid, 39.7% yield) was synthesized according to the procedures for the preparation of **H6** from **A1** (3,5-dibromo-4-hydroxybenzaldehyde), **C6** (6-(trifluoromethyl)indolin-2-one), tert-butyl 2-bromoacetate and Linker 2. **^{1}H NMR** (400 MHz, DMSO-d_6) δ 10.99 (d, J = 25.6 Hz, 1H), 8.82 (s, 1H), 8.18 (d, J = 16.4 Hz, 2H), 8.00 (d, J = 24.9 Hz, 1H), 7.87 (d, J = 7.8 Hz, 2/3H), 7.59 – 7.32 (m, 5H), 7.25 (d, J = 7.5 Hz, 1/3H), 7.08 (d, J = 20.1 Hz, 1H), 4.63 – 4.37 (m, 3H), 3.69 – 3.40 (m, 13H), 3.32 – 3.08 (m,

6H), 2.59 (s, 3H), 2.40 (s, 3H), 1.72 (dd, J = 18.4, 6.2 Hz, 4H), 1.61 (s, 3H). **^{13}C NMR** (101 MHz, DMSO-d_6) δ 169.93, 167.19, 166.60, 163.48, 155.55, 153.49, 150.24, 141.79, 137.21, 136.84, 136.48, 135.70, 133.93, 133.47, 132.73, 131.12, 130.57, 130.28, 130.00, 128.92, 127.42, 121.11, 118.64, 118.33, 117.54, 106.25, 71.45, 70.25, 70.10, 70.01, 68.87, 68.52, 54.36, 38.12, 36.56, 36.26, 29.93, 29.63, 14.49, 13.12, 11.75. **ESI-MS**: *m/z* [M + H]$^+$ calcd for $C_{47}H_{48}O_7N_7ClBr_2F_3S^+$, 1106.1328; found, 1106.1326; purity: > 99%.

(*S, Z*)-2-(4-(4-chlorophenyl)-2,3,9-trimethyl-6*H*-thieno[3,2-*f*][1,2,4]triazolo[4,3-*a*][1,4]diazepin-6-yl)-*N*-(1-(4-((5-fluoro-2-oxoindolin-3-ylidene)methyl)phenoxy)-2-oxo-7,10,13-trioxa-3-azahexadecan-16-yl)acetamide (H12)

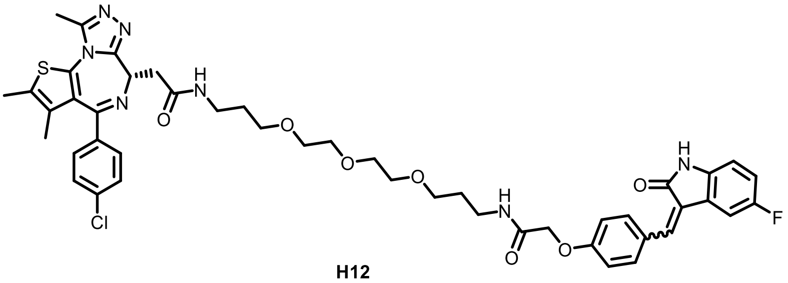

H12

The title compound **H12** (yellow solid, 29.4% yield) was synthesized according to the procedures for the preparation of **H6** from **A2** (4-hydroxybenzaldehyde), **C3** (5-fluoroindolin-2-one), tert-butyl 2-bromoacetate and Linker 2. **^{1}H NMR** (400 MHz, DMSO-d_6) δ 10.60 (s, 1H), 8.48 (d, J = 8.3 Hz, 1/3H), 8.16 (d, J = 22.8 Hz, 2H), 7.83 (s, 1/3H), 7.71 (d, J = 8.1 Hz, 4/3H), 7.65 (s, 2/3H), 7.61 (d, J = 8.5 Hz, 1/3H), 7.48 (d, J = 8.2 Hz, 2H), 7.42 (d, J = 8.0 Hz, 2H), 7.34 (d, J = 9.4 Hz, 2/3H), 7.17 – 6.95 (m, 3H), 6.90 – 6.75 (m, 1H), 4.58 (s, 2H), 4.52 (s, 1H), 3.59 – 3.38 (m, 13H), 3.29 – 3.10 (m, 6H), 2.59 (s, 3H), 2.40 (s, 3H), 1.73 – 1.64 (m, 4H), 1.61 (s, 3H). **^{13}C NMR** (101 MHz, DMSO-d_6) δ 169.93, 169.31, 167.86, 167.62, 167.56, 163.49, 160.30, 159.67, 156.46, 155.57, 150.26, 139.50, 138.82, 137.99, 137.22, 136.97, 135.70, 135.03, 132.73, 131.95, 131.14, 130.57, 130.29, 130.02, 128.93, 127.81, 127.29, 126.09, 124.51, 122.49, 116.57, 116.34, 115.58, 114.99, 110.41, 109.70, 70.22, 70.02, 68.64, 68.51, 67.47, 54.36, 38.12, 36.34, 36.26, 29.92, 29.73, 14.50, 13.12, 11.75. **ESI-MS**: *m/z* [M + H]$^+$ calcd for $C_{46}H_{50}O_7N_7ClFS^+$, 898.3160; found, 898.3161; purity: > 99%.

(*S, Z*)-2-(4-((5-chloro-2-oxoindolin-3-ylidene)methyl)phenoxy)-*N*-(1-(4-(4-chlorophenyl)-2,3,9-trimethy

l-6*H*-thieno[3,2-*f*][1,2,4]triazolo[4,3-*a*][1,4]diazepin-6-yl)-2-oxo-7,10,13-trioxa-3-azahexadecan-16-yl)acetamide (H13)

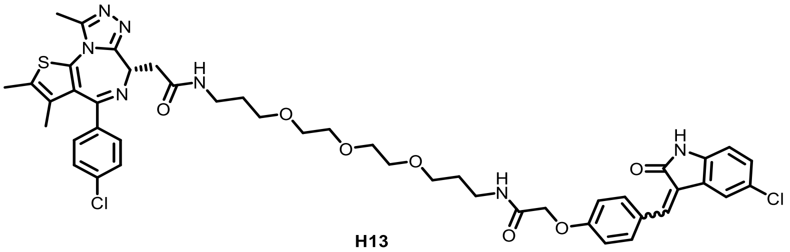

H13

The title compound **H13** (yellow solid, 38.5% yield) was synthesized according to the procedures for the preparation of **H6** from **A2** (4-hydroxybenzaldehyde), **C4** (5-chloroindolin-2-one), tert-butyl 2-bromoacetate and Linker 2. **^{1}H NMR** (400 MHz, DMSO-d_6) δ 10.72 (s, 1H), 8.49 (d, J = 8.1 Hz, 1H), 8.17 (d, J = 19.6 Hz, 2H), 7.85 (d, J = 31.6 Hz, 1H), 7.76 – 7.61 (m, 2H), 7.55 (s, 1H), 7.45 (dd, J = 24.1, 7.7 Hz, 4H), 7.24 (dd, J = 27.2, 8.0 Hz, 1H), 7.10 (dd, J = 26.6, 8.0 Hz, 2H), 6.86 (dd, J = 28.2, 8.3 Hz, 1H), 4.55 (d, J = 25.2 Hz, 3H), 3.65 – 3.38 (m, 12H), 3.21 (d, J = 6.8 Hz, 7H), 2.59 (s, 3H), 2.40 (s, 3H), 1.68 (s, 4H), 1.61 (s, 3H). **^{13}C NMR** (101 MHz, DMSO-d_6) δ 169.99, 168.94, 167.61, 163.49, 160.34, 159.72, 155.57, 150.80, 150.25, 141.93, 139.38, 138.22, 137.21, 135.70, 135.10, 132.72, 131.96, 131.14, 130.57, 130.29, 130.01, 129.71, 128.92, 127.28, 125.51, 125.37, 123.73, 123.23, 121.95, 119.83, 115.55, 115.01, 111.87, 111.01, 70.22, 70.02, 68.66, 68.51, 67.48, 54.36, 38.13, 36.35, 29.92, 29.73, 14.50, 13.12, 11.75. **ESI-MS**: *m/z* [M + H]$^+$ calcd for $C_{46}H_{50}O_7N_7Cl_2S^+$, 914.2864; found, 914.2867; purity: > 99%.

(*S*, *Z*)-2-(4-((5-bromo-2-oxoindolin-3-ylidene)methyl)phenoxy)-*N*-(1-(4-(4-chlorophenyl)-2,3,9-trimethyl-6*H*-thieno[3,2-*f*][1,2,4]triazolo[4,3-*a*][1,4]diazepin-6-yl)-2-oxo-7,10,13-trioxa-3-azahexadecan-16-yl)acetamide (H14)

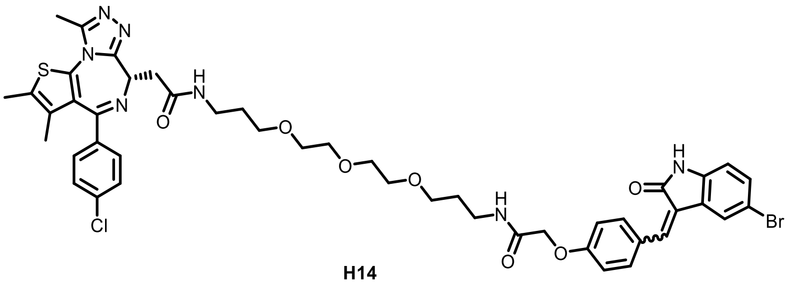

H14

The title compound **H14** (yellow solid, 50.0% yield) was synthesized according to the procedures for the preparation of **H6** from **A2** (4-hydroxybenzaldehyde), **C5** (5-bromoindolin-2-one), tert-butyl 2-bromoacetate and Linker 2. ^{1}**H NMR** (400 MHz, DMSO-d_6) δ 10.73 (s, 1H), 8.49 (s, 1H), 8.17 (d, J = 18.1 Hz, 2H), 7.91 (d, J = 16.4 Hz, 1H), 7.77 – 7.60 (m, 3H), 7.56 – 7.37 (m, 5H), 7.10 (d, J = 26.8 Hz, 2H), 6.91 – 6.72 (m, 1H), 4.59 (s, 2H), 4.53 (s, 1H), 3.57 – 3.39 (m, 13H), 3.22 (d, J = 5.1 Hz, 6H), 2.60 (d, J = 3.8 Hz, 3H), 2.40 (d, J = 3.2 Hz, 3H), 1.69 (s, 4H), 1.62 (s, 3H). ^{13}**C NMR** (101 MHz, DMSO-d_6) δ 169.93, 167.61, 163.49, 160.36, 159.75, 155.56, 150.24, 142.28, 139.09, 138.22, 137.22, 135.71, 135.12, 132.72, 132.49, 131.96, 131.13, 130.57, 130.30, 130.03, 128.93, 127.27, 125.41, 124.66, 122.57, 115.55, 115.01, 113.08, 112.37, 111.54, 70.24, 70.03, 68.66, 54.36, 38.12, 36.35, 29.94, 29.74, 14.51, 13.12, 11.75. **ESI-MS**: m/z [M + H]$^+$ calcd for $C_{46}H_{50}O_7N_7ClBrS^+$, 960.2340; found, 960.2340; purity: > 99%.

(*S, Z*)-2-(4-(4-chlorophenyl)-2,3,9-trimethyl-6*H*-thieno[3,2-*f*][1,2,4]triazolo[4,3-*a*][1,4]diazepin-6-yl)-*N*-(2-oxo-1-(4-((2-oxo-6-(trifluoromethyl)indolin-3-ylidene)methyl)phenoxy)-7,10,13-trioxa-3-azahexadecan-16-yl)acetamide (H15)

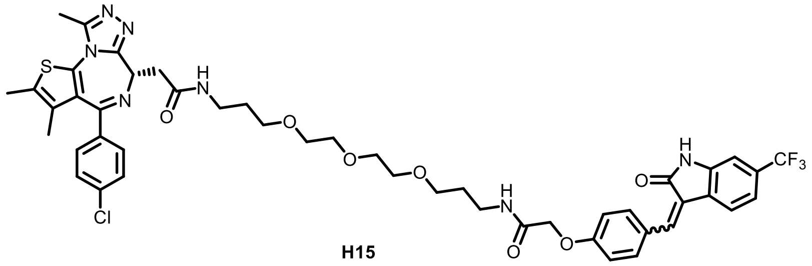

The title compound **H15** (yellow solid, 36.7% yield) was synthesized according to the procedures for the preparation of **H6** from **A2** (4-hydroxybenzaldehyde), **C6** (6-(trifluoromethyl)indolin-2-one), tert-butyl 2-bromoacetate and Linker 2. ^{1}**H NMR** (400 MHz, DMSO-d_6) δ 10.88 (d, J = 10.9 Hz, 1H), 8.54 (d, J = 8.7 Hz, 1H), 8.17 (dd, J = 14.9, 8.4 Hz, 2H), 8.01 – 7.85 (m, 1H), 7.82 (d, J = 8.0 Hz, 1H), 7.76 (d, J = 3.1 Hz, 2H), 7.45 (dd, J = 23.6, 8.3 Hz, 4H), 7.34 (d, J = 7.6 Hz, 1H), 7.23 (d, J = 7.8 Hz, 1H), 7.09 (dd, J = 23.4, 8.8 Hz, 3H), 4.59 (s, 2H), 4.55 – 4.47 (m, 1H), 3.57 – 3.40 (m, 13H), 3.23 (dd, J = 12.9, 6.8 Hz, 6H), 2.59 (s, 3H), 2.40 (s, 3H), 1.73 – 1.65 (m, 4H), 1.61 (s, 3H). ^{13}**C NMR** (101 MHz, DMSO-d_6) δ 169.92, 169.04, 168.35,

167.60, 167.55, 163.51, 159.97, 155.59, 150.24, 143.52, 139.78, 137.24, 135.73, 135.42, 132.76, 132.31, 131.15, 130.58, 130.31, 130.00, 128.92, 127.21, 125.51, 125.36, 124.88, 122.94, 118.37, 115.60, 115.05, 106.51, 70.22, 70.04, 68.66, 67.45, 54.35, 38.11, 36.34, 29.91, 29.74, 14.48, 13.10, 11.73. **ESI-MS**: *m*/*z* [M + H]$^+$ calcd for $C_{47}H_{50}O_7N_7ClF_3S^+$, 948.3128; found, 948.3132; purity: > 99%.

(*S*, *Z*)-2-(3-chloro-4-((5-fluoro-2-oxoindolin-3-ylidene)methyl)phenoxy)-*N*-(1-(4-(4-chlorophenyl)-2,3,9-trimethyl-6*H*-thieno[3,2-*f*][1,2,4]triazolo[4,3-*a*][1,4]diazepin-6-yl)-2-oxo-7,10,13-trioxa-3-azahexadecan-16-yl)acetamide (H16)

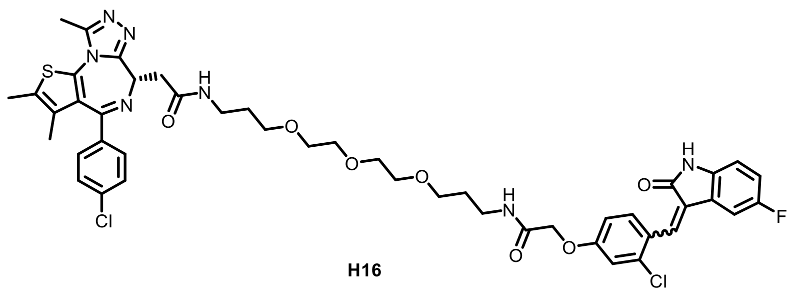

H16

The title compound **H16** (yellow solid, 41.2% yield) was synthesized according to the procedures for the preparation of **H6** from **A3** (2-chloro-4-hydroxybenzaldehyde), **C3** (5-fluoroindolin-2-one), tert-butyl 2-bromoacetate and Linker 2. **^{1}H NMR** (400 MHz, DMSO-*d*$_6$) δ 10.63 (d, *J* = 40.6 Hz, 1H), 8.17 (d, *J* = 5.4 Hz, 2H), 7.88 – 7.69 (m, 1H), 7.61 (s, 1H), 7.45 (dd, *J* = 23.6, 7.0 Hz, 4H), 7.27 (s, 1H), 7.18 – 6.93 (m, 3H), 6.88 (s, 1H), 4.62 (s, 2H), 4.51 (s, 1H), 3.46 (dd, *J* = 24.5, 10.0 Hz, 13H), 3.21 (s, 6H), 2.59 (s, 3H), 2.40 (s, 3H), 1.67 (d, *J* = 5.9 Hz, 4H), 1.61 (s, 3H). **^{13}C NMR** (101 MHz, DMSO-*d*$_6$) δ 169.92, 168.74, 167.33, 163.49, 160.15, 155.57, 150.26, 139.81, 137.21, 135.71, 134.84, 133.45, 132.72, 131.76, 131.15, 130.57, 130.29, 130.02, 128.93, 125.29, 117.25, 117.01, 116.64, 114.73, 70.22, 70.02, 68.59, 68.51, 67.68, 54.35, 38.11, 36.31, 36.26, 29.92, 29.73, 14.50, 13.12, 11.75. **ESI-MS**: *m*/*z* [M + H]$^+$ calcd for $C_{46}H_{49}O_7N_7Cl_2FS^+$, 932.2770; found, 932.2779; purity: > 99%.

(*S*, *Z*)-2-(3-chloro-4-((5-chloro-2-oxoindolin-3-ylidene)methyl)phenoxy)-*N*-(1-(4-(4-chlorophenyl)-2,3,9 -trimethyl-6*H*-thieno[3,2-*f*][1,2,4]triazolo[4,3-*a*][1,4]diazepin-6-yl)-2-oxo-7,10,13-trioxa-3-azahexadecan-16-yl)acetamide (H17)

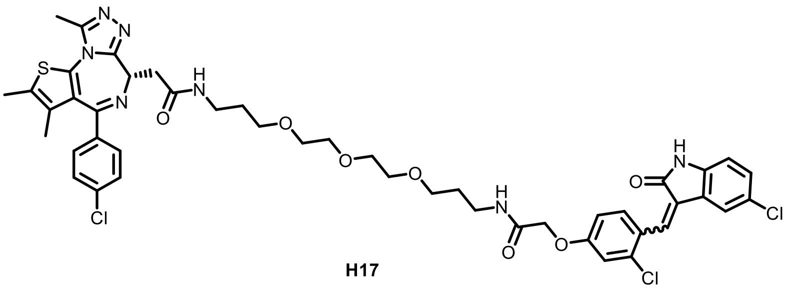

H17

The title compound **H17** (yellow solid, 38.9% yield) was synthesized according to the procedures for the preparation of **H6** from **A3** (2-chloro-4-hydroxybenzaldehyde), **C4** (5-chloroindolin-2-one), tert-butyl 2-bromoacetate and Linker 2. **^{1}H NMR** (400 MHz, DMSO-d_6) δ 10.75 (d, J = 42.0 Hz, 1H), 8.18 (d, J = 6.5 Hz, 2H), 7.81 (dd, J = 31.0, 19.9 Hz, 2H), 7.61 (s, 1H), 7.45 (dd, J = 24.0, 8.3 Hz, 4H), 7.33 – 7.25 (m, 2H), 7.23 (s, 1H), 7.18 – 7.07 (m, 1H), 6.90 (d, J = 8.3 Hz, 1H), 4.61 (d, J = 11.0 Hz, 2H), 4.51 (t, J = 6.9 Hz, 1H), 3.55 – 3.38 (m, 16H), 3.28 – 3.10 (m, 6H), 2.59 (s, 3H), 2.40 (s, 3H), 1.71 – 1.65 (m, 4H), 1.61 (s, 3H). **^{13}C NMR** (101 MHz, DMSO-d_6) δ 169.91, 168.46, 167.31, 163.48, 160.21, 155.57, 150.25, 142.24, 137.21, 135.70, 134.93, 133.66, 132.72, 131.79, 131.14, 130.57, 130.29, 130.02, 128.92, 127.88, 125.52, 125.30, 122.74, 122.34, 116.59, 114.74, 112.08, 70.23, 70.02, 68.60, 68.51, 67.69, 54.36, 38.11, 36.32, 36.25, 29.92, 29.74, 14.50, 13.12, 11.75. **ESI-MS**: m/z $[M + H]^+$ calcd for $C_{46}H_{49}O_7N_7Cl_3S^+$, 950.2445; found, 950.2448; purity: > 99%.

(*S, Z*)-2-(4-((5-bromo-2-oxoindolin-3-ylidene)methyl)-3-chlorophenoxy)-*N*-(1-(4-(4-chlorophenyl)-2,3,9-trimethyl-6*H*-thieno[3,2-*f*][1,2,4]triazolo[4,3-*a*][1,4]diazepin-6-yl)-2-oxo-7,10,13-trioxa-3-azahexadecan-16-yl)acetamide (H18)

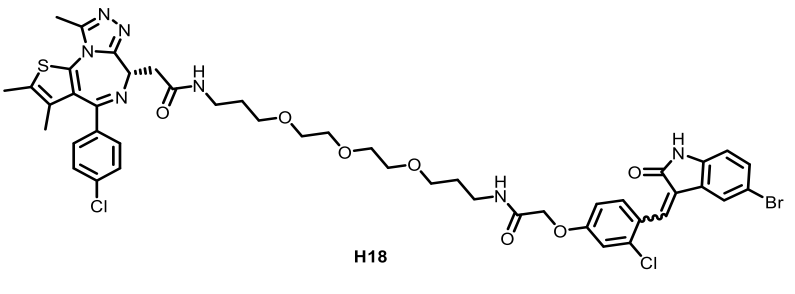

H18

The title compound **H18** (yellow solid, 33.9% yield) was synthesized according to the procedures for the preparation of **H6** from **A3** (2-chloro-4-hydroxybenzaldehyde), **C5** (5-bromoindolin-2-one), tert-butyl 2-

bromoacetate and Linker 2. **^{1}H NMR** (400 MHz, DMSO-*d*$_6$) δ 10.77 (d, *J* = 42.2 Hz, 1H), 8.19 (d, *J* = 5.4 Hz, 2H), 7.91 (d, *J* = 17.1 Hz, 1H), 7.78 (d, *J* = 8.5 Hz, 1H), 7.61 (s, 1H), 7.45 (dd, *J* = 23.6, 8.1 Hz, 5H), 7.33 (d, *J* = 37.8 Hz, 2H), 7.14 (d, *J* = 8.7 Hz, 1H), 6.82 (dd, *J* = 29.9, 8.1 Hz, 1H), 4.62 (d, *J* = 10.9 Hz, 2H), 4.52 (t, *J* = 6.7 Hz, 1H), 3.56 – 3.39 (m, 15H), 3.25 (dd, *J* = 19.3, 10.7 Hz, 6H), 2.60 (s, 3H), 2.40 (s, 3H), 1.69 (s, 4H), 1.62 (s, 3H). **^{13}C NMR** (101 MHz, DMSO-*d*$_6$) δ 169.92, 168.33, 167.30, 163.45, 160.22, 155.53, 150.25, 142.60, 137.17, 135.66, 134.96, 133.64, 133.10, 132.72, 131.78, 131.14, 130.57, 130.25, 129.98, 128.92, 127.74, 125.30, 125.05, 124.71, 123.21, 116.57, 114.72, 113.16, 112.58, 70.23, 70.02, 68.61, 67.70, 54.35, 38.11, 36.30, 29.92, 29.72, 14.51, 13.13, 11.72. **ESI-MS**: *m*/*z* [M + H]$^+$ calcd for $C_{46}H_{49}O_7N_7Cl_2BrS^+$, 994.1949; found, 994.1948; purity: > 99%.

(*S*, *Z*)-2-(3-chloro-4-((2-oxo-6-(trifluoromethyl)indolin-3-ylidene)methyl)phenoxy)-*N*-(1-(4-(4-chloro-phenyl)-2,3,9-trimethyl-6*H*-thieno[3,2-*f*][1,2,4]triazolo[4,3-*a*][1,4]diazepin-6-yl)-2-oxo-7,10,13-trioxa-3-azahexadecan-16-yl)acetamide (H19)

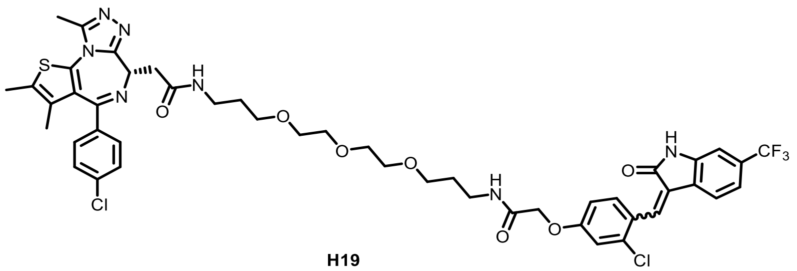

H19

The title compound **H19** (yellow solid, 43.6% yield) was synthesized according to the procedures for the preparation of **H6** from **A3** (2-chloro-4-hydroxybenzaldehyde), **C6** (6-(trifluoromethyl)indolin-2-one), tert-butyl 2-bromoacetate and Linker 2. **^{1}H NMR** (400 MHz, DMSO-*d*$_6$) δ 10.93 (d, *J* = 26.3 Hz, 1H), 8.19 (d, *J* = 6.1 Hz, 2H), 7.80 (d, *J* = 8.6 Hz, 1H), 7.72 (s, 1H), 7.45 (dd, *J* = 23.8, 8.5 Hz, 5H), 7.33 (d, *J* = 8.4 Hz, 1H), 7.28 (s, 1H), 7.20 (d, *J* = 8.1 Hz, 1H), 7.14 – 6.96 (m, 2H), 4.63 (s, 2H), 4.52 (t, *J* = 6.9 Hz, 1H), 3.46 (ddd, *J* = 18.0, 12.0, 7.9 Hz, 16H), 3.21 (dt, *J* = 23.5, 12.7 Hz, 6H), 2.60 (s, 3H), 2.40 (s, 3H), 1.69 (d, *J* = 6.1 Hz, 4H), 1.62 (s, 3H). **^{13}C NMR** (101 MHz, DMSO-*d*$_6$) δ 169.93, 168.47, 167.28, 163.49, 160.35, 155.57, 150.26, 143.88, 137.21, 135.70, 135.11, 132.72, 131.96, 131.14, 130.56, 130.29, 130.02, 128.92, 127.42, 125.21,

124.90, 123.34, 118.58, 116.59, 114.86, 70.23, 70.02, 68.60, 68.51, 67.68, 54.35, 38.12, 36.31, 36.26, 29.91, 29.74, 14.49, 13.11, 11.74. **ESI-MS**: *m*/*z* $[M + H]^+$ calcd for $C_{47}H_{48}O_7N_7Cl_2F_3S^+$, 982.2719; found, 982.2745; purity: > 99%.

(*S*, *Z*)-2-(2-bromo-4-((5-fluoro-2-oxoindolin-3-ylidene)methyl)phenoxy)-*N*-(1-(4-(4-chlorophenyl)-2,3,9-trimethyl-6*H*-thieno[3,2-*f*][1,2,4]triazolo[4,3-*a*][1,4]diazepin-6-yl)-2-oxo-7,10,13-trioxa-3-azahexadecan-16-yl)acetamide (H20)

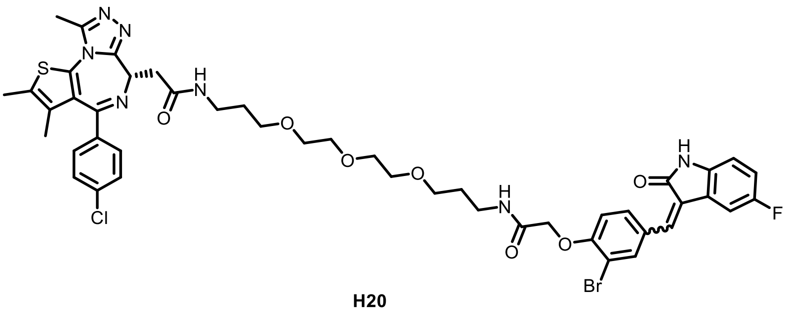

H20

The title compound **H20** (yellow solid, 52.3% yield) was synthesized according to the procedures for the preparation of **H6** from **A4** (3-bromo-4-hydroxybenzaldehyde), **C3** (5-fluoroindolin-2-one), tert-butyl 2-bromoacetate and Linker 2. **^{1}H NMR** (400 MHz, DMSO-*d*$_6$) δ 10.64 (d, J = 10.7 Hz, 1H), 8.19 (s, 1H), 7.96 (s, 2H), 7.87 – 7.69 (m, 1H), 7.60 (d, J = 15.8 Hz, 1H), 7.45 (dd, J = 24.4, 7.5 Hz, 4H), 7.25 (d, J = 8.2 Hz, 1H), 7.19 – 6.95 (m, 2H), 6.84 (d, J = 26.0 Hz, 1H), 4.71 (s, 2H), 4.52 (s, 1H), 3.46 (dd, J = 25.3, 8.2 Hz, 13H), 3.23 (s, 6H), 2.59 (s, 3H), 2.40 (s, 3H), 1.68 (s, 4H), 1.62 (s, 3H). **^{13}C NMR** (101 MHz, DMSO-*d*$_6$) δ 169.91, 169.02, 167.78, 167.14, 167.06, 166.01, 163.48, 155.90, 155.57, 150.25, 139.75, 137.21, 136.32, 135.70, 134.66, 134.36, 132.73, 131.14, 130.73, 130.57, 130.28, 130.02, 128.92, 128.80, 122.23, 116.75, 114.35, 111.75, 70.23, 70.04, 70.01, 68.55, 68.51, 68.32, 55.39, 54.36, 38.11, 36.37, 36.25, 29.92, 29.65, 14.50, 13.12, 11.75. **ESI-MS**: *m*/*z* $[M + H]^+$ calcd for $C_{46}H_{49}O_7N_7ClBrFS^+$, 978.2244; found, 978.2243; purity: > 99%.

(*S*, *Z*)-2-(2-bromo-4-((5-chloro-2-oxoindolin-3-ylidene)methyl)phenoxy)-*N*-(1-(4-(4-chlorophenyl)-2,3,9-trimethyl-6*H*-thieno[3,2-*f*][1,2,4]triazolo[4,3-*a*][1,4]diazepin-6-yl)-2-oxo-7,10,13-trioxa-3-

azahexadecan-16-yl)acetamide (H21)

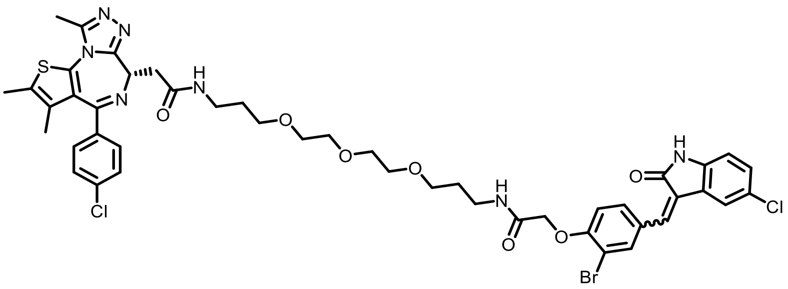

H21

The title compound **H21** (yellow solid, 43.6% yield) was synthesized according to the procedures for the preparation of **H6** from **A4** (3-bromo-4-hydroxybenzaldehyde), **C4** (5-chloroindolin-2-one), tert-butyl 2-bromoacetate and Linker 2. **^{1}H NMR** (400 MHz, DMSO-*d*$_6$) δ 10.76 (d, J = 9.0 Hz, 1H), 8.19 (s, 1H), 8.06 – 7.91 (m, 2H), 7.84 (d, J = 30.1 Hz, 1H), 7.73 (d, J = 8.4 Hz, 1H), 7.63 (s, 1H), 7.45 (dd, J = 24.3, 8.1 Hz, 5H), 7.26 (dd, J = 25.1, 8.3 Hz, 1H), 7.13 (dd, J = 17.8, 8.5 Hz, 1H), 6.87 (dd, J = 25.0, 8.2 Hz, 1H), 4.72 (s, 2H), 4.52 (t, J = 6.8 Hz, 1H), 3.46 (dd, J = 25.5, 8.3 Hz, 13H), 3.31 – 3.06 (m, 6H), 2.59 (s, 3H), 2.40 (s, 3H), 1.68 (s, 4H), 1.61 (s, 3H). **^{13}C NMR** (101 MHz, DMSO-*d*$_6$) δ 169.91, 168.75, 167.53, 167.13, 163.48, 155.96, 155.57, 150.25, 142.16, 139.64, 137.41, 137.21, 137.09, 136.53, 135.70, 134.64, 134.45, 132.73, 131.13, 130.88, 130.57, 130.29, 130.01, 129.07, 128.92, 128.76, 126.66, 125.89, 125.41, 124.99, 122.96, 122.08, 114.30, 113.74, 112.00, 111.73, 111.20, 70.23, 70.05, 68.56, 68.52, 68.33, 55.39, 54.36, 38.12, 36.39, 36.26, 29.92, 29.66, 14.51, 13.13, 11.76. **ESI-MS**: *m*/*z* [M + H]$^+$ calcd for $C_{46}H_{49}O_7N_7Cl_2BrS^+$, 994.1949; found, 994.1952; purity: > 99%.

(*S, Z*)-2-(2-bromo-4-((5-bromo-2-oxoindolin-3-ylidene)methyl)phenoxy)-*N*-(1-(4-(4-chlorophenyl)-2,3,9 -trimethyl-6*H*-thieno[3,2-*f*][1,2,4]triazolo[4,3-*a*][1,4]diazepin-6-yl)-2-oxo-7,10,13-trioxa-3-azahexadecan-16-yl)acetamide (H22)

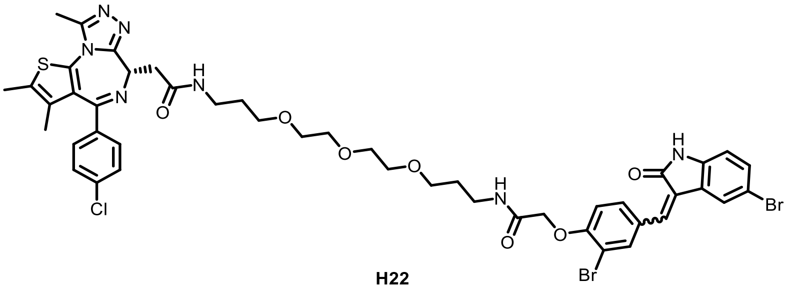

H22

The title compound **H22** (yellow solid, 69.3% yield) was synthesized according to the procedures for the preparation of **H6** from **A4** (3-bromo-4-hydroxybenzaldehyde), **C5** (5-bromoindolin-2-one), tert-butyl 2-bromoacetate and Linker 2. **^{1}H NMR** (400 MHz, DMSO-d_6) δ 10.80 (d, J = 3.3 Hz, 1H), 9.01 (s, 1H), 8.20 (s, 1H), 8.00 (d, J = 8.4 Hz, 1H), 7.95 – 7.78 (m, 2H), 7.63 (d, J = 7.1 Hz, 2H), 7.54 – 7.25 (m, 7H), 7.13 (dd, J = 17.1, 8.6 Hz, 1H), 6.83 (dd, J = 26.1, 8.2 Hz, 1H), 4.72 (s, 2H), 4.51 (d, J = 7.0 Hz, 1H), 3.64 – 3.45 (m, 13H), 3.19 (dd, J = 17.6, 10.1 Hz, 6H), 2.59 (s, 3H), 2.40 (s, 3H), 1.68 (s, 4H), 1.61 (s, 3H). **^{13}C NMR** (101 MHz, DMSO-d_6) δ 169.95, 167.40, 167.15, 167.06, 163.48, 156.51, 155.56, 150.24, 144.26, 143.30, 140.04, 137.42, 137.20, 137.09, 136.48, 135.71, 134.61, 134.49, 132.84, 132.71, 131.34, 131.13, 130.59, 130.27, 129.99, 129.08, 128.95, 127.81, 126.70, 126.66, 124.83, 124.45, 122.85, 119.31, 114.38, 114.28, 113.75, 112.57, 111.72, 110.46, 70.24, 70.03, 68.54, 68.30, 68.22, 68.20, 54.36, 53.86, 42.16, 38.12, 36.39, 36.18, 29.92, 29.65, 14.49, 13.12, 12.85, 11.75. **ESI-MS**: m/z [M + H]$^+$ calcd for $C_{46}H_{49}O_7N_7ClBr_2S^+$, 1038.1444; found, 1038.1455; purity: > 99%.

(*S*, *Z*)-2-(2-bromo-4-((2-oxo-6-(trifluoromethyl)indolin-3-ylidene)methyl)phenoxy)-*N*-(1-(4-(4-chloro-phenyl)-2,3,9-trimethyl-6*H*-thieno[3,2-*f*][1,2,4]triazolo[4,3-*a*][1,4]diazepin-6-yl)-2-oxo-7,10,13-trioxa-3-azahexadecan-16-yl)acetamide (H23)

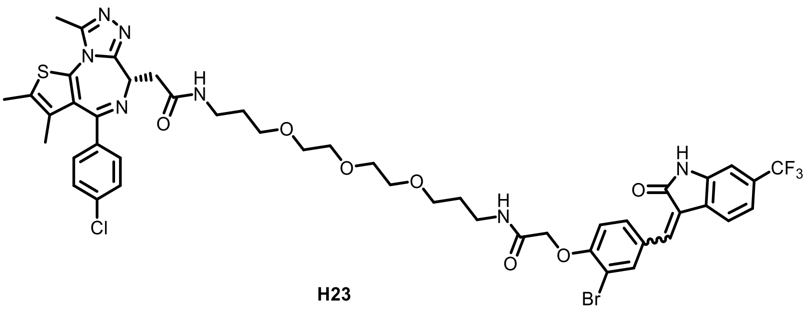

H23

The title compound **H23** (yellow solid, 36.7% yield) was synthesized according to the procedures for the preparation of **H6** from **A4** (3-bromo-4-hydroxybenzaldehyde), **C6** (6-(trifluoromethyl)indolin-2-one), tert-butyl 2-bromoacetate and Linker 2. **^{1}H NMR** (400 MHz, DMSO-d_6) δ 10.92 (d, J = 17.4 Hz, 1H), 8.19 (s, 1H), 7.99 (d, J = 14.8 Hz, 2H), 7.83 (dd, J = 45.1, 8.1 Hz, 1H), 7.71 (d, J = 7.6 Hz, 1H), 7.45 (dd, J = 24.5, 8.2 Hz, 4H), 7.30 (dd, J = 48.2, 8.0 Hz, 1H), 7.11 (dd, J = 24.6, 14.1 Hz, 2H), 4.72 (s, 2H), 4.52 (t, J = 6.8 Hz, 1H), 3.47 (dd, J = 20.0, 9.1 Hz, 12H), 3.31 – 3.07 (m, 6H), 2.59 (s, 3H), 2.40 (s, 3H), 1.69 (d, J = 5.0 Hz, 4H), 1.61 (s, 3H). **^{13}C NMR** (101 MHz, DMSO-d_6) δ 169.93, 168.76, 167.48, 167.02, 163.48, 156.77, 156.15, 155.56, 150.25, 143.79, 141.18, 138.83, 138.10, 137.33, 137.21, 135.69, 134.91, 134.72, 132.73, 131.12, 131.00, 130.56, 130.28, 130.00, 129.45, 128.92, 128.69, 125.14, 124.51, 123.68, 123.33, 122.93, 120.52, 118.40, 114.35, 113.74, 111.83, 111.24, 105.98, 70.22, 70.05, 70.01, 68.55, 68.51, 68.23, 55.38, 54.36, 38.12, 36.37, 36.25, 29.92, 29.64, 14.48, 13.11, 11.75. **ESI-MS**: m/z $[M + H]^+$ calcd for $C_{47}H_{49}O_7N_7ClBrF_3S^+$, 1028.2212; found, 1028.2210; purity: > 99%.

(*S, Z*)-2-(4-bromo-2-((5-fluoro-2-oxoindolin-3-ylidene)methyl)phenoxy)-*N*-(1-(4-(4-chlorophenyl)-2,3,9-trimethyl-6*H*-thieno[3,2-*f*][1,2,4]triazolo[4,3-*a*][1,4]diazepin-6-yl)-2-oxo-7,10,13-trioxa-3-azahexadecan-16-yl)acetamide (H24)

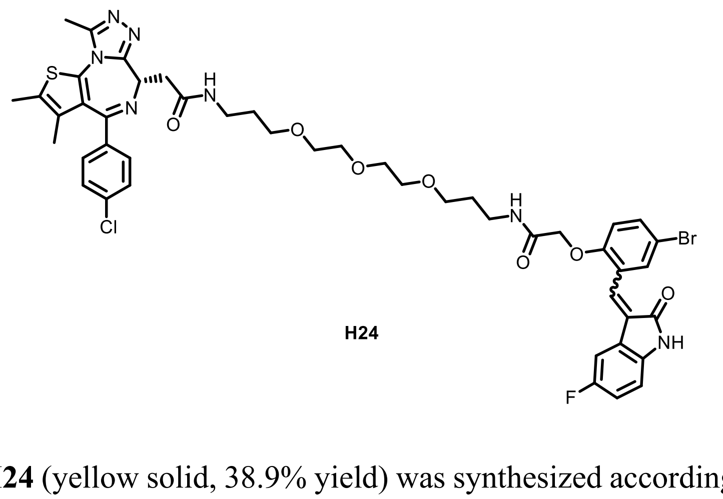

H24

The title compound **H24** (yellow solid, 38.9% yield) was synthesized according to the procedures for the preparation of **H6** from **A5** (5-bromo-2-hydroxybenzaldehyde), **C3** (5-fluoroindolin-2-one), tert-butyl 2-bromoacetate and Linker 2. **^{1}H NMR** (400 MHz, DMSO-d_6) δ 10.66 (s, 1H), 8.19 (s, 1H), 8.00 (s, 1H), 7.80 (s, 1H), 7.75 (s, 1H), 7.65 (d, J = 8.6 Hz, 1H), 7.45 (dd, J = 24.0, 8.1 Hz, 4H), 7.10 (t, J = 8.3 Hz, 1H), 7.01

(d, J = 8.3 Hz, 2H), 6.95 – 6.79 (m, 1H), 4.62 (s, 2H), 4.52 (t, J = 6.8 Hz, 1H), 3.47 (dd, J = 18.2, 5.5 Hz, 13H), 3.28 – 3.11 (m, 6H), 2.60 (s, 3H), 2.40 (s, 3H), 1.71 – 1.57 (m, 7H). **^{13}C NMR** (101 MHz, DMSO-d_6) δ 169.90, 169.20, 168.74, 168.11, 167.41, 163.50, 156.01, 155.57, 150.30, 139.85, 137.20, 135.74, 134.31, 132.77, 132.48, 132.10, 131.18, 130.59, 130.28, 130.02, 128.92, 125.75, 117.19, 116.94, 115.52, 112.63, 112.58, 111.47, 109.91, 109.64, 70.21, 70.00, 68.49, 68.06, 54.36, 38.14, 36.24, 29.91, 29.67, 14.50, 13.17, 11.75. **ESI-MS**: m/z $[M + H]^+$ calcd for $C_{46}H_{49}O_7N_7ClBrFS^+$, 978.2246; found, 978.2248; purity: > 99%.

(*S, Z*)-2-(4-bromo-2-((5-chloro-2-oxoindolin-3-ylidene)methyl)phenoxy)-*N*-(1-(4-(4-chlorophenyl)-2,3,9-trimethyl-6*H*-thieno[3,2-*f*][1,2,4]triazolo[4,3-*a*][1,4]diazepin-6-yl)-2-oxo-7,10,13-trioxa-3-azahexadecan-16-yl)acetamide (H25)

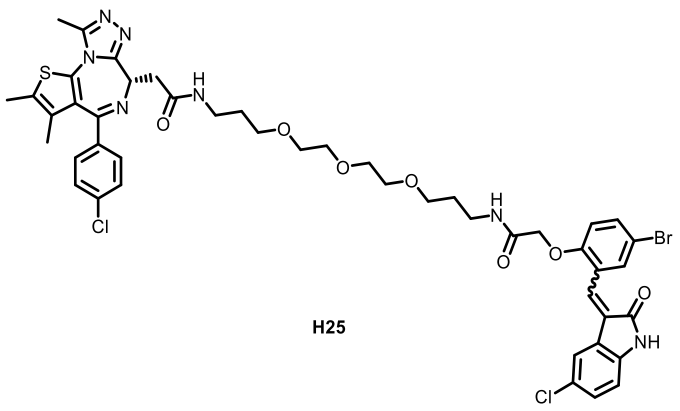

H25

The title compound **H25** (yellow solid, 58.4% yield) was synthesized according to the procedures for the preparation of **H6** from **A5** (5-bromo-2-hydroxybenzaldehyde), **C4** (5-chloroindolin-2-one), tert-butyl 2-bromoacetate and Linker 2. **^{1}H NMR** (400 MHz, DMSO-d_6) δ 10.76 (d, J = 9.7 Hz, 1H), 8.19 (s, 1H), 8.09 (d, J = 16.8 Hz, 1H), 8.01 (s, 1H), 7.82 (s, 1H), 7.76 (s, 1H), 7.65 (d, J = 8.6 Hz, 1H), 7.45 (dd, J = 23.3, 7.9 Hz, 4H), 7.28 (d, J = 14.2 Hz, 2H), 6.99 (dd, J = 16.9, 8.9 Hz, 1H), 6.87 (dd, J = 21.7, 8.1 Hz, 1H), 4.62 (s, 2H), 4.52 (t, J = 6.3 Hz, 1H), 3.47 (dd, J = 17.5, 5.7 Hz, 12H), 3.29 – 3.07 (m, 6H), 2.60 (s, 3H), 2.40 (s, 3H), 1.65 (d, J = 23.6 Hz, 7H). **^{13}C NMR** (101 MHz, DMSO-d_6) δ 174.71, 169.95, 168.51, 167.36, 163.46, 156.09, 155.59, 150.29, 142.28, 137.19, 135.72, 134.38, 132.70, 132.23, 131.65, 131.13, 130.57, 130.27, 130.01, 128.90, 128.22, 125.66, 125.41, 122.38, 120.58, 115.48, 112.60, 112.01, 70.20, 70.00, 68.49, 36.24, 29.95,

29.67, 14.53, 13.16, 11.80. **ESI-MS**: *m/z* [M + H]$^+$ calcd for $C_{46}H_{49}O_7N_7Cl_2BrS^+$, 994.1949; found, 994.1950; purity: > 99%.

(*S, Z*)-2-(4-bromo-2-((5-bromo-2-oxoindolin-3-ylidene)methyl)phenoxy)-*N*-(1-(4-(4-chlorophenyl)-2,3,9-trimethyl-6*H*-thieno[3,2-*f*][1,2,4]triazolo[4,3-*a*][1,4]diazepin-6-yl)-2-oxo-7,10,13-trioxa-3-azahexadecan-16-yl)acetamide (H26)

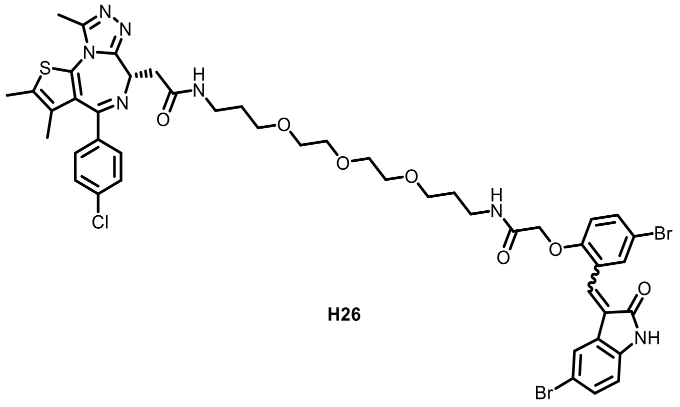

H26

The title compound **H26** (yellow solid, 46.9% yield) was synthesized according to the procedures for the preparation of **H6** from **A5** (5-bromo-2-hydroxybenzaldehyde), **C5** (5-bromoindolin-2-one), tert-butyl 2-bromoacetate and Linker 2. **^{1}H NMR** (400 MHz, DMSO-*d*$_6$) δ 10.76 (d, *J* = 9.1 Hz, 1H), 8.19 (s, 1H), 8.00 (s, 1H), 7.82 (s, 1H), 7.75 (s, 1H), 7.65 (d, *J* = 8.8 Hz, 1H), 7.54 – 7.33 (m, 6H), 6.99 (dd, *J* = 16.1, 8.9 Hz, 1H), 6.93 – 6.73 (m, 1H), 4.62 (s, 2H), 4.51 (t, *J* = 6.7 Hz, 1H), 3.62 – 3.38 (m, 12H), 3.20 (ddd, *J* = 21.9, 18.1, 10.6 Hz, 6H), 2.59 (s, 3H), 2.40 (s, 3H). **^{13}C NMR** (101 MHz, DMSO-*d*$_6$) δ 169.91, 168.34, 167.48, 167.33, 167.09, 163.47, 156.29, 156.06, 155.57, 150.25, 142.60, 137.20, 135.69, 134.39, 134.16, 132.99, 132.72, 132.62, 132.22, 131.64, 131.14, 130.57, 130.29, 130.02, 128.92, 128.10, 127.16, 126.98, 125.65, 125.19, 123.44, 115.47, 113.08, 112.56, 70.21, 70.01, 68.51, 68.04, 54.35, 38.11, 36.25, 29.92, 29.64, 14.51, 13.13, 11.76. **ESI-MS**: *m/z* [M + H]$^+$ calcd for $C_{46}H_{49}O_7N_7ClBr_2S^+$, 1038.1444; found, 1038.1451; purity: > 99%.

(*S, Z*)-2-(4-bromo-2-((2-oxo-6-(trifluoromethyl)indolin-3-ylidene)methyl)phenoxy)-*N*-(1-(4-(4-chlorophenyl)-2,3,9-trimethyl-6*H*-thieno[3,2-*f*][1,2,4]triazolo[4,3-*a*][1,4]diazepin-6-yl)-2-oxo-7,10,13-trioxa-3-azahexadecan-16-yl)acetamide (H27)

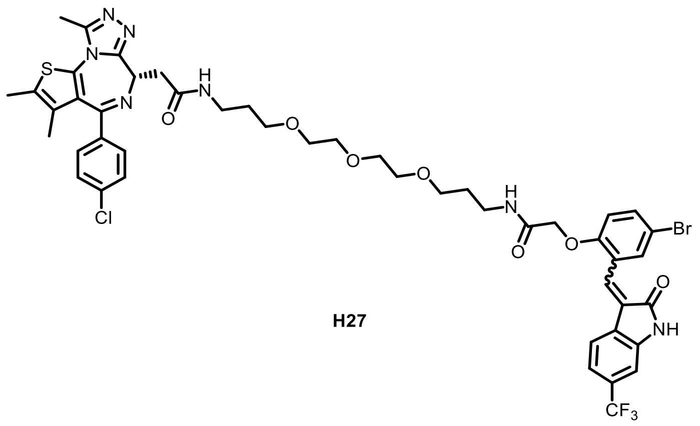

H27

The title compound **H27** (yellow solid, 29.8% yield) was synthesized according to the procedures for the preparation of **H6** from **A5** (5-bromo-2-hydroxybenzaldehyde), **C6** (6-(trifluoromethyl)indolin-2-one), tert-butyl 2-bromoacetate and Linker 2. **^{1}H NMR** (400 MHz, DMSO-*d*$_6$) δ 10.91 (s, 1H), 8.18 (s, 1H), 7.98 (s, 1H), 7.83 (d, *J* = 11.1 Hz, 2H), 7.74 – 7.56 (m, 1H), 7.56 – 7.32 (m, 5H), 7.25 (d, *J* = 7.5 Hz, 1H), 7.14 – 6.92 (m, 2H), 4.63 (s, 2H), 4.51 (s, 1H), 3.47 (d, *J* = 22.6 Hz, 13H), 3.27 – 3.07 (m, 6H), 2.59 (s, 3H), 2.40 (s, 3H), 1.65 (d, *J* = 23.5 Hz, 7H). **^{13}C NMR** (101 MHz, DMSO-*d*$_6$) δ 169.92, 168.50, 167.34, 163.48, 161.74, 156.47, 156.06, 155.57, 153.36, 150.27, 144.75, 143.88, 137.89, 137.21, 135.70, 135.01, 134.57, 134.12, 133.50, 132.72, 132.28, 131.15, 130.57, 130.29, 130.02, 128.92, 127.86, 127.21, 126.64, 125.66, 125.14, 123.29, 120.83, 118.55, 115.48, 112.76, 106.66, 70.20, 70.00, 68.51, 68.03, 54.35, 38.11, 36.25, 29.91, 29.62, 14.50, 13.12, 11.75. **ESI-MS**: *m*/*z* $[M + H]^+$ calcd for $C_{47}H_{49}O_7N_7ClBrF_3S^+$, 1028.2212; found, 1028.2217; purity: > 99%.

(*S, Z*)-2-(4-(4-chlorophenyl)-2,3,9-trimethyl-6*H*-thieno[3,2-*f*][1,2,4]triazolo[4,3-*a*][1,4]diazepin-6-yl)-*N*-(1-(2,6-difluoro-4-((2-oxo-6-(trifluoromethyl)indolin-3-ylidene)methyl)phenoxy)-2-oxo-7,10,13-trioxa-3-azahexadecan-16-yl)acetamide (H28)

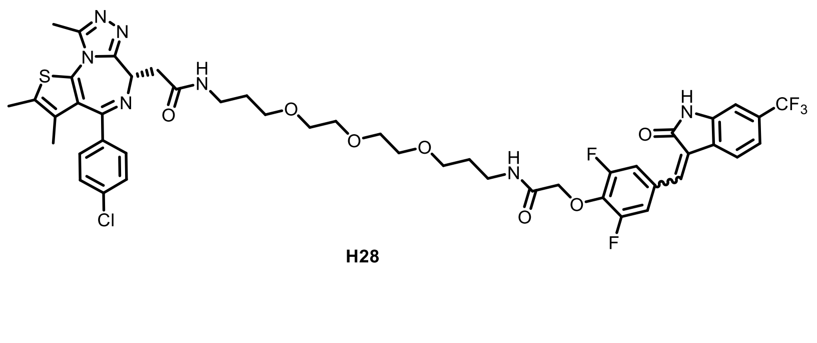

H28

The title compound **H28** (yellow solid, 22.3% yield) was synthesized according to the procedures for the preparation of **H6** from **A6** (3,5-difluoro-4-hydroxybenzaldehyde), **C6** (6-(trifluoromethyl)-2-one), tert-butyl 2-bromoacetate and Linker 2. **^{1}H NMR** (400 MHz, DMSO-d_6) δ 10.98 (d, J = 36.3 Hz, 1H), 8.35 (d, J = 10.2 Hz, 1H), 8.20 (s, 1H), 8.12 (s, 1H), 7.95 (s, 1H), 7.87 (d, J = 7.8 Hz, 1H), 7.69 (d, J = 9.9 Hz, 1H), 7.56 (d, J = 8.8 Hz, 1H), 7.48 (d, J = 8.3 Hz, 2H), 7.40 (dd, J = 15.1, 8.2 Hz, 3H), 7.08 (d, J = 17.7 Hz, 1H), 4.70 (d, J = 8.1 Hz, 2H), 4.51 (t, J = 6.9 Hz, 1H), 3.46 (dd, J = 26.4, 8.3 Hz, 12H), 3.30 – 3.04 (m, 6H), 2.59 (s, 3H), 2.40 (s, 3H), 1.66 (dd, J = 18.7, 12.6 Hz, 7H). **^{13}C NMR** (101 MHz, DMSO-d_6) δ 169.92, 167.29, 163.48, 155.56, 152.62, 150.25, 144.05, 141.58, 137.75, 137.21, 135.70, 132.73, 131.13, 130.57, 130.28, 130.01, 128.92, 126.57, 120.90, 117.05, 116.80, 70.22, 70.01, 68.58, 68.51, 54.36, 38.11, 36.29, 29.92, 29.67, 14.48, 13.11, 11.74. **ESI-MS**: *m/z* $[M + H]^+$ calcd for $C_{47}H_{48}O_7N_7ClF_5S^+$, 984.2950; found, 984.2942; purity: > 99%.

Tert-butyl (Z)-2-(4-bromo-2-((2-oxo-6-(trifluoromethyl)indolin-3-ylidene)methyl)phenoxy)acetate (D27)

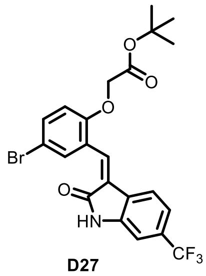

D27

The title compound **D27** (yellow solid, 56.8% yield) was synthesized according to the procedures for the preparation of **D1** from **A5** (5-bromo-2-hydroxybenzaldehyde), **C6** (6-(trifluoromethyl)indolin-2-one) and tert-butyl 2-bromoacetate. **^{1}H NMR** (400 MHz, DMSO-d_6) δ 10.92 (s, 1H), 7.82 (d, J = 2.2 Hz, 1H), 7.75 (s, 1H), 7.66 (dd, J = 8.9, 2.2 Hz, 1H), 7.46 (d, J = 8.0 Hz, 1H), 7.25 (d, J = 8.0 Hz, 1H), 7.12 – 7.06 (m, 2H), 4.81 (s, 2H), 1.38 (s, 9H). **^{13}C NMR** (101 MHz, DMSO-d_6) δ 168.49, 167.68, 155.81, 143.85, 134.58, 133.52, 132.27, 128.00, 125.52, 125.15, 123.36, 118.61, 115.56, 114.97, 112.84, 112.43, 106.74, 99.99, 82.21, 66.03, 28.07. **ESI-MS**: *m/z* $[M + H]^+$ calcd for $C_{22}H_{20}O_4NBrF_3^+$, 498.0522; found, 498.0529; purity: 97%.
