# Supplementary material for: Alkenyl oxindole is a novel PROTAC moiety that recruits the CRL4DCAF11 E3 ubiquitin ligase complex for targeted protein degradation
Source: PLoS Biol. 2024 May 20;22(5):e3002550. doi: 10.1371/journal.pbio.3002550 (PMC11104598; doi:10.1371/journal.pbio.3002550)
Supplement: S3 Data — (PDF) [file pbio.3002550.s011.pdf]

0 h

(*E*) isomer

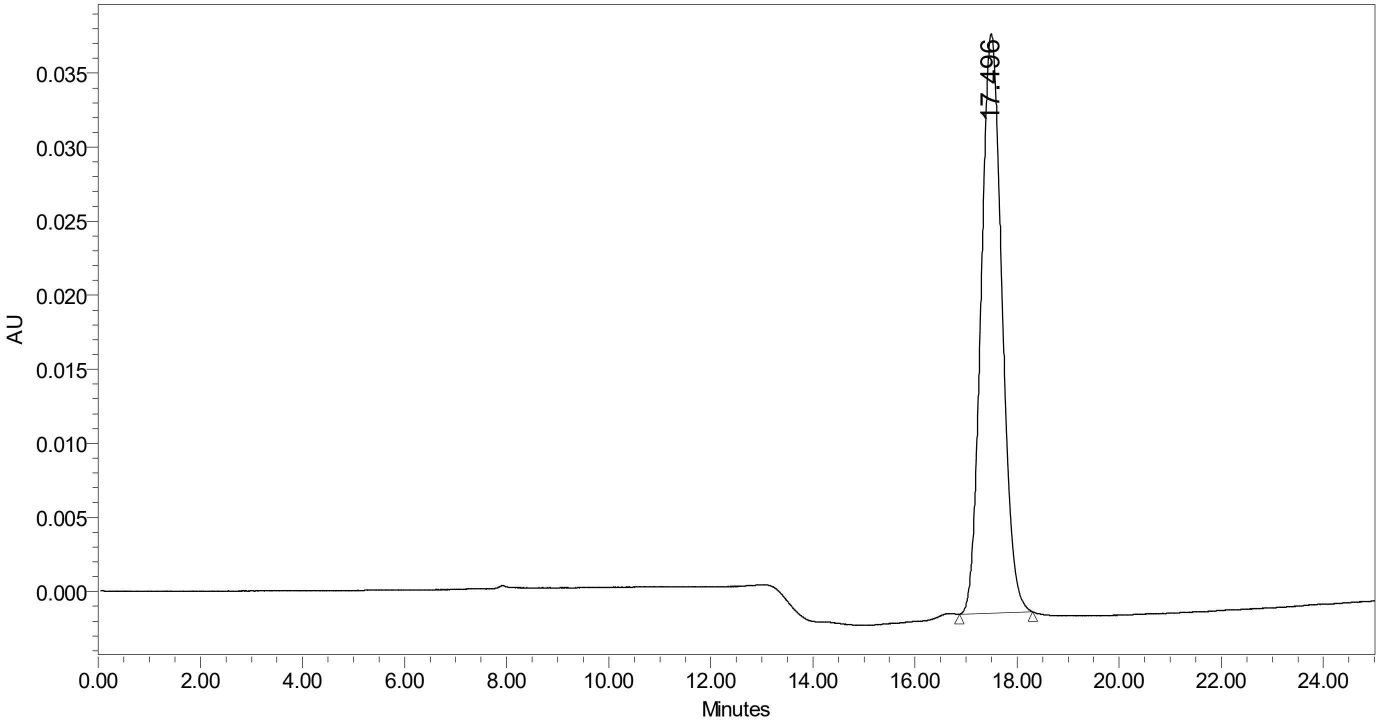

	RT	Area	% Area	Height
1	17.496	1113157	100.00	39113

4℃ 24 h (*E*) isomer

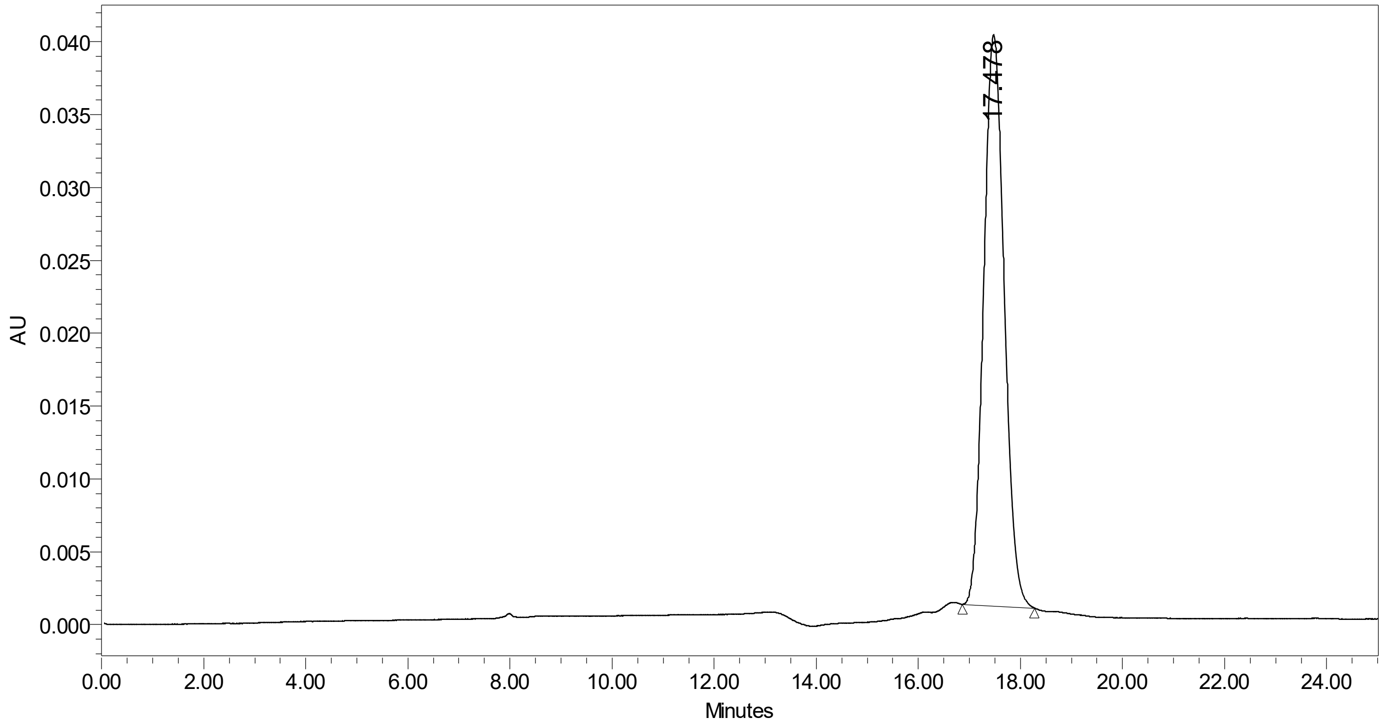

	RT	Area	% Area	Height
1	17.478	1103429	100.00	39201

4℃ 72 h (*E*) isomer

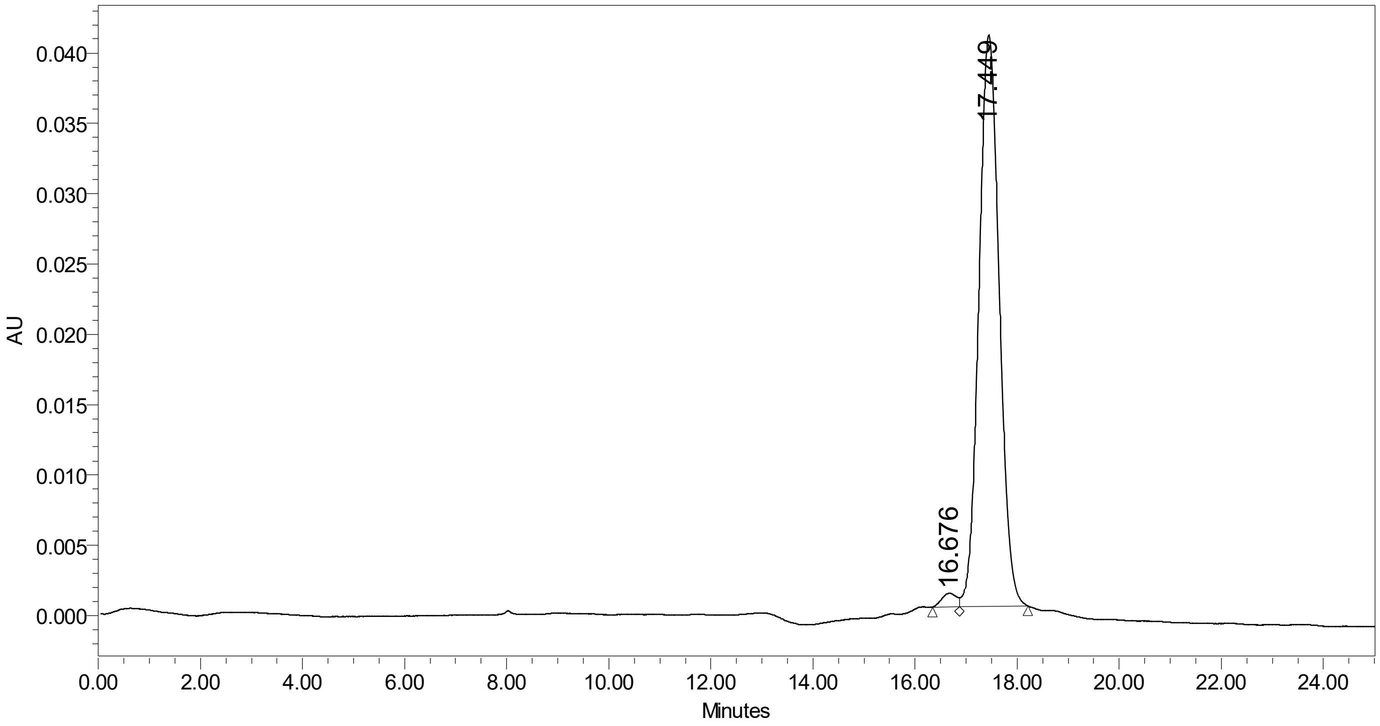

	RT	Area	% Area	Height
1	16.676	19343	1.68	961
2	17.449	1130761	98.32	40629

50℃ 1 h (*E*) isomer

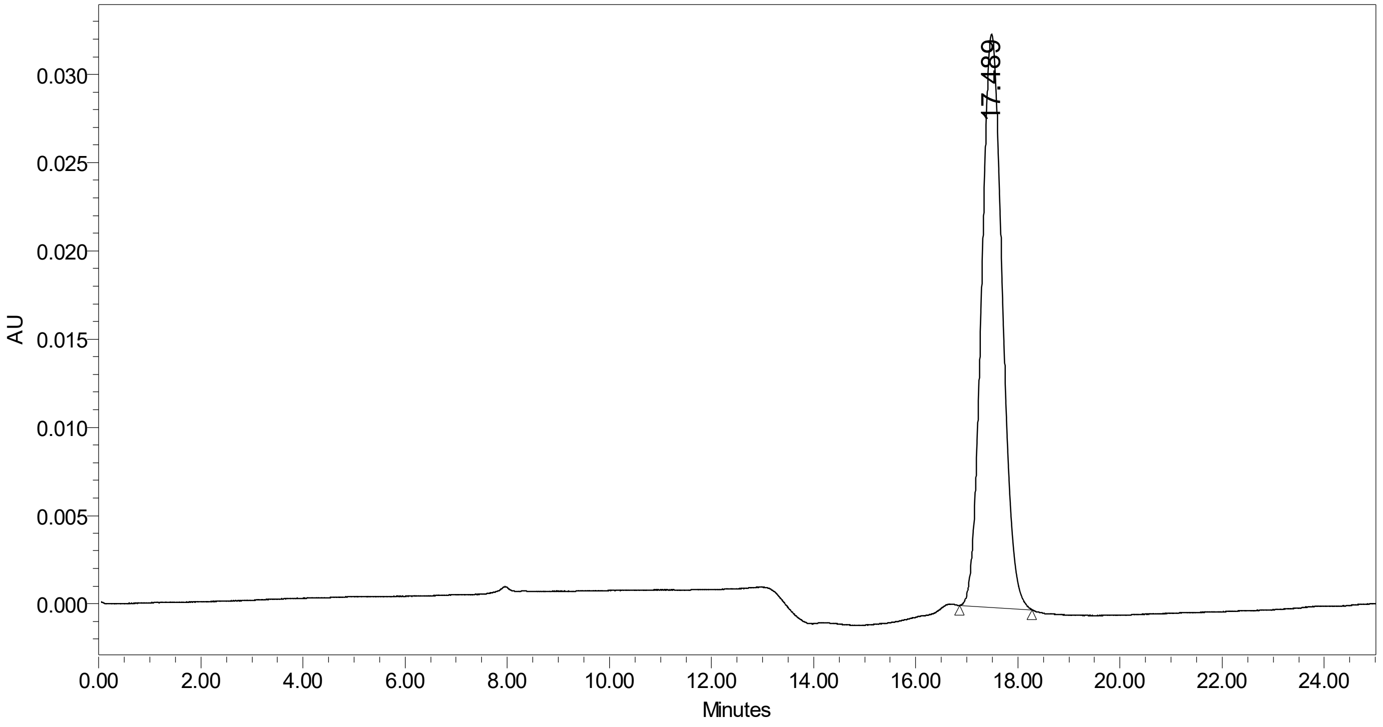

	RT	Area	% Area	Height
1	17.489	922015	100.00	32476

50℃ 2 h (*E*) isomer

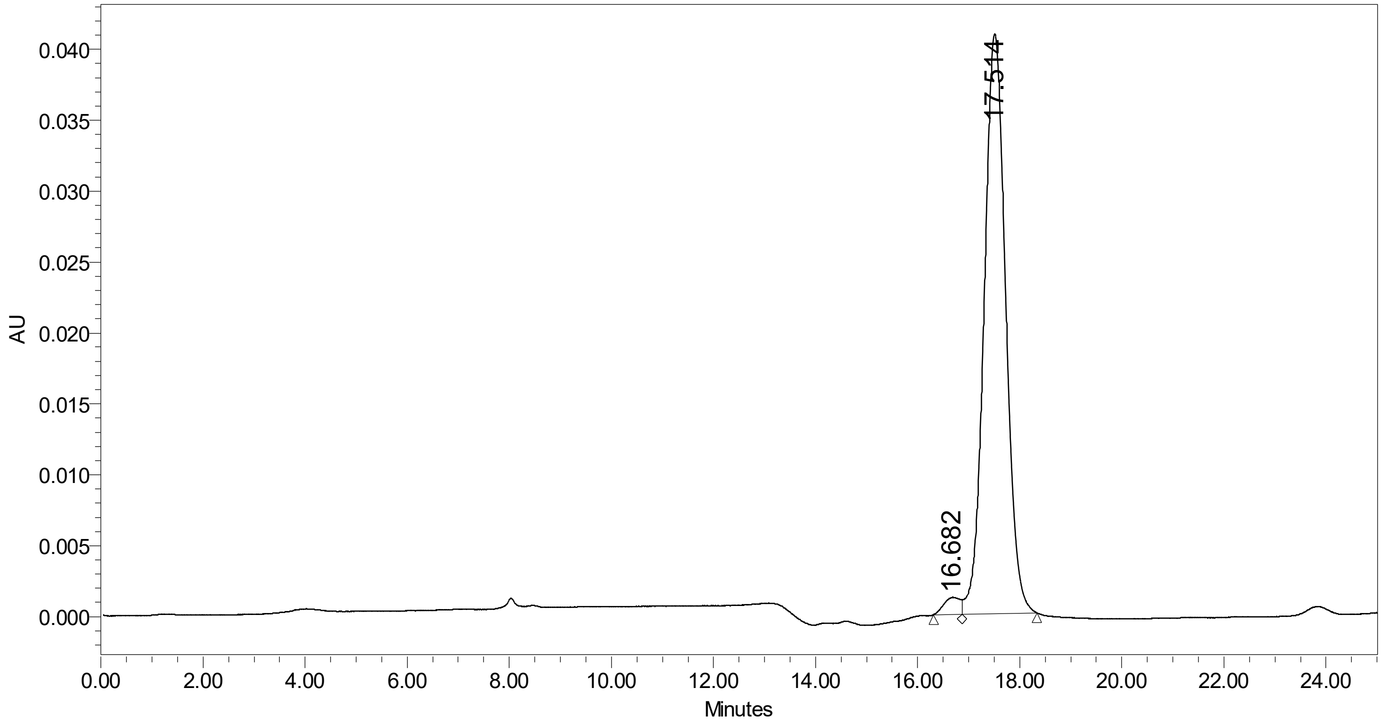

	RT	Area	% Area	Height
1	16.682	26571	2.17	1200
2	17.514	1199863	97.83	40875

50℃ 7 h

(*E*) isomer

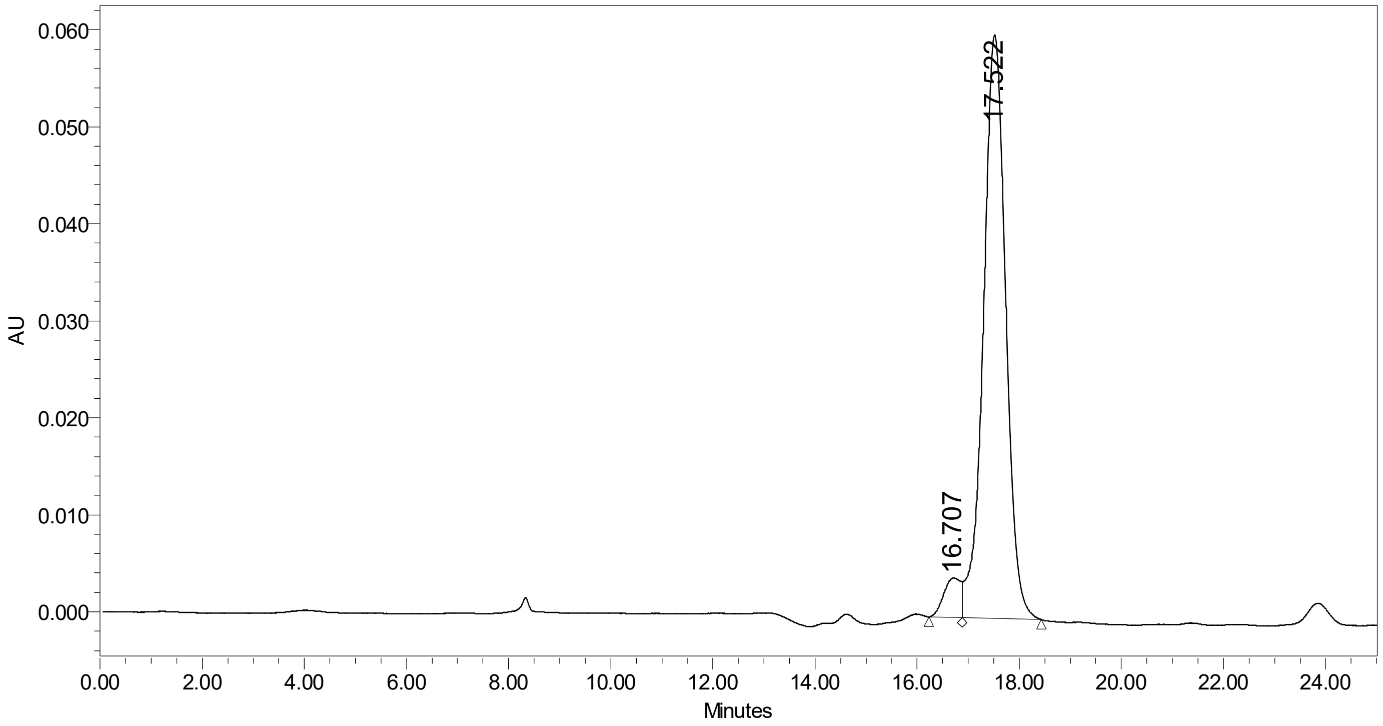

	RT	Area	% Area	Height
1	16.707	93896	4.88	4062
2	17.522	1831123	95.12	60160

rt 24 h (*E*) isomer

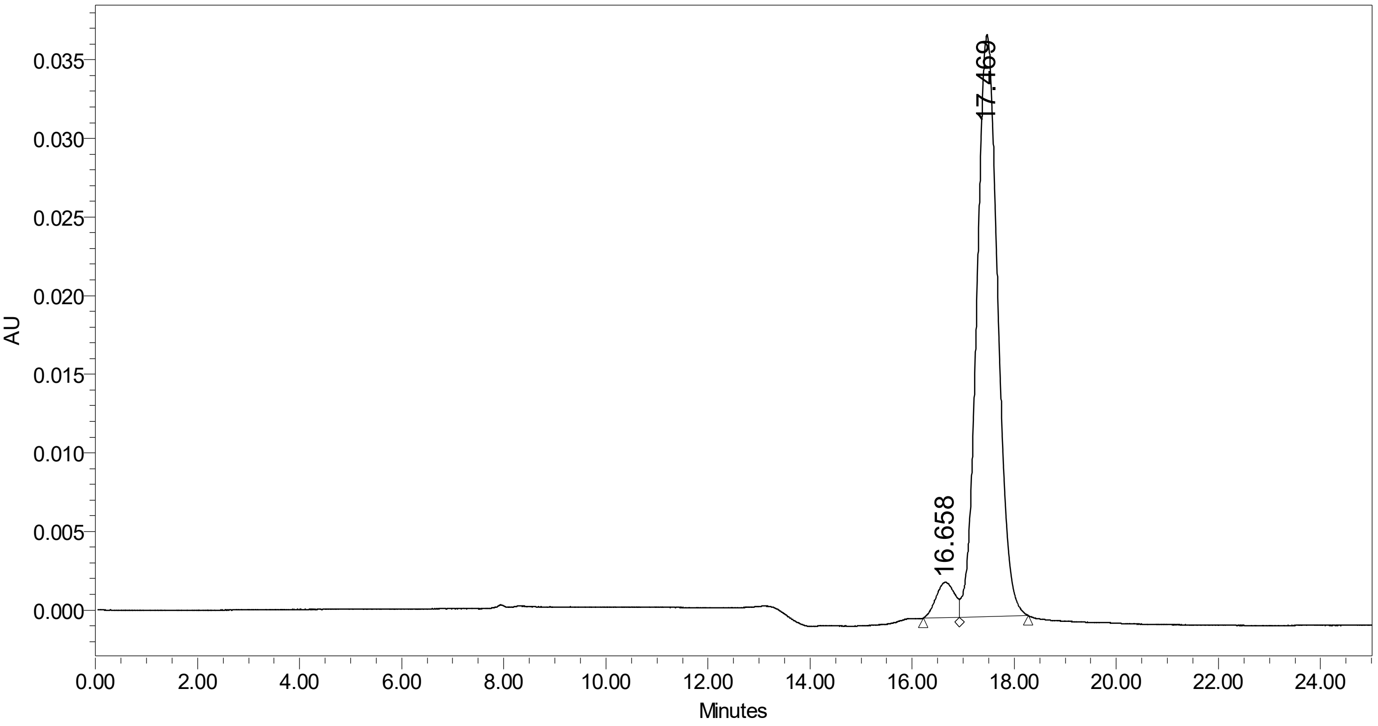

	RT	Area	% Area	Height
1	16.658	57029	5.12	2235
2	17.469	1055737	94.88	37010

rt 72 h (*E*) isomer

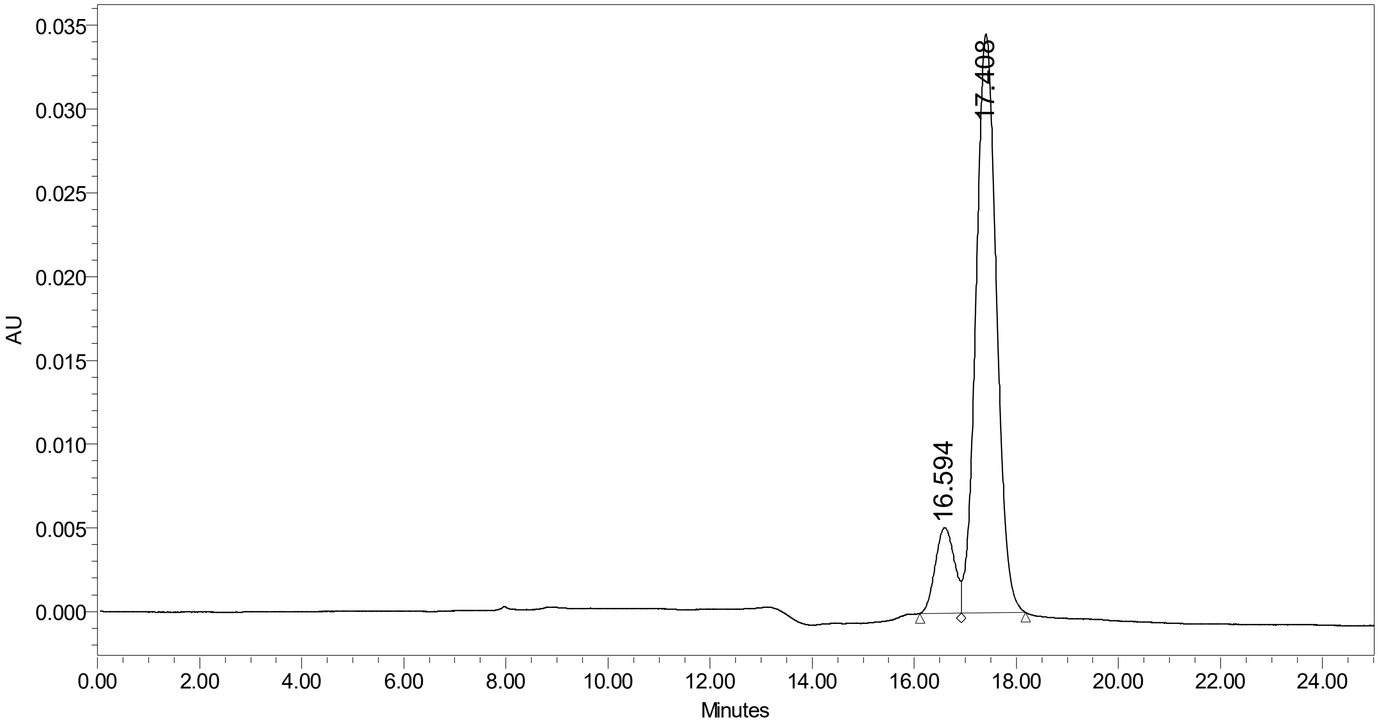

	RT	Area	% Area	Height
1	16.594	133919	12.15	5114
2	17.408	968273	87.85	34540

0 h

(*Z*) isomer

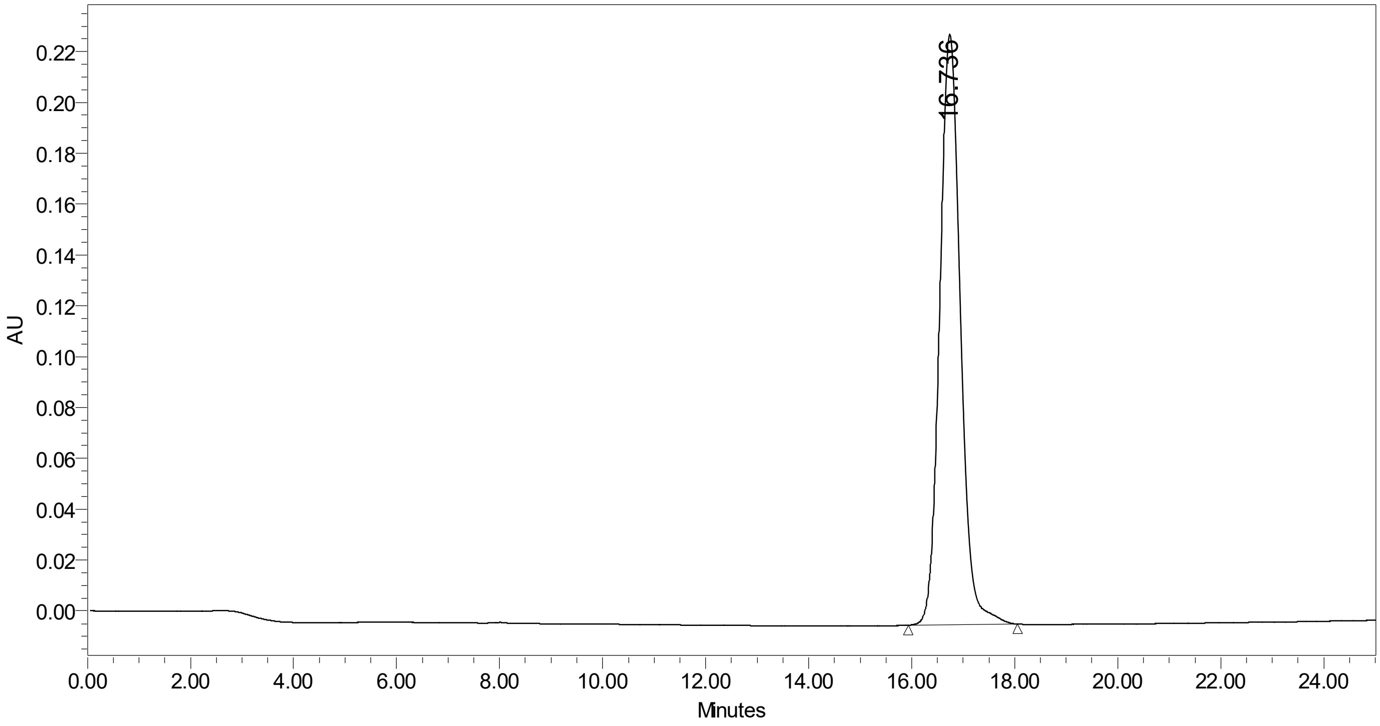

	RT	Area	% Area	Height
1	16.736	6475695	100.00	232252

4℃ 24 h (*Z*) isomer

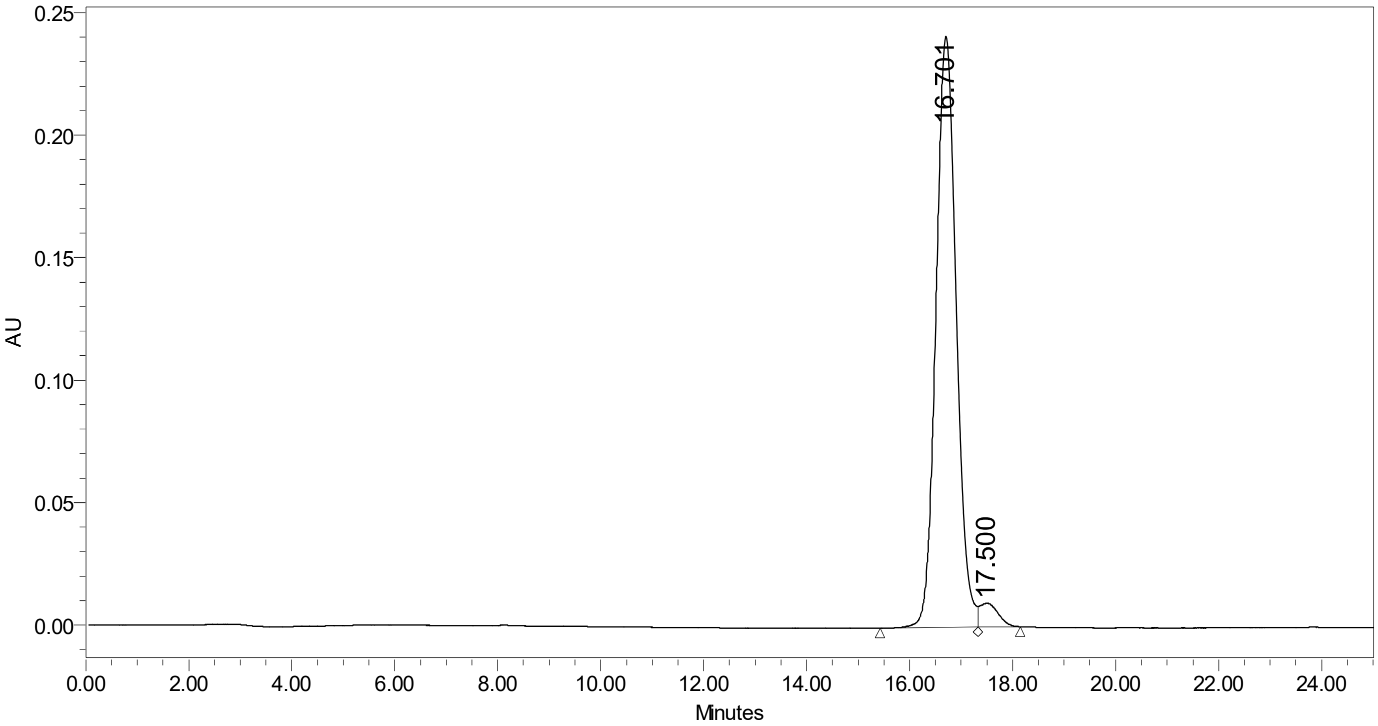

	RT	Area	% Area	Height
1	16.701	6581853	96.46	241196
2	17.500	241350	3.54	9665

4℃ 72 h (*Z*) isomer

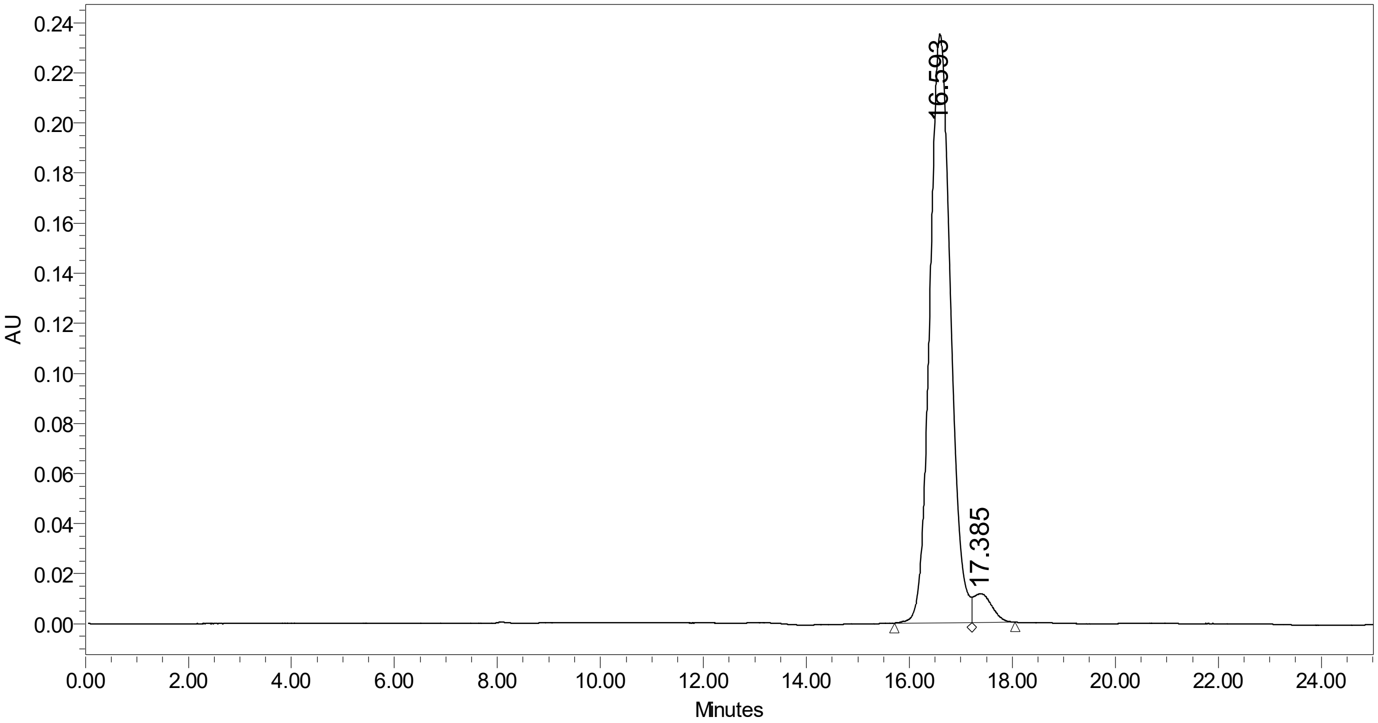

	RT	Area	% Area	Height
1	16.593	6547214	95.80	235078
2	17.385	287240	4.20	11498

50℃ 1 h (*Z*) isomer

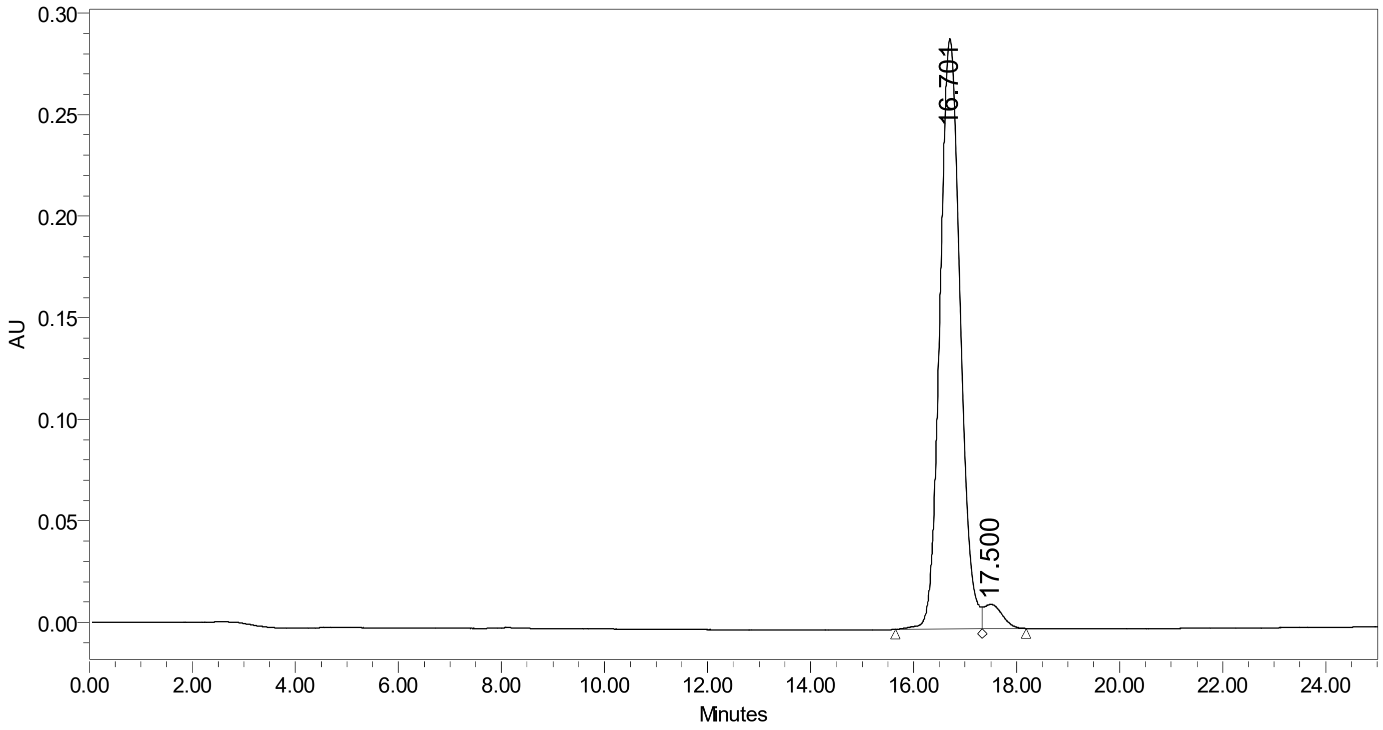

	RT	Area	% Area	Height
1	16.701	7953763	96.37	290617
2	17.500	299262	3.63	12004

50℃ 2 h (*Z*) isomer

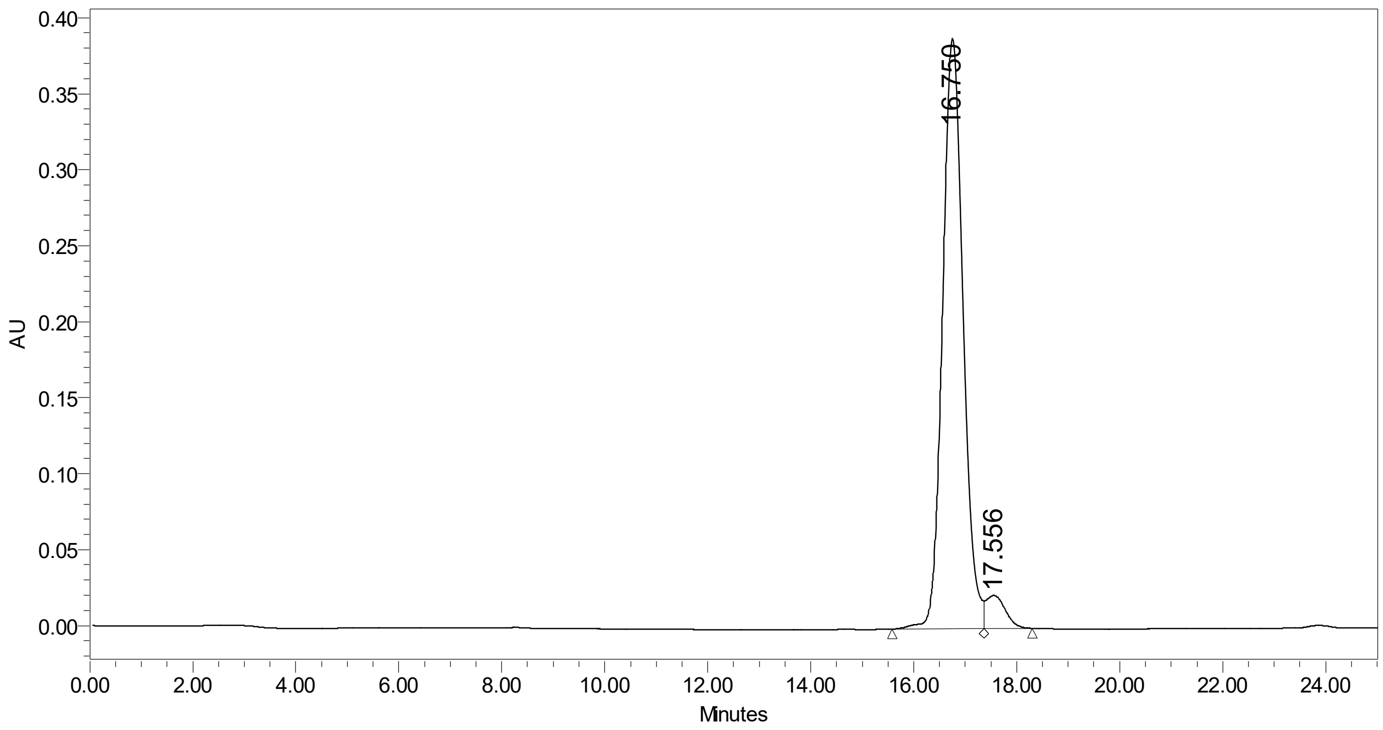

	RT	Area	% Area	Height
1	16.750	10786976	94.94	388332
2	17.556	574937	5.06	21819

50℃ 7 h

(*Z*) isomer

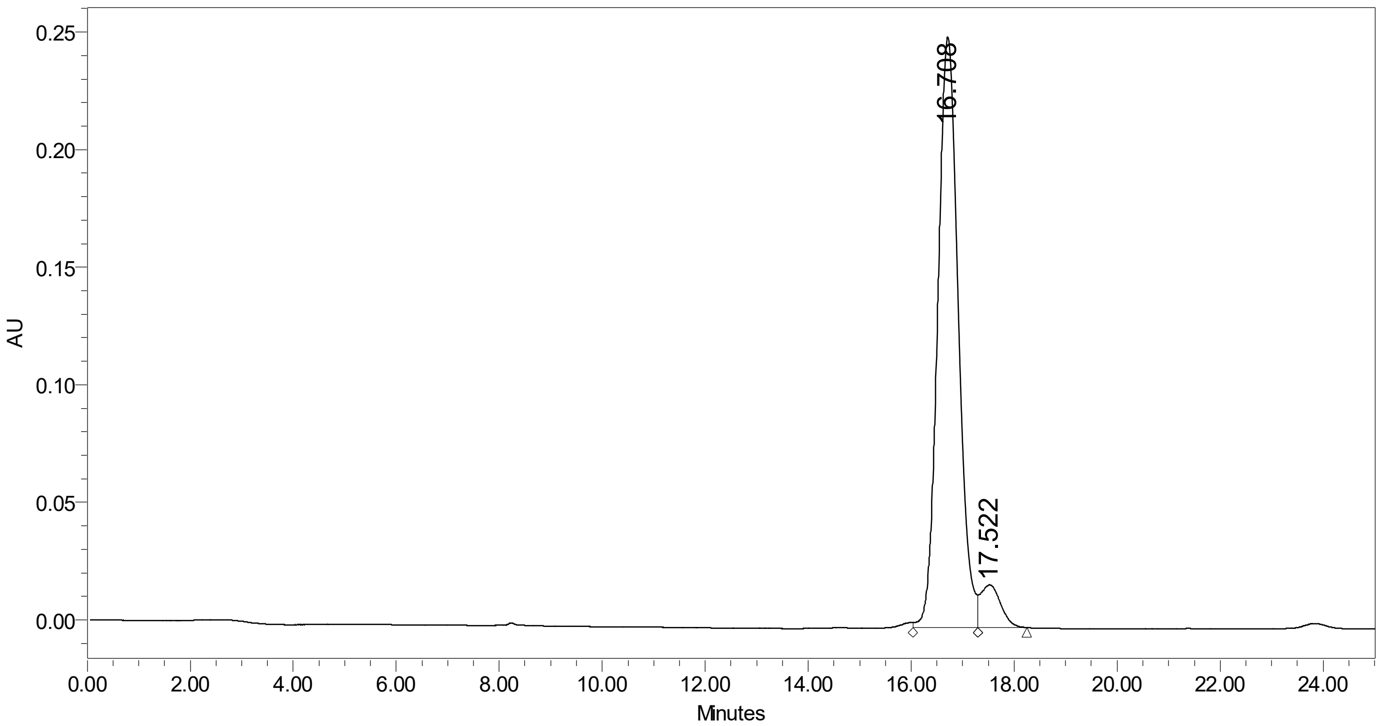

	RT	Area	% Area	Height
1	16.708	6936466	93.36	250975
2	17.522	493398	6.64	18146

rt 24 h (*Z*) isomer

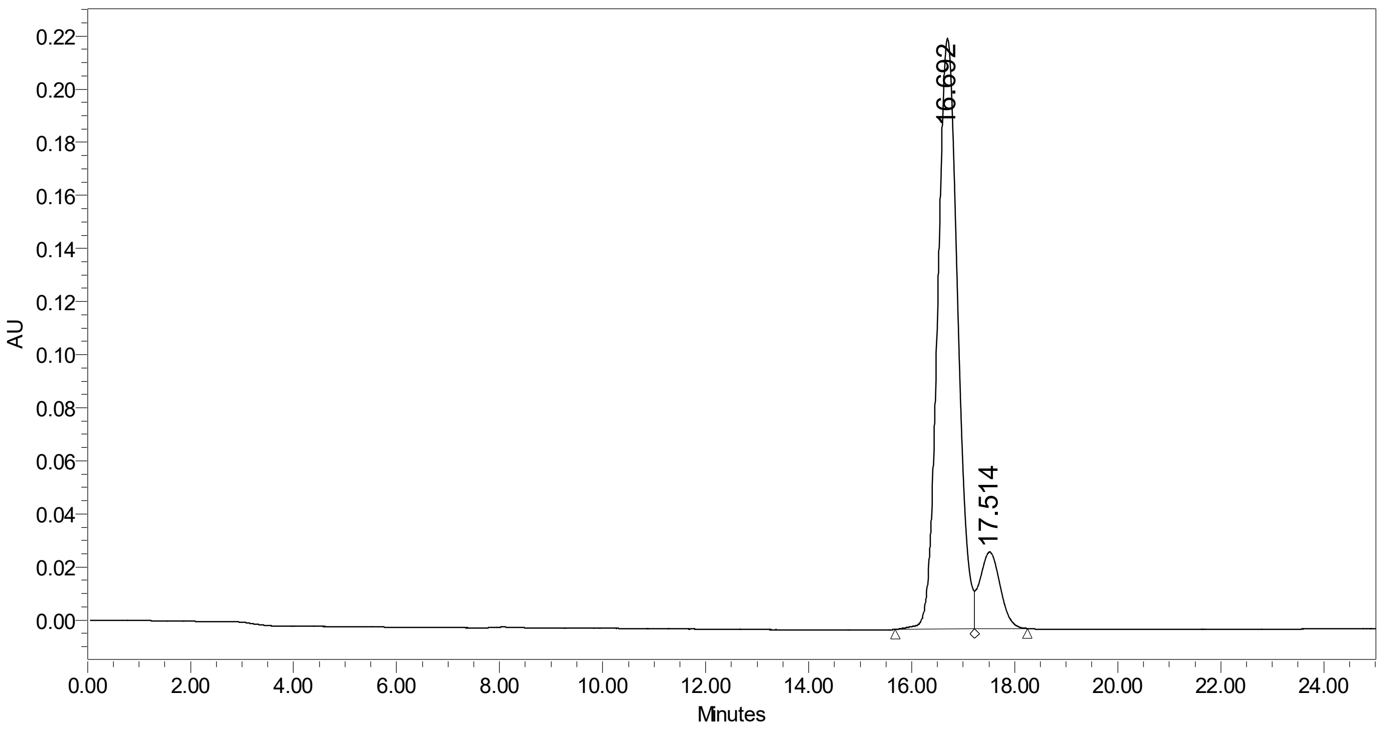

	RT	Area	% Area	Height
1	16.692	6025255	88.28	222502
2	17.514	800266	11.72	28907

rt 72 h (*Z*) isomer

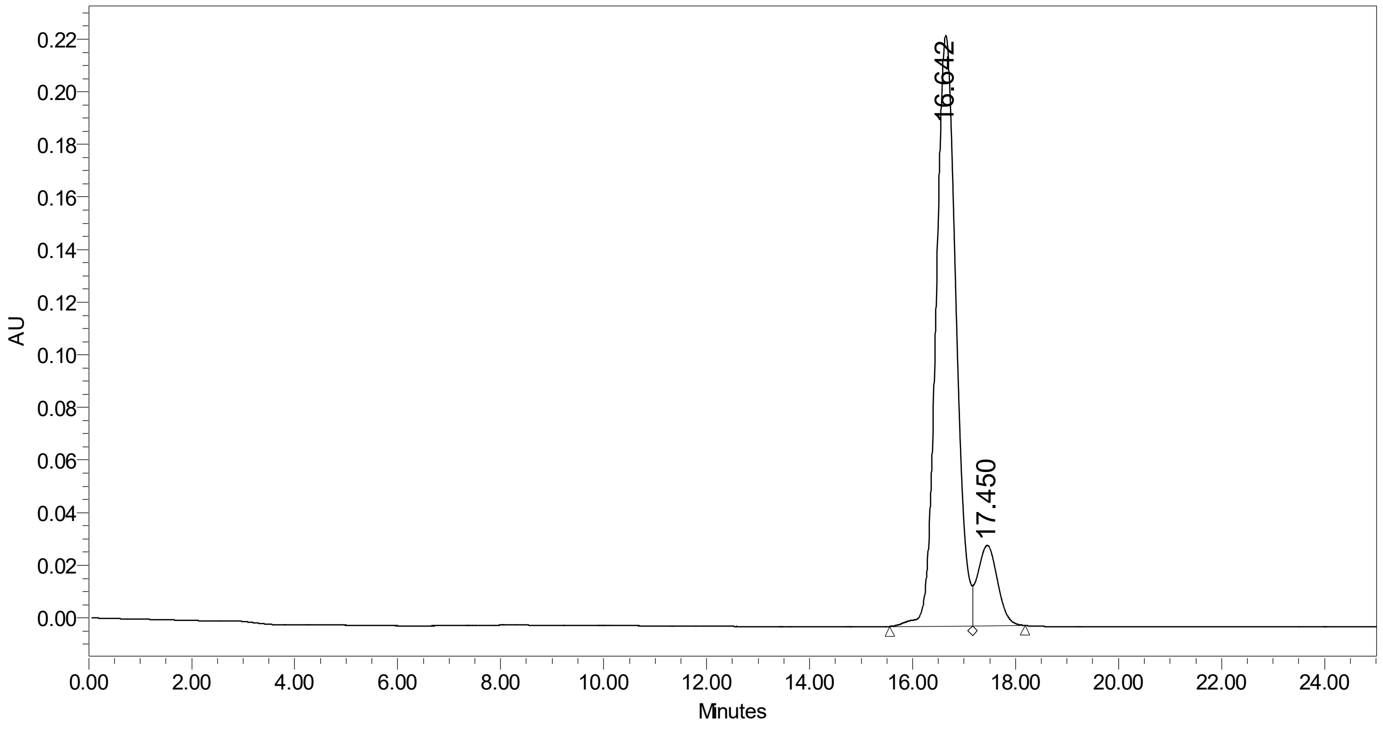

	RT	Area	% Area	Height
1	16.642	6024498	87.88	224468
2	17.450	831051	12.12	30590

The stability of **H6** under different conditions

Configuration	0 h	50 ℃ 1 h	50 ℃ 2 h	50 ℃ 7 h	rt 24 h	rt 72h	4 ℃ 24 h	4 ℃ 72 h
(*Z*) isomer (%)	100	96.37	94.94	93.36	88.28	87.88	96.46	95.8
(*E*) isomer (%)	100	100	97.83	95.12	94.88	87.85	100	98.32
