# Supplementary material for: Alkenyl oxindole is a novel PROTAC moiety that recruits the CRL4DCAF11 E3 ubiquitin ligase complex for targeted protein degradation
Source: PLoS Biol. 2024 May 20;22(5):e3002550. doi: 10.1371/journal.pbio.3002550 (PMC11104598; doi:10.1371/journal.pbio.3002550)
Supplement: S1 Raw Images — (PDF) [file pbio.3002550.s014.pdf]

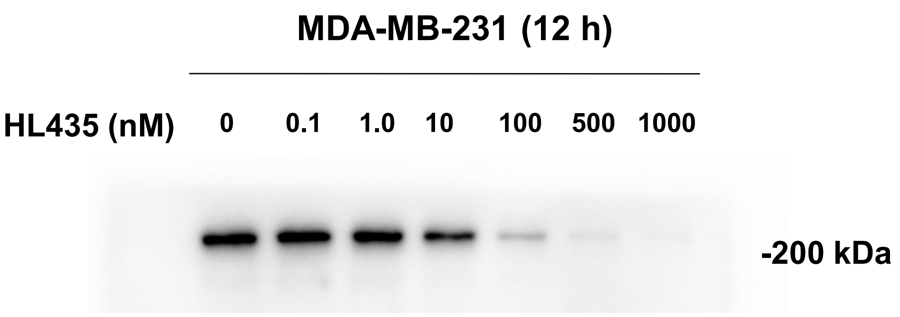

Figure 2B BRD4

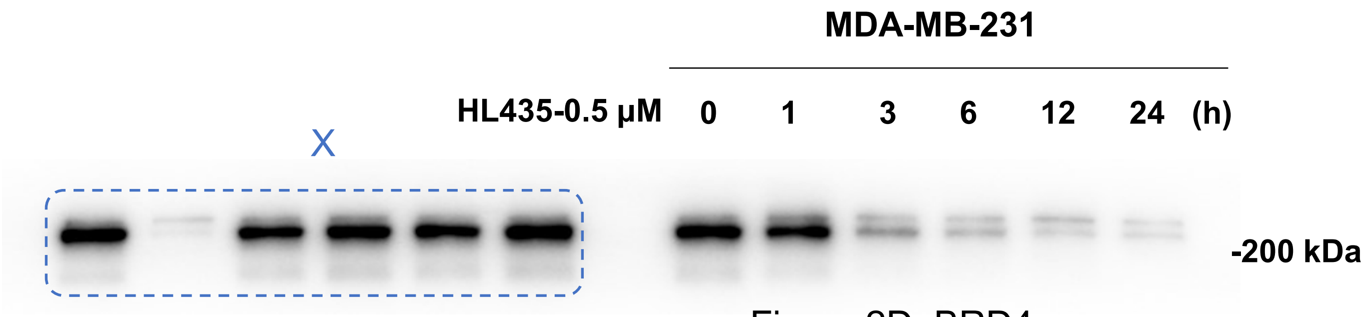

Figure 2D BRD4

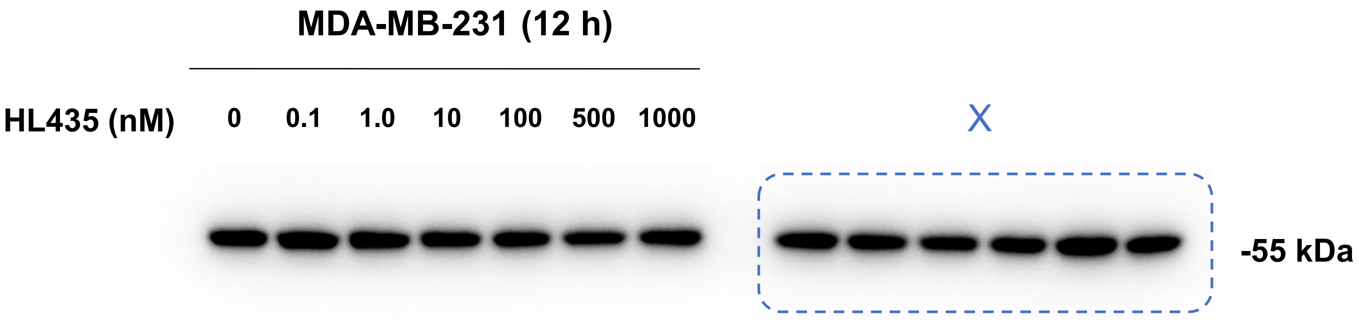

Figure 2B α-Tubulin

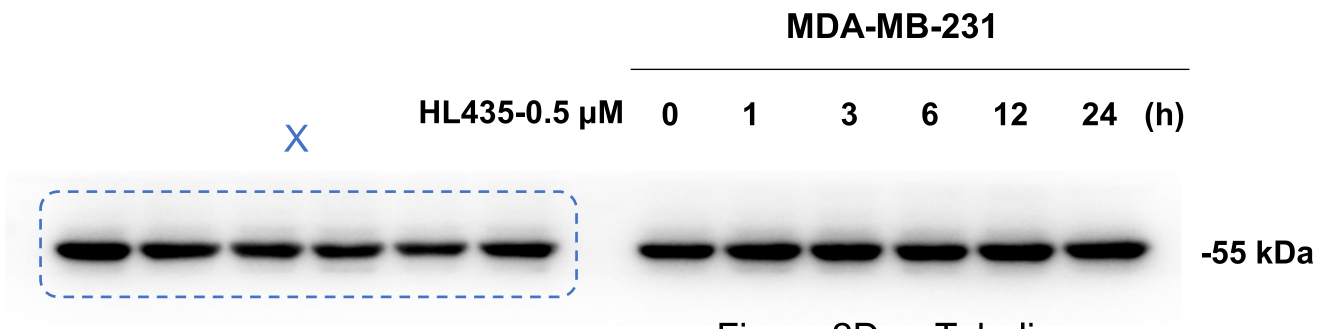

Figure 2D α-Tubulin

X stands for irrelevant or unpresented lanes in figure

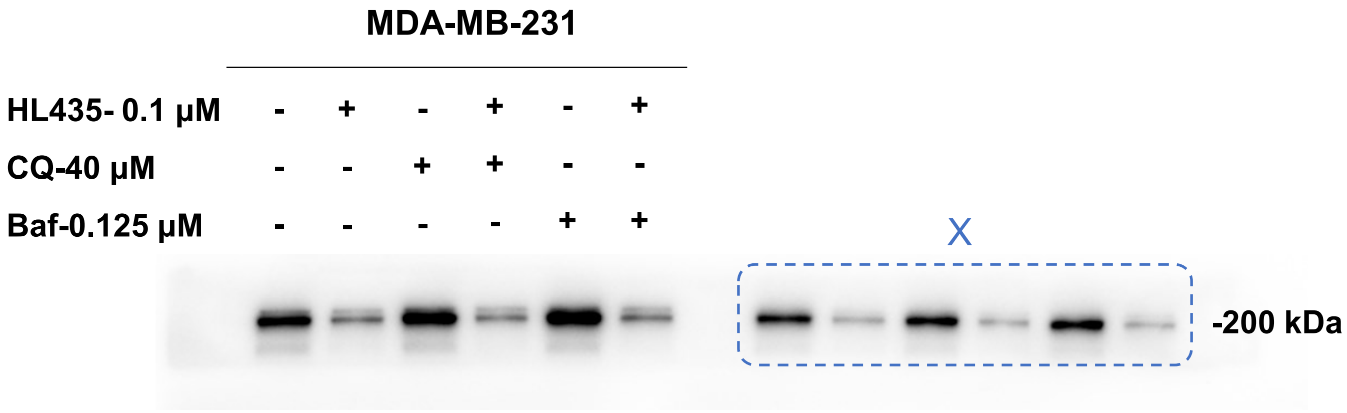

Figure 2G BRD4

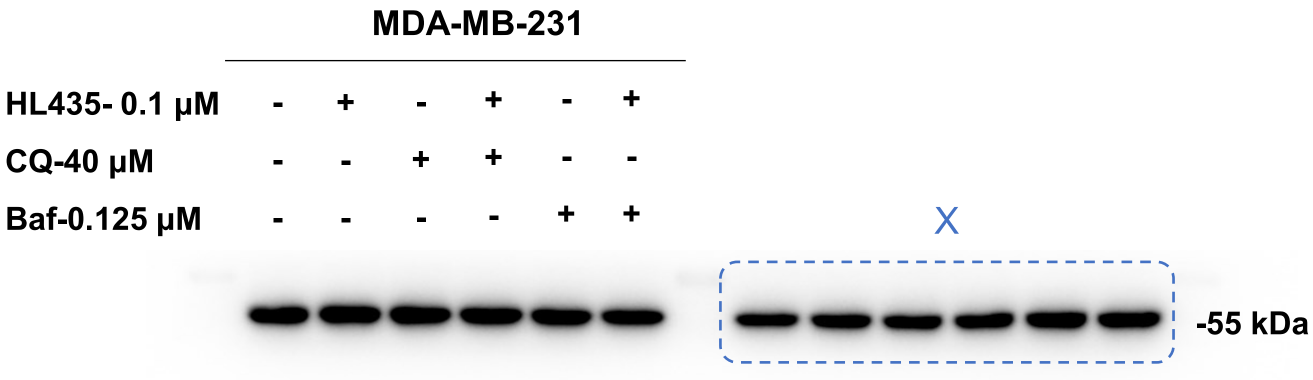

Figure 2G α-Tubulin

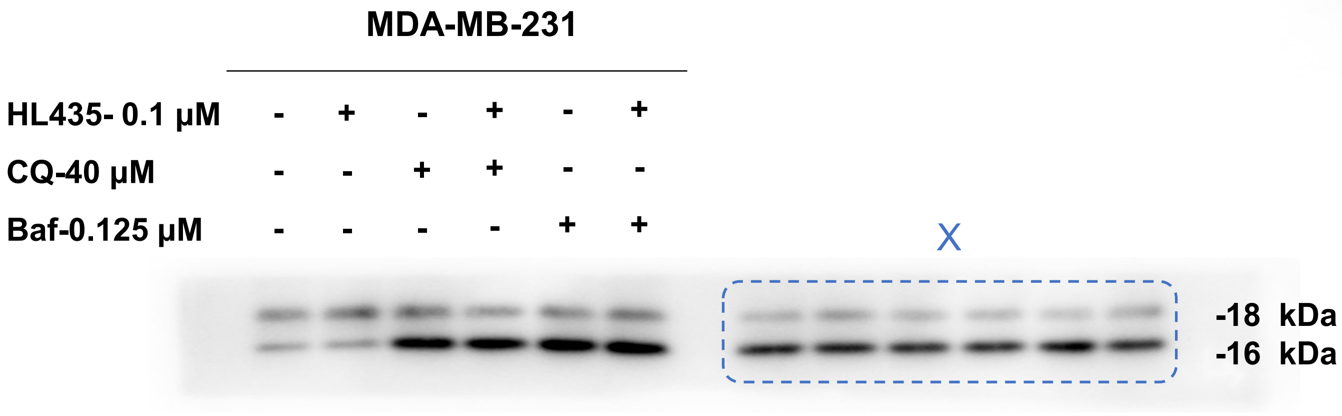

Figure 2G LC3

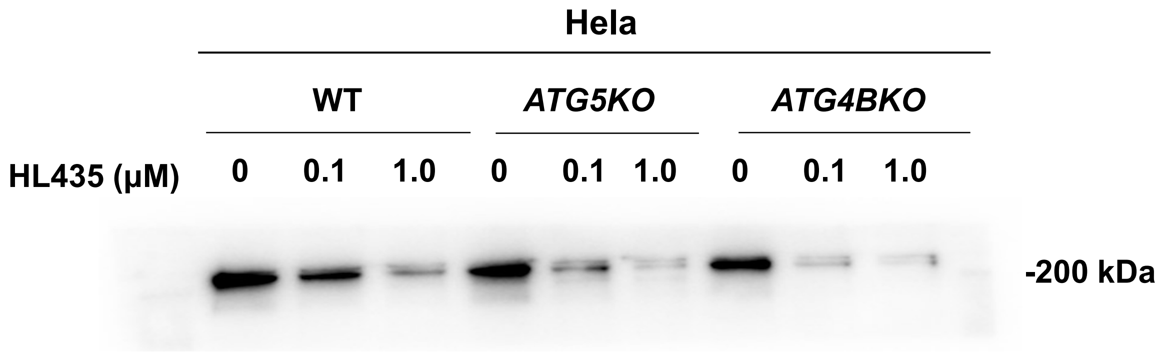

Figure 2I BRD4

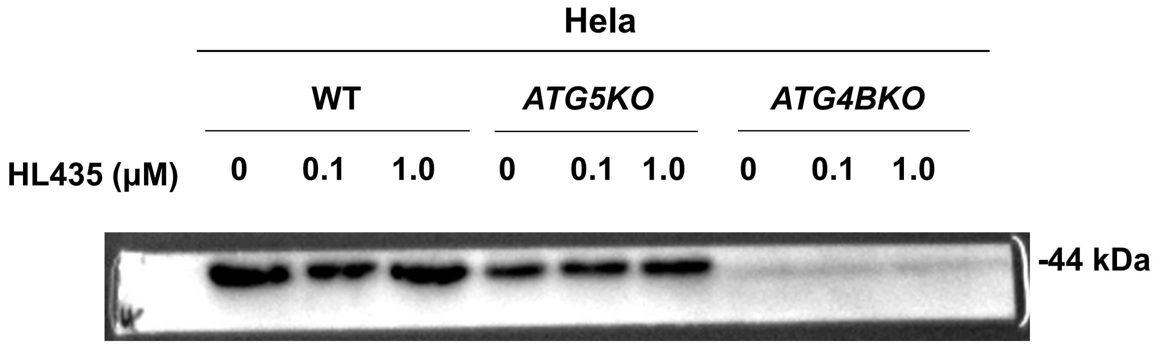

Figure 2I ATG4B

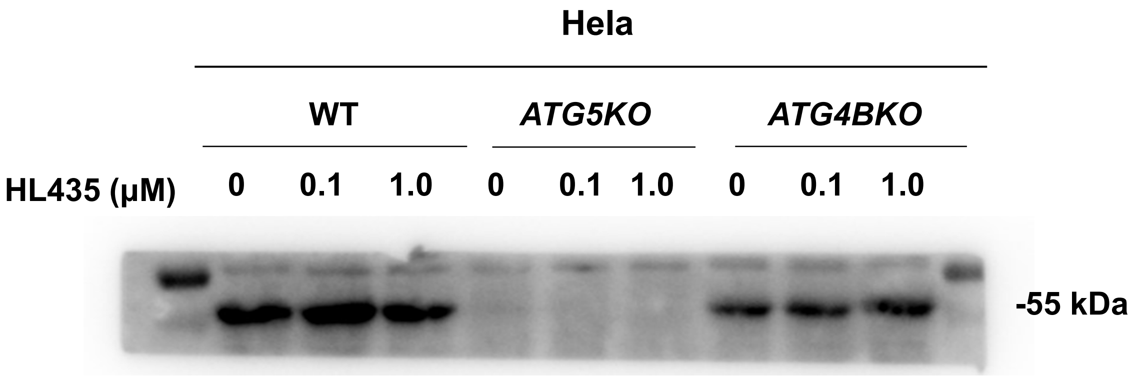

Figure 2I ATG5

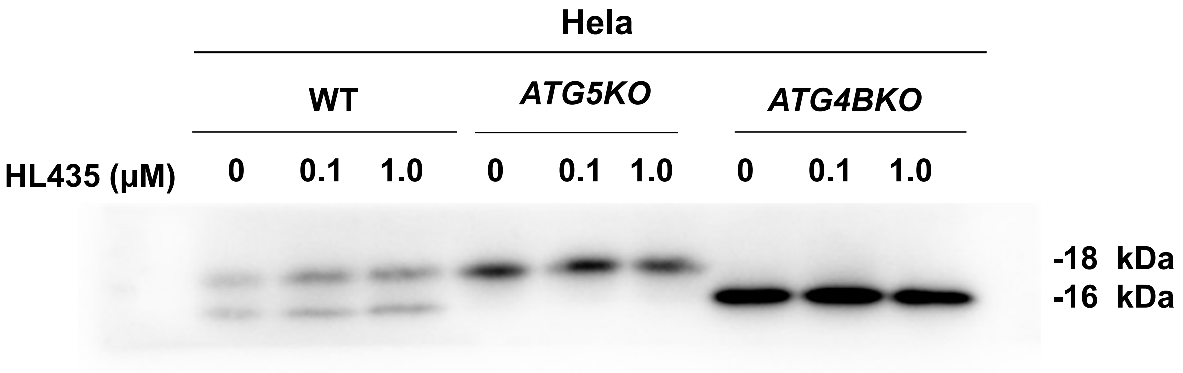

Figure 2I LC3

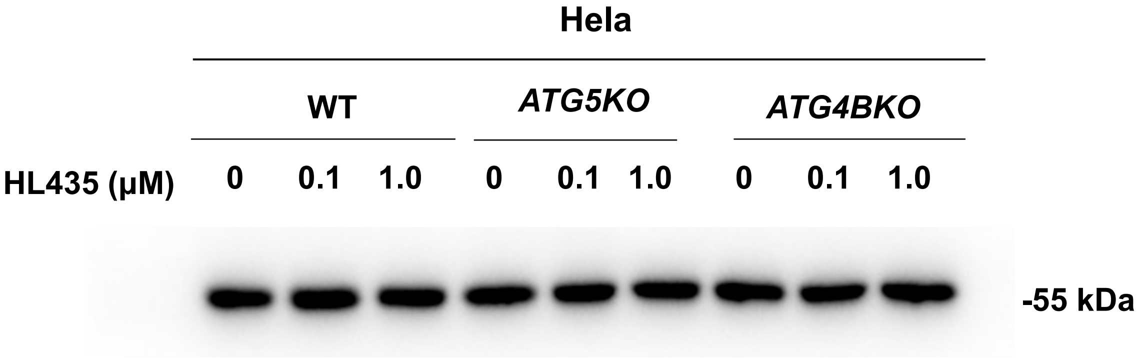

Figure 2I α-Tubulin

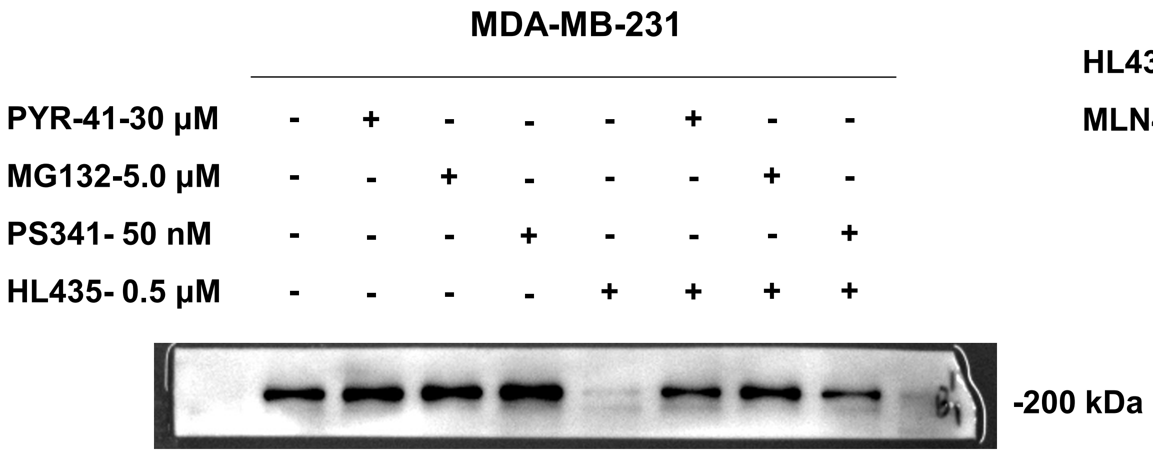

Figure 2J BRD4

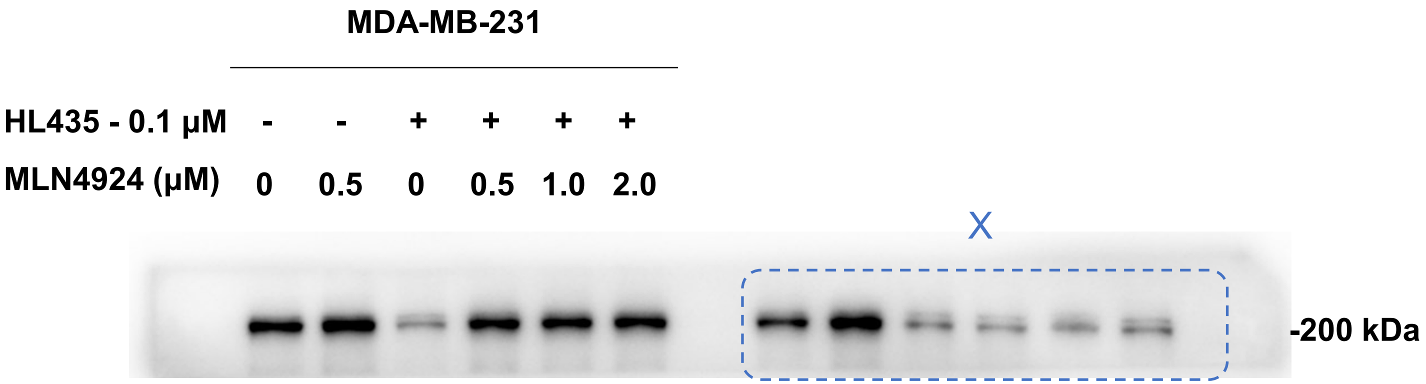

Figure 2L BRD4

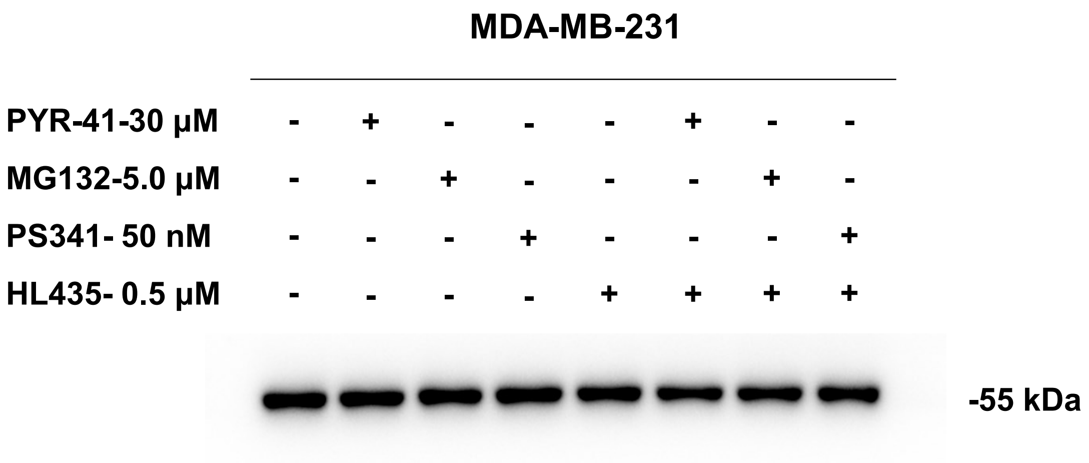

Figure 2J α-Tubulin

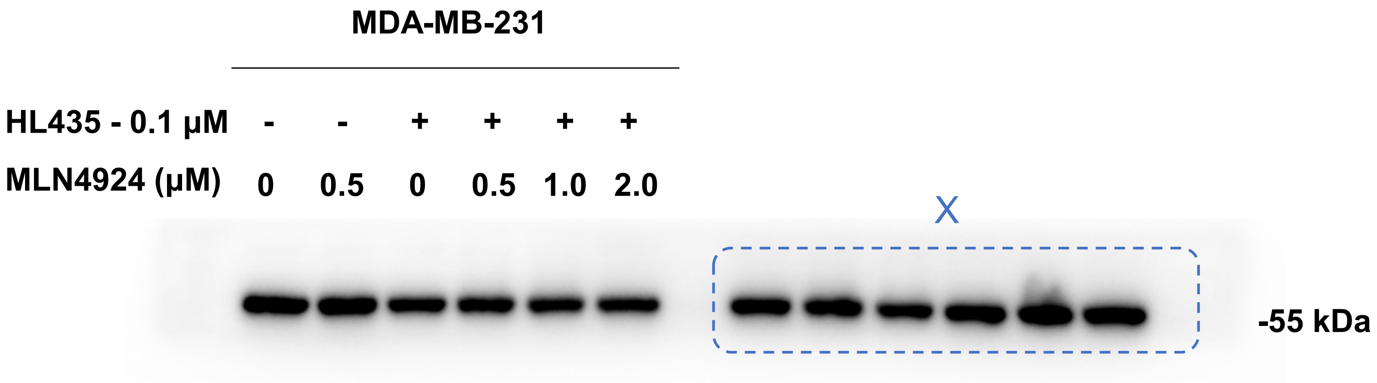

Figure 2L α-Tubulin

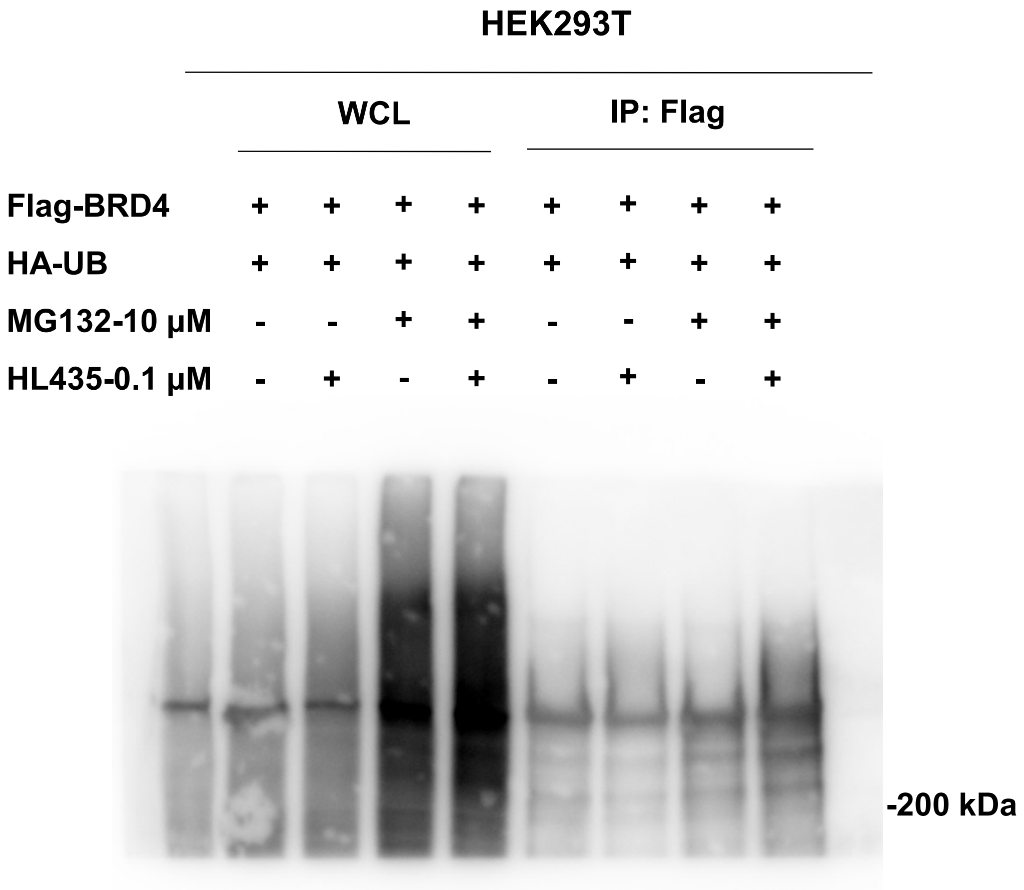

Figure 2M Ub

For the detection of ubiquitination level, 3% Tris-Acetate Polyacrylamide Gradient Gels were used to separate protein samples.

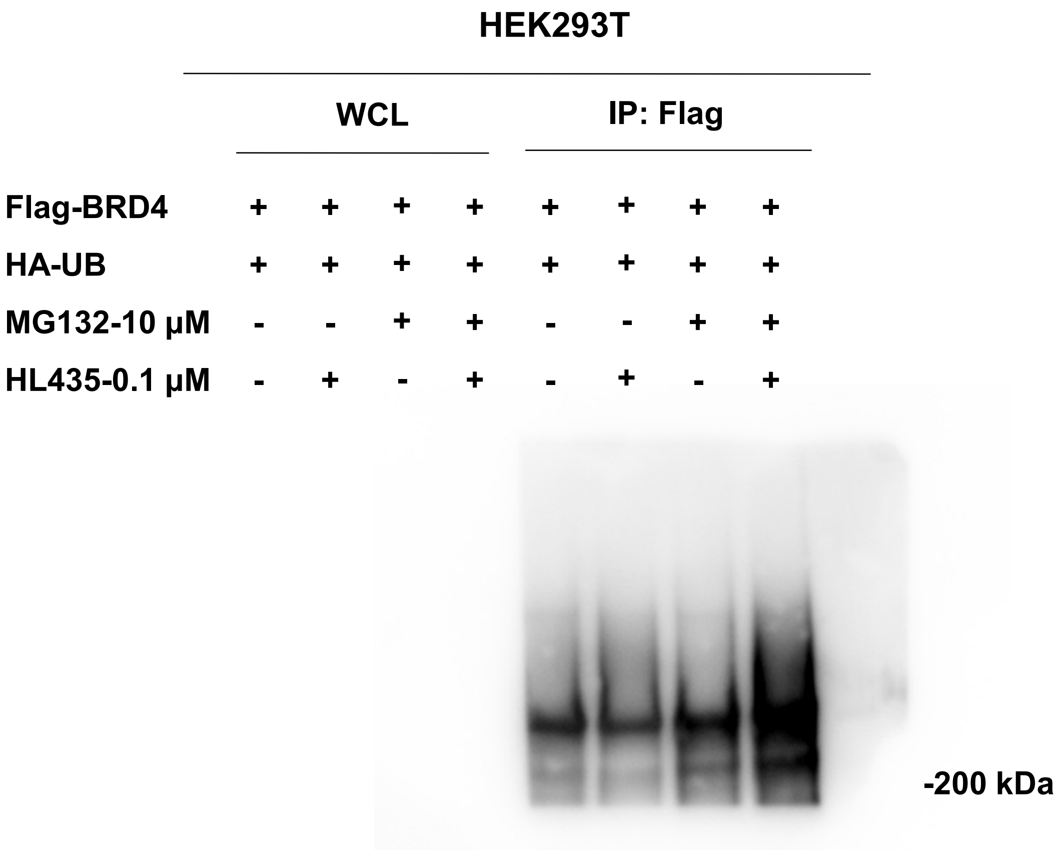

Figure 2M Ub（IP）

For the detection of ubiquitination level, 3% Tris-Acetate Polyacrylamide Gradient Gels were used to separate protein samples.

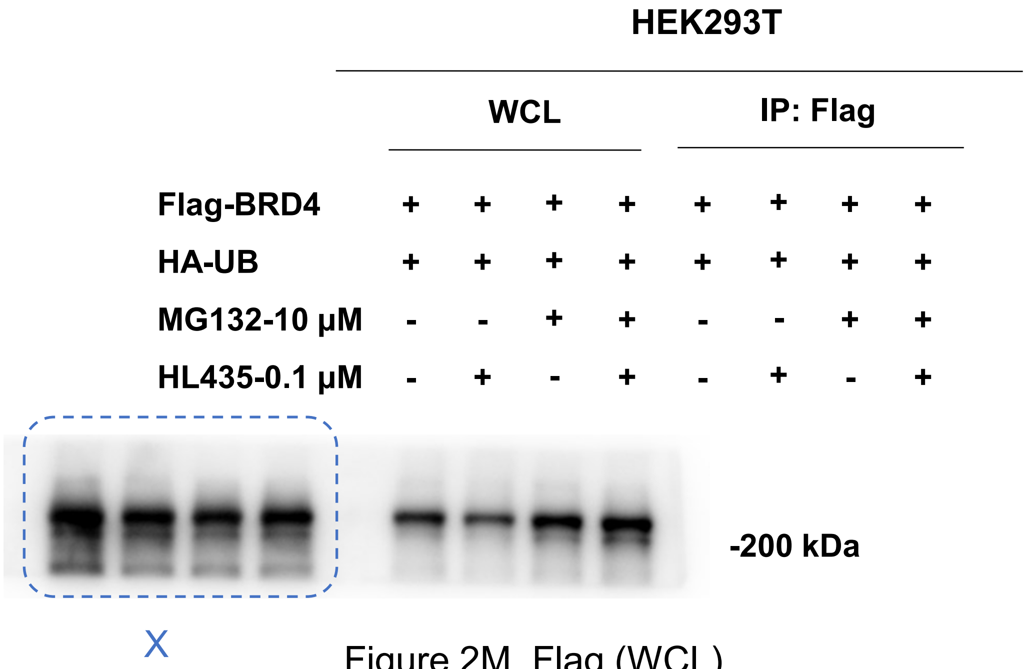

Figure 2M Flag (WCL)

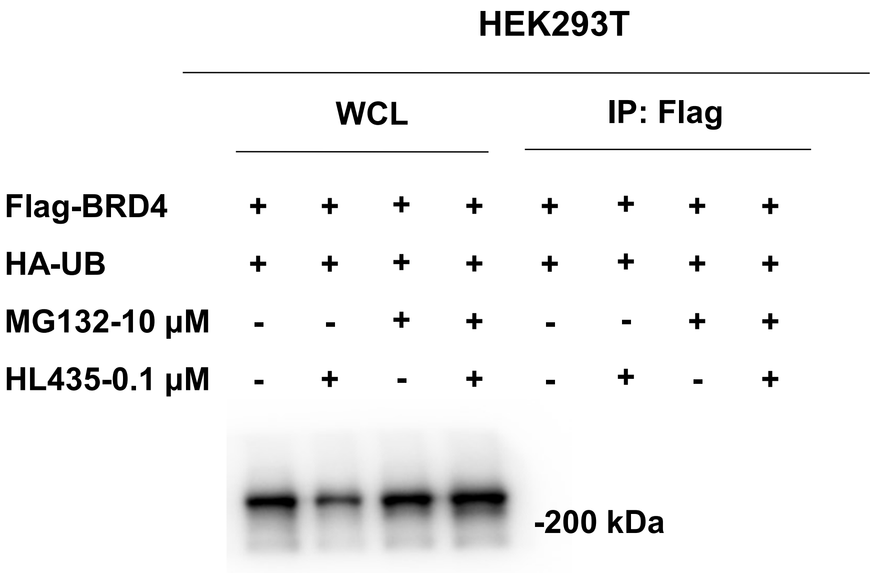

Figure 2M BRD4 (WCL)

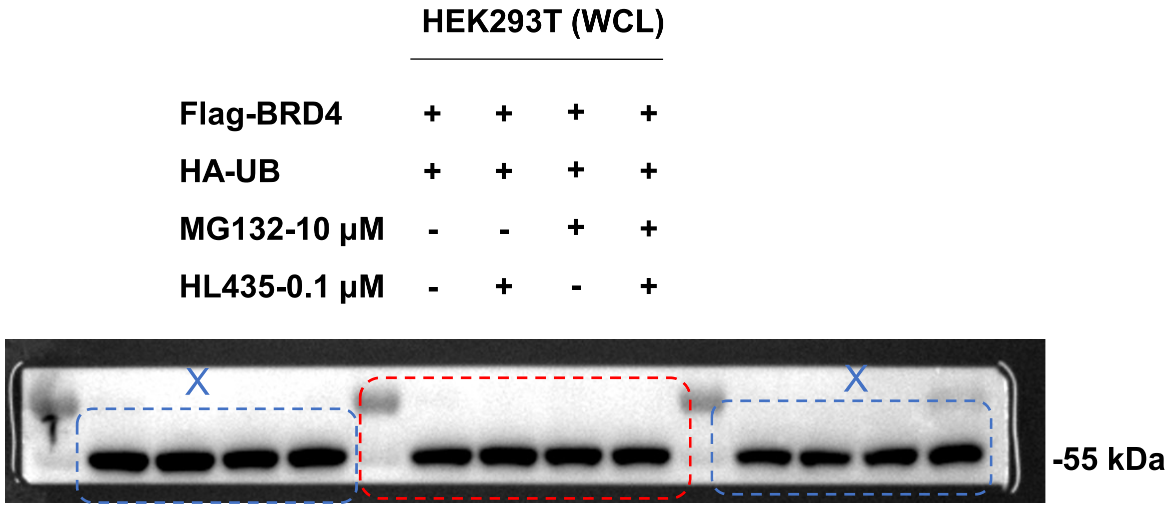

Figure 2M α-Tubulin (WCL)

MDA-MB-231

HL435-0.1 μM	-	+	-	-	-	+
JQ1 - 5.0 μM	-	-	+	-	+	+
HL389-5.0 μM	-	-	-	+	+	-

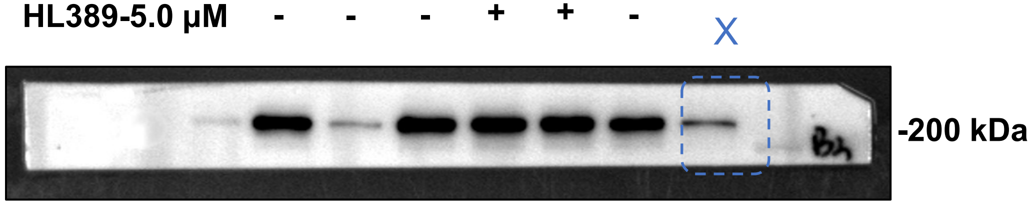

Figure 2N BRD4

MDA-MB-231

HL435-0.1 μM	-	+	-	-	-	+
JQ1 - 5.0 μM	-	-	+	-	+	+
HL389-5.0 μM	-	-	-	+	+	-

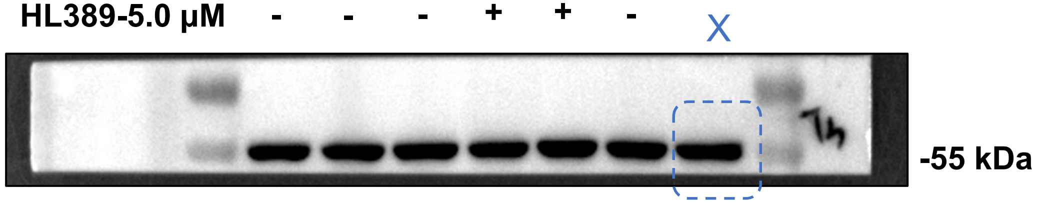

Figure 2N α-Tubulin

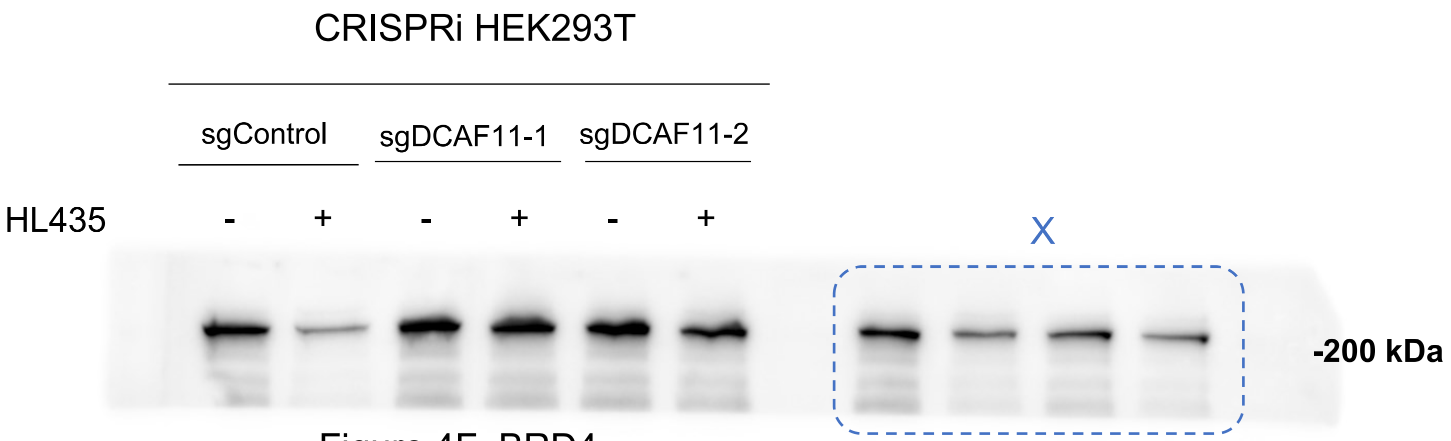

Figure 4F BRD4

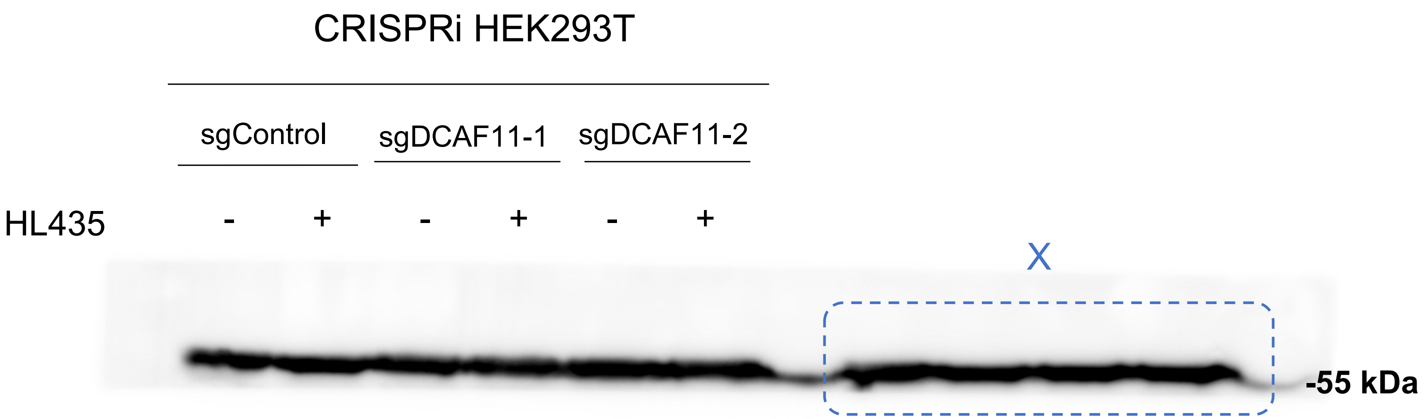

Figure 4F α-Tubulin

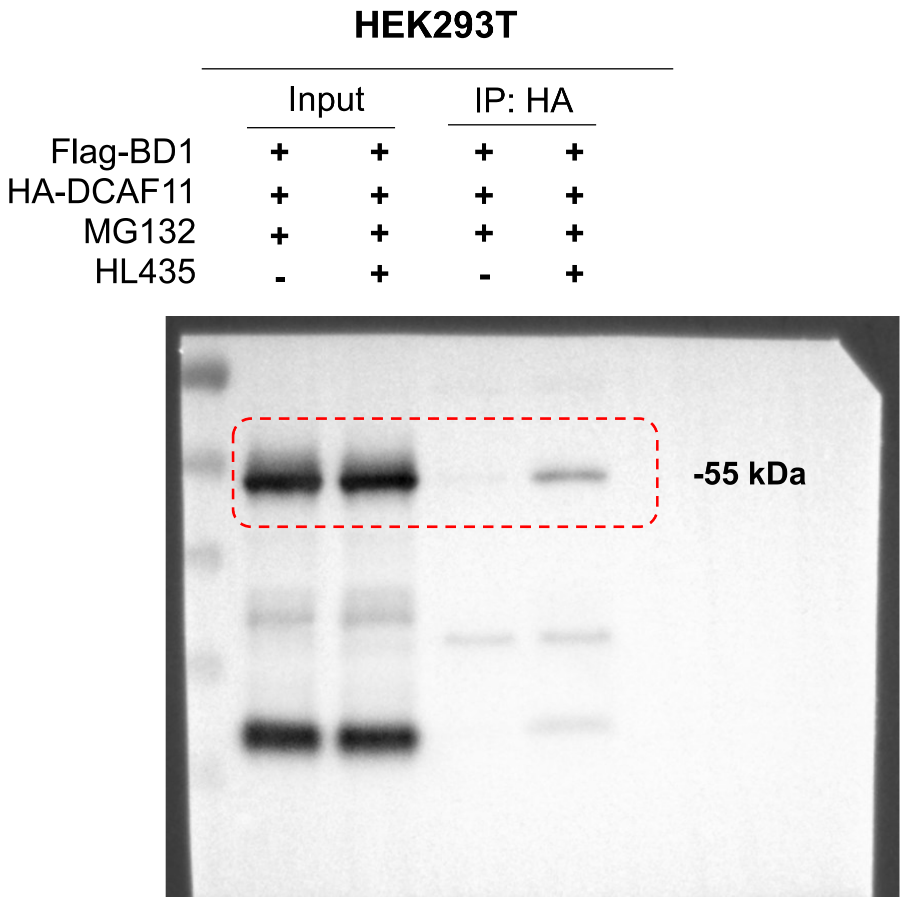

Figure 4G Flag

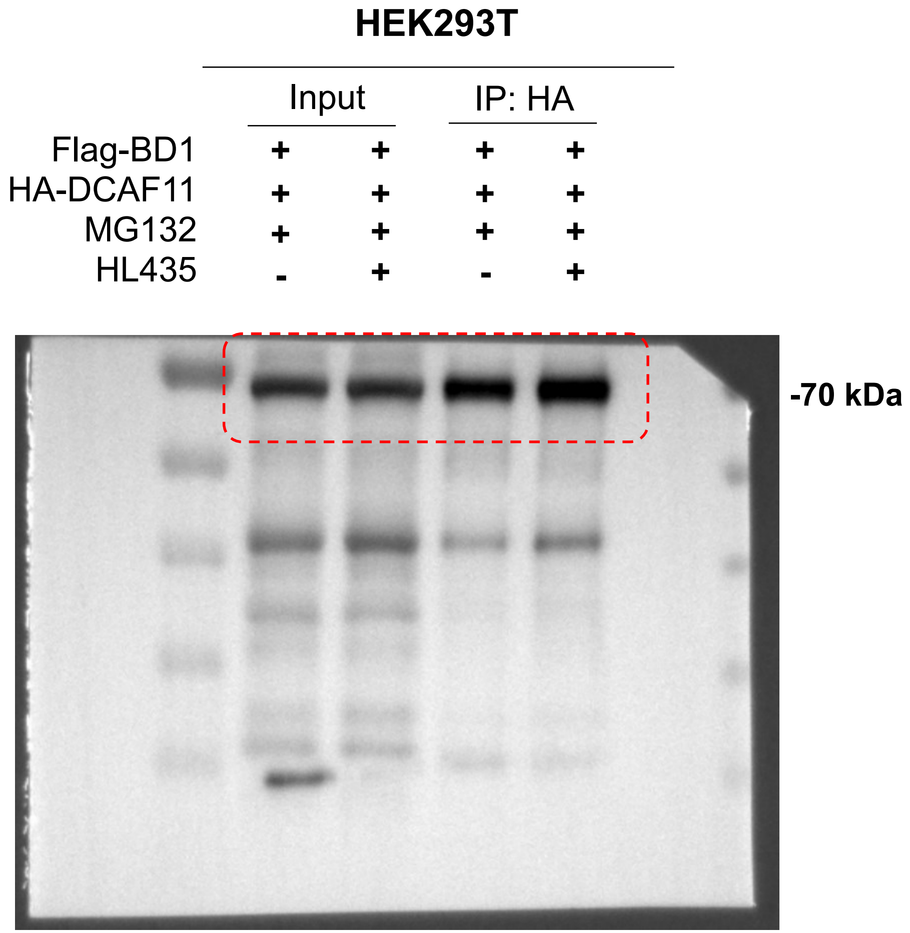

Figure 4G HA

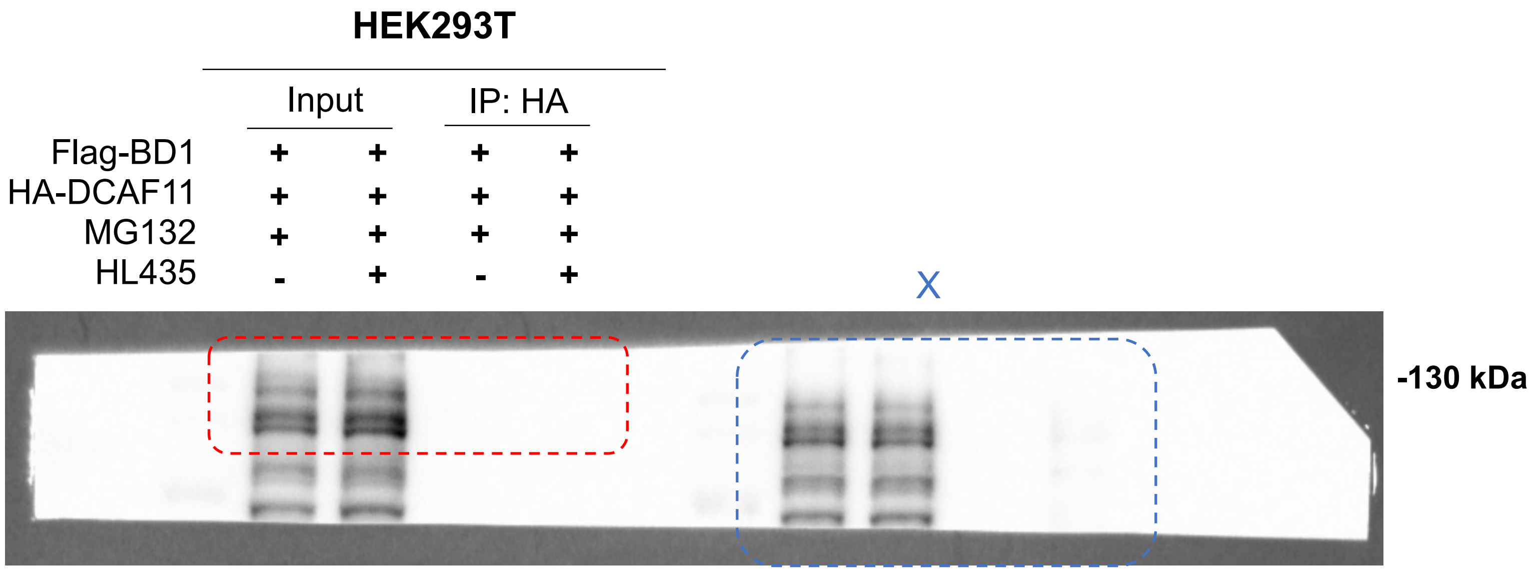

Figure 4G Vinculin

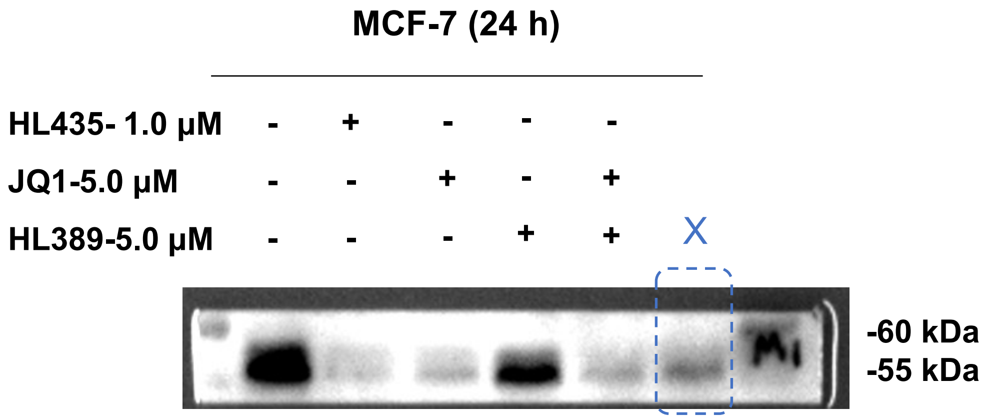

Figure 5E c-Myc

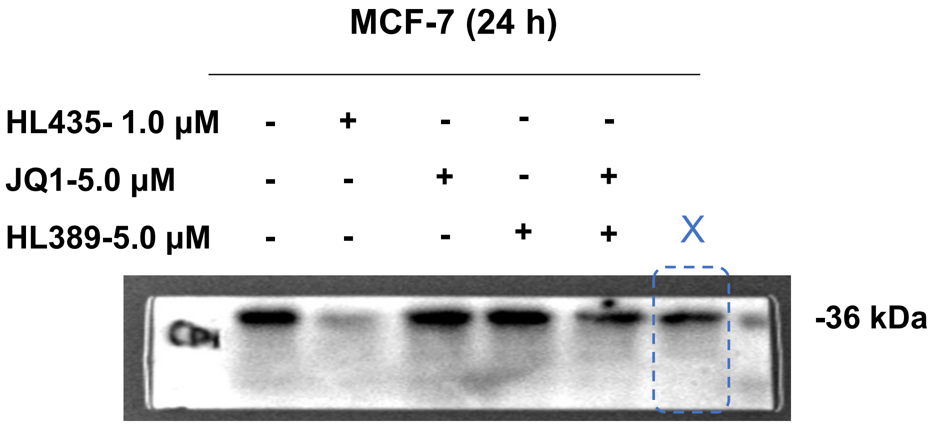

Figure 5E Cyclin D1

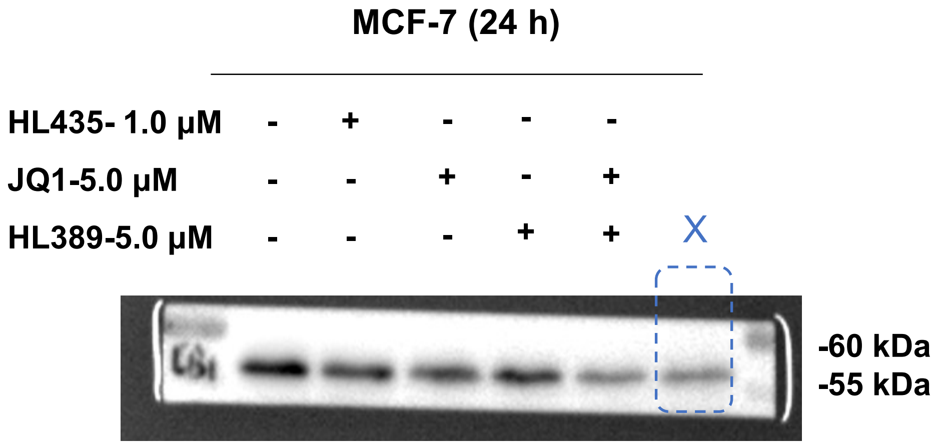

Figure 5E Cyclin B1

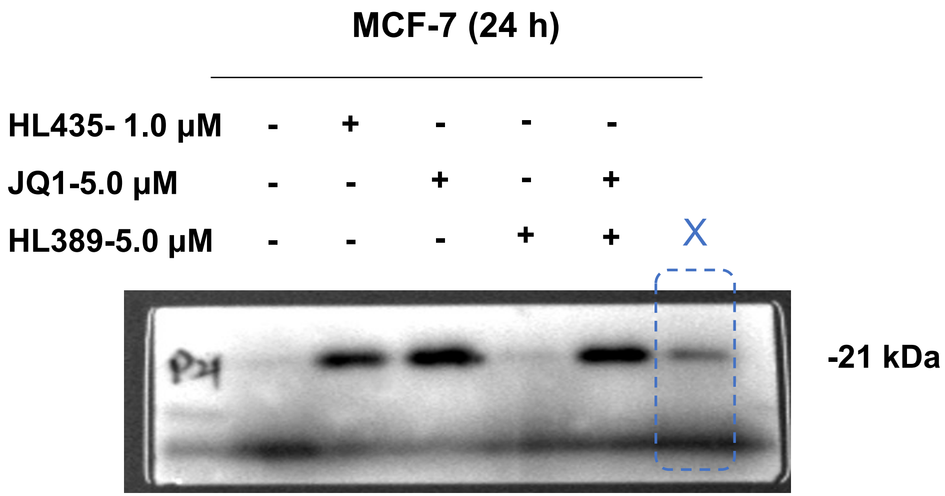

Figure 5E P21

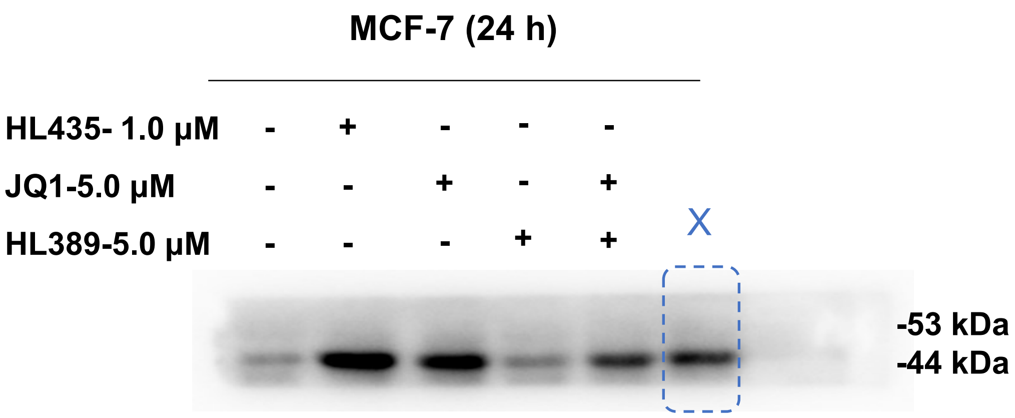

Figure 5E P53

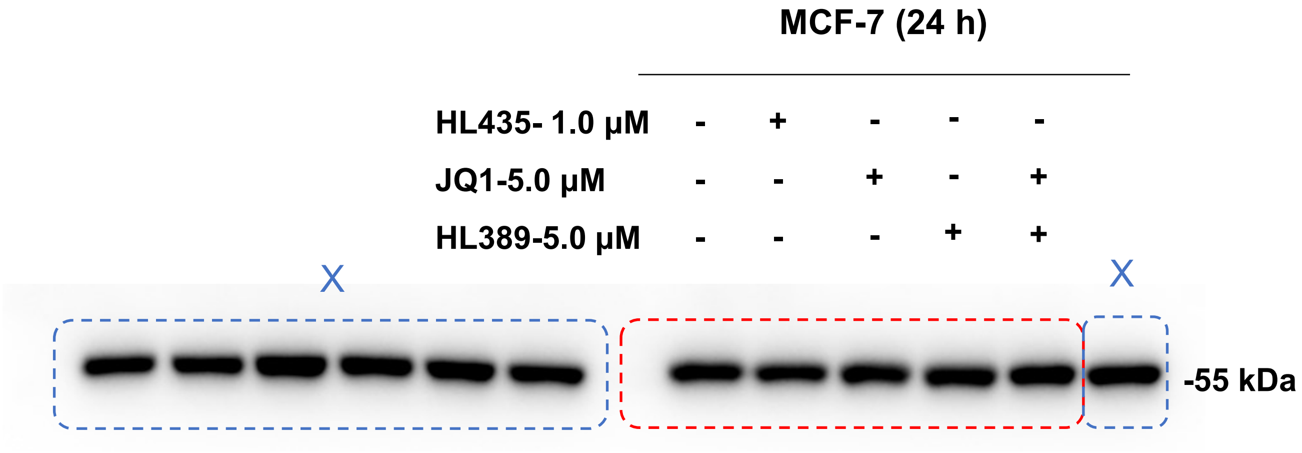

Figure 5E α-Tubulin

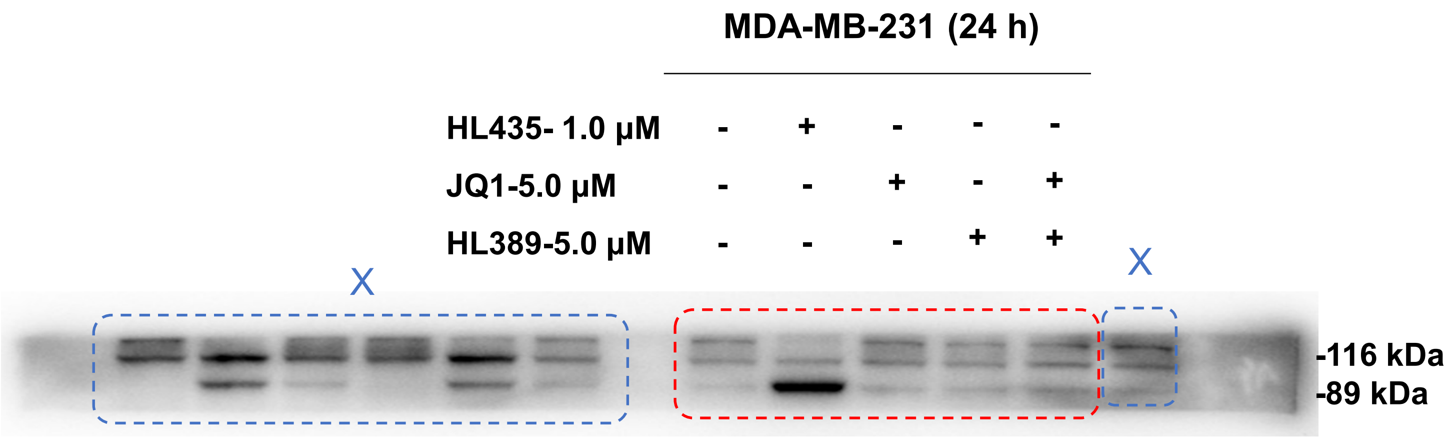

Figure 5F PARP1/Cl-PARP1

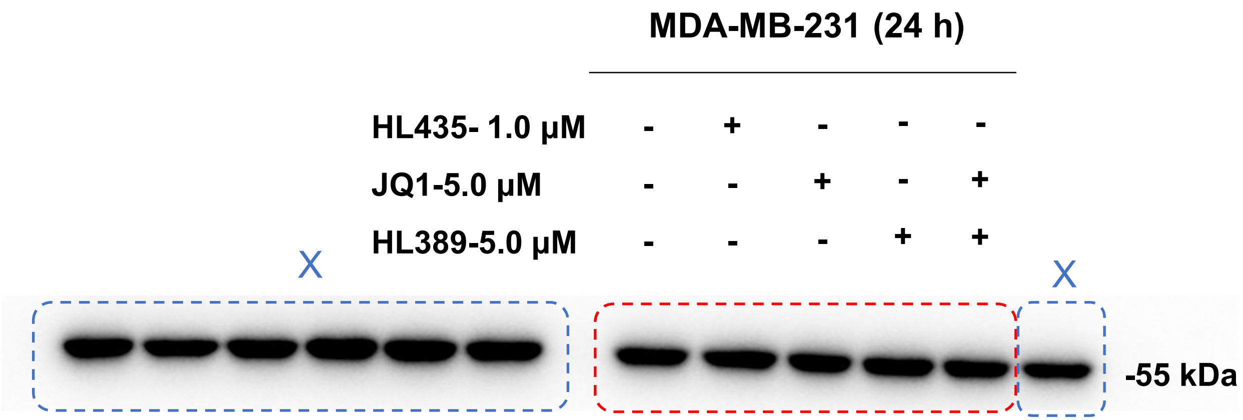

Figure 5F α-Tubulin

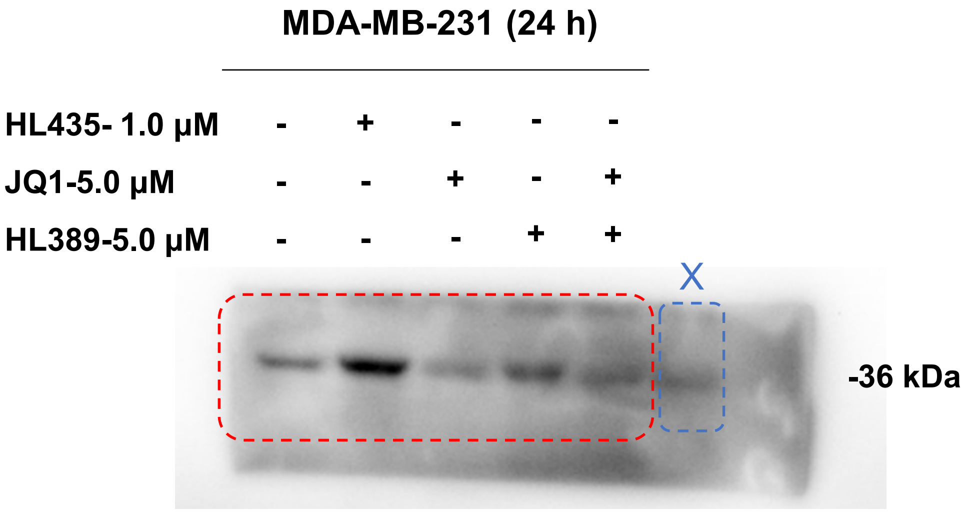

Figure 5F Cl-Caspase-9

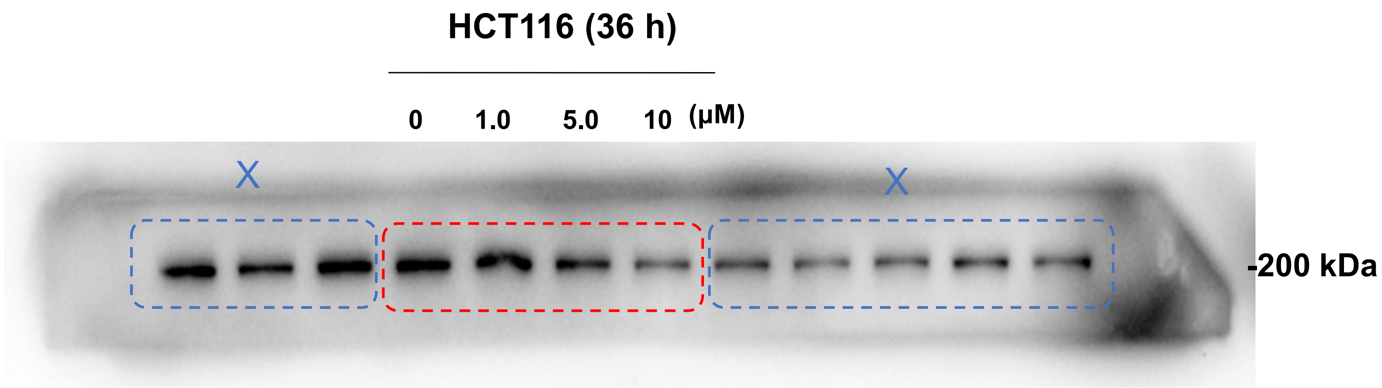

Figure S1 compound **H1** BRD4

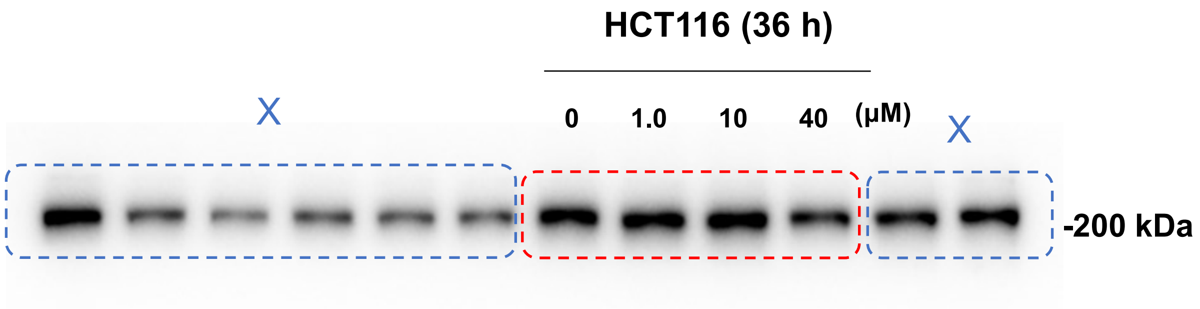

Figure S1 compound **H2** BRD4

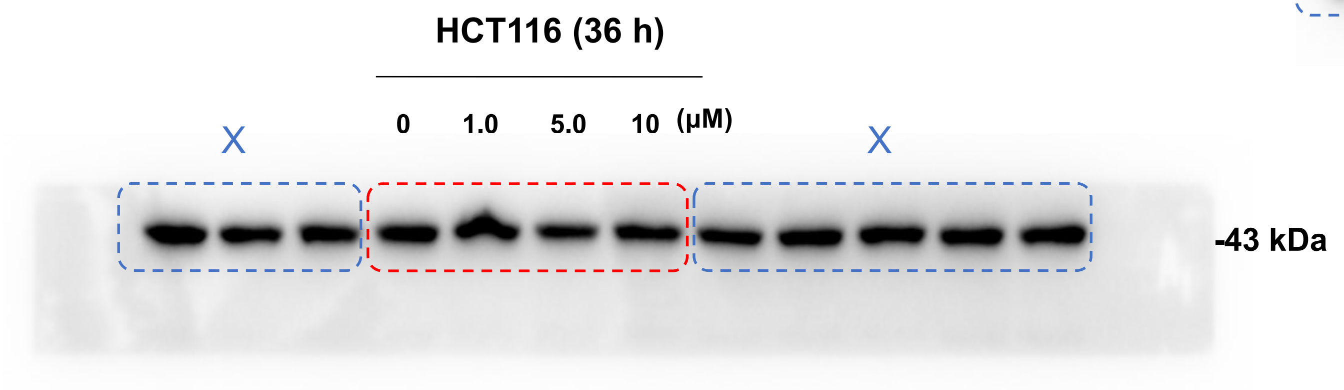

Figure S1 compound **H1** β-Actin

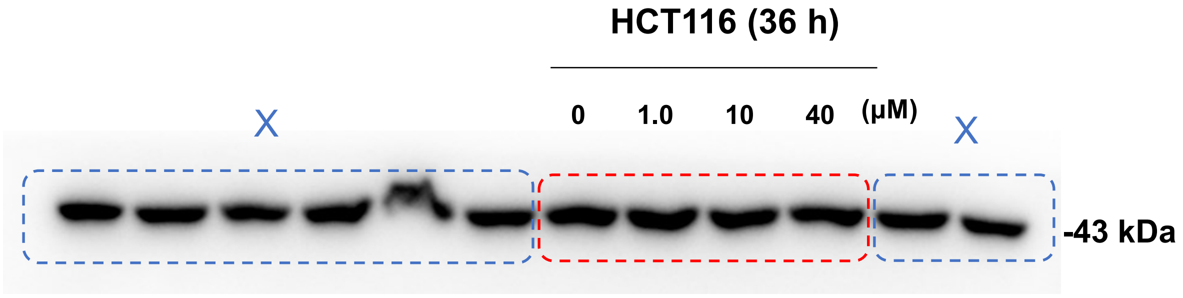

Figure S1 compound **H2** β-Actin

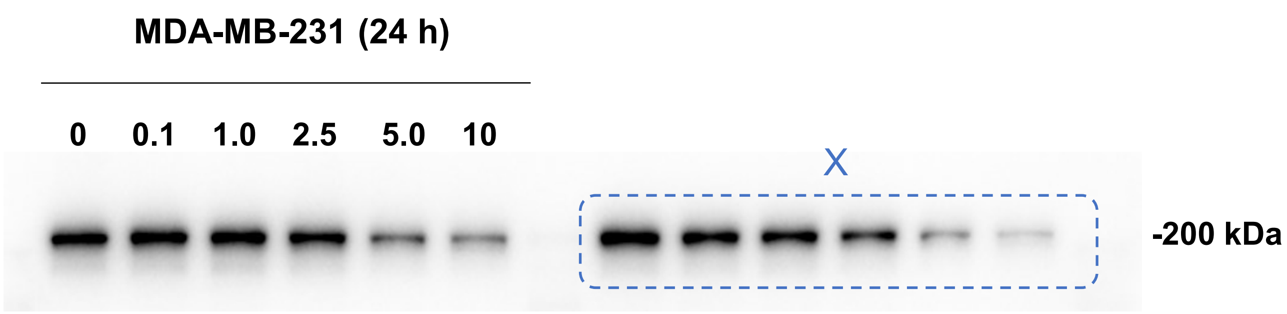

Figure S1 compound **H3** BRD4

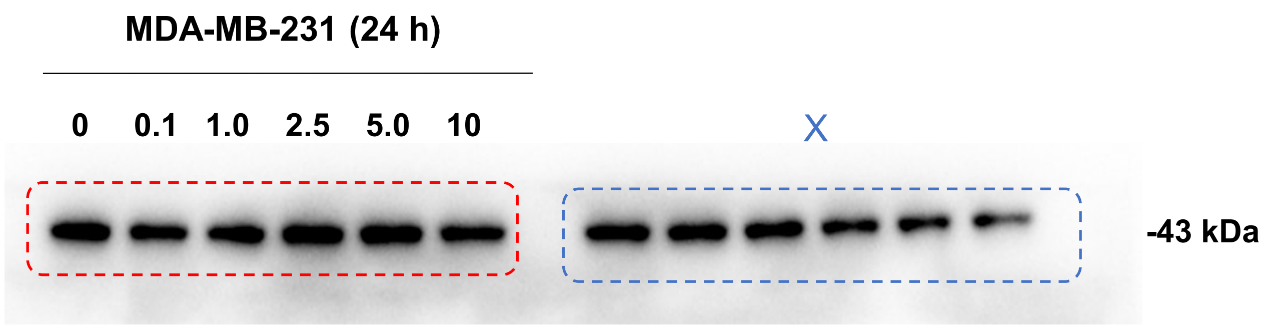

Figure S1 compound **H3** β-Actin

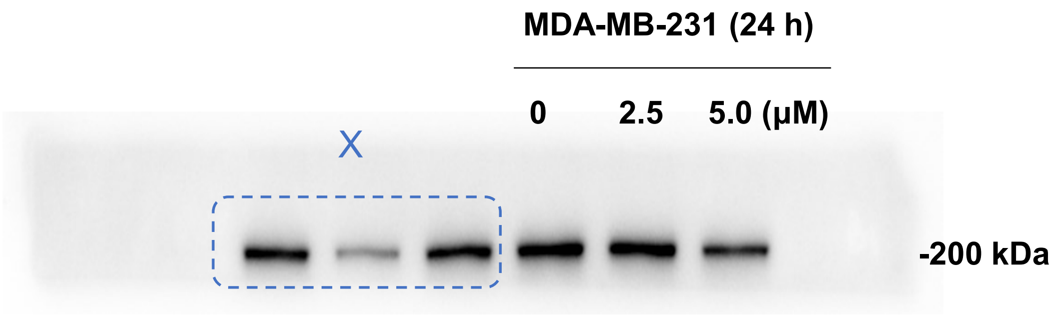

Figure S1 compound **H4** BRD4

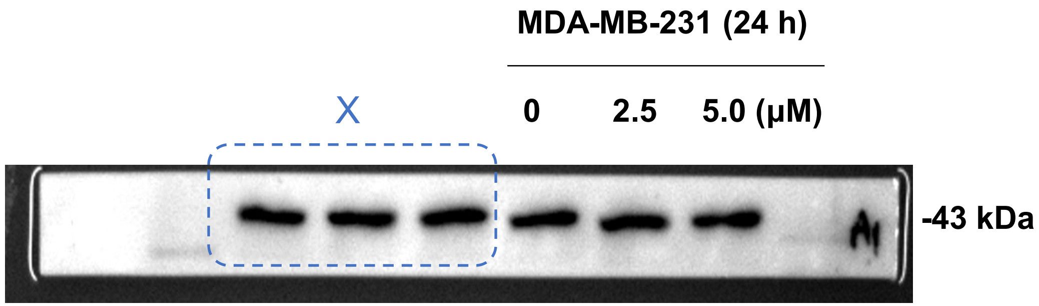

Figure S1 compound **H4** β-Actin

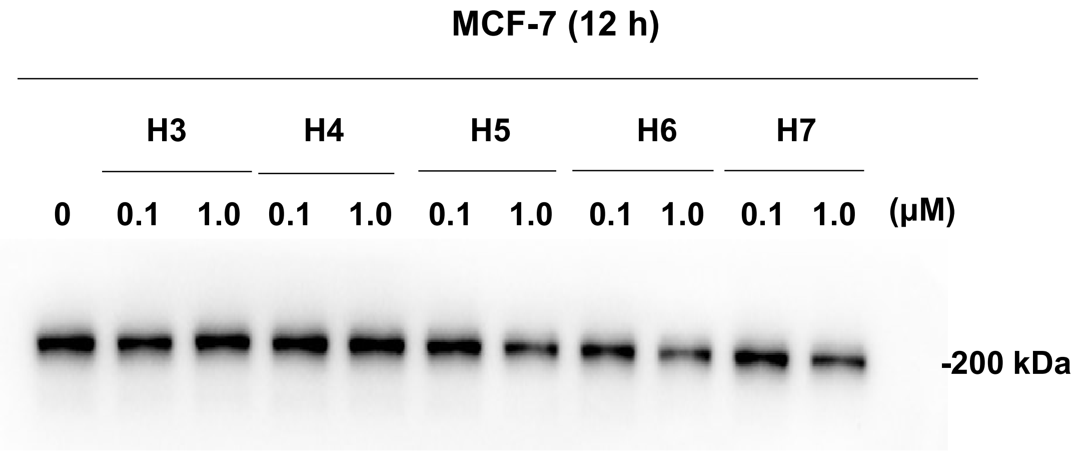

Figure S1 compound **H3-H7** BRD4

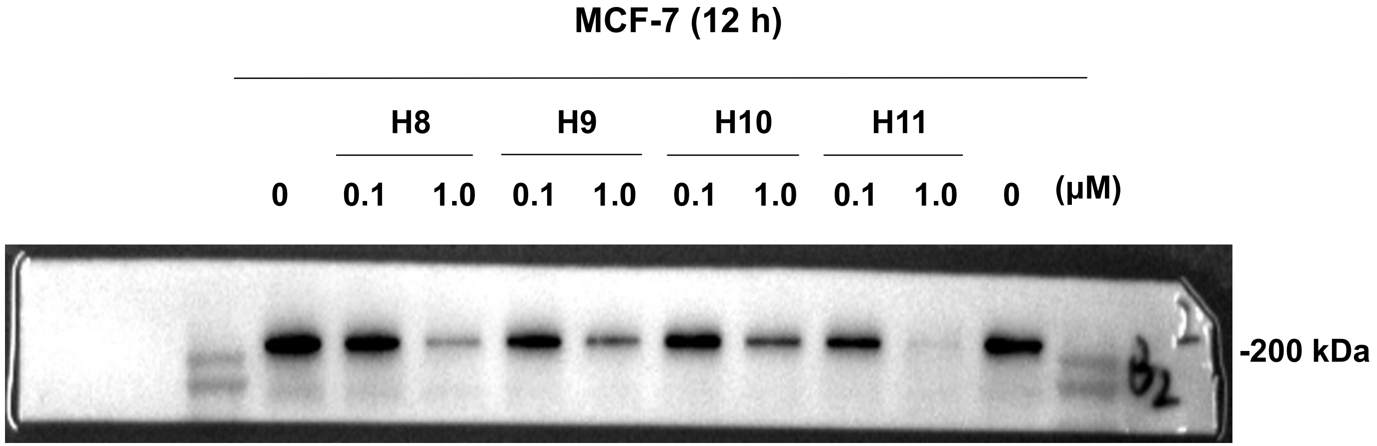

Figure S1 compound **H8-H11** BRD4

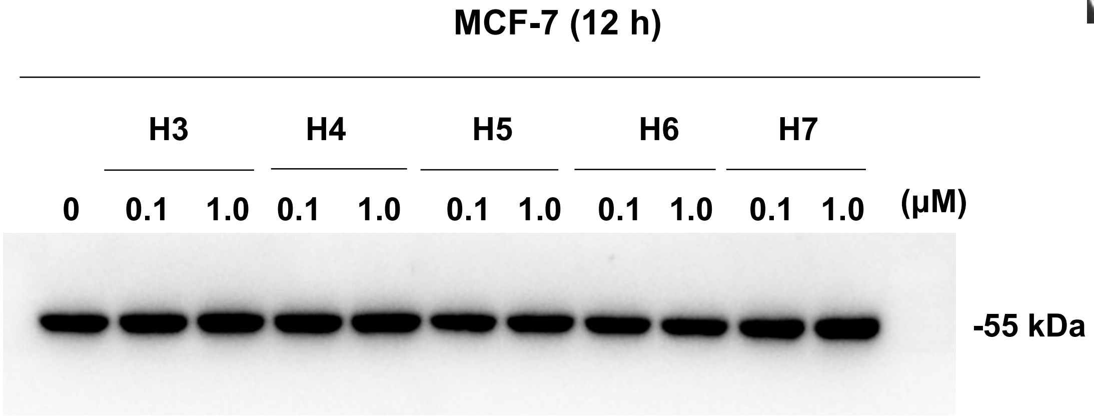

Figure S1 compound **H3-H7** α-Tubulin

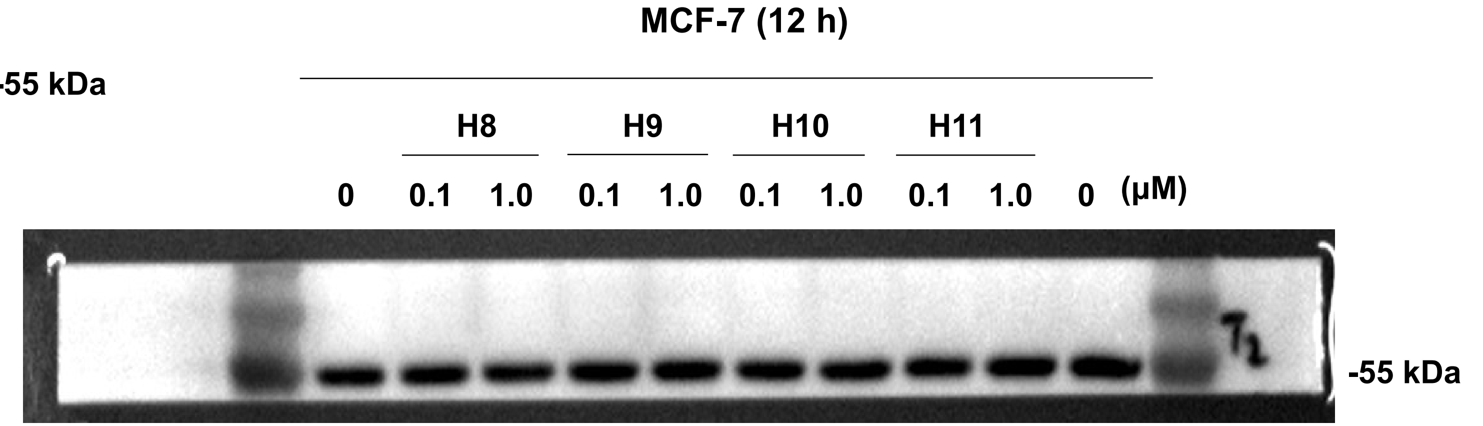

Figure S1 compound **H8-H11** α-Tubulin

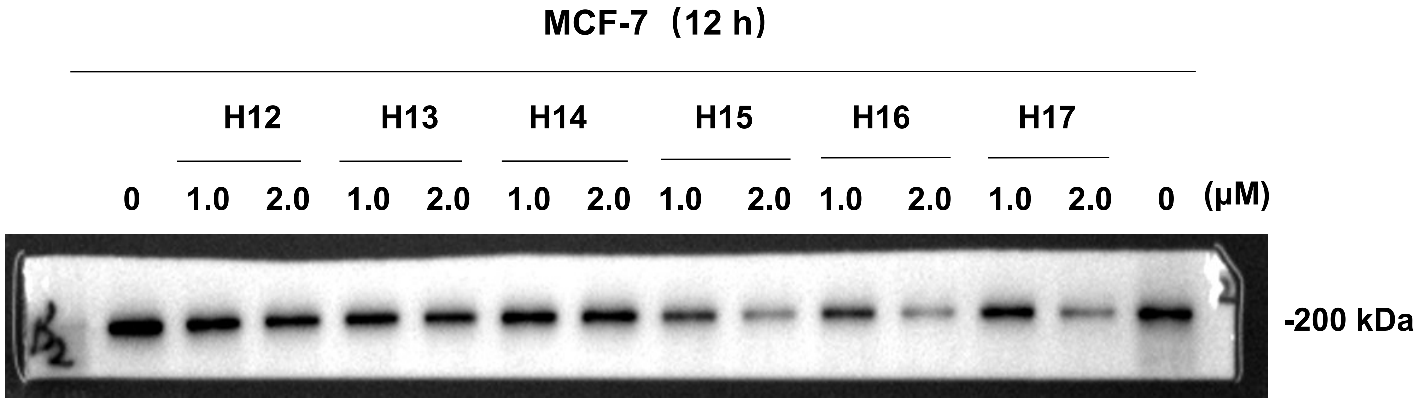

Figure S1 compound **H12-H17** BRD4

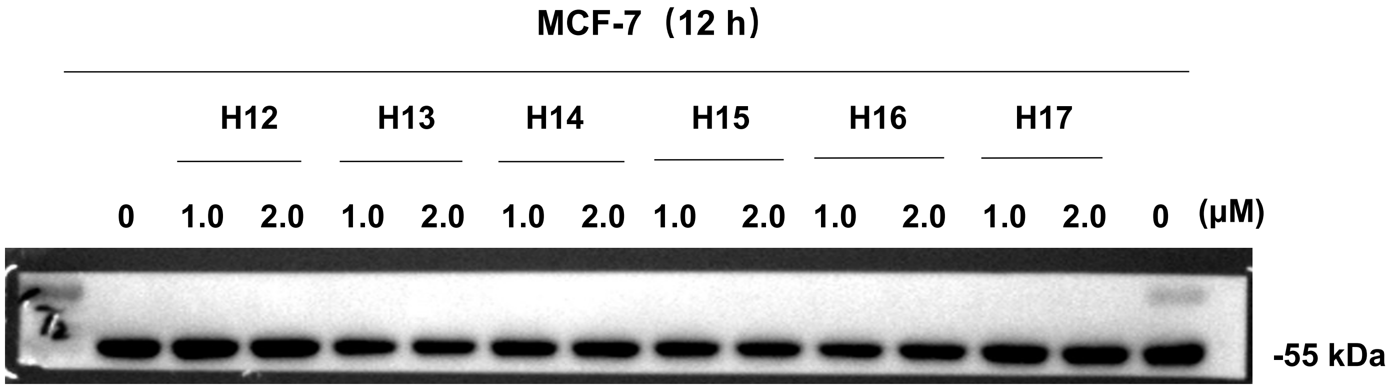

Figure S1 compound **H12-H17** α-Tubulin

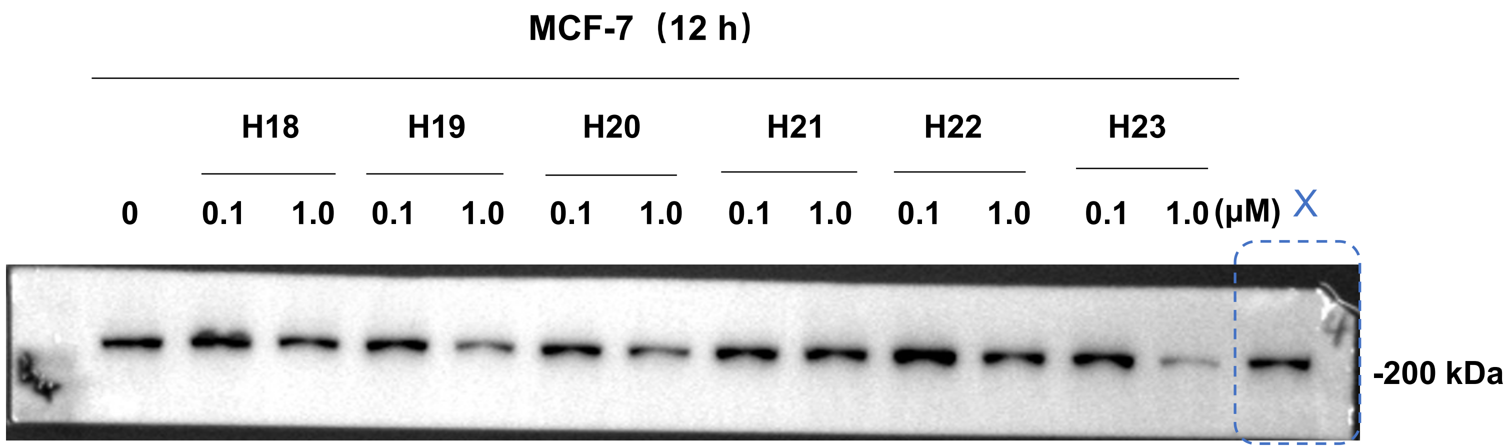

Figure S1 compound **H18-H23** BRD4

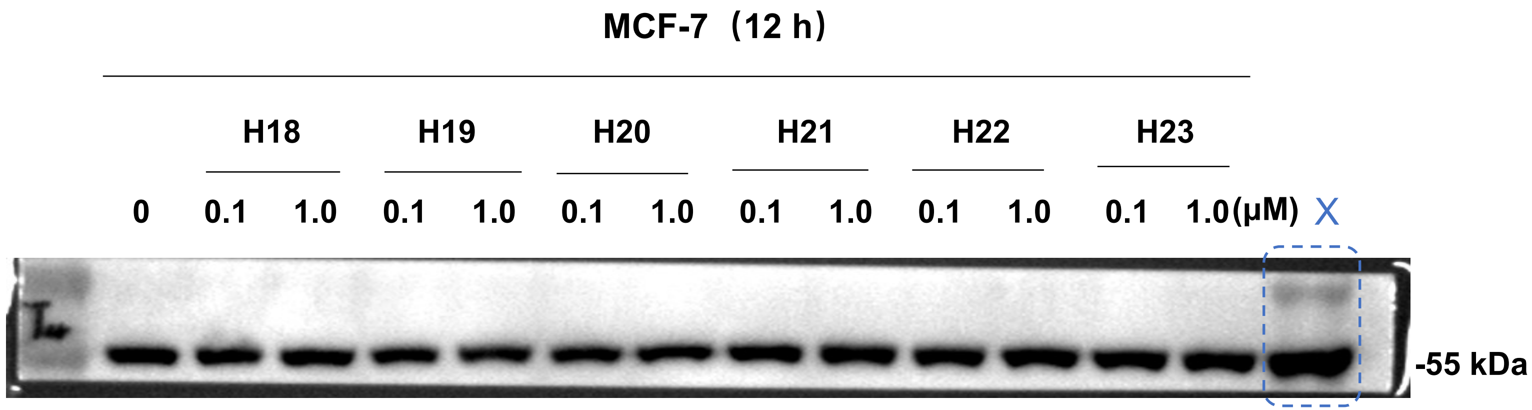

Figure S1 compound **H18-H23** α-Tubulin

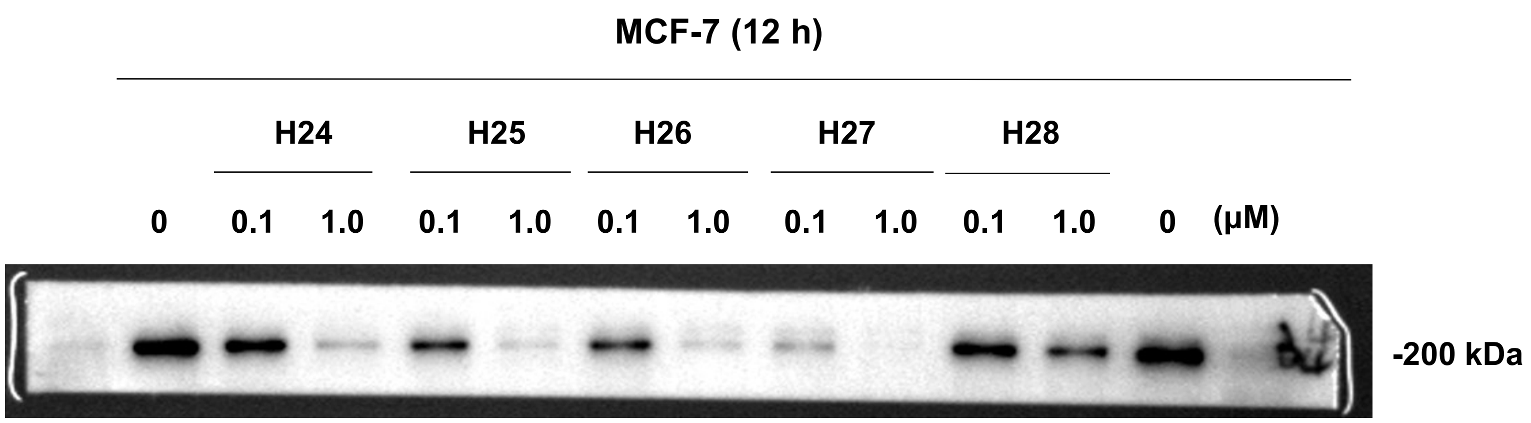

Figure S1 compound **H24-H28** BRD4

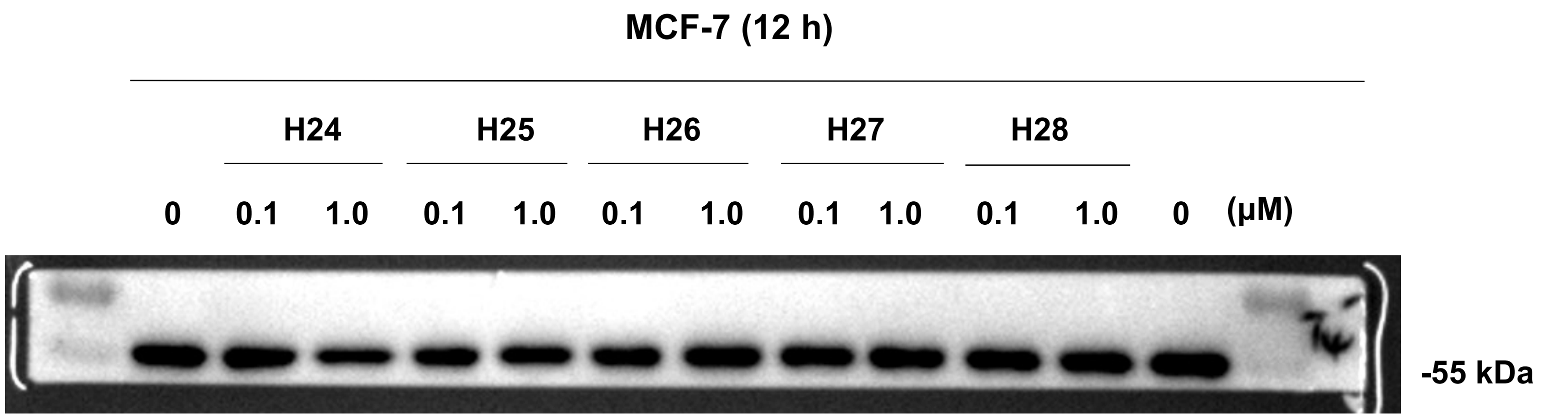

Figure S1 compound **H24-H28** α-Tubulin

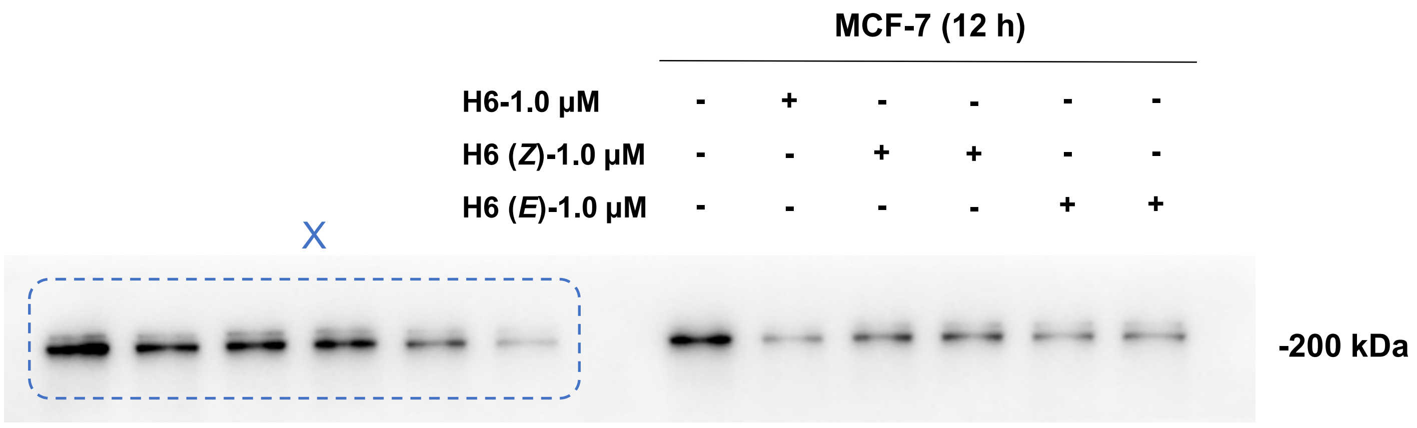

Figure S2 BRD4

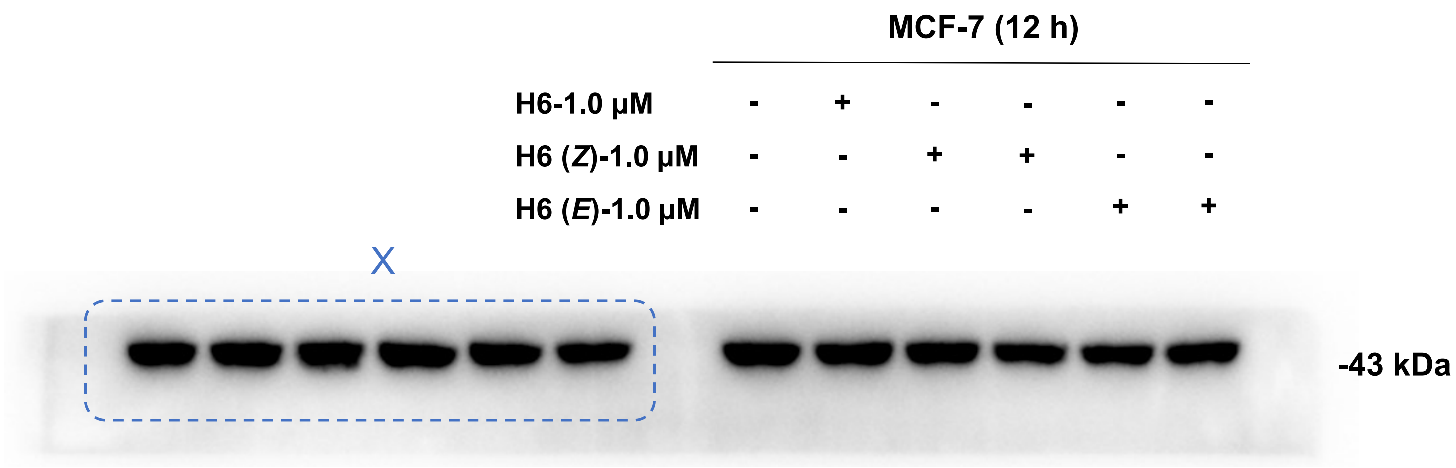

Figure S2 β-Actin

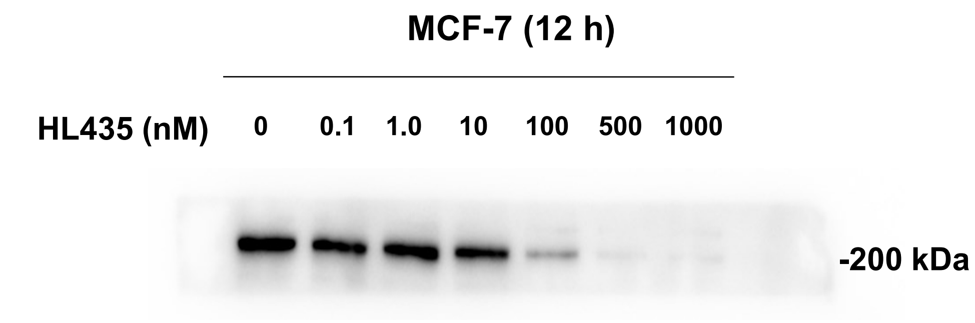

Figure S3A BRD4

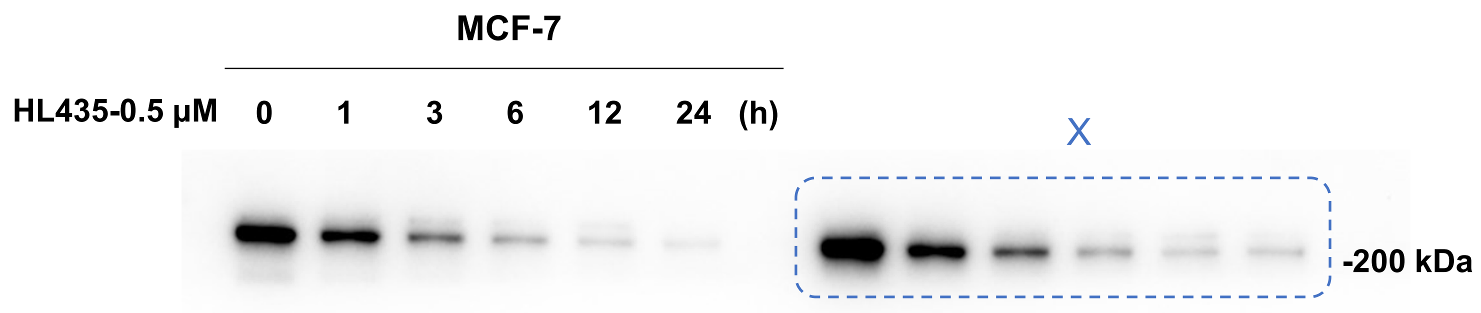

Figure S3B BRD4

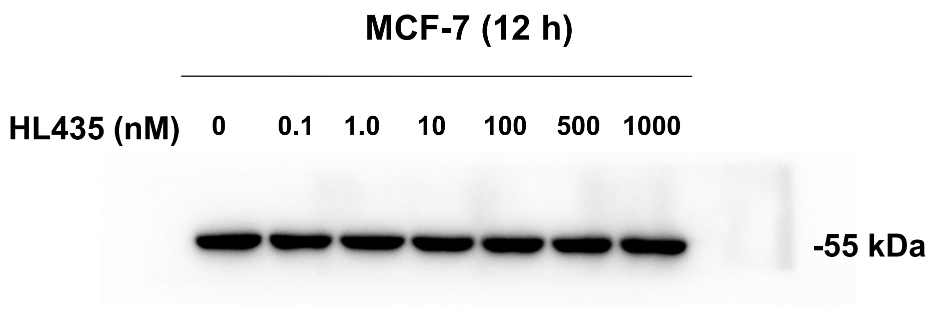

Figure S3A α-Tubulin

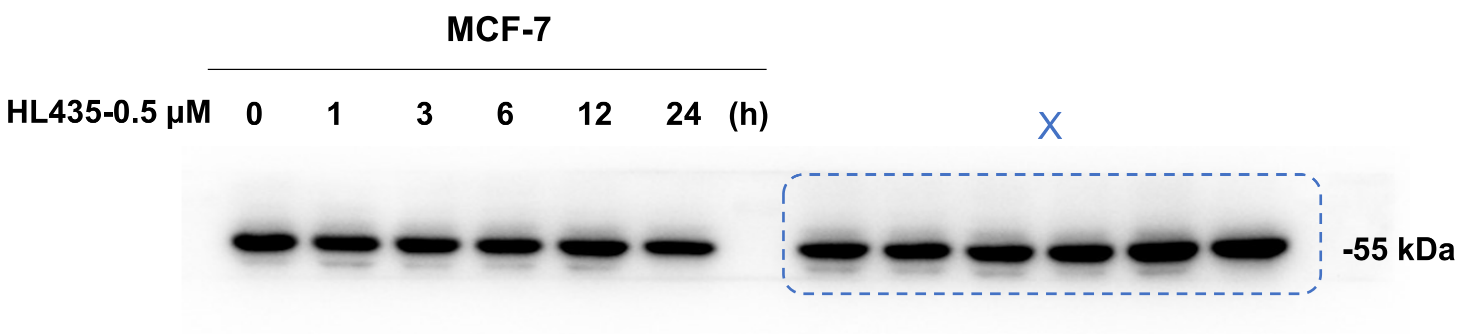

Figure S3B α-Tubulin

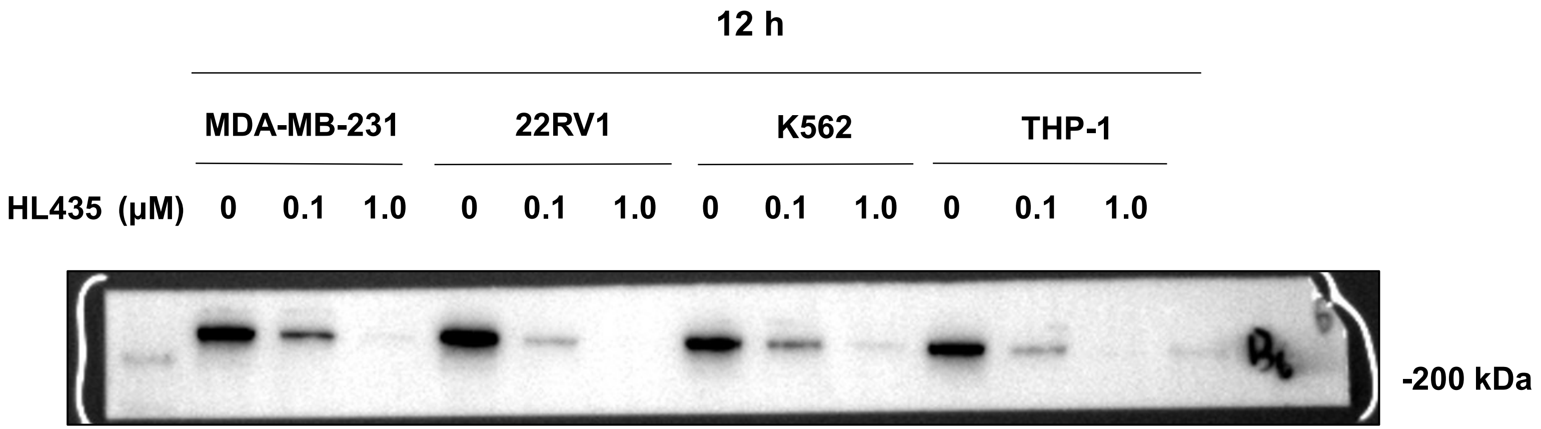

Figure S4 BRD4

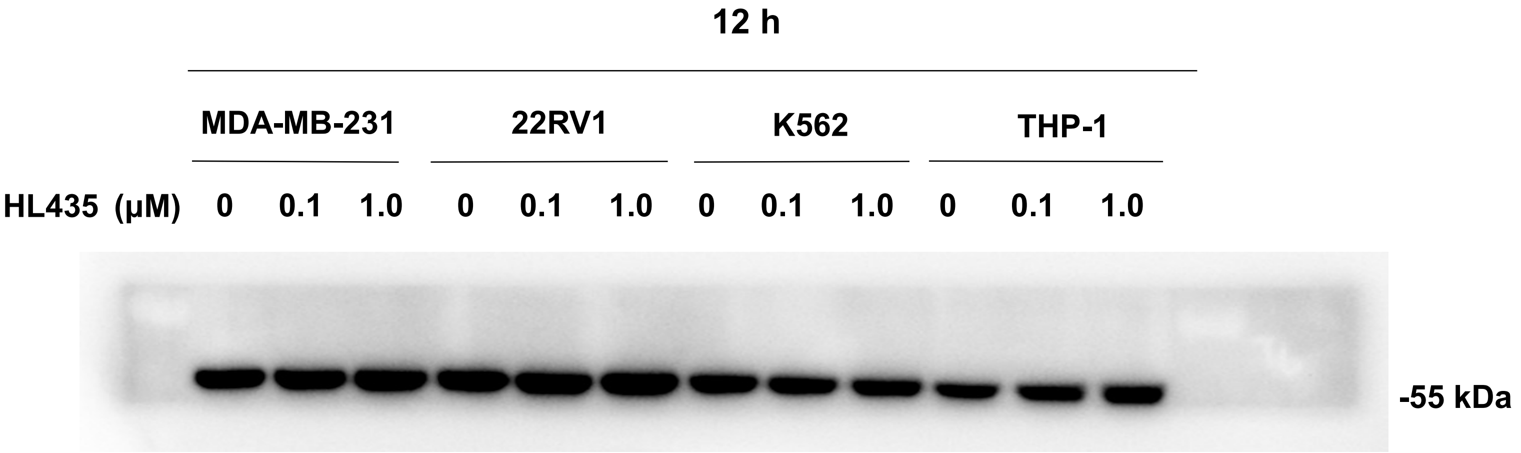

Figure S4 α-Tubulin

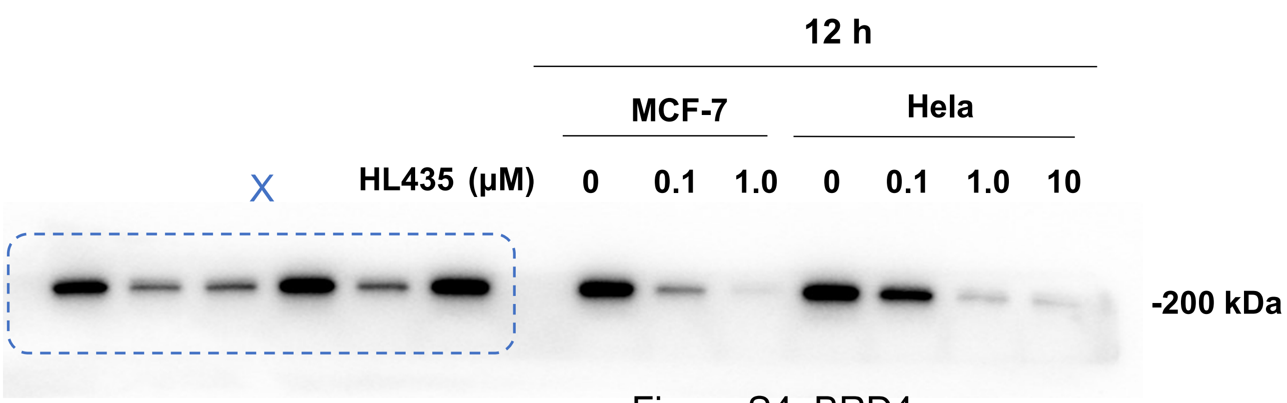

Figure S4 BRD4

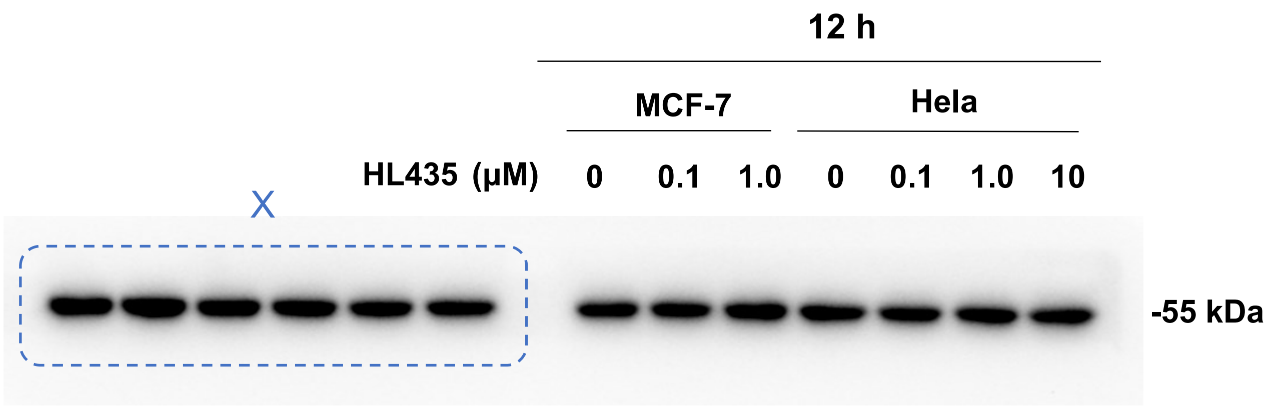

Figure S4 α-Tubulin

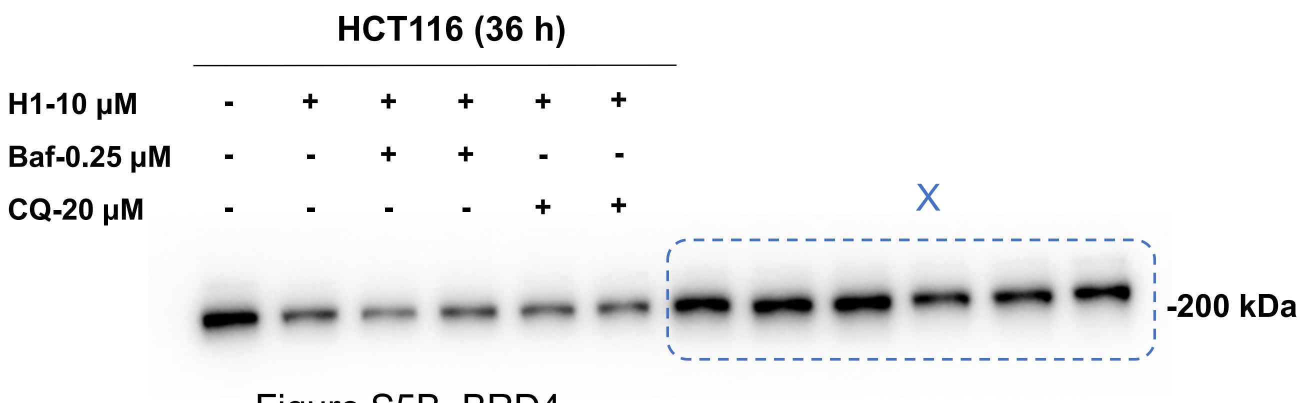

Figure S5B BRD4

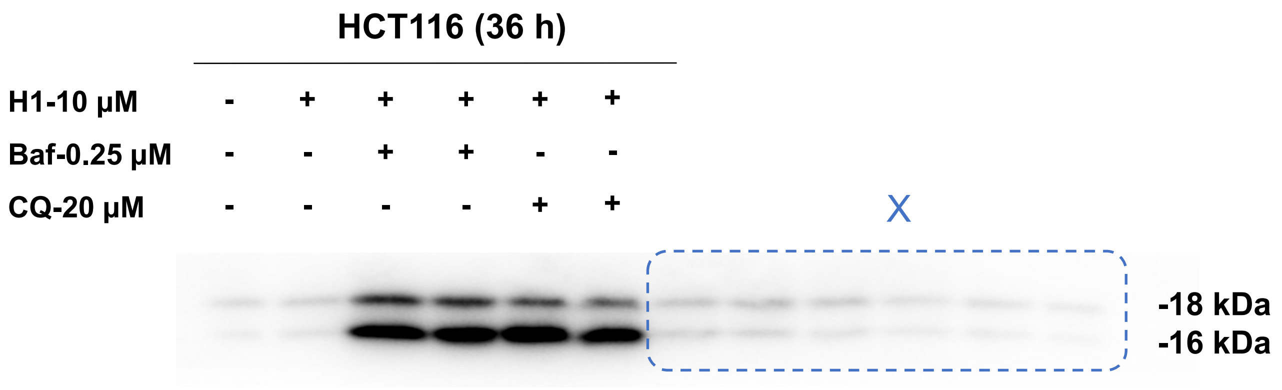

Figure S5B LC3

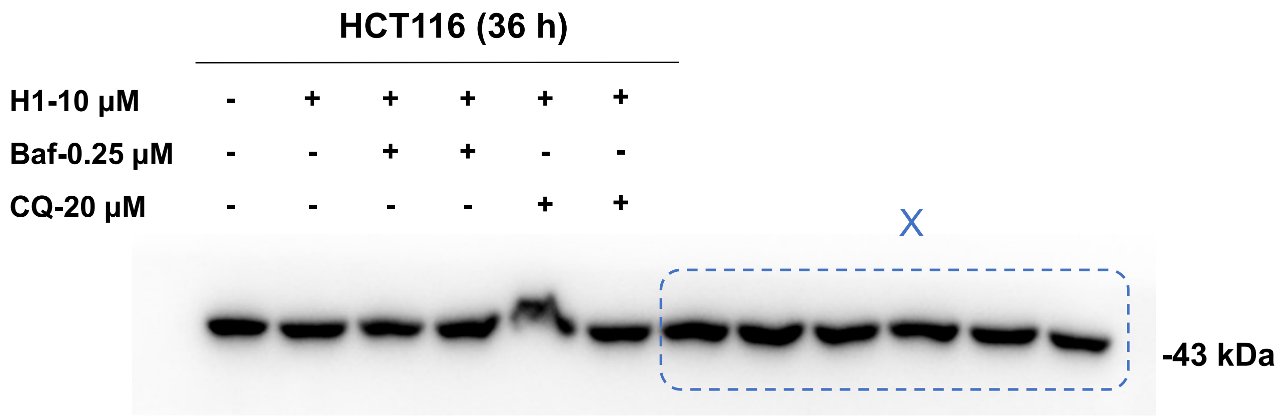

Figure S5B β-Actin

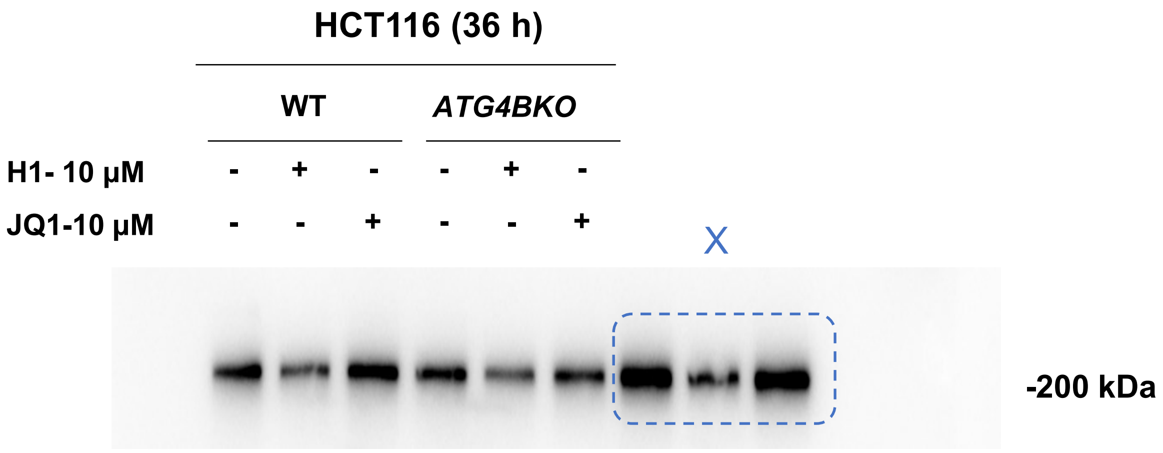

Figure S5C BRD4

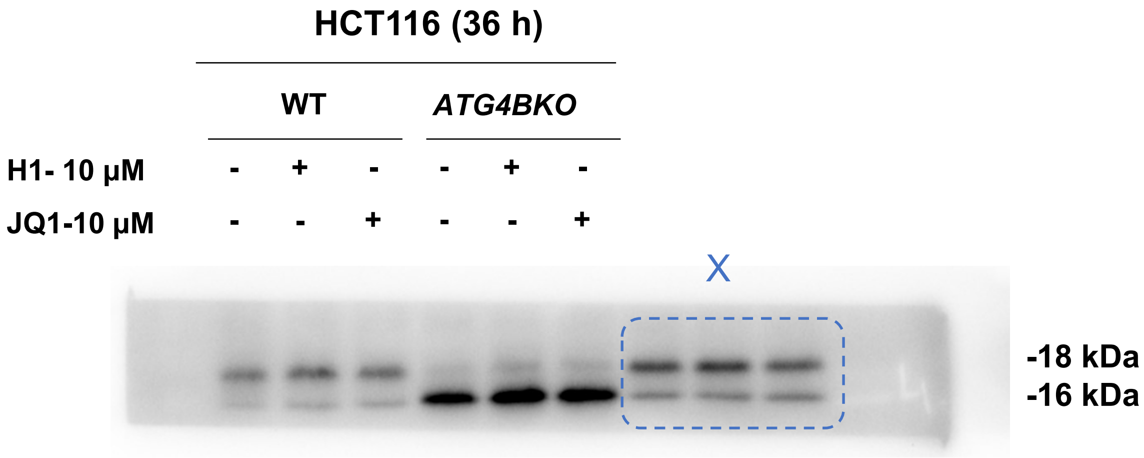

Figure S5C LC3

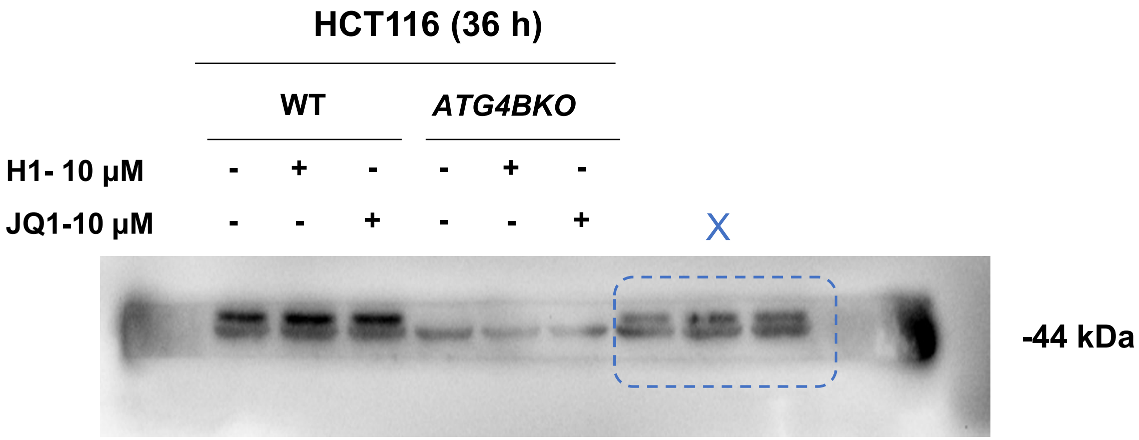

Figure S5C ATG4B

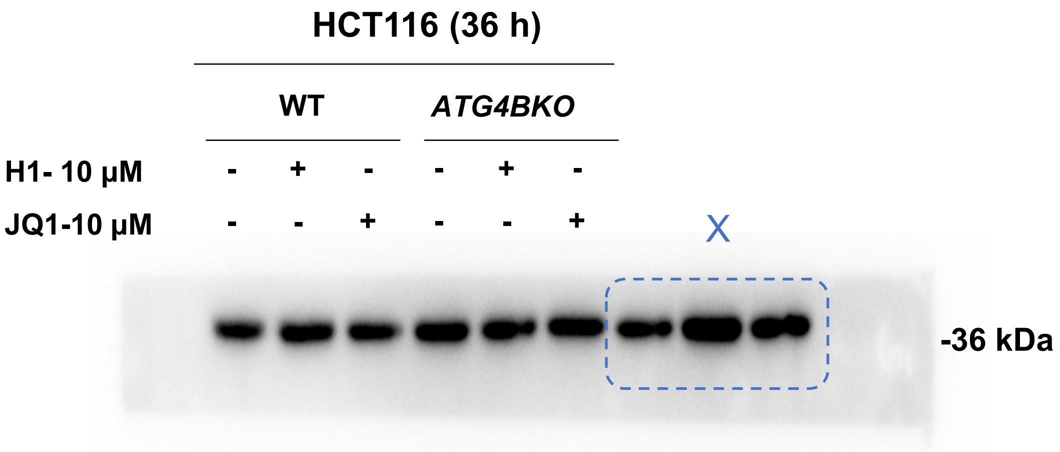

Figure S5C GAPDH

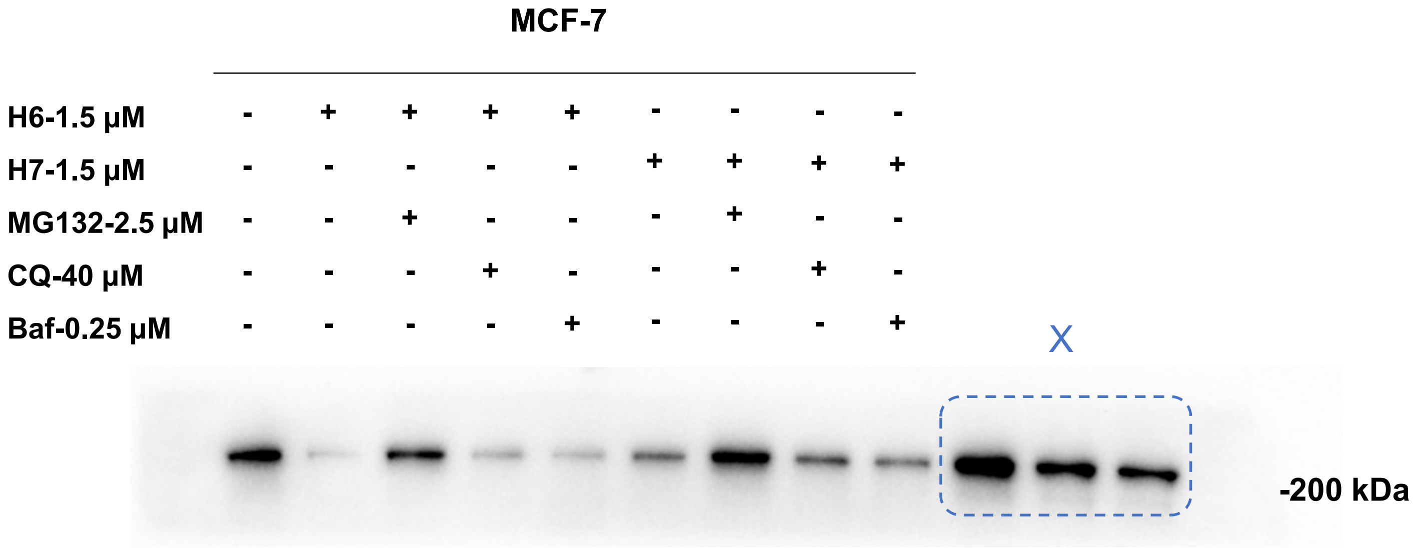

Figure S5D BRD4

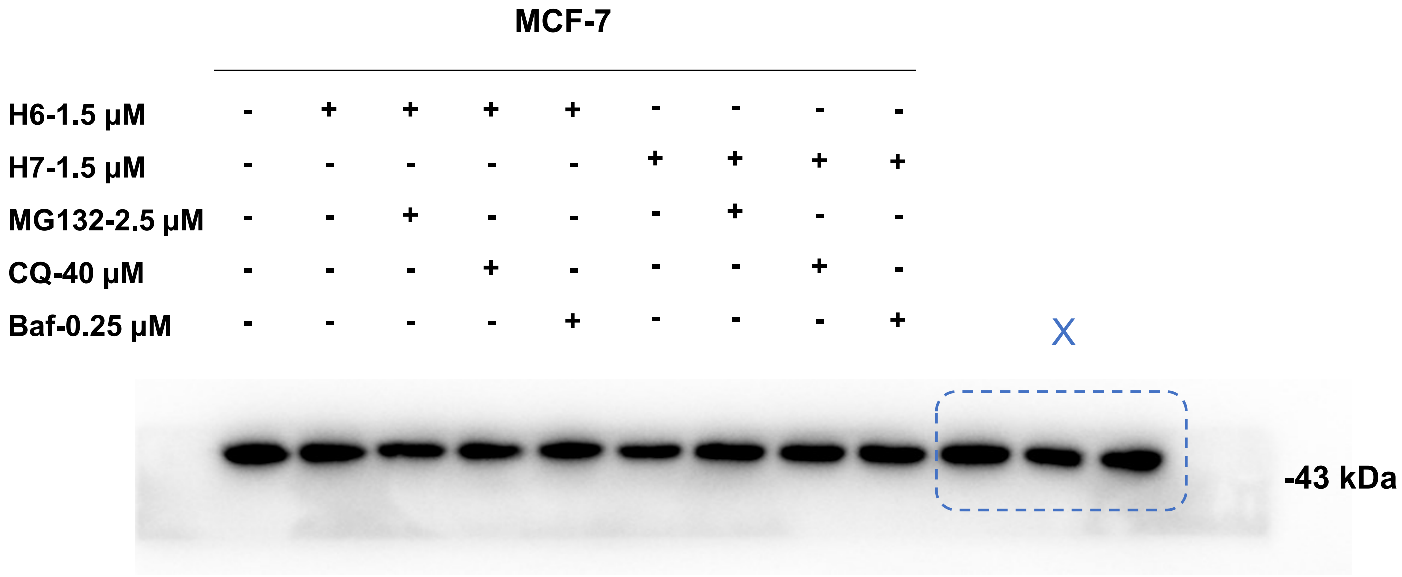

Figure S5D β-Actin

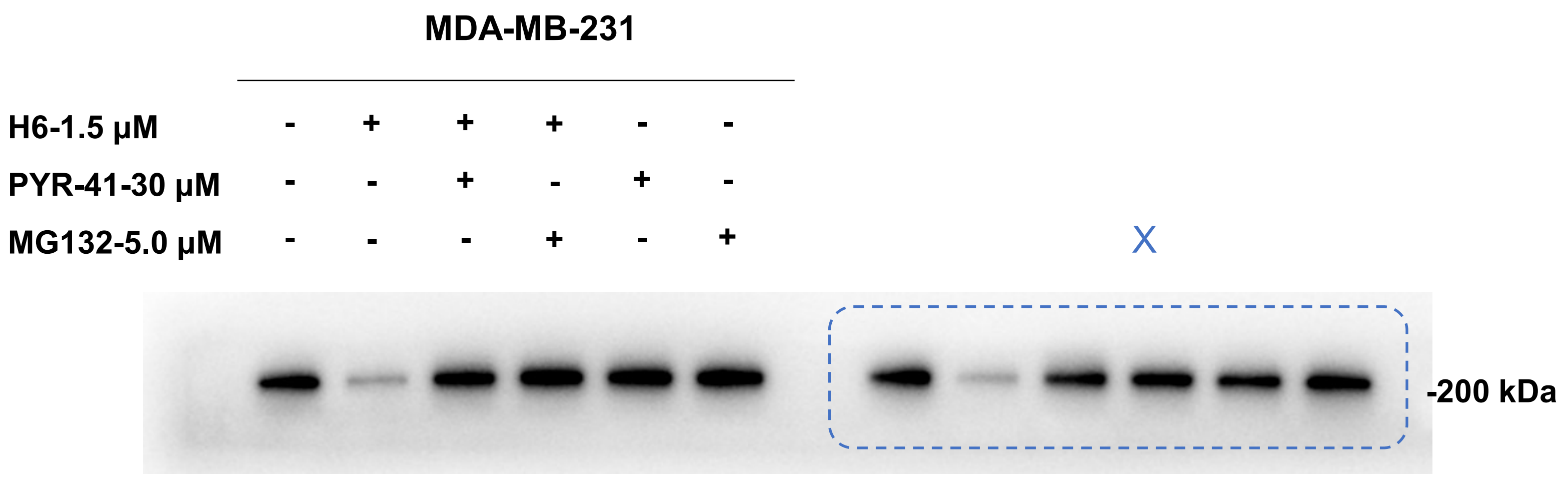

Figure S5E BRD4

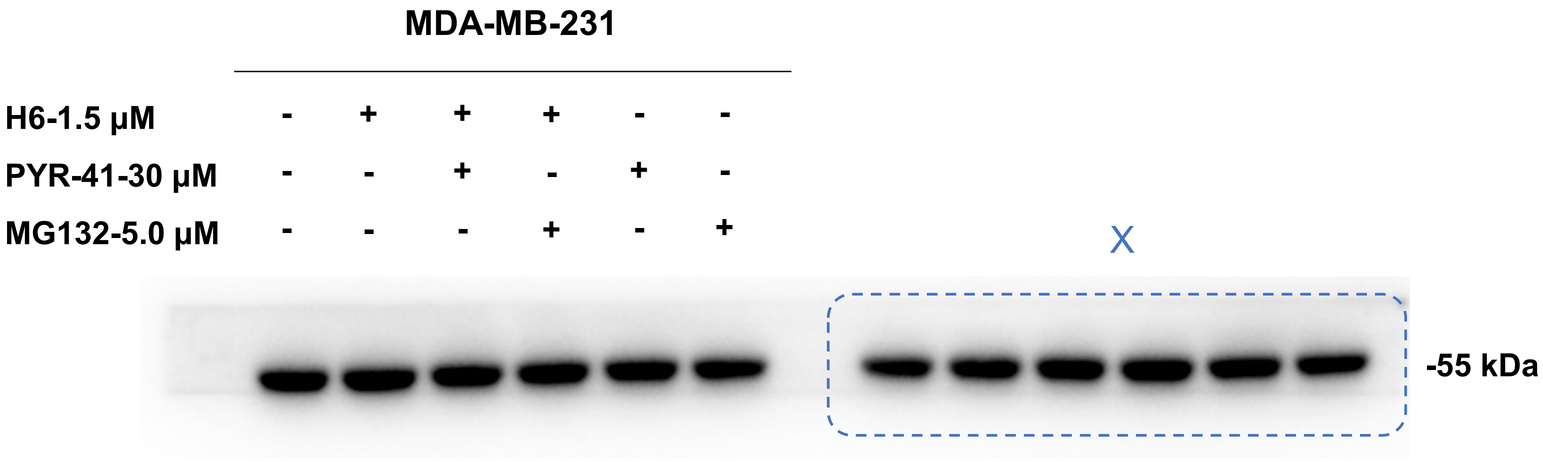

Figure S5E α-Tubulin

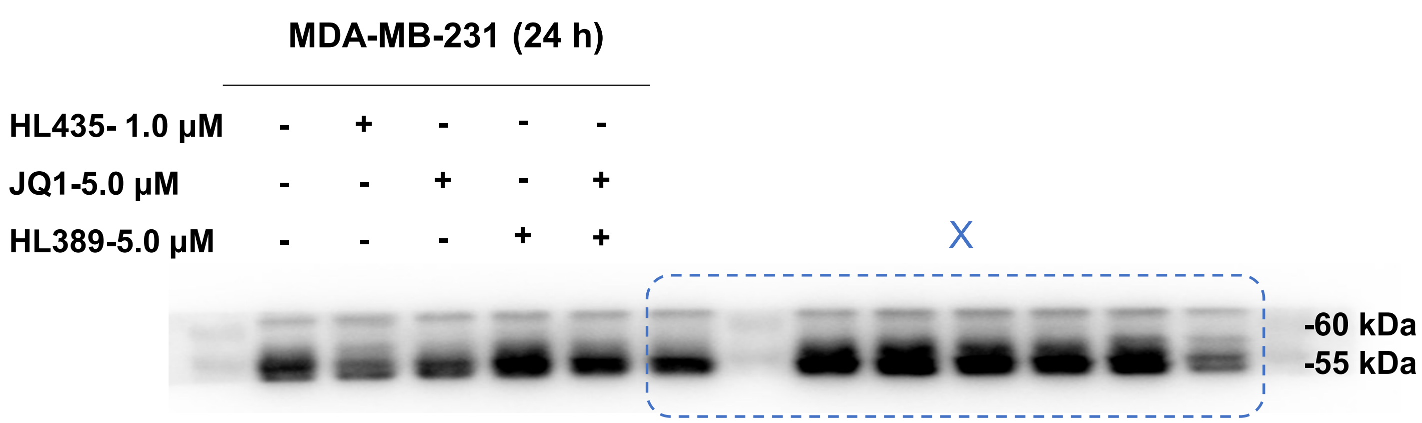

Figure S6C c-Myc

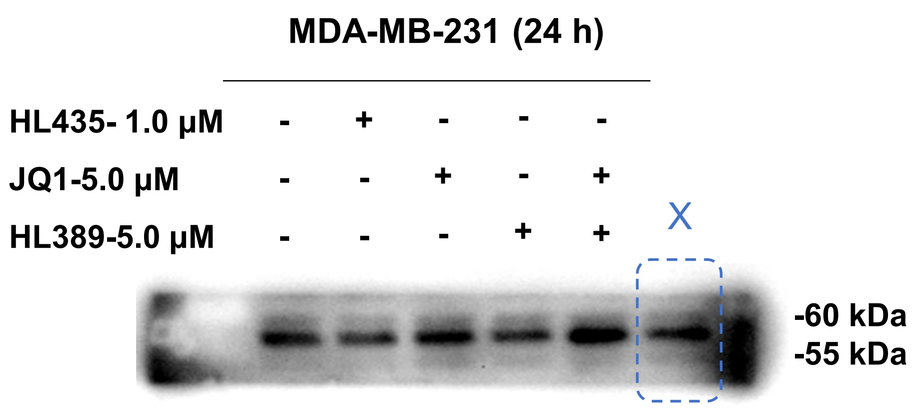

Figure S6C Cyclin B1

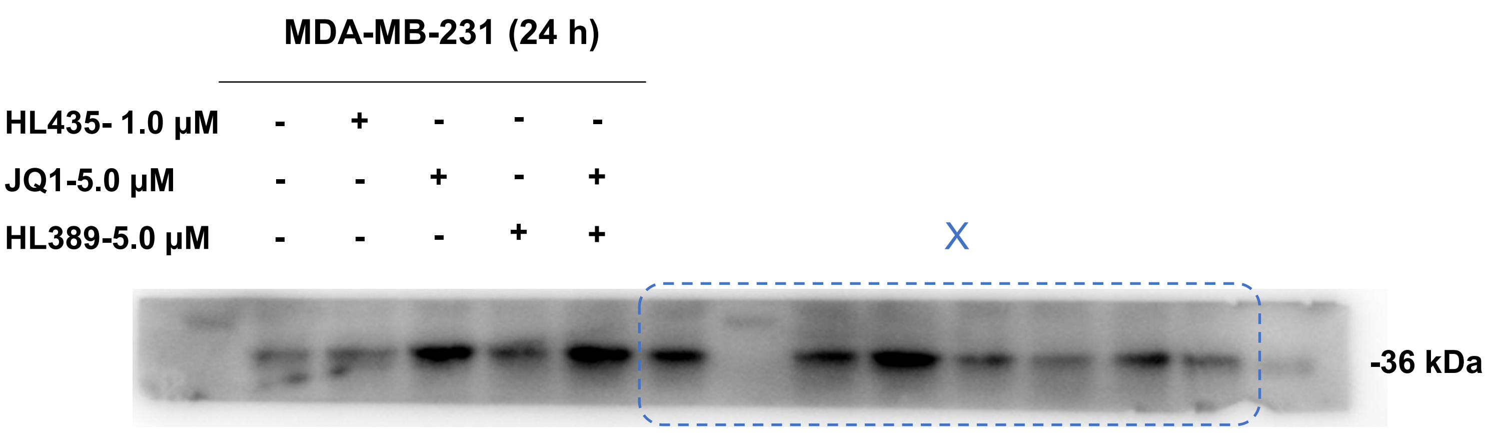

Figure S6C Cyclin D1

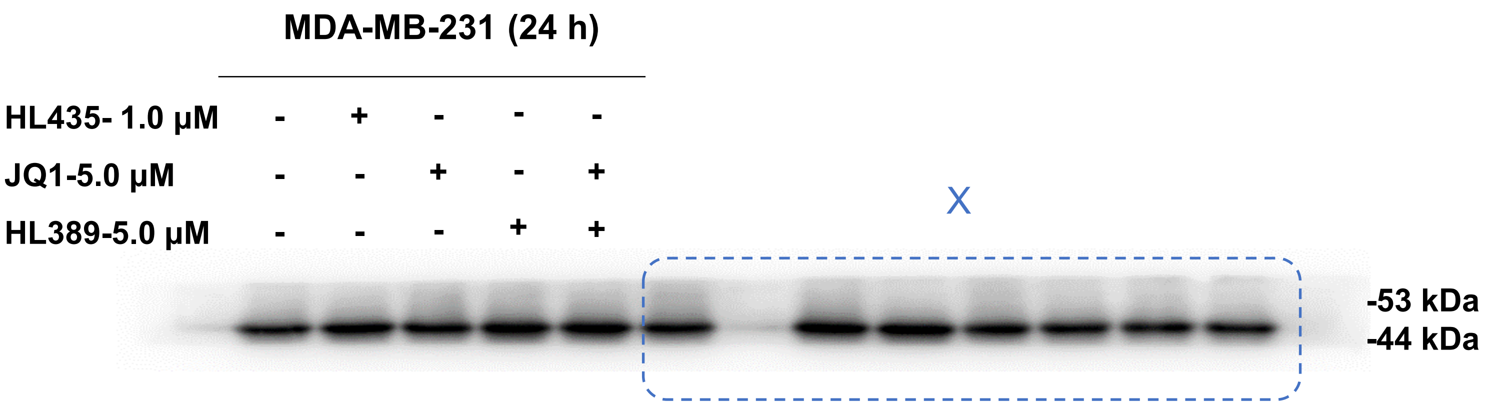

Figure S6C P53

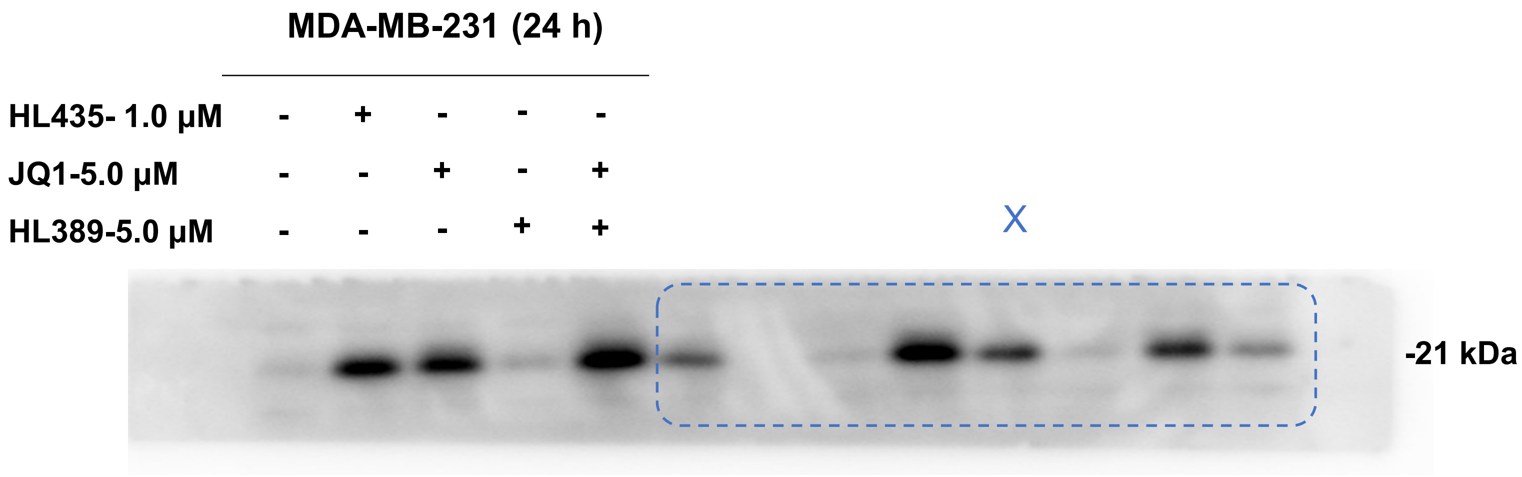

Figure S6C P21

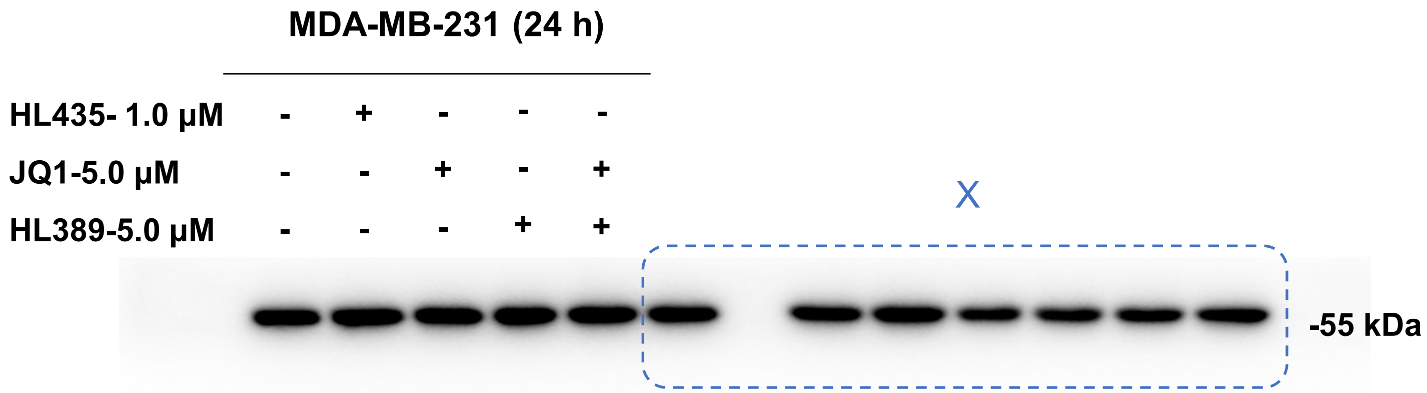

Figure S6C α-Tubulin

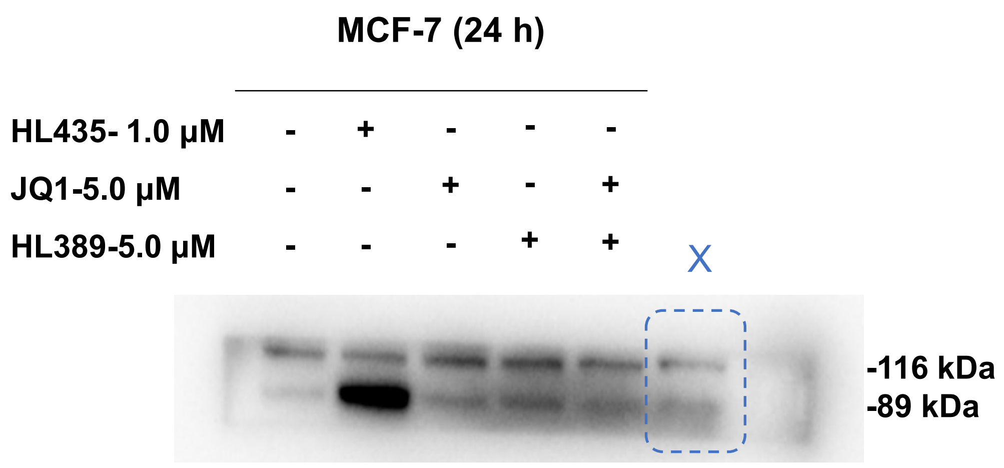

Figure S7B PARP-1

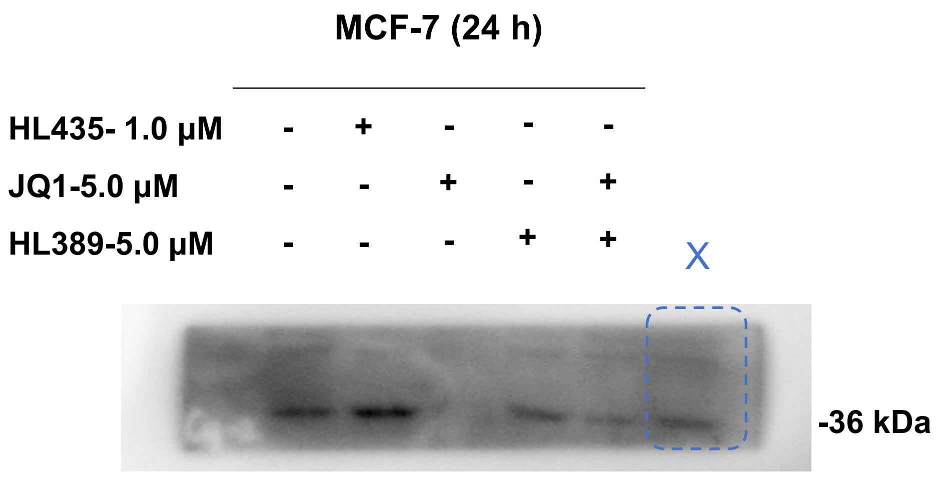

Figure S7B Cl-Caspase-9

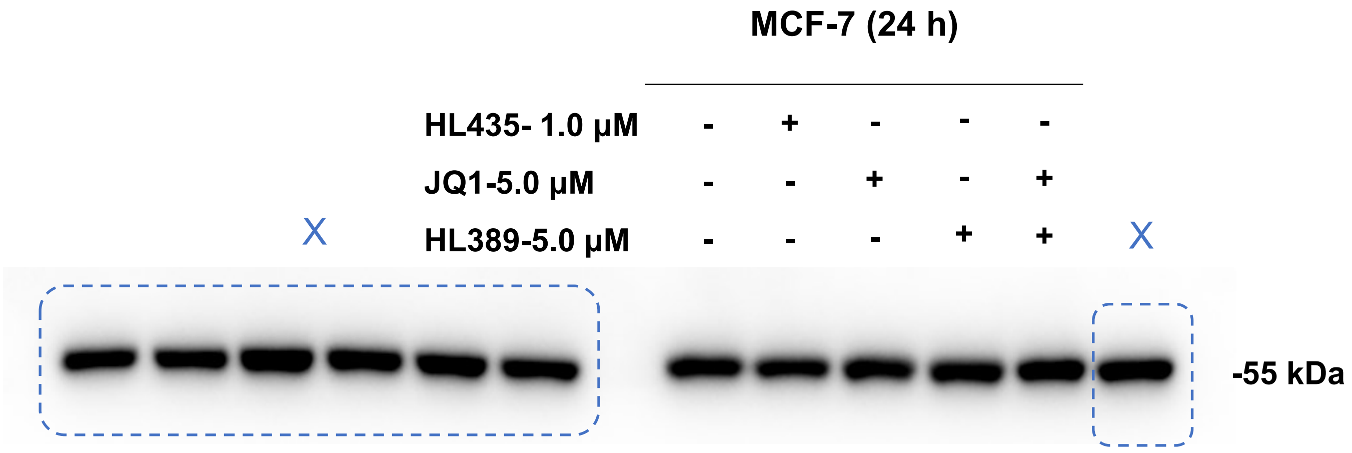

Figure S7B α-Tubulin
